# Microwave-assisted synthesis and antitumor evaluation of a new series of thiazolylcoumarin derivatives

**DOI:** 10.17179/excli2017-208

**Published:** 2017-08-30

**Authors:** Moustafa T. Gabr, Nadia S. El-Gohary, Eman R. El-Bendary, Mohamed M. El-Kerdawy, Nanting Ni

**Affiliations:** 1 Department of Medicinal Chemistry, Faculty of Pharmacy, Mansoura University, Mansoura 35516, Egypt; 2 Department of Chemistry, Georgia State University, Atlanta, Georgia 30303, USA

**Keywords:** thiazolylcoumarins, synthesis, antitumor activity, cytotoxic activity, 3D pharmacophore elucidation, in silico studies

## Abstract

A new series of thiazolylcoumarin derivatives was synthesized. The designed strategy embraced a molecular hybridization approach which involves the combination of the thiazole and coumarin pharmacophores together. The new hybrid compounds were tested for *in vitro* antitumor efficacy over cervical (Hela) and kidney fibroblast (COS-7) cancer cells. Compounds **5f**, **5h**, **5m** and **5r** displayed promising efficacy toward Hela cell line. In addition, **5h **and **5r **were found to be the most active candidates toward COS-7 cell line. The four active analogs, **5f**, **5h**, **5m** and **5r** were screened for *in vivo *antitumor activity over EAC cells in mice, as well as *in vitro* cytotoxicity toward W138 normal cells. Results illustrated that **5r** has the highest *in vivo* activity, and that the four analogs are less cytotoxic than 5-FU toward W138 normal cells. In this study, 3D pharmacophore analysis was performed to investigate the matching pharmacophoric features of the synthesized compounds with trichostatin A. *In silico* studies showed that the investigated compounds meet the optimal needs for good oral absorption with no expected toxicity hazards.

## Introduction

Cancer is a collection of related diseases. Cancer cells can metastasize and invade nearby tissues through blood stream or lymphatic system (Bagi, 2002[4]). In general, cancer develops as a result of genetic changes, such as mutations in DNA. Cancer treatment includes radiation therapy, gene therapy and chemotherapy. Ideal anticancer agents would kill cancer cells without affecting normal tissues. Therefore, the evolution of new safe anticancer agents is a serious task for medicinal chemists. 

Histone deacetylases (HDACs) are Zn^2+ ^dependent enzymes that catalyze the deacetylation of lysine residues located at the N-ε terminal extensions of core histones resulting in chromatin condensation and transcriptional repression (Kouzarides, 2007[36]). Eleven isoforms of HDACs are present in human (Gregoretti et al., 2004[24]). Abnormalities in the deacetylation function of histones were recognized in various human tumors (Falkenberg and Johnstone, 2014[17]).

**Figure 1:**
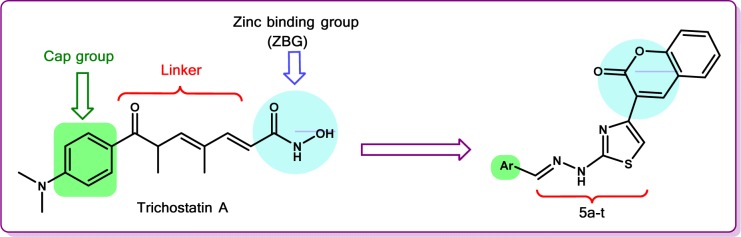
Common pharmacophoric features of trichostatin A and the proposed thiazolylcoumarin hybrids 5a-t

Most HDACs inhibitors share common pharmacophoric features which can be exemplified by trichostatin A, a natural HDACs inhibitor. The common pharmacophore is composed of three regions: zinc binding group (ZBG) that chelates Zn^2+ ^at the active site of the enzyme, cap group which binds to the surface of the active pocket and a linker between the ZBG and the cap group (Feng et al., 2013[18]). Literature survey revealed the significance of variations in the cap group (Bowers et al., 2009[6][7]) and the linker (Weerasinghe et al., 2008[78]) on the HDACs inhibitory activity. However, the type of ZBG is believed to greatly affect the potency and isoform selectivity of HDACs inhibitors (Methot et al., 2008[44]). Hydroxamic acid moiety is a typical ZBG which is common in numerous HDACs inhibitors. Due to the drawbacks of hydroxamate functional group which include non-specific inhibition of all HDAC isoforms (Day and Cohen, 2013[12]), diverse moieties such as thiols, benzamides, sulphamides and trithiocarbonates were incorporated into diverse scaffolds and investigated for their capability as ZBG (Chen et al., 2013[8]; Di Micco et al., 2013[14]; Kawai and Nagata, 2012[34]). Methyl ketone was utilized as ZBG in the design of HDACs inhibitors (Ilies et al., 2011[30]). Analogously, we introduced coumarin moiety as a novel ZBG in the design of new HDACs inhibitors aiming to explore its effect as a non-hydroxamate functional group. The hydrazinylthiazole in the synthesized hybrids is implied as a linker which projects the ZBG into the active site of HDACs. The cap region in the common pharmacophore has a strong contribution to the overall binding affinity of HDACs inhibitors (Salisbury and Cravatt, 2007[60]). The common pharmacophoric features of trichostatin A and the proposed thiazolylcoumarin hybrids are illustrated in Figure 1(Fig. 1). 

Literature revealed that numerous HDACs inhibitors have antitumor activity (Zain et al., 2010[82]). Most of the reported HDACs inhibitors are hydroxamic acid derivatives that exhibit non-specific inhibition of all HDAC isoforms (Day and Cohen, 2013[12]). As a result, extensive research is directed toward the development of non-hydroxamate HDACs inhibitors (Madsen et al., 2014[42]). 

Coumarins of natural and synthetic origins constitute an important class of compounds. They were proved to possess significant therapeutic potential, including antitumor activity (Morsy et al., 2017[45]; Emami and Dadashpour, 2015[16]; Klenkar and Molnar, 2015[35]; Amin et al., 2015[3]; Pingaew et al., 2014[54]; Sandhu et al., 2014[61]; Liu et al., 2014[39]; Li et al., 2014[37]; Seidel et al., 2014[63]; Sashidhara et al., 2010[62]; Riveiro et al., 2010[56]). On the same line, thiazole ring is a prominent skeleton in various bioactive molecules, including antitumor compounds (Tay et al., 2017[71]; Gomha et al., 2015[23]; Abouzeid and El-Subbagh, 2015[2]; Nofal et al., 2014[47]; Rouf and Tanyeli, 2015[59]; Prashanth et al., 2014[55]; Yuan et al., 2014[81]; Shitre et al., 2014[65]; Tung et al., 2013[73]).

Moreover, literature survey indicated that thiazolylcoumarin hybrids (Abdul Rahman et al., 2016[1]; Vaarla et al., 2015[75]; Sreekanth et al., 2014[67]; Srimanth et al., 2002[68]) and other aryl(heteroaryl)coumarin hybrids (Zhang et al., 2017[83]; Pangal et al., 2017[50]; Luo et al., 2017[40]; Garazd et al., 2017[20]; Holiyachi et al., 2016[28]; Goel et al., 2015[22]; Zhang et al., 2014[84]; Kamal et al., 2009[33]; Ganina et al., 2008[19]) have promising antitumor activity. 

Hybrid approaches in drug design proved to offer advantages in drug-resistance (Hubschwerlen et al., 2003[29]), introducing compounds with improved biological activity (Pingaew et al., 2014[53]) as well as their contribution in the development of promising agents with potent antitumor activity (Piens et al., 2014[52]; Romagnoli et al., 2013[58]). Therefore, we followed the hybridization strategy, combining the thiazole and coumarin pharmacophores together hoping to obtain new safe antitumor compounds. In addition, the design strategy embraced the profiling of diverse aromatic moieties (representing the cap group) on the thiazolylcoumarin scaffold in order to study the relationship between the interaction forces of the cap group to the target receptor and the antitumor activity of the proposed thiazolylcoumarin hybrids. The new hybrid compounds were assessed for *in vitro* antitumor activity, and the four active analogs, **5f**, **5h**, **5m** and **5r** were screened for *in vivo* antitumor activity over EAC in mice, as well as *in vitro* cytotoxicity toward W138 normal cells. HDACs inhibitory activity of the new active compounds is a plausible mechanism that might shed light toward the discovery of a new class of HDACs inhibitors. 

## Materials and Methods

### Chemistry

Stuart SMP10 melting point apparatus was utilized to determine melting points °C. Bruker Avance 400 MHz spectrometer was applied for recording ^1^H and ^13^C NMR spectra; chemical shifts are expressed in *δ* ppm with reference to TMS (Georgia State University, USA). HRMS were obtained on nano LC-Q-TOF spectrometer in +ve or -ve ion mode (Georgia State University, USA). Elemental analyses (C, H, N) were determined, and were within ± 0.4% of the calculated values (Georgia State University, USA). The completion of reactions was controlled utilizing TLC plates (silica gel 60 F254, Merck) and UV (366 nm) was used for visualization of the spots. Chloroform/methanol (9:1) and *n*-hexane/ethyl acetate (3:1) were utilized as elution solvents.

**Synthesis of 3-acetylcoumarin (2):** Salicylaldehyde **(1)** (2.20 g, 18 mmol), ethyl acetoacetate (3.12 g, 24 mmol) and piperidine (0.1 mL) were heated in ethanol (5 mL) under microwave irradiation (50 W) at 45 °C for 5 min. The precipitated solid upon cooling was filtered and crystallized from ethanol. Yield 85%, m.p. 117-118 °C (Valizadeh et al., 2007[76]). ^1^H NMR (DMSO-*d_6_*) *δ* 2.40 (s, 2H, CH_2_), 7.40-8.00 (m, 4H, Ar-H), 8.80 (s, 1H, C_4_-H of chromone). 

**Synthesis of 3-(bromoacetyl)coumarin (3):** A solution of bromine (1.60 g, 20 mmol) was added dropwise with constant stirring to a solution of compound **2** (2 g, 11 mmol) in chloroform (15 mL). The mixture was stirred at 0-5 °C for 6 hrs and the orange solid obtained was filtered and crystallized from glacial acetic acid. Yield 63%, m.p. 161-162 °C (Siddiqui et al., 2009[66]). ^1^H NMR (DMSO-*d_6_*) *δ* 4.90 (s, 2H, CH_2_), 7.40-8.00 (m, 4H, Ar-H), 8.80 (s, 1H, C_4_-H of chromone).

**Synthesis of 2-arylidenehydrazinocarbothioamides 4a-t: **Thiosemicarbazide (0.092 g, 1 mmol), aromatic aldehyde (1 mmol) and glacial acetic acid (0.1 mL) were heated in ethanol (10 mL) under microwave irradiation (50 W) at 80 °C for 10 min. The precipitate formed upon cooling was filtered and crystallized to afford **4a-t**.

**2-(2-Bromobenzylidene)hydrazinocarbothioamide (4a): **Yield 84%, m.p. 202-203 °C (Coxon et al., 2013[10]), (ethyl acetate/ethanol (3:1)), C_8_H_8_BrN_3_S. ^1^H NMR (DMSO-*d_6_*) *δ* 7.25-8.45 (m, 7H, Ar-H, NH_2_, CH=N), 11.65 (s, 1H, NH). ^13^C NMR (DMSO-*d_6_*) *δ* 124.0, 128.1, 128.2, 131.8, 133.3, 133.4, 141.2, 178.6.

**2-(2-Cyanobenzylidene)hydrazinocarbothioamide (4b): **Yield 91%, m.p. 212-213 °C (Hernandez et al., 2010[26]), (chloroform), C_9_H_8_N_4_S. ^1^H NMR (DMSO-*d_6_*) *δ* 7.60-8.55 (m, 7H, Ar-H, CH=N, NH_2_), 11.85 (s, 1H, NH).

**2-(3-Cyanobenzylidene)hydrazinocarbothioamide (4c): **Yield 82%, m.p. 204-205 °C (Hernandez et al*.*, 2008[27]), (ethanol/water (2:1)), C_9_H_8_N_4_S. ^1^H NMR (DMSO-*d_6_*) *δ* 7.55-8.45 (m, 7H, Ar-H, CH=N, NH_2_), 11.60 (s, 1H, NH). ^13^C NMR (DMSO-*d_6_*) *δ* 112.1, 116.6, 130.2, 132.1, 133.7, 134.1, 134.6, 144.3, 177.9.

**2-(4-(Trifluoromethyl)benzylidene)hydrazinocarbothioamide (4d): **Yield 79%, m.p. 169-170 °C (Bernstein et al., 1951[5]), (ethyl acetate/ethanol (3:1)), C_9_H_8_F_3_N_3_S. ^1^H NMR (DMSO-*d_6_*) *δ* 7.75-8.45 (m, 7H, Ar-H, NH_2_, CH=N), 11.65 (s, 1H, NH). ^13^C NMR (DMSO-*d_6_*) *δ* 123.2, 125.8, 128.2, 130.0, 138.6, 144.2, 178.8.

**2-(3-Methylbenzylidene)hydrazinocarbothioamide (4e): **Yield 79%, m.p. 190-191 °C (Lv et al., 2010[41]), (methanol), C_9_H_11_N_3_S. ^1^H NMR (DMSO-*d_6_*) *δ* 2.45 (s, 3H, CH_3_), 7.15-8.45 (m, 7H, Ar-H, NH_2_, CH=N), 11.40 (s, 1H, NH). ^13^C NMR (DMSO-*d_6_*) *δ* 23.6, 125.7, 127.9, 129.2, 130.9, 133.6, 136.5, 144.1, 179.2.

**2-(2,6-Dichlorobenzylidene)hydrazinocarbothioamide (4f): **Yield 81%, m.p. 236-238 °C (Bernstein et al., 1951[5]), (ethanol/water (2:1)), C_8_H_7_Cl_2_N_3_S. ^1^H NMR (DMSO-*d_6_*) *δ* 7.30-8.50 (m, 6H, Ar-H, CH=N, NH_2_), 11.75 (s, 1H, NH).

**2-(2-Chloro-6-fluorobenzylidene)hydrazinocarbothioamide (4g): **Yield 85%, m.p. 241-242 °C (Sumangala et al., 2012[70]), (ethyl acetate/ethanol (3:1)), C_8_H_7_ClFN_3_S. ^1^H NMR (DMSO-*d_6_*) *δ* 7.20-8.45 (m, 6H, Ar-H, NH_2_, CH=N), 11.75 (s, 1H, NH). 

**2-(2-Chloro-5-nitrobenzylidene)hydrazinocarbothioamide (4h): **Yield 79%, m.p. 207-208 °C (Hao, 2010[25]), (ethanol /water (2:1)), C_8_H_7_ClN_4_O_2_S. ^1^H NMR (DMSO-*d_6_*) *δ* 7.75-9.00 (m, 6H, Ar-H, CH=N, NH_2_), 11.80 (s, 1H, NH). ^13^C NMR (DMSO-*d_6_*) *δ* 125.1, 125.8, 129.6, 133.9, 141.2, 145.1, 146.9, 179.1.

**2-(3-Bromo-4-hydroxybenzylidene)hydrazinocarbothioamide (4i): **Yield 67%, m.p. 169-172 °C (Tsurkan et al., 1982[72]), (methanol), C_8_H_8_BrN_3_OS. ^1^H NMR (DMSO-*d_6_*) *δ *6.95-8.20 (m, 6H, Ar-H, NH_2_, CH=N), 10.75 (s, 1H, OH), 11.30 (s, 1H, NH).

**2-(2-Hydroxy-5-methylbenzylidene)hydrazinocarbothioamide (4j):** Yield 72%, m.p. 196-198 °C (Pahontu et al., 2013[49]), (ethyl acetate/ethanol (3:1)), C_9_H_11_N_3_OS. ^1^H NMR (DMSO-*d_6_*) *δ* 2.20 (s, 3H, CH_3_), 6.70-8.30 (m, 6H, Ar-H, NH_2_, CH=N), 9.50 (s, 1H, OH), 11.30 (s, 1H, NH). 

**2-(4-Hydroxy-3-methylbenzylidene)hydrazinocarbothioamide (4k): **Yield 71%, m.p. 173-175 °C, (ethyl acetate/ethanol (3:1)) (Kaishi, 1953[32]), C_9_H_11_N_3_OS. ^1^H NMR (DMSO-*d_6_*) *δ* 2.15 (s, 3H, CH_3_), 6.85-8.05 (m, 6H, Ar-H, NH_2_, CH=N), 9.85 (s, 1H, OH), 11.20 (s, 1H, NH). 

**2-(2,4-Dimethoxybenzylidene)hydrazinocarbothioamide (4l):** Yield 69%, m.p. 221-223 °C (Pasha et al., 2008[51]), (ethanol/water (2:1)), C_10_H_13_N_3_O_2_S. ^1^H NMR (DMSO-*d_6_*) *δ* 3.82 (s, 6H, 2OCH_3_), 6.60 (s, 2H, NH_2_), 7.85-8.10 (m, 3H, Ar-H), 8.35 (s, 1H, CH=N), 11.30 (s, 1H, NH). 

**2-((1*H*-Pyrrol-2-yl)methylidene)hydrazinocarbothioamide (4m):** Yield 84%, m.p. 200-201 °C (Yi et al., 2011[80]), (methanol), C_6_H_8_N_4_S. ^1^H NMR (DMSO-*d_6_*) *δ* 6.15-7.95 (m, 5H, pyrrole-H, NH_2_), 7.90 (s, 1H, CH=N), 11.20 (s, 1H, NH), 11.35 (s, 1H, NH).

**2-((1,1'-Biphenyl)-4-ylmethylidene)hydrazinocarbothioamide (4n):** Yield 80%, m.p. 202-203 °C (Mendoza-Merono et al., 2010[43]), (ethanol/water (2:1)), C_14_H_13_N_3_S. ^1^H NMR (DMSO-*d_6_*) *δ* 7.45-8.25 (m, 12H, Ar-H, NH_2_, CH=N), 11.50 (s, 1H, NH). ^13^C NMR (DMSO-*d_6_*) *δ* 127.1, 127.3, 128.2, 128.3, 129.4, 133.7, 139.8, 141.7, 142.3, 178.4.

**2-(Naphthalen-2-ylmethylidene)hydrazinocarbothioamide (4o):** Yield 88%, m.p. 245-246 °C (Yi et al., 2011[80]), (ethanol/water (2:1)), C_12_H_11_N_3_S. ^1^H NMR (DMSO-*d_6_*) *δ* 7.10-8.70 (m, 10H, Ar-H, NH_2_, CH=N), 11.55 (s, 1H, NH).

**2-((1-Nitronaphthalen-2-yl)methylidene)hydrazinocarbothioamide (4p):** Yield 77%, m.p. 183-185 °C, (ethyl acetate/ethanol (3:1)). ^1^H NMR (DMSO-*d_6_*) *δ* 7.60-8.45 (m, 9H, Ar-H, NH_2_, CH=N), 11.70 (s, 1H, NH). Anal. C_12_H_10_N_4_O_2_S (C, H, N). 

**2-((2-Oxo-2*H*-chromen-6-yl)methylidene)hydrazinocarbothioamide (4q): **Yield 71%, m.p. 276-278 °C (Datta and Daniels, 1963[11]), (methanol), C_11_H_9_N_3_O_2_S. ^1^H NMR (DMSO-*d_6_*) *δ* 6.55-8.20 (m, 8H, Ar-H, C4-H of chromone, NH_2_, CH=N), 11.55 (s, 1H, NH).

**2-((10-Chloroanthracen-9-yl)methylidene)hydrazinocarbothioamide (4r):** Yield 81%, m.p. 195-197 °C, (ethyl acetate/ethanol (3:1)). ^1^H NMR (DMSO-*d_6_*) *δ* 7.70-8.65 (m, 10H, Ar-H), 9.20 (s, 1H, CH=N), 11.85 (s, 1H, NH). Anal. C_16_H_12_ClN_3_S (C, H, N). 

**2-(Phenanthren-9-ylmethylidene)hydrazinocarbothioamide (4s):** Yield 69%, m.p. 219-220 °C (Ebrahimi et al., 2015[15]), (ethanol/water (2:1)), C_16_H_13_N_3_S. ^1^H NMR (DMSO-*d_6_*) *δ* 7.65-8.95 (m, 12H, Ar-H, NH_2_, CH=N), 11.55 (s, 1H, NH). ^13^C NMR (DMSO-*d_6_*) *δ* 123.3, 123.9, 124.4, 127.4, 127.7, 127.9, 128.0, 128.2, 128.4, 128.7, 129.4, 130.5, 130.7, 131.2, 142.0, 178.4.

**2-(Pyren-2-ylmethylidene)hydrazinocarbothioamide (4t):** Yield 83%, m.p. 200-202 °C, (ethyl acetate/ethanol (3:1)). ^1^H NMR (DMSO-*d_6_*) *δ* 8.05-8.90 (m, 11H, Ar-H, NH_2_), 9.25 (s, 1H, CH=N), 11.65 (s, 1H, NH). Anal. C_18_H_13_N_3_S (C, H, N).

**Synthesis of hydrazinothiazolylcoumarin derivatives 5a-t:** 3-(Bromoacetyl)coumarin **(3)** (0.107 g, 4 mmol), 2-arylidenehydrazinocarbothioamides **4a-t** (4 mmol) and glacial acetic acid (0.1 mL) were heated in ethanol (10 mL) under microwave irradiation (60 W) at 100 °C for 10 min. The attained solid was filtered and crystallized to give **5a-t**.

**3-(2-(2-(2-Bromobenzylidene)hydrazino)thiazol-4-yl)-2*H*-chromen-2-one (5a):** Yield 75%, m.p. 212-213 °C, (ethanol/water (2:1)). ^1^H NMR (DMSO-*d_6_*) *δ* 7.30-8.65 (m, 11H, Ar-H, C_5_-H of thiazole, C_4_-H of chromone, CH=N), 12.50 (s, 1H, NH).^ 13^C NMR (DMSO-*d_6_*) *δ* 116.3, 119.6, 120.9, 123.1, 125.2, 127.1, 128.6, 129.3, 129.9, 131.4, 132.2, 133.4, 133.6, 138.7, 140.2, 144.5, 152.8, 159.2, 167.9. HRMS: *m/z* (ESI) calcd for C_19_H_11_BrN_3_O_2_S^-^, [M-H]^-^ : 423.9749; found: 423.9758. Anal. C_19_H_12_BrN_3_O_2_S (C, H, N). 

**3-(2-(2-(2-Cyanobenzylidene)hydrazino)thiazol-4-yl)-2*H*-chromen-2-one (5b): **Yield 69%, m.p. 219-220 °C, (ethanol/water (2:1)). ^1^H NMR (DMSO-*d_6_*) *δ* 7.30-8.80 (m, 11H, Ar-H, C_5_-H of thiazole, C_4_-H of chromone, CH=N), 12.55 (s, 1H, NH). ^13^C NMR (DMSO-*d_6_*) *δ* 112.3, 113.6, 116.1, 120.7, 123.1, 125.2, 127.3, 128.9, 129.7, 129.9, 131.7, 132.1, 132.9, 133.5, 138.2, 143.7, 145.8, 152.6, 161.3, 168.4. HRMS: *m/z* (ESI) calcd for C_20_H_11_N_4_O_2_S^-^, [M-H]^-^ : 371.0581; found: 371.0593. Anal. C_20_H_12_N_4_O_2_S (C, H, N). 

**3-(2-(2-(3-Cyanobenzylidene)hydrazino)thiazol-4-yl)-2*H*-chromen-2-one (5c): **Yield 63%, m.p. 224-225 °C, (methanol). ^1^H NMR (DMSO-*d_6_*) *δ* 7.30-8.10 (m, 10H, Ar-H), 8.55 (s, 1H, CH=N), 12.40 (s, 1H, NH). HRMS: *m/z* (ESI) calcd for C_20_H_13_N_4_O_2_S^+^, [M+H]^+^: 373.0785; found: 373.0766. Anal. C_20_H_12_N_4_O_2_S (C, H, N).

**3-(2-(2-(4-(Trifluoromethyl)benzylidene)hydrazino)thiazol-4-yl)-2*H*-chromen-2-one (5d): **Yield 72%, m.p. 189-190 °C, (ethanol/water (2:1)). ^1^H NMR (DMSO-*d_6_*) *δ* 7.30-8.15 (m, 9H, Ar-H, C_5_-H of thiazole), 8.30 (s, 1H, C_4_-H of chromone), 8.60 (s, 1H, CH=N), 12.45 (s, 1H, NH). ^13^C NMR (DMSO-*d_6_*) *δ* 111.5, 116.3, 119.6, 120.9, 125.2, 126.1, 126.2, 127.4, 129.2, 129.3, 132.2, 138.7, 140.3, 144.5, 152.7, 159.2, 159.3, 167.9. HRMS: *m/z* (ESI) calcd for C_20_H_11_F_3_N_3_O_2_S^-^, [M-H]^-^ : 414.0535; found: 414.0541. Anal. C_20_H_12_F_3_N_3_O_2_S (C, H, N). 

**3-(2-(2-(3-Methylbenzylidene)hydrazino)thiazol-4-yl)-2*H*-chromen-2-one (5e): **Yield 82%, m.p. 190-191 °C, (ethyl acetate/ethanol (3:1)). ^1^H NMR (DMSO-*d_6_*) *δ* 2.30 (s, 3H, CH_3_), 7.15-8.05 (m, 9H, Ar-H, C_5_-H of thiazole), 8.30 (s, 1H, C_4_-H of chromone), 8.52 (s, 1H, CH=N), 12.20 (s, 1H, NH). ^13^C NMR (DMSO-*d_6_*) *δ* 21.4, 111.1, 116.3, 119.6, 120.9, 121.6, 124.1, 125.2, 127.1, 129.2, 129.3, 130.5, 132.1, 138.5, 138.6, 142.3, 144.4, 152.7, 159.2, 168.1. HRMS: *m/z* (ESI) calcd for C_20_H_14_N_3_O_2_S^-^, [M-H]^-^ : 360.0800; found: 360.0813. Anal. C_20_H_15_N_3_O_2_S (C, H, N).

**3-(2-(2-(2,6-Dichlorobenzylidene)hydrazino)thiazol-4-yl)-2*H*-chromen-2-one (5f): **Yield 81%, m.p. 230-232 °C, (chloroform). ^1^H NMR (DMSO-*d_6_*) *δ* 7.30-7.90 (m, 8H, Ar-H, C_5_-H of thiazole), 8.30 (s, 1H, C_4_-H of chromone), 8.60 (s, 1H, CH=N), 12.50 (s, 1H, NH). ^13^C NMR (DMSO-*d_6_*) *δ* 111.7, 116.3, 119.6, 120.9, 125.1, 125.3, 129.3, 130.9, 132.1, 134.0, 136.8, 139.7, 144.4, 152.8, 159.2, 159.3, 167.9. HRMS: *m/z* (ESI) calcd for C_19_H_12_Cl_2_N_3_O_2_S^+^, [M+H]^+^: 416.0034; found: 416.0030. Anal. C_19_H_11_Cl_2_N_3_O_2_S (C, H, N).

**3-(2-(2-(2-Chloro-6-fluorobenzylidene)hydrazino)thiazol-4-yl)-2*H*-chromen-2-one (5g): **Yield 69%, m.p. 195-196 °C, (ethanol/water (2:1)). ^1^H NMR (DMSO-*d_6_*) *δ* 7.25-7.90 (m, 8H, Ar-H, C_5_-H of thiazole), 8.25 (s, 1H, C_4_-H of chromone), 8.65 (s, 1H, CH=N), 12.50 (s, 1H, NH). ^13^C NMR (DMSO-*d_6_*) *δ* 111.7, 115.9, 116.1, 119.6, 120.8, 120.9, 125.2, 126.7, 126.8, 129.3, 133.4, 134.7, 138.7, 144.4, 152.8, 159.2, 159.3, 161.9, 167.9. HRMS: *m/z* (ESI) calcd for C_19_H_12_ClFN_3_O_2_S^+^, [M+H]^+^: 400.0328; found: 400.0314. Anal. C_19_H_11_ClFN_3_O_2_S (C, H, N). 

**3-(2-(2-(2-Chloro-5-nitrobenzylidene)hydrazino)thiazol-4-yl)-2*H*-chromen-2-one** **(5h): **Yield 74%, m.p. 176-177 °C, (ethanol/water (2:1)). ^1^H NMR (DMSO-*d_6_*) *δ* 7.30-8.65 (m, 10H, Ar-H, C_5_-H of thiazole, C_4_-H of chromone, CH=N), 12.70 (s, 1H, NH). ^13^C NMR (DMSO-*d_6_*) *δ* 112.3, 120.9, 123.1, 125.4, 125.7, 126.9, 127.2, 128.4, 129.3, 129.9, 133.7, 138.1, 141.0, 142.8, 145.9, 146.2, 151.3, 161.6, 169.2. HRMS: *m/z* (ESI) calcd for C_19_H_12_ClN_4_O_4_S^+^, [M+H]^+^: 427.0246; found: 427.0267. Anal. C_19_H_11_ClN_4_O_4_S (C, H, N).

**3-(2-(2-(3-Bromo-4-hydroxybenzylidene)hydrazino)thiazol-4-yl)-2*H*-chromen-2-one (5i): **Yield 80 %, m.p. 182-184 °C, (ethyl acetate/ethanol (3:1)). ^1^H NMR (DMSO-*d_6_*) *δ* 7.00-8.85 (m, 10H, Ar-H, C_5_-H of thiazole, C_4_-H of chromone, CH=N), 10.85 (s, 1H, OH), 12.05 (s, 1H, NH). HRMS: *m/z* (ESI) calcd for C_19_H_11_BrN_3_O_3_S^-^, [M-H]^-^ : 439.9685; found: 439.9698. Anal. C_19_H_12_BrN_3_O_3_S (C, H, N). 

**3-(2-(2-(2-Hydroxy-5-methylbenzylidene)hydrazino)thiazol-4-yl)-2*H*-chromen-2-one** **(5j): **Yield 84%, m.p. 206-207 °C, (ethyl acetate/ethanol (3:1)).^ 1^H NMR (DMSO-*d_6_*) *δ* 2.20 (s, 3H, CH_3_), 6.80-7.90 (m, 8H, Ar-H, C_5_-H of thiazole), 8.30 (s, 1H, C_4_-H of chromone), 8.55 (s, 1H, CH=N), 9.80 (s, 1H, OH), 12.15 (s, 1H, NH). ^13^C NMR (DMSO-*d_6_*) *δ* 20.6, 110.8, 116.3, 116.5, 119.6, 120.1, 120.9, 125.2, 126.7, 128.4, 129.3, 131.8, 132.1, 138.6, 140.6, 144.6, 152.8, 154.4, 159.2, 167.8. HRMS: *m/z* (ESI) calcd for C_20_H_14_N_3_O_3_S^-^, [M-H]^- ^: 376.0781; found: 376.0771. Anal. C_20_H_15_N_3_O_3_S (C, H, N).

**3-(2-(2-(4-Hydroxy-3-methylbenzylidene)-hydrazino)thiazol-4-yl)-2*H*-chromen-2-one** **(5k): **Yield 71%, m.p. 193-194 °C, (ethanol/water (2:1)). ^1^H NMR (DMSO-*d_6_*) *δ* 2.15 (s, 3H, CH_3_), 6.80 (s, 1H, C_5_-H of thiazole), 7.25-7.95 (m, 8H, Ar-H, C_4_-H of chromone), 8.50 (s, 1H, CH=N), 9.75 (s, 1H, OH), 11.90 (s, 1H, NH). HRMS: *m/z* (ESI) calcd for C_20_H_14_N_3_O_3_S^-^, [M-H]^-^ : 376.0781; found: 376.0765. Anal. C_20_H_15_N_3_O_3_S (C, H, N).

**3-(2-(2-(2,4-Dimethoxybenzylidene)-hydrazino)thiazol-4-yl)-2*H*-chromen-2-one (5l): **Yield 79%, m.p. 219-220 °C, (chloroform). ^1^H NMR (DMSO-*d_6_*) *δ* 3.80 (s, 3H, OCH_3_), 3.86 (s, 3H, OCH_3_), 6.65-8.55 (m, 10H, Ar-H, C_5_-H of thiazole, C_4_-H of chromone, CH=N), 11.90 (s, 1H, NH). ^13^C NMR (DMSO-*d_6_*) *δ* 56.1, 56.2, 102.3, 106.3, 111.3, 115.6, 120.6, 121.8, 125.9, 126.6, 128.2, 129.6, 132.3, 136.7, 144.1, 146.4, 151.3, 159.6, 161.3, 163.5, 169.1. HRMS: *m/z* (ESI) calcd for C_21_H_18_N_3_O_4_S^+^, [M+H]^+^: 408.1037; found: 408.1025. Anal. C_21_H_17_N_3_O_4_S (C, H, N).

**3-(2-(2-((1*H*-Pyrrol-2-yl)methylidene)hydrazino)thiazol-4-yl)-2*H*-chromen-2-one (5m): **Yield 62%, m.p. 167-168 °C, (chloroform). ^1^H NMR (DMSO-*d_6_*) *δ* 6.10-6.90 (m, 4H, pyrrole-H, C_5_-H of thiazole), 7.30-7.95 (m, 5H, Ar-H, C_4_-H of chromone), 8.55 (s, 1H, CH=N), 11.30 (s, 1H, NH), 11.85 (s, 1H, NH). ^13^C NMR (DMSO-*d_6_*) *δ* 111.3, 113.7, 116.6, 120.3, 120.9, 125.1, 125.6, 128.1, 128.6, 129.7, 133.1, 139.4, 145.2, 146.3, 155.4, 162.1, 170.9. HRMS: *m/z* (ESI) calcd for C_17_H_11_N_4_O_2_S^-^, [M-H]^-^ : 335.0610; found: 335.0619. Anal. C_17_H_12_N_4_O_2_S (C, H, N). 

**3-(2-(2-((1,1'-Biphenyl)-4-ylmethylidene)-hydrazino)thiazol-4-yl)-2*H*-chromen-2-one (5n): **Yield 77%, m.p. 198-200 °C, (ethyl acetate/ethanol (3:1)). ^1^H NMR (DMSO-*d_6_*) *δ* 7.30-8.65 (m, 16H, Ar-H, C_5_-H of thiazole, C_4_-H of chromone, CH=N), 12.25 (s, 1H, NH).^ 13^C NMR (DMSO-*d_6_*) *δ* 111.2, 116.4, 119.6, 121.0, 125.2, 127.1, 127.4, 127.5, 128.3, 129.3, 129.5, 132.2, 133.8, 138.7, 139.8, 141.3, 141.8, 144.5, 152.8, 159.3, 168.2. HRMS: *m/z* (ESI) calcd for C_25_H_16_N_3_O_2_S^-^, [M-H]^-^ : 422.0955; found: 422.0959. Anal. C_25_H_17_N_3_O_2_S (C, H, N).

**3-(2-(2-(Naphthalen-2-ylmethylidene)hydrazino)thiazol-4-yl)-2*H*-chromen-2-one (5o): **Yield 79%, m.p. 225-227 °C, (ethyl acetate/ethanol (3:1)). ^1^H NMR (DMSO-*d_6_*) *δ* 7.30-8.60 (m, 14H, Ar-H, C_5_-H of thiazole, C_4_-H of chromone, CH=N), 12.30 (s, 1H, NH). HRMS: *m/z* (ESI) calcd for C_23_H_14_N_3_O_2_S^-^, [M-H]^-^ : 396.0812; found: 396.0795. Anal. C_23_H_15_N_3_O_2_S (C, H, N). 

**3-(2-(2-((1-Nitronaphthalen-2-yl)methylidene)hydrazino)thiazol-4-yl)-2*H*-chromen-2-one (5p): **Yield 63%. m.p. 211-213 °C, (chloroform). ^1^H NMR (DMSO-*d_6_*) *δ* 7.35-8.25 (m, 12H, Ar-H, C_5_-H of thiazole, C_4_-H of chromone), 8.55 (s, 1H, CH=N), 12.75 (s, 1H, NH). ^13^C NMR (DMSO-*d_6_*) *δ* 113.2, 115.9, 121.1, 123.6, 124.2, 125.1, 125.7, 126.3, 127.3, 127.6, 128.4, 128.9, 129.5, 129.8, 135.2, 137.4, 142.9, 144.6, 145.9, 147.2, 154.3, 162.2, 171.7. HRMS: *m/z* (ESI) calcd for C_23_H_13_N_4_O_4_S^-^, [M-H]^-^ : 441.0663; found: 441.0660. Anal. C_23_H_14_N_4_O_4_S (C, H, N). 

**6-((2-(4-(2-Oxo-2*H*-chromen-3-yl)thiazol-2-yl)hydrazono)methyl)-2*H*-chromen-2-one (5q): **Yield 76%, m.p. 181-183 °C, (ethanol/water (2:1)). ^1^H NMR (DMSO-*d_6_*) *δ* 6.50 (d, 1H, C_3_-H of chromone), 7.30-8.15 (m, 10H, Ar-H, C_5_-H of thiazole, C_4_-H of two chromone moieties), 8.55 (s, 1H, CH=N), 12.25 (s, 1H, NH). ^13^C NMR (DMSO-*d_6_*) *δ* 111.2, 116.3, 117.2, 117.5, 119.5, 119.6, 120.9, 125.2, 126.7, 129.2, 129.7, 131.2, 132.2, 138.6, 140.6, 144.5, 151.9, 152.7, 154.3, 159.2, 160.1, 168.0. HRMS: *m/z* (ESI) calcd for C_22_H_12_N_3_O_4_S^-^, [M-H]^-^ : 414.0560; found: 414.0565. Anal. C_22_H_13_N_3_O_4_S (C, H, N). 

**3-(2-(2-((10-Chloroanthracen-9-yl)-methylidene)hydrazino)thiazol-4-yl)-2*H*-chromen-2-one (5r): **Yield 86%, m.p. 238-240 °C, (ethanol/water (2:1)). ^1^H NMR (DMSO-*d_6_*) *δ* 7.20-8.75 (m, 15H, Ar-H, C_5_-H of thiazole, C_4_-H of chromone, CH=N), 12.50 (s, 1H, NH). ^13^C NMR (DMSO-*d_6_*) *δ* 111.3, 116.4, 120.4, 122.5, 125.1, 125.2, 125.9, 126.7, 127.8, 128.4, 128.8, 129.3, 130.1, 132.5, 134.0, 138.8, 140.1, 142.3, 152.0, 164.2, 168.0. HRMS: *m/z* (ESI) calcd for C_27_H_15_ClN_3_O_2_S^-^, [M-H]^-^ : 480.0574; found: 480.0574. Anal. C_27_H_16_ClN_3_O_2_S (C, H, N). 

**3-(2-(2-(Phenanthren-9-ylmethylidene)-hydrazino)thiazol-4-yl)-2*H*-chromen-2-one (5s): **Yield 87%, m.p. 245-247 °C, (chloroform). ^1^H NMR (DMSO-*d_6_*) *δ* 7.30-8.20 (m, 11H, Ar-H, C_5_-H of thiazole, C_4_-H of chromone, CH=N), 8.55-9.10 (m, 5H, Ar-H), 12.45 (s, 1H, NH). HRMS: *m/z* (ESI) calcd for C_27_H_16_N_3_O_2_S^-^, [M-H]^-^ : 446.0941; found: 446.0960. Anal. C_27_H_17_N_3_O_2_S (C, H, N). 

**3-(2-(2-(Pyren-2-ylmethylidene)hydrazino) thiazol-4-yl)-2*H*-chromen-2-one (5t): **Yield 89%, m.p. 237-239 °C, (ethanol/water (2:1)). ^1^H NMR (DMSO-*d_6_*) *δ* 7.35-9.00 (m, 16H, Ar-H, C_5_-H of thiazole, C_4_-H of chromone, CH=N), 12.50 (s, 1H, NH). HRMS: *m/z* (ESI) calcd for C_29_H_16_N_3_O_2_S^-^, [M-H]^-^ : 470.0975; found: 470.0979. Anal. C_29_H_17_N_3_O_2_S (C, H, N). 

### Biology 

Detailed biological screening methods are provided in the supplementary information. 

#### In vitro antitumor assay

The new analogs were tested for *in vitro* antitumor efficacy adopting the reported procedure (Mosmann, 1983[46]; Denizot and Lang, 1986[13]; Gerlier and Thomasset, 1986[21]). 

#### In vivo antitumor assay

*In vivo* antitumor assessment of **5f, 5h, 5m **and** 5r **was performed according to the literature method (Oberling and Guerin, 1954[48]; Sheeja et al., 1997[64]; Clarkson and Burchenal, 1965[9]).

#### In vitro cytotoxicity testing

*In vitro *cytotoxic activity of **5f, 5h, 5m **and** 5r **was evaluated in accord to the reported method (Mosmann, 1983[46]; Denizot and Lang, 1986[13]; Gerlier and Thomasset, 1986[21]).

## Results and Discussion

### Chemistry

**Figure 2:**
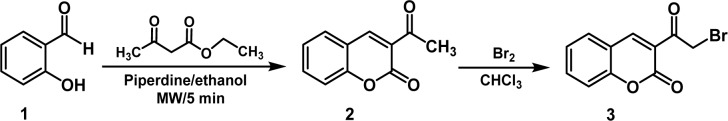
Synthesis of 3-(bromoacetyl)coumarin (3)

**Figure 3:**
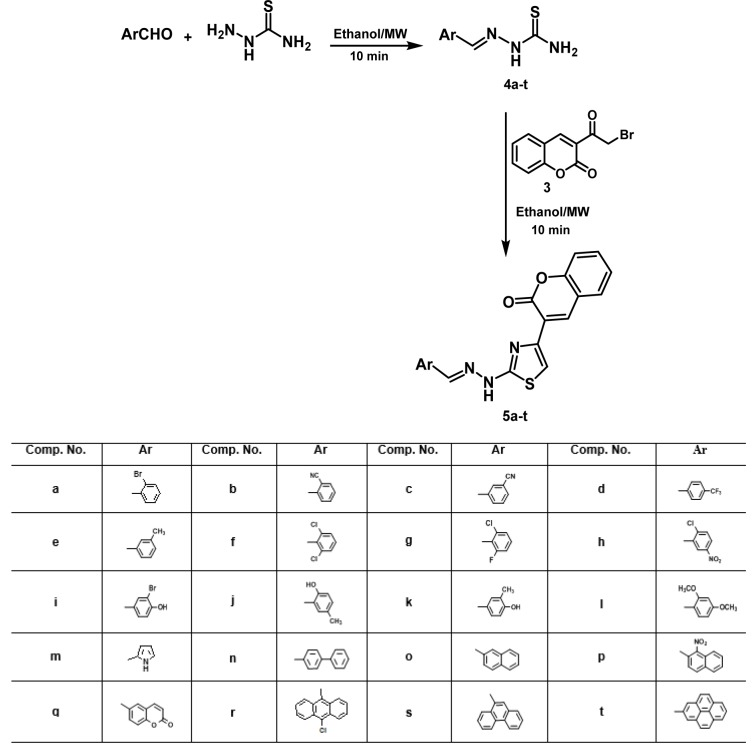
Synthesis of thiazolylcoumarin hybrids 5a-t

3-(Bromoacetyl)coumarin **(3)** was synthesized *via* a two step procedure (Figure 2(Fig. 2)). First, cyclocondensation of salicylaldehyde **(1)** and ethyl acetoacetate under microwave irradiation utilizing piperidine as a catalyst to give the 3-acetylcoumarin **(2)** (Valizadeh et al., 2007[76]). Second, bromination of compound **2** in chloroform to yield the bromoketone **3** in 63% yield (Siddiqui et al., 2009[66]) (Figure 2(Fig. 2)). The 2-arylidenehydrazinocarbothioamides **4a-t **were synthesized through condensation of the aromatic aldehydes and thiosemicarbazide in ethanol under microwave irradiation (Figure 3(Fig. 3)). Microwave irradiation of **4a-t** and bromoketone **3 **in ethanol, followed by addition of ammonium hydroxide 5%, furnished the desired thiazolylcoumarin hybrids **5a-t** in moderate to good yields (62-89%) (Figure 3(Fig. 3)).

### Biological screening 

#### In vitro antitumor screening

*In vitro* antitumor screening of compounds **5a-t** was carried out on cervical (Hela) and kidney fibroblast (COS-7) cancer cell lines in accord to MTT assay (Mosmann, 1983[46]; Denizot and Lang, 1986[13]; Gerlier and Thomasset, 1986[21]) and utilizing doxorubicin as a standard drug. The concentrations of the compounds that cause 50% inhibition of cell viability (IC_50_, µM) were calculated. Compounds **5f, 5h, 5m** and **5r** exhibited remarkable activity against Hela cell line. In addition, **5h** and **5r** displayed outstanding efficacy toward COS-7 cell line (Table 1(Tab. 1)). The rest of the tested compounds displayed weaker efficacy. 

#### Structure-activity relationship 

Compound **5f** incorporating 2,6-dichlorophenyl moiety displayed prominent antitumor efficacy toward Hela cell line and it represents the basic framework for further structural modifications. Replacing this moiety with 2-chloro-6-fluorophenyl counterpart abolished the activity against the same cell line (compound **5g**), whereas its replacement with 2-chloro-5-nitrophenyl counterpart led to increased efficacy toward the same cell line and a tremendous improvement in the activity toward COS-7 cell line (compound **5h**). Incorporation of pyrrol-2-yl moiety into the thiazolylcoumarin resulted in considerable efficiency toward Hela and COS-7 cell lines which might be attributed to additional interaction with the target receptor (compound **5m**). 10-Chloroanthracen-9-yl moiety was proved to exhibit the optimum hydrophobic binding affinity and displayed the most potent antitumor efficacy against both cell lines (compound **5r**).

#### In vivo antitumor screening

Results of *in vivo *antitumor screening of compounds **5f**, **5h**, **5m **and **5r** (showing the highest *in vitro* antitumor activity) against EAC cells in mice are listed in Tables 2-4(Tab. 2)(Tab. 3)(Tab. 4). The % increase in lifespan of EAC inoculated mice (%ILS), the decrease in viable tumor cell count and the retrieval of normal blood profile are three substantial measures used for estimation of antitumor efficacy of the selected compounds and 5-fluorouracil (5-FU) (standard agent) (Oberling and Guerin, 1954[48]; Sheeja et al., 1997[64]; Clarkson and Burchenal, 1965[9]). The mean survival time (MST) of each group was rated and %ILS of mice inoculated with EAC cells was determined adopting the equation: %ILS = [(MST of treated group/ MST of positive control group)-1] x 100, where MST = days of the mouse in a group/total no. of mice. Compound **5r **displayed prominent increase in lifespan of mice (Table 2(Tab. 2)). Also, this compound produced considerable decrease in viable tumor cell count (Table 3(Tab. 3)). Regarding the effect on blood profile, compound **5r** showed higher Hb and RBC levels and lower WBC count than 5-FU (Table 4(Tab. 4)). 

#### In vitro cytotoxicity testing

The effective antitumor compounds,** 5f**, **5h**, **5m** and **5r** were further assessed for *in vitro* cytotoxicity toward human normal lung fibroblast (W138) cell line (Mosmann, 1983[46]; Denizot and Lang, 1986[13]; Gerlier and Thomasset, 1986[21]). IC_50_ values (µM) of the tested compounds and 5-FU (reference cytotoxic agent) were calculated. Results (Table 5(Tab. 5)) revealed that the four tested compounds are less cytotoxic than 5-FU. Comparing the IC_50_ values of **5h**, **5m** and **5r** on the tested normal cell line (19.75-29.47 µM) with those on the tested cancer cell lines (1.29-12.50 µM), we can conclude that the three compounds are more selective cytotoxic agents toward cancer cells than normal cells. In addition, **5f** was found to be more selective toward Hela cancer cell line (IC_50 _= 1.90 µM) than W138 normal cell line (IC_50 _= 36.21 µM).

### 3D Pharmacophore elucidation

A pharmacophore is a set of common structural features shared by a group of compounds that interacts with the complementary sites on a specific target leading to biological activity (Rodolpho and Andrade, 2013[57]). Based on this assumption, analysis of the molecular recognitions in the biological target interacting with the lead compound will enable the design of more potent analogs.

**Figure 4:**
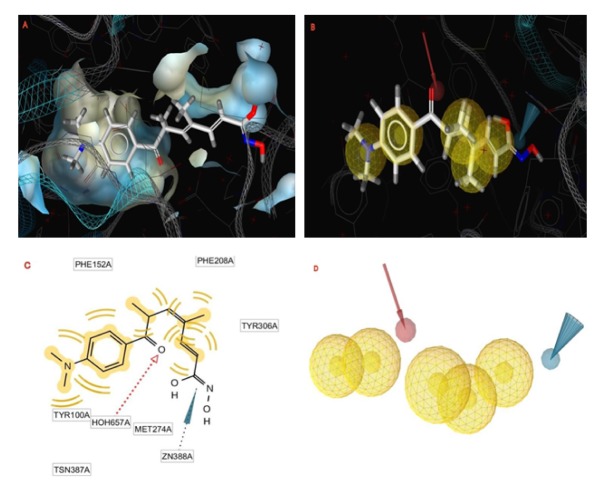
A. LigandScout 3D proposed docking pose for trichostatin A in HDAC8 domain (PDB ID: 1T64). B. 3D Pharmacophore of trichostatin A (in ball and stick presentation); The pharmacophore color coding is red for hydrogen acceptor, yellow for hydrophobic regions, and blue for zinc binding group. C. 2D Representation of the pharmacophoric features of trichostatin A. D. The 3D pharmacophore model for HDAC8 domain (PDB ID: 1T64). The pharmacophore color coding is red for hydrogen acceptor, yellow for hydrophobic regions, and blue for zinc binding group

LigandScout software allows accurate virtual screening based on 3D pharmacophore models, and it is utilized to produce a pharmacophore for trichostatin A (Wolber and Langer, 2005[79]). The model (Figure 4(Fig. 4)) was generated by overlaying the pharmacophoric features of HDAC8 domain complexed with trichostatin A (PDB ID: 1T64) (PDB; http://www.rcsb.org/pdb/home/home.do).

**Figure 5:**
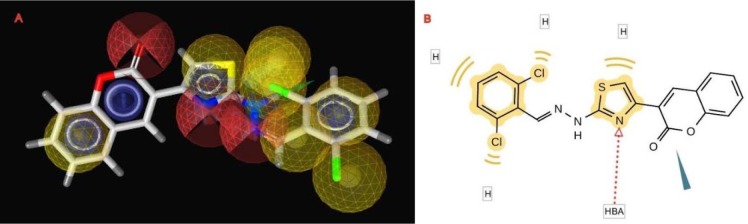
The 3D and 2D alignments of 5f with HDAC8 pharmacophore model. A. 3D Alignment of 5f with HDAC8 pharmacophore model. The pharmacophore color coding is red for hydrogen acceptors, yellow for hydrophobic regions and blue for zinc binding groups. B. 2D Representation of structural features of 5f that can be aligned with the pharmacophore hypothesis. HBA; hydrogen bond acceptor and H; hydrophobic center

**Figure 6:**
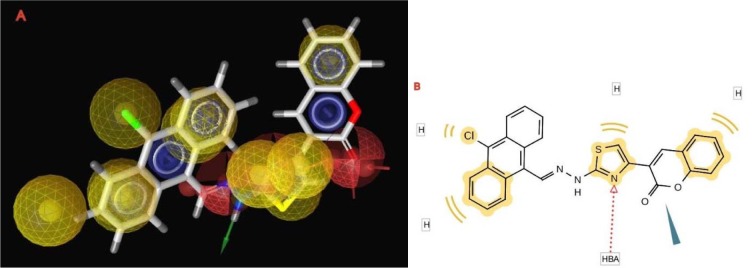
The 3D and 2D alignments of 5r with HDAC8 pharmacophore model. A. 3D Alignment of 5r with HDAC8 pharmacophore model. The pharmacophore color coding is red for hydrogen acceptors, yellow for hydrophobic regions and blue for zinc binding groups. B. 2D Representation of structural features of 5r that can be aligned with the pharmacophore hypothesis. HBA; hydrogen bond acceptor and H; hydrophobic center

**Figure 7:**
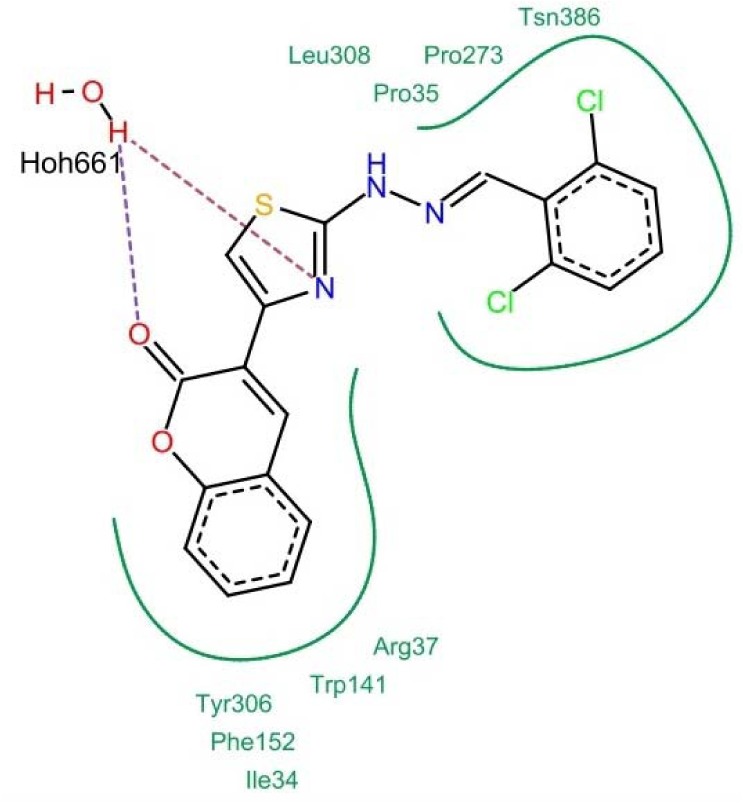
2D Interaction of 5f with HDAC8 domain. Hydrogen bonds are shown by dashed lines. Green solid lines represent hydrophobic interactions

**Figure 8:**
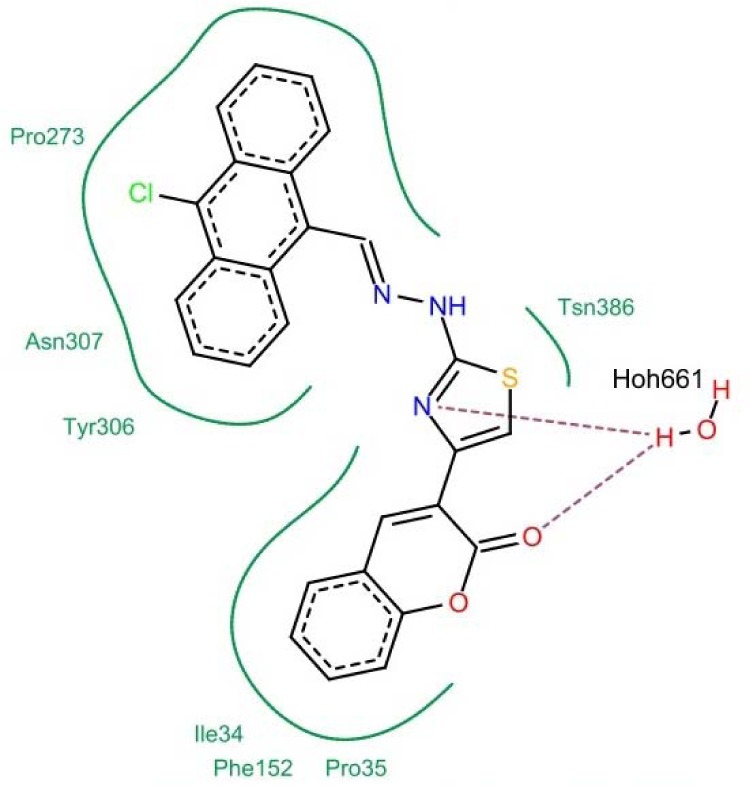
2D Interaction of 5r with HDAC8 domain. Hydrogen bonds are shown by dashed lines. Green solid lines represent hydrophobic interactions

The pharmacophore created by LigandScout revealed the presence of one hydrogen bond acceptor site (red arrow) embedded between five hydrophobic regions represented by yellow spheres which conveys the tremendous contribution of hydrophobic interactions with the receptor. Moreover, ZBG represented by a blue conical shape, was oriented at the terminal of the hydrophobic regions and is proposed to be an essential feature in the presented pharmacophore. The four active antitumor compounds in this study, **5f**, **5h**, **5m** and **5r** were subjected to a pharmacophore-based virtual screening against the target pharmacophore of trichostatin A. The matching pharmacophoric features between the active compounds and trichostatin A are identified in Table 6(Tab. 6). All the active compounds attained a ZBG, a hydrogen bond acceptor site and at least two sites for hydrophobic interactions matching the orientation exhibited by the target pharmacophore. In addition, a relative pharmacophore score illustrated in Table 6(Tab. 6) was calculated for each compound. Compounds **5f** and **5r** exhibited the highest relative pharmacophore score of 0.76 and 0.81, respectively. Figures 5A(Fig. 5) and 6A(Fig. 6) illustrate the 3D alignments of **5f** and **5r**, respectively with the pharmacophore model. 2D Mappings of the pharmacophore model with **5f** and **5r** are shown in Figures 5B(Fig. 5) and 6B(Fig. 6), respectively. The proposed pharmacophore of HDAC8 revealed that hydrophobic forces represent the major contributing interaction with the compounds, accordingly, LeadIT program was utilized to examine the hydrophobic interaction of the active analogs with the target receptor (Stahl and Rarey, 2001[69]). The lipophilic area of each compound exposed toward HDAC8 domain was given a score (Table 6(Tab. 6)). Compounds **5f** and **5r **attained the highest lipophilic area score of -14.12 and -14.65, respectively. 2D Interactions of **5f** and **5r** with HDAC8 domain are presented in Figures 7(Fig. 7) and 8(Fig. 8), respectively.

### In silico studies

Computational chemists follow different approaches for estimation of molecular diversity. Drug-likeness is a qualitative notion used to study how a particular substance is "druglike". So, computer softwares were utilized for predicting the drug-likeness of the new drugs (Ursu et al., 2011[74]). The most active compounds, **5f**, **5h**, **5m** and **5r** were studied for the expectation of Lipinski's rule (Lipinski et al., 2001[38]) along with other molecular properties. 

#### Molinspiration calculations

Lipinski's rule is related to drug absorption (Lipinski et al., 2001[38]). Also, topological polar surface area (TPSA) and number of rotatable bonds (Nrotb) influence oral absorption of drugs (Veber et al., 2002[77]). 

TPSA, Nrotb, and the parameters of Lipinski's rule for the effective analogs, **5f**, **5h**, **5m** and **5r** were evaluated using molinspiration software. 

Results illustrated that all examined analogs have zero or one violation of Lipinski's rule, as well as TPSA values and Nrotb under the acceptable norms; therefore, they are anticipated to be well absorbed (Table 7(Tab. 7)).

#### Drug-likeness

Osiris software (Jarrahpour et al., 2011[31]) was applied for studying the toxicity hazards (mutagenicity, tumorigenicity, irritation & reproductive effects) and drug-likeness of the analyzed compounds. Results revealed that all the analyzed analogs are expected to have no toxicity hazards. It is well established that molecules containing fragments which are extremely available in commercial drugs, have positive drug-likeness values. Results listed in Table 7(Tab. 7) showed that **5f**, **5m** and **5r** have positive drug-likeness values, and they are expected to have fragments which are available in commercial drugs.

For more results see the Supplementary data.

## Conclusion

The recent study led to the development of new efficient antitumor thiazolylcoumarin derivatives. Compounds **5f**,** 5h**,** 5m** and **5r **are the most active antitumor analogs toward Hela cell line; in addition, **5h **and **5r **displayed eminent activity toward COS-7 cell line. Moreover, **5r** displayed the highest *in vivo* activity. Furthermore, the four active analogs were proved to be less cytotoxic than 5-FU on W138 normal cells; therefore, they might be used as potent antitumor agents with low toxicity toward normal cells. Further mechanistic and kinetic investigations concerning the HDACs inhibitory activity of these active compounds will shed light on possible structural modifications desired to obtain new more active antitumor agents.

## Acknowledgments

The authors extend their appreciation to Professor Binghe Wang, Georgia State University, USA, for offering the facilities needed for performing spectral analysis and *in vitro* antitumor testing. Thanks to Mr. Ahmed Abbas, Faculty of Pharmacy, Mansoura University, Egypt, for assessment of *in vivo* antitumor and *in vitro* cytotoxic activities.

## Supplementary Material

Supplementary information

Supplementary data

## Figures and Tables

**Table 1:**
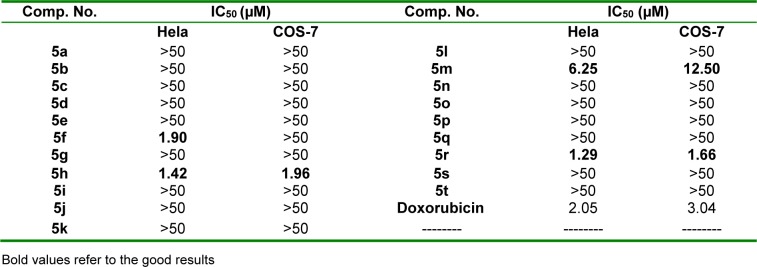
*In vitro* antitumor activity of 5a-t toward Hela and COS-7 cancer cell lines

**Table 2:**
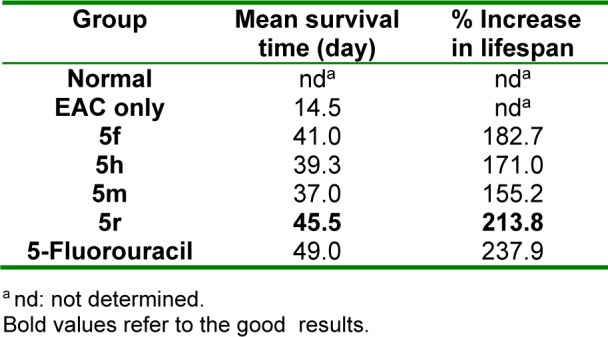
Effect of 5f, 5h, 5m and 5r on mean survival time and % increase in lifespan of mice inoculated with EAC cells

**Table 3:**
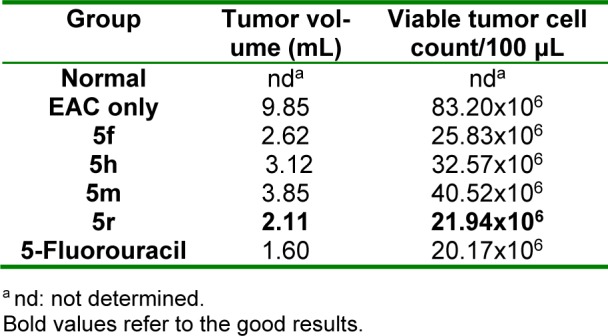
Effect of 5f, 5h, 5m and 5r on tumor volume and viable tumor cell count of mice inoculated with EAC cells

**Table 4:**
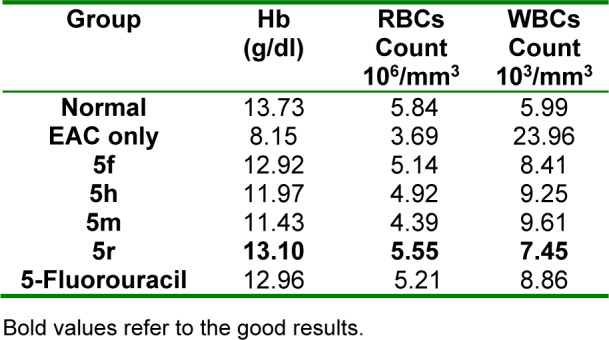
Effect of 5f, 5h, 5m and 5r on blood profile of mice inoculated with EAC cells

**Table 5:**
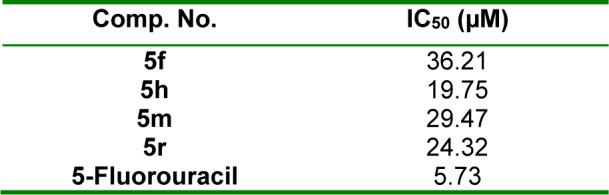
*In vitro* cytotoxic activity of 5f, 5h, 5m and 5r toward W138 normal cell line

**Table 6:**
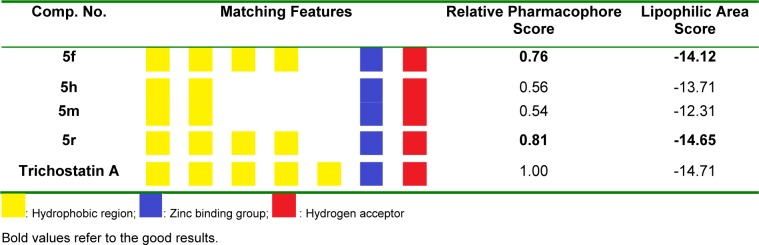
Results of pharmacophore analysis of 5f, 5h, 5m and 5r

**Table 7:**
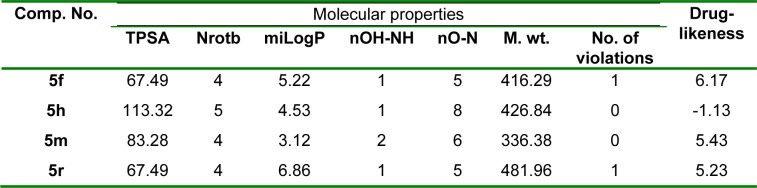
TPSA, Nrotb, calculated Lipinski's rule and drug-likeness of 5f, 5h, 5m and 5r
