# Supplementary material for: Microwave-assisted synthesis and antitumor evaluation of a new series of thiazolylcoumarin derivatives
Source: EXCLI J. 2017 Aug 30;16:1114–31. doi: 10.17179/excli2017-208 (PMC5735336; doi:10.17179/excli2017-208)
Supplement: Supplementary information [file EXCLI-16-1114-s-001.pdf]

EXCLI Journal 2017;16:1114-1131 – ISSN 1611-2156
Received: February 10, 2017, accepted: July 31, 2017, published: August 30, 2017

Supplementary information to:

MICROWAVE-ASSISTED SYNTHESIS AND ANTITUMOR EVALUATION OF A NEW SERIES OF THIAZOLYLCOUMARIN DERIVATIVES

Moustafa T. Gabr[a,b], Nadia S. El-Gohary[a*], Eman R. El-Bendary[a], Mohamed M. El-Kerdawy[a], Nanting Ni[b]

[a] Department of Medicinal Chemistry, Faculty of Pharmacy, Mansoura University, Mansoura 35516, Egypt
[b] Department of Chemistry, Georgia State University, Atlanta, Georgia 30303, USA

[*] Corresponding author: Nadia S. El-Gohary, Department of Medicinal Chemistry, Faculty of Pharmacy, Mansoura University, Mansoura 35516, Egypt; Tel.: +2 010 00326839; Fax: +2 050 2247496; E-mail: dr.nadiaelgohary@yahoo.com

http://dx.doi.org/10.17179/excli2017-208

This is an Open Access article distributed under the terms of the Creative Commons Attribution License (http://creativecommons.org/licenses/by/4.0/).

BIOLOGICAL TESTING

In vitro antitumor screening

Compounds **5a-t** were screened for *in vitro* antitumor activity against HepG2 (liver), HCT-116 (colon) and MCF-7 (breast) cancer cell lines adopting MTT assay (Mosmann, 1983; Denizot and Lang, 1986; Gerlier and Thomasset, 1986) and using 5-fluorouracil as a reference antitumor agent. Cells were cultured in Roswell Park Memorial Institute (RPMI) 1640 medium with 10% fetal bovine serum, penicillin (100 units/mL) and streptomycin (100 µg/mL) at 37 °C in an atmosphere of 5% CO_2 were added.

Cells were placed in 96-multiwell microtiter plates (10^4 cells/well), for 24 hrs at 37 °C and in an atmosphere of 5% CO_2 before treatment with the compounds to allow attachment of the cells to the wall of the plate. The tested compounds were dissolved in DMSO and diluted with phosphate buffer saline (PBS) to obtain different concentrations. Tested compounds of different concentrations were added to each well and cells were incubated with the compounds for 48 hrs at 37 °C and in an atmosphere of 5% CO_2. All tests were performed in triplicates. The treated cells were washed with PBS and 100 µL of 3-(4,5-dimethylthiazol-2-yl)-2,5-diphenyltetrazolium bromide solution (MTT) (5 mg/mL MTT stock in PBS diluted to 1 mg/mL with 10% RPMI-1640 medium) was added. The 96-multiwell plates were read by microarray reader Perkinelmer vector 3V multilabel counter model 1420 (Perkinelmer, Boston, MA) for optical density at 490 nm (Mosmann, 1983; Denizot and Lang, 1986; Gerlier and Thomasset, 1986). The relative percentage cell viability was calculated from the following equation:

$$\%\ \text{cell viability} = \frac{A_{\text{treated cells}} - A_{\text{blank}}}{A_{\text{untreated cells}} - A_{\text{blank}}} \times 100$$

The relation between surviving fraction and drug concentration is plotted to get the survival curve for HepG2, HCT-116 and MCF-7 cancer cell lines. The concentration of each compound

EXCLI Journal 2017;16:1114-1131 – ISSN 1611-2156
Received: February 10, 2017, accepted: July 31, 2017, published: August 30, 2017

required for 50% inhibition of cell viability (IC_{50}, μM) was obtained from the curve fitting using Sigma plot10.

In vivo antitumor screening

Materials

Swiss male albino mice (weighing 20-25 g) were utilized. They were dwelled in microlon boxes at temperature 25 ± 2 °C with a regular 12 hrs light/dark cycle, in addition, food and water intake were permitted.

EAC cells were acquired from the NCI, Cairo, Egypt.

Procedure

Mice were divided into 7 groups (*n*= 6).

Group 1: Negative control (no EAC cells) - received normal saline.
Group 2: Positive control (EAC cells) - received normal saline.
Group 3: EAC cells - received compound **5f** (0.5 mg/mouse).
Group 4: EAC cells - received compound **5h** (0.5 mg/mouse).
Group 5: EAC cells - received compound **5m** (0.5 mg/mouse).
Group 6: EAC cells - received compound **5r** (0.5 mg/mouse).
Group 7: EAC cells - received 5-fluorouracil (0.5 mg/mouse).

2 x 10^6 cells were inoculated intraperitoneally to each mouse in all groups except the negative control group. The treatment began 24 hrs after inoculation (0.5 mg/mouse) and continued for nine days (Oberling and Guerin, 1954; Sheeja et al., 1997; Clarkson and Burchenal, 1965).

Determination of tumor volume

The ascetic fluid was gathered from the peritoneal cavity of dissected mice. It was then centrifuged for 5 min and the tumor cell volume was set.

Determination of viable tumor cell count after five days of treatment

EAC cells (100 μL, from three mice of each group) were obtained and diluted to twenty-fold with saline. The cells were stained with trypan blue, the dead cells are stained, whereas the viable ones did not take up the dye. The number of viable cells was counted.

In vitro cytotoxicity testing

In vitro cytotoxic activity of benzimidazoles **5f, 5h, 5m** and **5r** was evaluated adopting the same procedure described under *in vitro* antitumor testing (Mosmann, 1983; Denizot and Lang, 1986; Gerlier and Thomasset, 1986).

REFERENCES

Clarkson BD, Burchenal JH. Preliminary screening of antineoplastic drugs. Prog Clin Cancer. 1965;1:625-9.

Denizot F, Lang R. Rapid colorimetric assay for cell growth and survival. Modifications to the tetrazolium dye procedure giving improved sensitivity and reliability. J Immunol Methods. 1986;89:271-7.

Gerlier D, Thomasset T. Use of MTT colorimetric assay to measure cell activation. J Immunol Methods. 1986; 94:57-63.

Mosmann T. Rapid colorimetric assay for cellular growth and survival: Application to proliferation and cytotoxicity assays. J Immunol Methods. 1983;65:55-63.

Oberling C, Guerin M. The role of viruses in the production of cancer. Adv Cancer Res. 1954;2:353-423.

Sheeja KR, Kuttan G, Kuttan R. Cytotoxic and antitumour activity of Berberin. Amala Res Bull. 1997;17:73-6.
