# Supplementary material for: Microwave-assisted synthesis and antitumor evaluation of a new series of thiazolylcoumarin derivatives
Source: EXCLI J. 2017 Aug 30;16:1114–31. doi: 10.17179/excli2017-208 (PMC5735336; doi:10.17179/excli2017-208)
Supplement: Supplementary data [file EXCLI-16-1114-s-002.pdf]

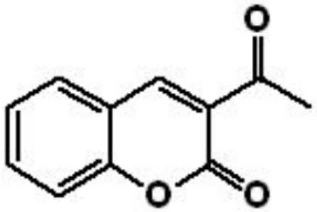

2

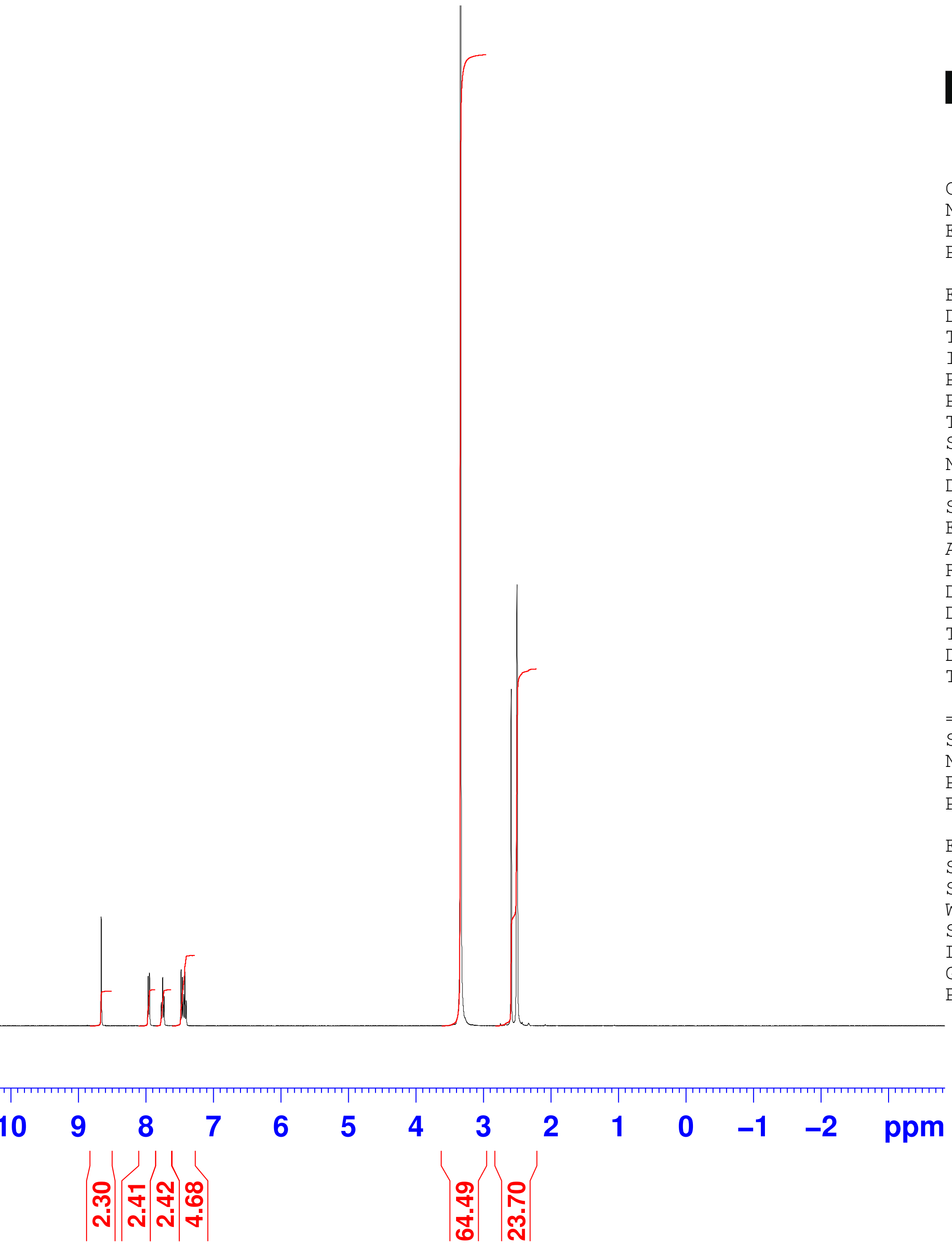


```
Current Data Parameters
NAME                  MG46
EXPNO                    1
PROCNO                   1

F2 - Acquisition Parameters
Date_             20130206
Time                 17.47
INSTRUM              spect
PROBHD    5 mm PABBO BB-
PULPROG               zg30
TD                   65536
SOLVENT               DMSO
NS                      16
DS                       2
SWH               8012.820 Hz
FIDRES            0.122266 Hz
AQ               4.0894465 sec
RG                     181
DW                  62.400 usec
DE                    6.50 usec
TE                   298.0 K
D1              1.00000000 sec
TD0                      1

======== CHANNEL f1 ========
SFO1           400.1424710 MHz
NUC1                    1H
P1                   13.50 usec
PLW1           16.00000000 W

F2 - Processing parameters
SI                   65536
SF             400.1400000 MHz
WDW                     EM
SSB      0
LB                    0.30 Hz
GB       0
PC                    1.40
```

test

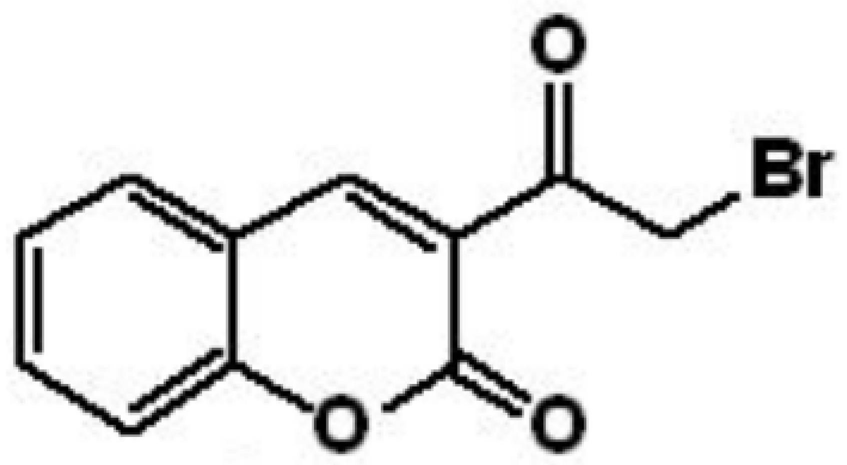

3

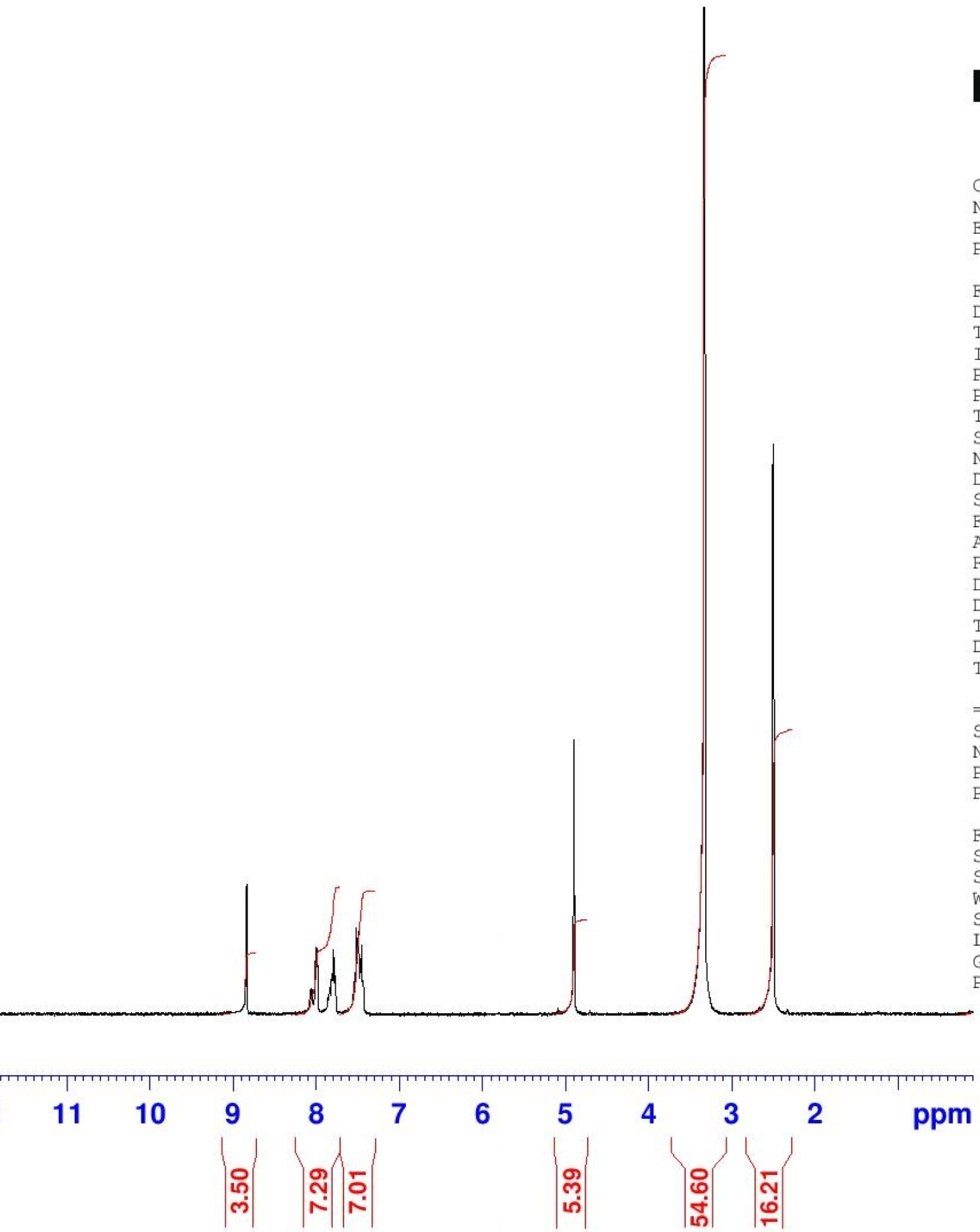

Current Data Parameters
NAME MG53
EXPNO 1
PROCNO 1

F2 - Acquisition Parameters
Date_ 20130220
Time 14.18
INSTRUM spect
PROBHD 5 mm PABBO BB-
PULPROG zg30
TD 65536
SOLVENT DMSO
NS 8
DS 2
SWH 8012.820 Hz
FIDRES 0.122266 Hz
AQ 4.0894465 sec
RG 203
DW 62.400 usec
DE 6.50 usec
TE 298.0 K
D1 1.00000000 sec
TD0 1

======== CHANNEL f1 ========
SFO1 400.1424710 MHz
NUC1 1H
P1 13.50 usec
PLW1 16.00000000 W

F2 - Processing parameters
SI 65536
SF 400.1400000 MHz
WDW EM
SSB 0
LB 0.30 Hz
GB 0
PC 1.00

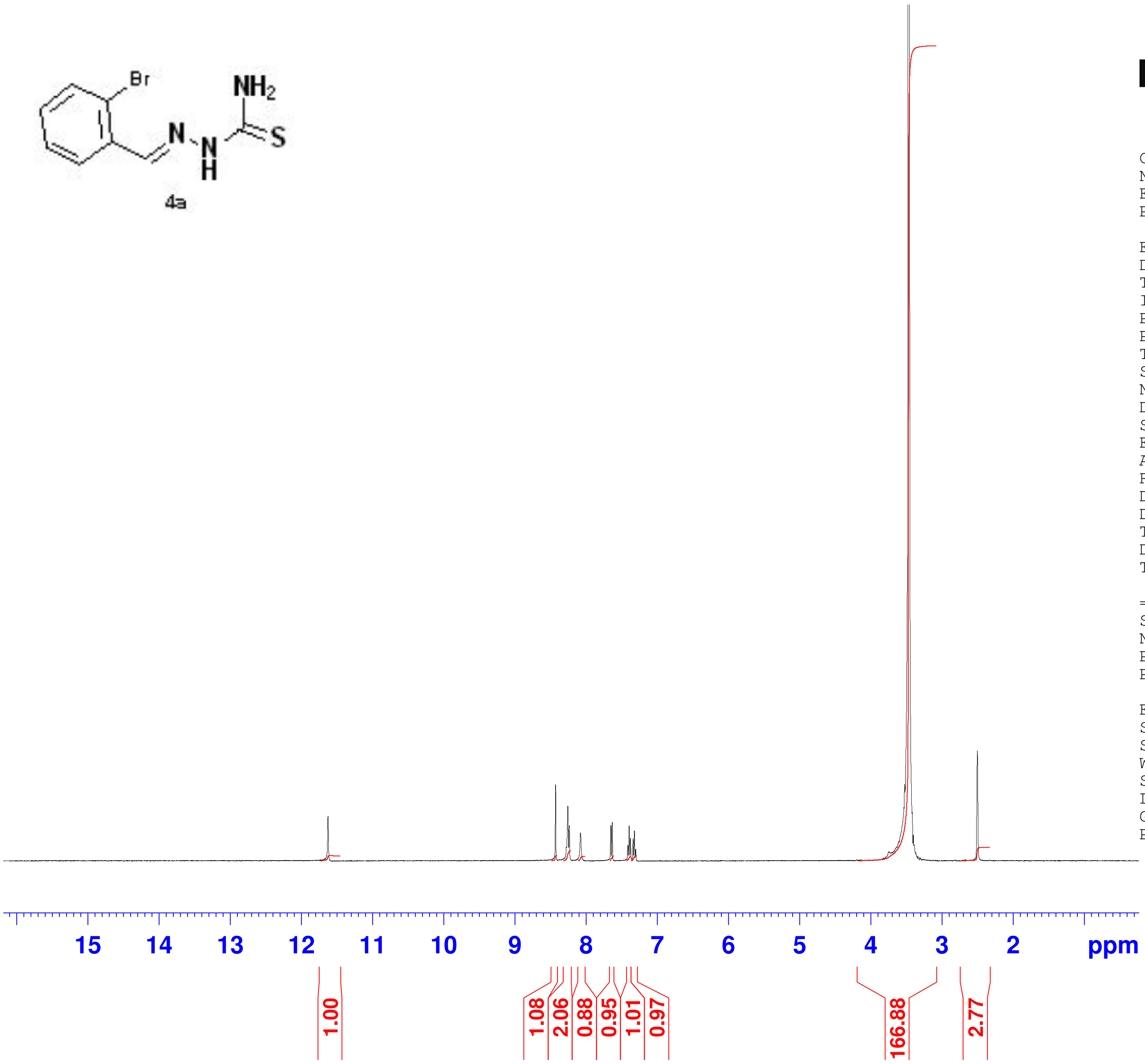

Current Data Parameters	
NAME	MG115
EXPNO	1
PROCNO	1

F2 - Acquisition Parameters	
Date_	20130329
Time	18.27
INSTRUM	spect
PROBHD	5 mm PABBO BB-
PULPROG	zg30
TD	65536
SOLVENT	DMSO
NS	3
DS	2
SWH	8012.820 Hz
FIDRES	0.122266 Hz
AQ	4.0894465 sec
RG	64
DW	62.400 usec
DE	6.50 usec
TE	298.1 K
D1	1.00000000 sec
TD0	1

======== CHANNEL f1 ========	
SFO1	400.1424710 MHz
NUC1	1H
P1	13.50 usec
PLW1	16.00000000 W

F2 - Processing parameters	
SI	65536
SF	400.1400000 MHz
WDW	EM
SSB	0
LB	0.30 Hz
GB	0
PC	1.00

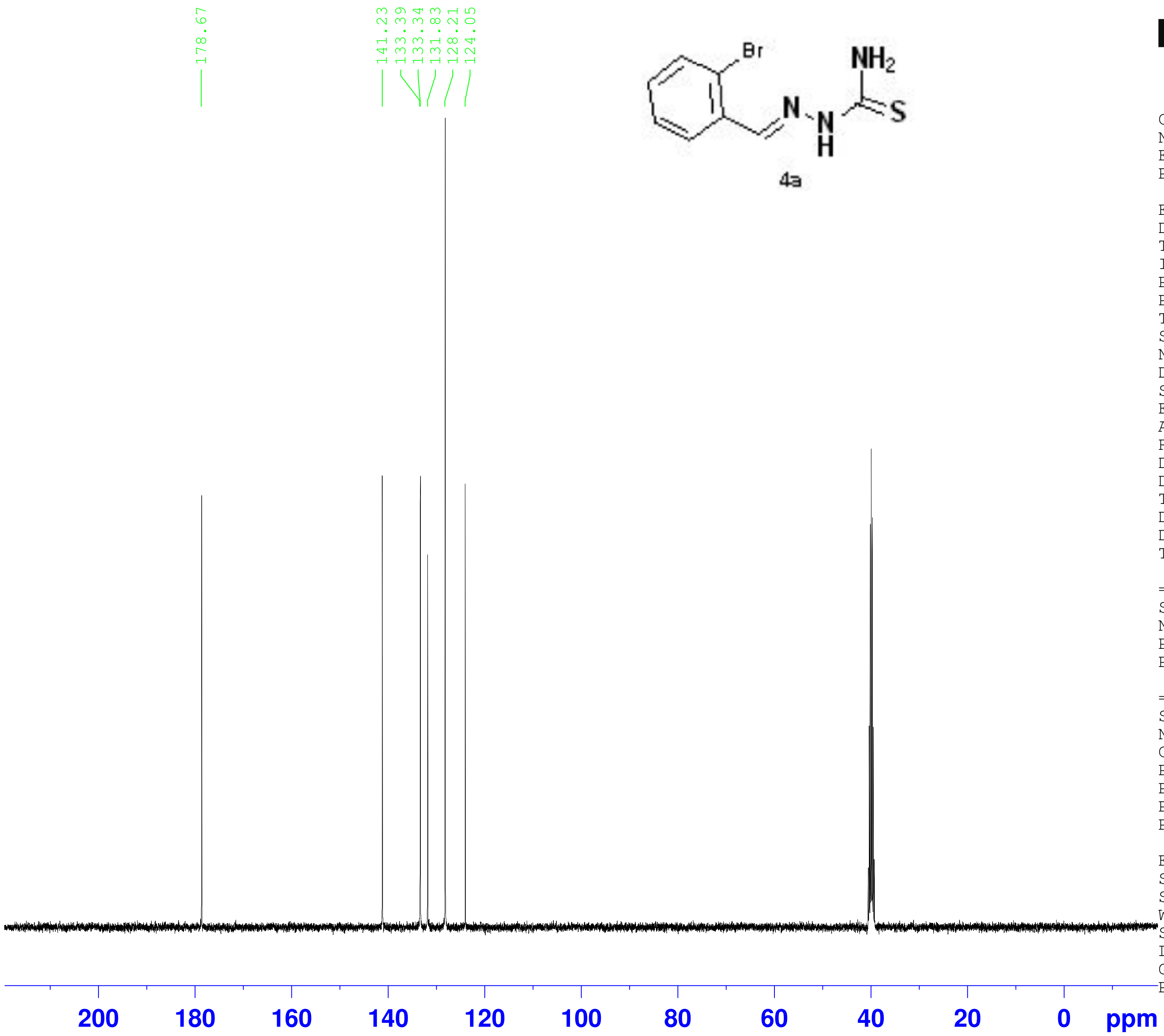

4a

Current Data Parameters
NAME MG-I-115c
EXPNO 1
PROCNO 1

F2 - Acquisition Parameters
Date_ 20130524
Time 16.29
INSTRUM spect
PROBHD 5 mm PABBO BB-
PULPROG zgpg30
TD 65536
SOLVENT DMSO
NS 61
DS 4
SWH 24038.461 Hz
FIDRES 0.366798 Hz
AQ 1.3631488 sec
RG 181
DW 20.800 usec
DE 6.50 usec
TE 298.2 K
D1 2.00000000 sec
D11 0.03000000 sec
TD0 1

======== CHANNEL f1 ========
SFO1 100.6253441 MHz
NUC1 13C
P1 9.00 usec
PLW1 62.00000000 W

======== CHANNEL f2 ========
SFO2 400.1416006 MHz
NUC2 1H
CPDPRG[2 waltz16
PCPD2 90.00 usec
PLW2 16.00000000 W
PLW12 0.36000001 W
PLW13 0.29159999 W

F2 - Processing parameters
SI 32768
SF 100.6152830 MHz
WDW EM
SSB 0
LB 1.00 Hz
GB 0
PC 1.40

test

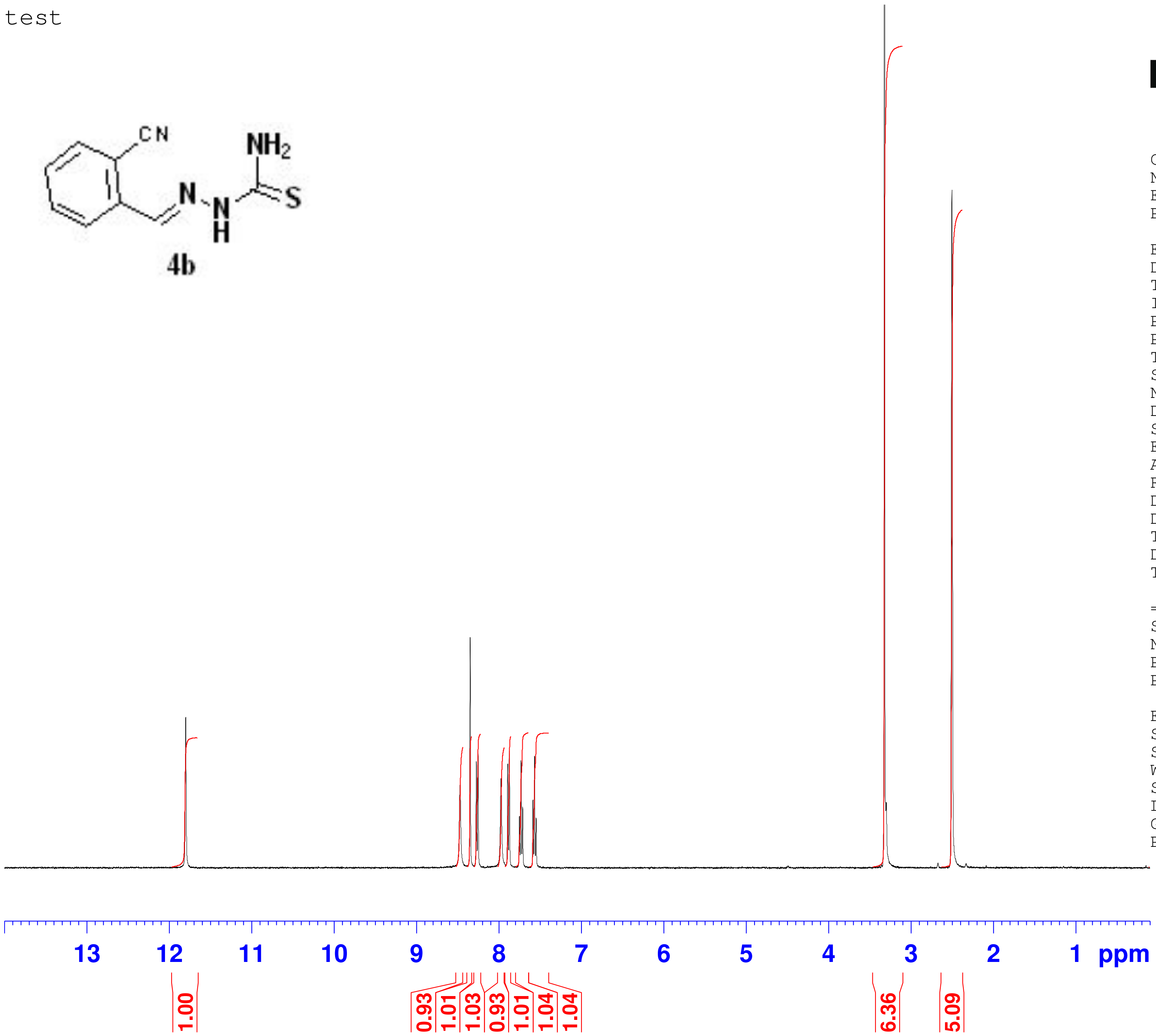

BRUKER

Current Data Parameters
NAME MG-I-143
EXPNO 1
PROCNO 1

F2 - Acquisition Parameters
Date_ 20130510
Time 16.02
INSTRUM spect
PROBHD 5 mm PABBO BB-
PULPROG zg30
TD 65536
SOLVENT DMSO
NS 8
DS 2
SWH 8012.820 Hz
FIDRES 0.122266 Hz
AQ 4.0894465 sec
RG 203
DW 62.400 usec
DE 6.50 usec
TE 298.0 K
D1 1.00000000 sec
TD0 1

======== CHANNEL f1 ========
SFO1 400.1424710 MHz
NUC1 1H
P1 13.50 usec
PLW1 16.00000000 W

F2 - Processing parameters
SI 65536
SF 400.1400000 MHz
WDW EM
SSB 0
LB 0.30 Hz
GB 0
PC 1.00

This report was created by ACD/NMR Processor Academic Edition. For more information go to www.acdlabs.com/nmrproc/

3/4/2013 5:41:51 AM

Acquisition Time (sec)	4.0894	***Comment***	5 mm PABBO BB-1H/D Z-GRD Z108618/0320	***Date***	08 Feb 2013 18:38:40
Date Stamp	08 Feb 2013 18:38:40	***File Name***	C:\Users\moustafa\Desktop\NMR folder\MG47\1\pdata\1\1r		
Frequency (MHz)	400.14	***Nucleus***	1H	***Number of Transients***	16
Origin	spect	***Original Points Count***	32768	***Owner***	mgabr
Points Count	65536	***Pulse Sequence***	zg30	***Receiver Gain***	203.00
SW(cyclical) (Hz)	8012.82	***Solvent***	DMSO-d6	***Spectrum Offset (Hz)***	2471.0281
Spectrum Type	STANDARD	***Sweep Width (Hz)***	8012.70	***Temperature (degree C)***	25.048

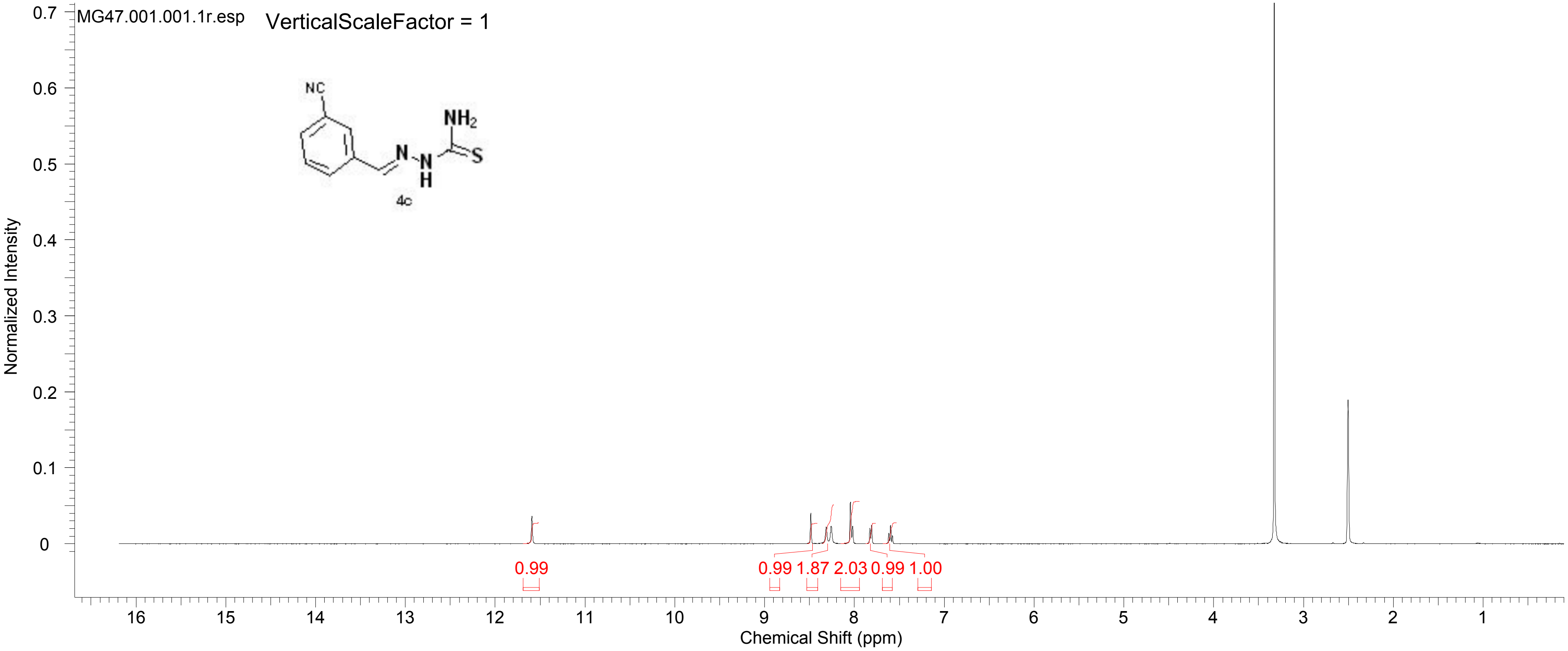

No.	(ppm)	Value	Absolute Value	Non-Negative Value
1	[7.5317 .. 7.68	1.00000000	2.74639300e+7	1.00000000
2	[7.7658 .. 7.87	0.98532182	2.70608100e+7	0.98532182
3	[7.9448 .. 8.15	2.02552295	5.56288200e+7	2.02552295
4	[8.2339 .. 8.35	1.86501074	5.12205240e+7	1.86501074
5	[8.4129 .. 8.52	0.98538440	2.70625280e+7	0.98538440
6	[11.5111 .. 11.6	0.99302292	2.72723120e+7	0.99302292

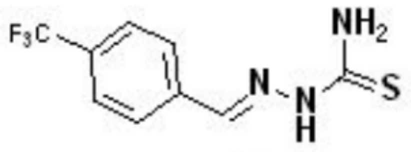

4d

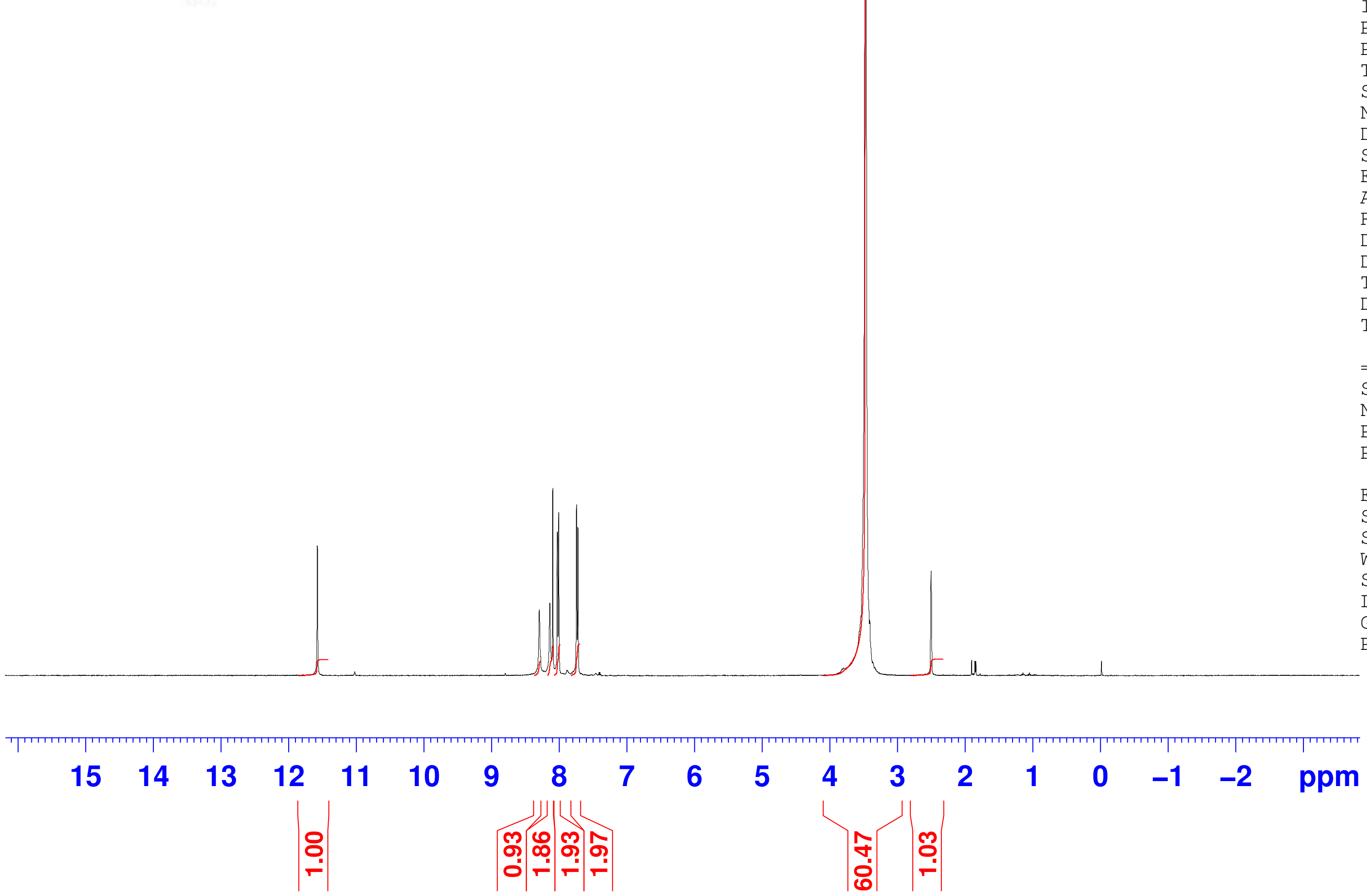

Current Data Parameters
NAME MG103
EXPNO 1
PROCNO 1

F2 - Acquisition Parameters
Date_ 20130322
Time 18.23
INSTRUM spect
PROBHD 5 mm PABBO BB-
PULPROG zg30
TD 65536
SOLVENT DMSO
NS 3
DS 2
SWH 8012.820 Hz
FIDRES 0.122266 Hz
AQ 4.0894465 sec
RG 57
DW 62.400 usec
DE 6.50 usec
TE 298.1 K
D1 1.00000000 sec
TD0 1

======== CHANNEL f1 ========
SFO1 400.1424710 MHz
NUC1 1H
P1 13.50 usec
PLW1 16.00000000 W

F2 - Processing parameters
SI 65536
SF 400.1400000 MHz
WDW EM
SSB 0
LB 0.30 Hz
GB 0
PC 1.40

test

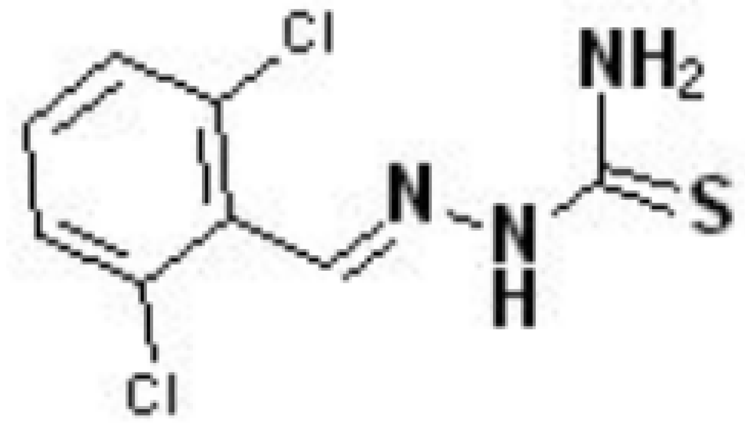

4f

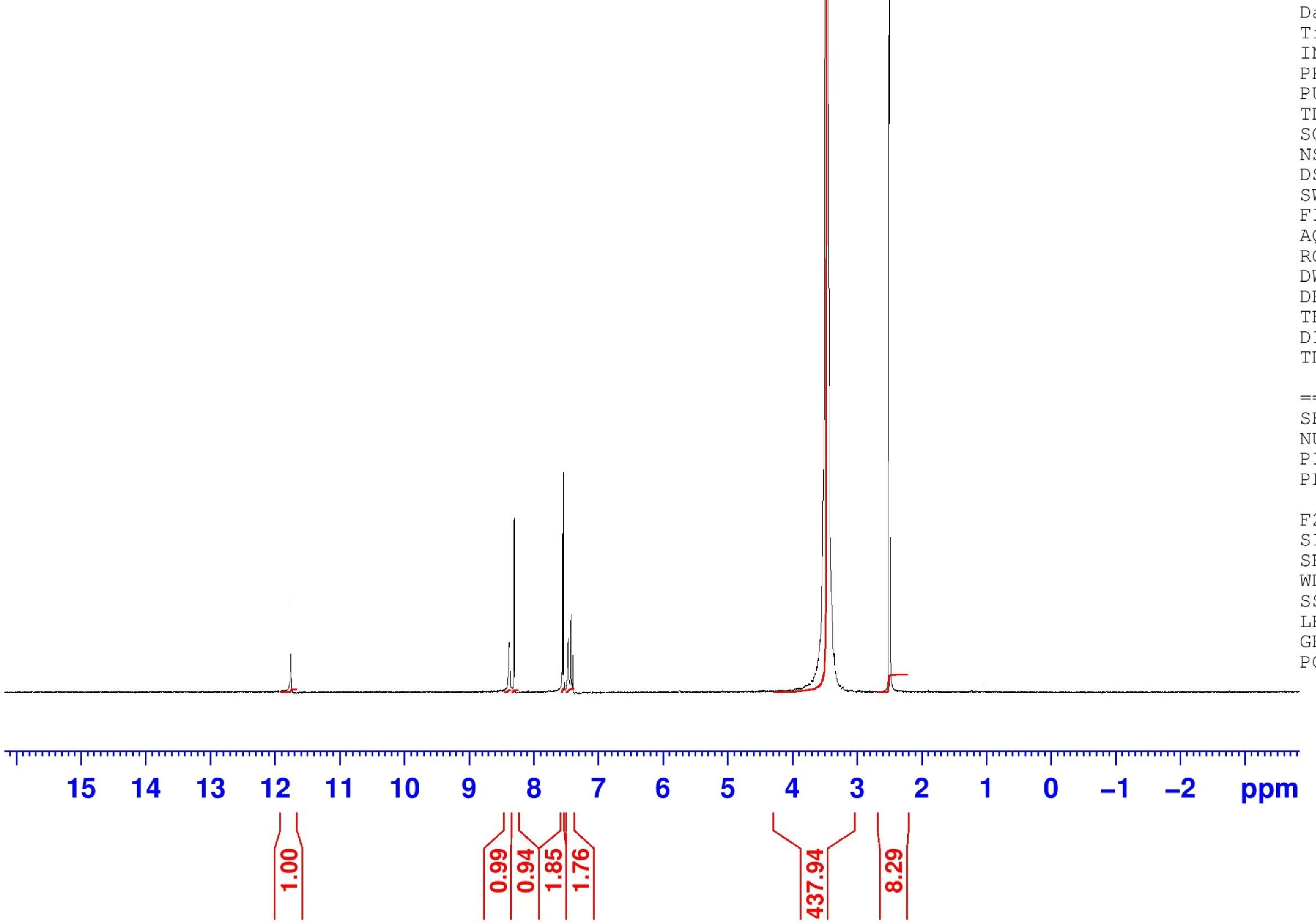


```
Current Data Parameters
NAME                 MG72
EXPNO                   1
PROCNO                  1

F2 - Acquisition Parameters
Date_            20130308
Time                17.40
INSTRUM             spect
PROBHD   5 mm PABBO BB-
PULPROG              zg30
TD                  65536
SOLVENT              DMSO
NS                     16
DS                      2
SWH              8012.820 Hz
FIDRES           0.122266 Hz
AQ              4.0894465 sec
RG                   71.8
DW                 62.400 usec
DE                   6.50 usec
TE                  293.1 K
D1             1.00000000 sec
TD0                     1

======== CHANNEL f1 ========
SFO1          400.1424710 MHz
NUC1                   1H
P1                  13.50 usec
PLW1          16.00000000 W

F2 - Processing parameters
SI                  65536
SF            400.1400000 MHz
WDW                    EM
SSB      0
LB                   0.30 Hz
GB       0
PC                   1.00
```

test

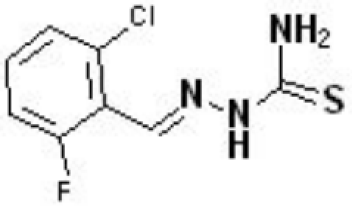

4g

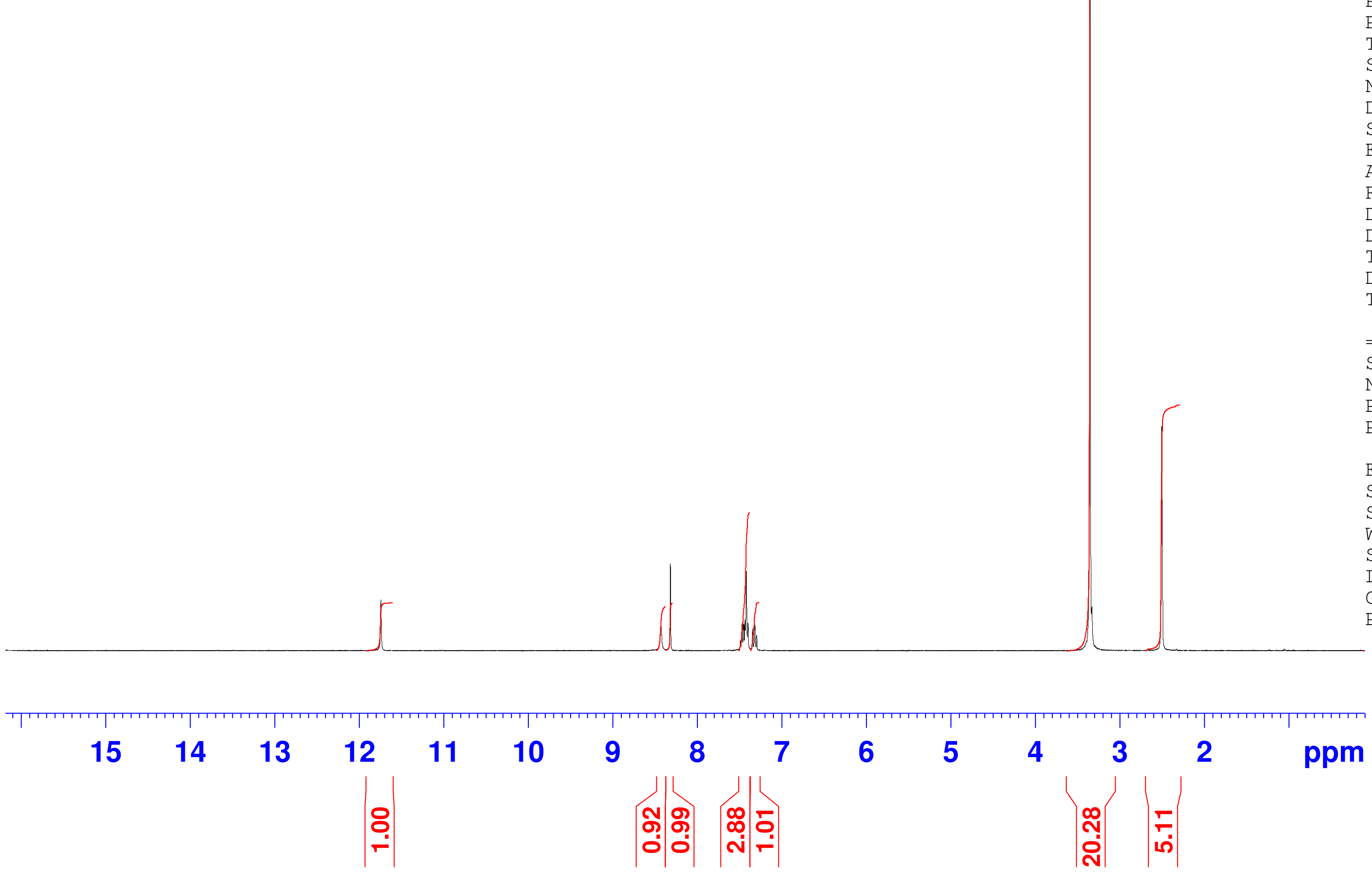

Current Data Parameters
NAME MG80
EXPNO 1
PROCNO 1

F2 - Acquisition Parameters
Date_ 20130314
Time 18.23
INSTRUM spect
PROBHD 5 mm PABBO BB-
PULPROG zg30
TD 65536
SOLVENT DMSO
NS 16
DS 2
SWH 8012.820 Hz
FIDRES 0.122266 Hz
AQ 4.0894465 sec
RG 203
DW 62.400 usec
DE 6.50 usec
TE 293.1 K
D1 1.00000000 sec
TD0 1

======== CHANNEL f1 ========
SFO1 400.1424710 MHz
NUC1 1H
P1 13.50 usec
PLW1 16.00000000 W

F2 - Processing parameters
SI 65536
SF 400.1400000 MHz
WDW EM
SSB 0
LB 0.30 Hz
GB 0
PC 1.00

test

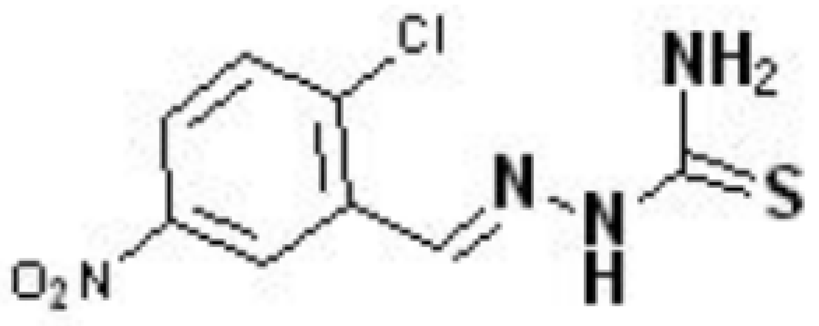

4h

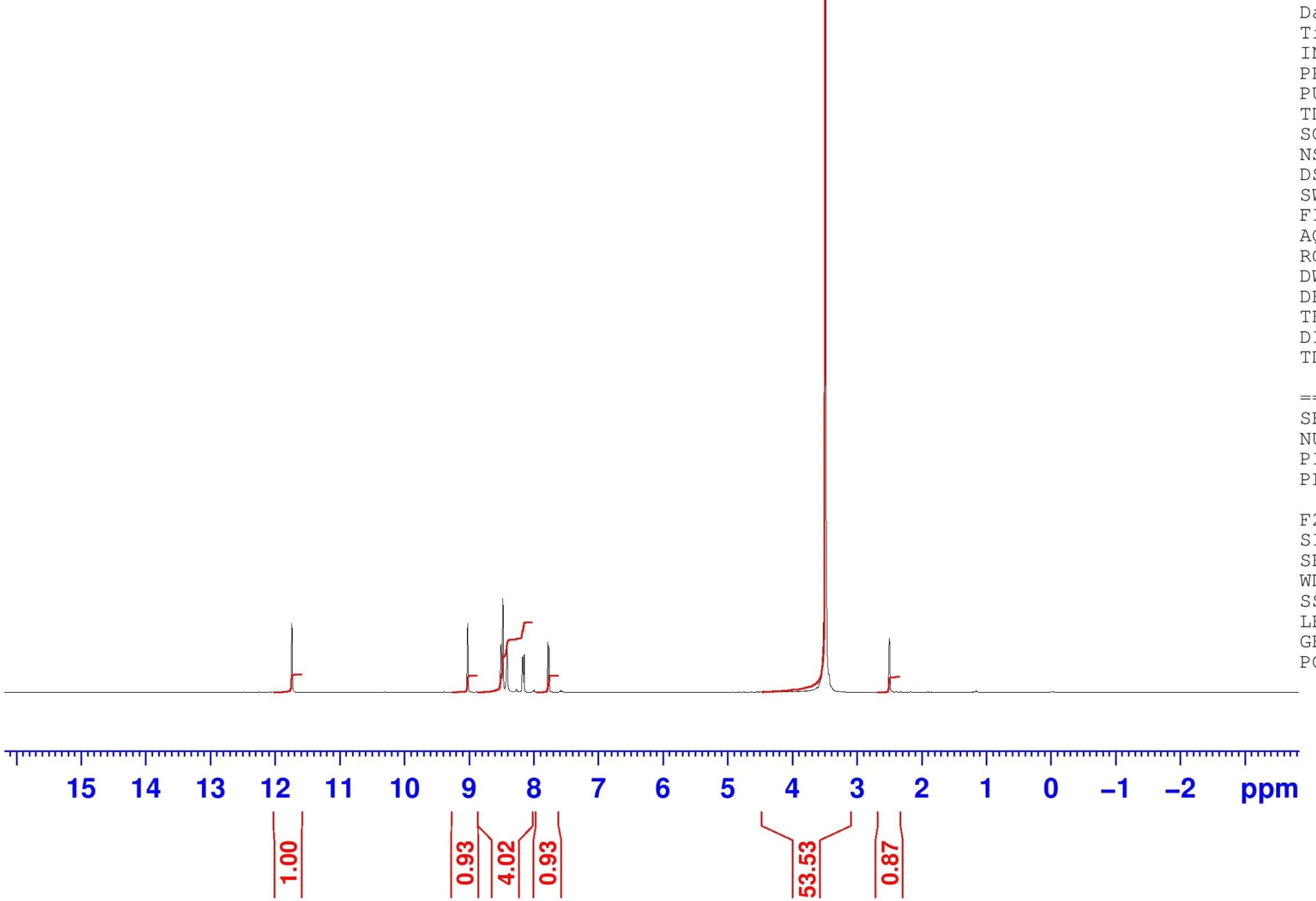


```
Current Data Parameters
NAME                 MG73
EXPNO                   1
PROCNO                  1

F2 - Acquisition Parameters
Date_            20130307
Time                12.47
INSTRUM             spect
PROBHD   5 mm PABBO BB-
PULPROG              zg30
TD                  65536
SOLVENT              DMSO
NS                     16
DS                      2
SWH              8012.820 Hz
FIDRES           0.122266 Hz
AQ              4.0894465 sec
RG                     57
DW                 62.400 usec
DE                   6.50 usec
TE                  292.6 K
D1             1.00000000 sec
TD0                     1

======== CHANNEL f1 ========
SFO1          400.1424710 MHz
NUC1                   1H
P1                  13.50 usec
PLW1          16.00000000 W

F2 - Processing parameters
SI                  65536
SF            400.1400000 MHz
WDW                    EM
SSB      0
LB                   0.30 Hz
GB       0
PC                   1.00
```

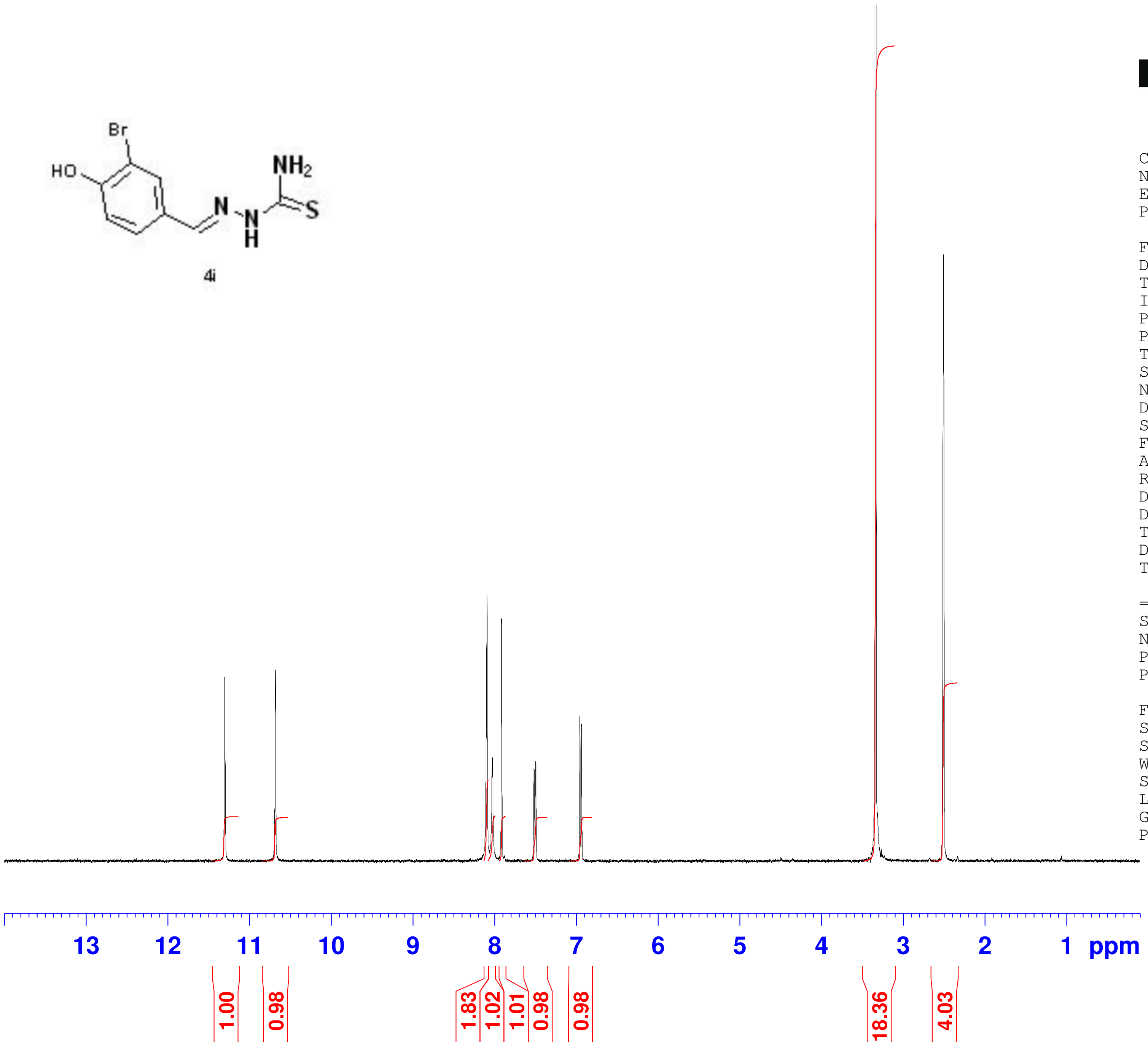

Current Data Parameters
NAME MG-II-21
EXPNO 1
PROCNO 1

F2 - Acquisition Parameters
Date_ 20130509
Time 18.23
INSTRUM spect
PROBHD 5 mm PABBO BB-
PULPROG zg30
TD 65536
SOLVENT DMSO
NS 3
DS 2
SWH 8012.820 Hz
FIDRES 0.122266 Hz
AQ 4.0894465 sec
RG 203
DW 62.400 usec
DE 6.50 usec
TE 298.1 K
D1 1.00000000 sec
TD0 1

======== CHANNEL f1 ========
SFO1 400.1424710 MHz
NUC1 1H
P1 13.50 usec
PLW1 16.00000000 W

F2 - Processing parameters
SI 65536
SF 400.1400000 MHz
WDW EM
SSB 0
LB 0.30 Hz
GB 0
PC 1.00

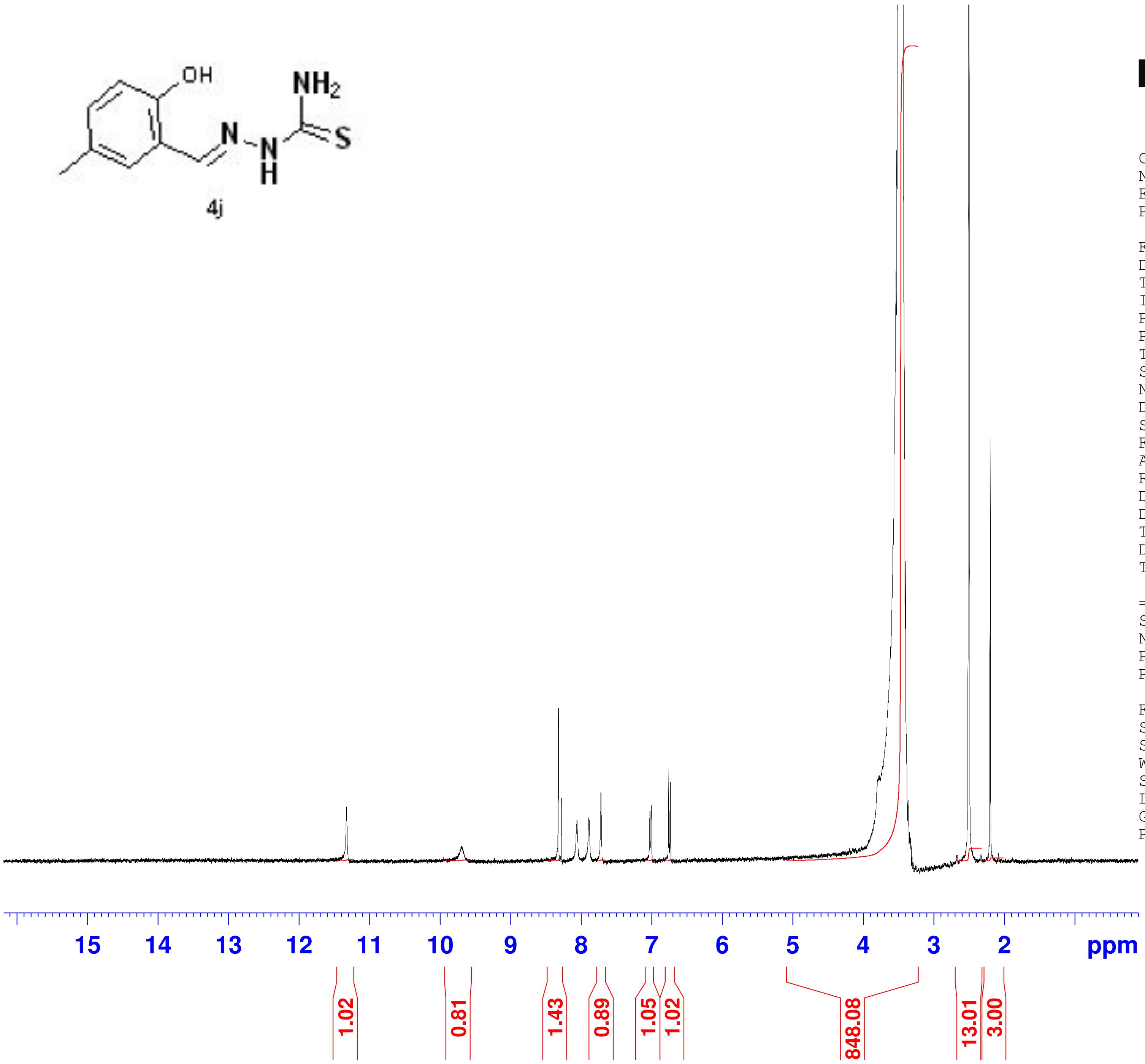

BRUKER

```
Current Data Parameters
NAME                 MG108
EXPNO                    1
PROCNO                   1

F2 - Acquisition Parameters
Date_             20130329
Time                 18.06
INSTRUM              spect
PROBHD    5 mm PABBO BB-
PULPROG               zg30
TD                   65536
SOLVENT               DMSO
NS                      16
DS                       2
SWH               8012.820 Hz
FIDRES            0.122266 Hz
AQ               4.0894465 sec
RG                      64
DW                  62.400 usec
DE                    6.50 usec
TE                   298.0 K
D1              1.00000000 sec
TD0                      1

======== CHANNEL f1 ========
SFO1           400.1424710 MHz
NUC1                    1H
P1                   13.50 usec
PLW1           16.00000000 W

F2 - Processing parameters
SI                   65536
SF             400.1400000 MHz
WDW                     EM
SSB       0
LB                    0.30 Hz
GB        0
PC                    1.40
```

test

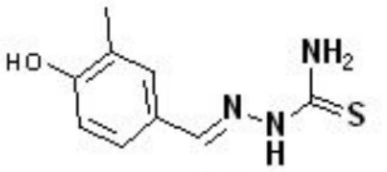

4k

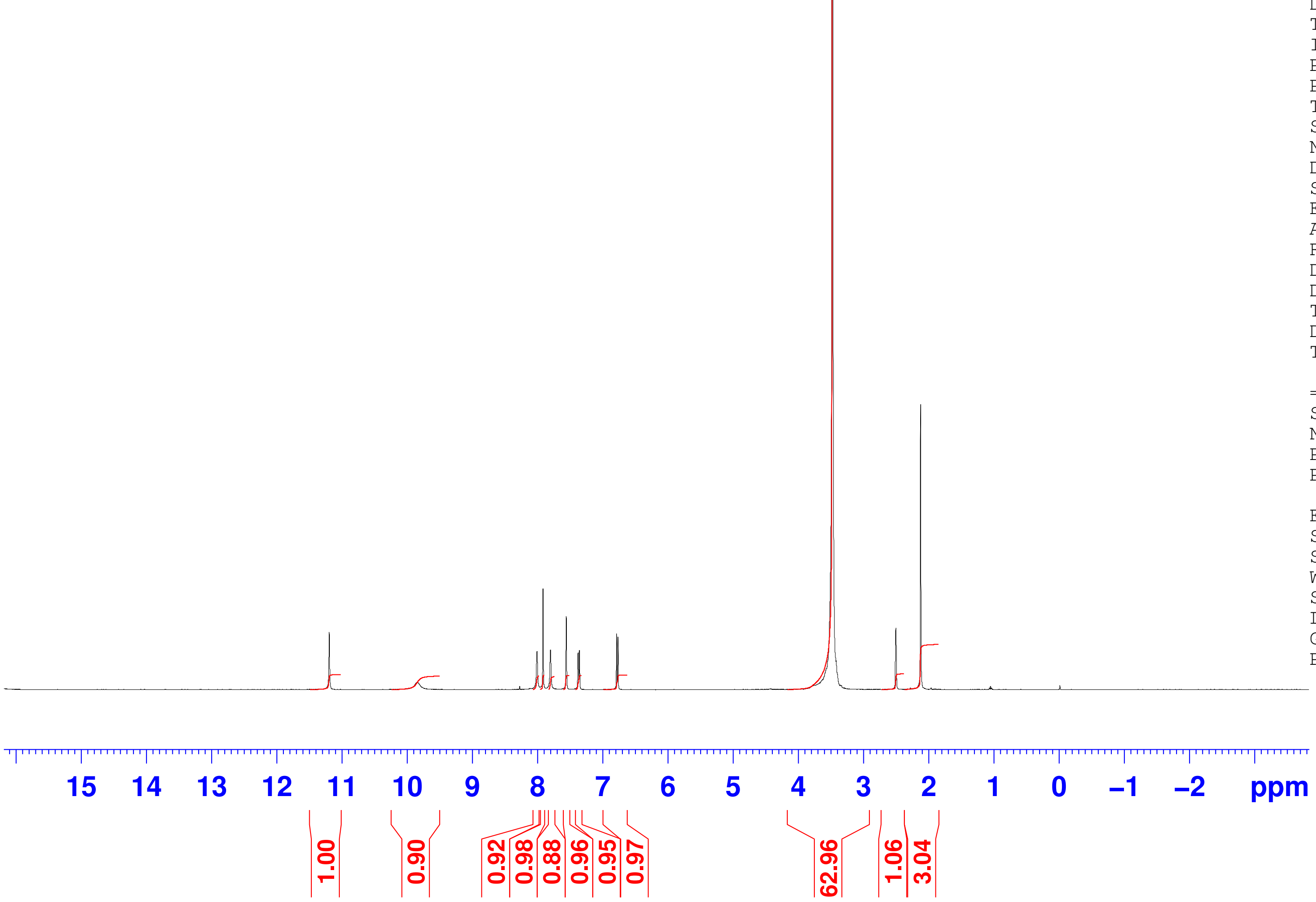


```
Current Data Parameters
NAME              MG83
EXPNO                1
PROCNO               1

F2 - Acquisition Parameters
Date_         20130327
Time             16.33
INSTRUM          spect
PROBHD   5 mm PABBO BB-
PULPROG           zg30
TD               65536
SOLVENT           DMSO
NS                   4
DS                   2
SWH           8012.820 Hz
FIDRES        0.122266 Hz
AQ           4.0894465 sec
RG                  57
DW              62.400 usec
DE                6.50 usec
TE               298.1 K
D1          1.00000000 sec
TD0                  1

======== CHANNEL f1 ========
SFO1       400.1424710 MHz
NUC1                1H
P1               13.50 usec
PLW1       16.00000000 W

F2 - Processing parameters
SI               65536
SF         400.1400000 MHz
WDW                 EM
SSB      0
LB                0.30 Hz
GB       0
PC                1.40
```

test

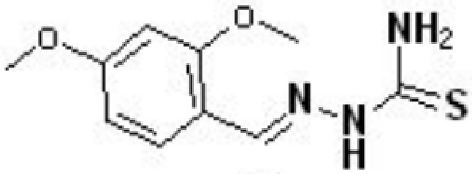

4l

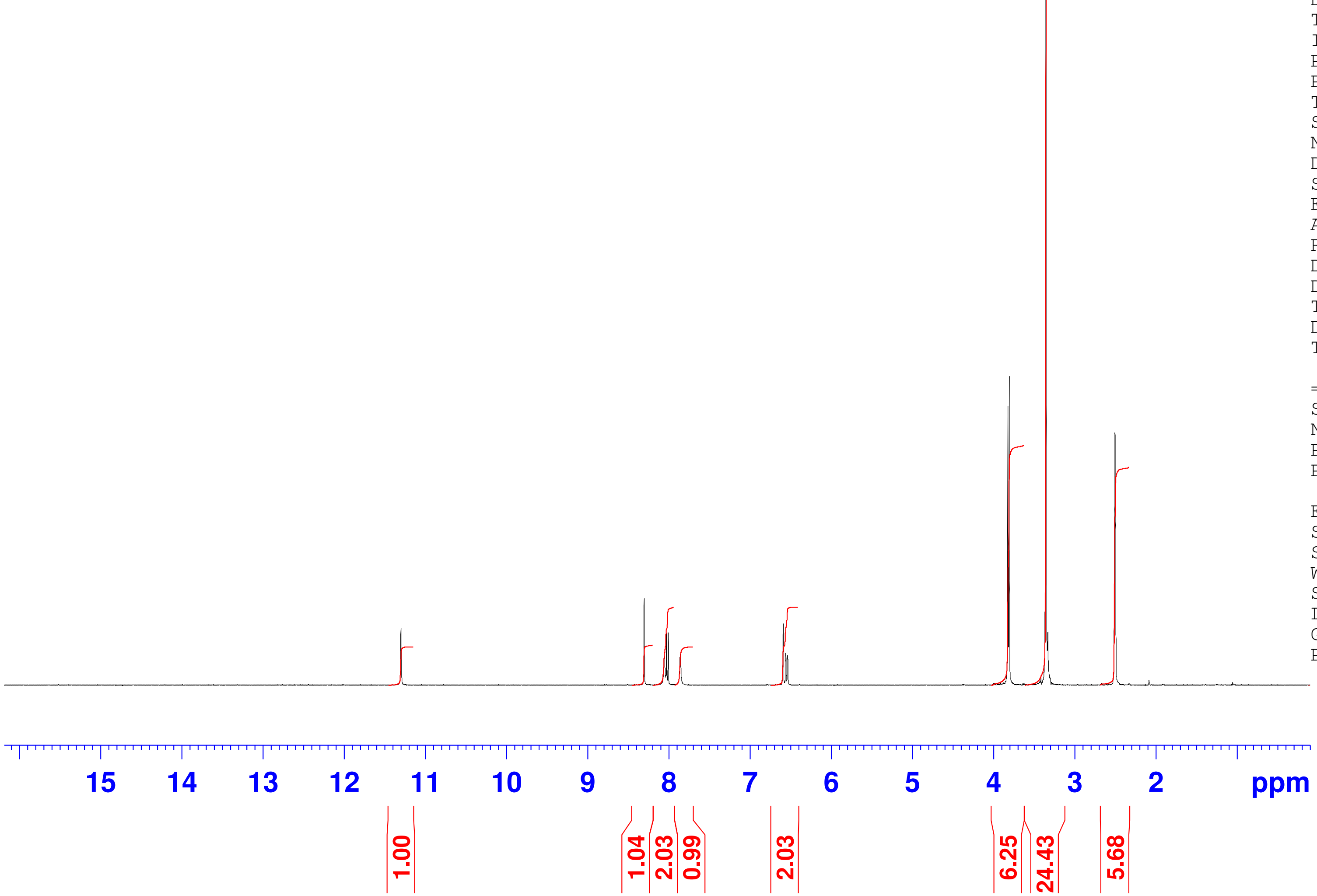

BRUKER

Current Data Parameters
NAME MG64p
EXPNO 1
PROCNO 1

F2 - Acquisition Parameters
Date_ 20130314
Time 18.18
INSTRUM spect
PROBHD 5 mm PABBO BB-
PULPROG zg30
TD 65536
SOLVENT DMSO
NS 16
DS 2
SWH 8012.820 Hz
FIDRES 0.122266 Hz
AQ 4.0894465 sec
RG 203
DW 62.400 usec
DE 6.50 usec
TE 293.1 K
D1 1.00000000 sec
TD0 1

======== CHANNEL f1 ========
SFO1 400.1424710 MHz
NUC1 1H
P1 13.50 usec
PLW1 16.00000000 W

F2 - Processing parameters
SI 65536
SF 400.1400000 MHz
WDW EM
SSB 0
LB 0.30 Hz
GB 0
PC 1.00

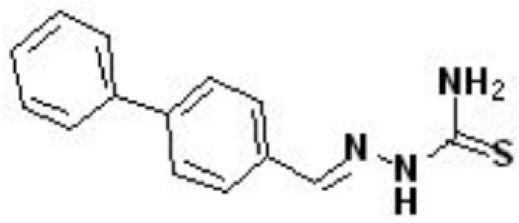

4n

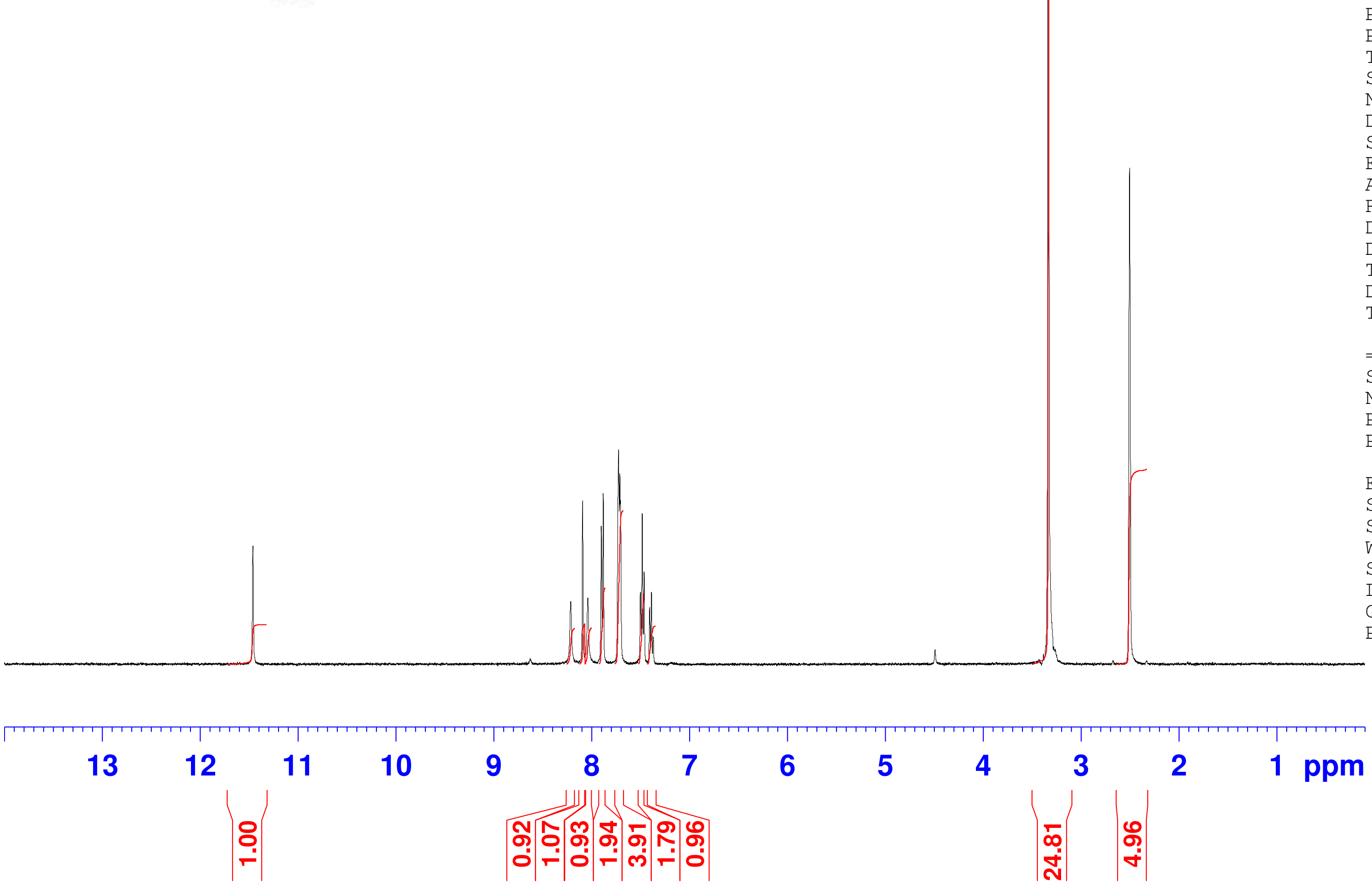

BRUKER

```
Current Data Parameters
NAME              MG-I-141
EXPNO                    1
PROCNO                   1

F2 - Acquisition Parameters
Date_             20130509
Time                 18.03
INSTRUM              spect
PROBHD    5 mm PABBO BB-
PULPROG               zg30
TD                   65536
SOLVENT               DMSO
NS                       3
DS                       2
SWH               8012.820 Hz
FIDRES            0.122266 Hz
AQ               4.0894465 sec
RG                     203
DW                  62.400 usec
DE                    6.50 usec
TE                   298.0 K
D1              1.00000000 sec
TD0                      1

======== CHANNEL f1 ========
SFO1           400.1424710 MHz
NUC1                    1H
P1                   13.50 usec
PLW1           16.00000000 W

F2 - Processing parameters
SI                   65536
SF             400.1400000 MHz
WDW                     EM
SSB       0
LB                    0.30 Hz
GB        0
PC                    1.00
```

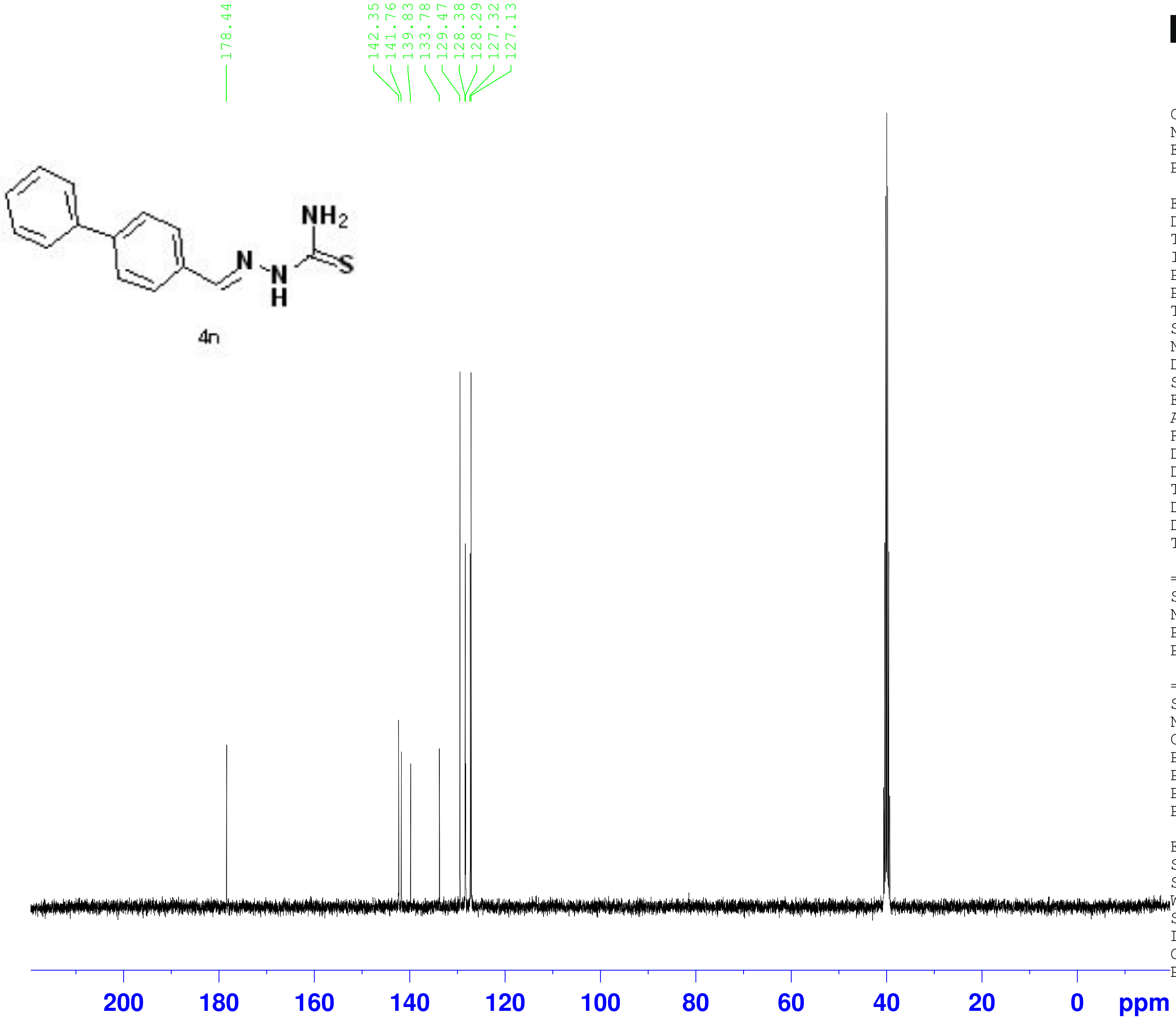

Current Data Parameters
NAME MG-I-141c
EXPNO 1
PROCNO 1

F2 - Acquisition Parameters
Date_ 20130515
Time 18.00
INSTRUM spect
PROBHD 5 mm PABBO BB-
PULPROG zgpg30
TD 65536
SOLVENT DMSO
NS 70
DS 4
SWH 24038.461 Hz
FIDRES 0.366798 Hz
AQ 1.3631488 sec
RG 144
DW 20.800 usec
DE 6.50 usec
TE 298.2 K
D1 2.00000000 sec
D11 0.03000000 sec
TD0 1

======== CHANNEL f1 ========
SFO1 100.6253441 MHz
NUC1 13C
P1 9.00 usec
PLW1 62.00000000 W

======== CHANNEL f2 ========
SFO2 400.1416006 MHz
NUC2 1H
CPDPRG[2 waltz16
PCPD2 90.00 usec
PLW2 16.00000000 W
PLW12 0.36000001 W
PLW13 0.29159999 W

F2 - Processing parameters
SI 32768
SF 100.6152830 MHz
WDW EM
SSB 0
LB 1.00 Hz
GB 0
PC 1.40

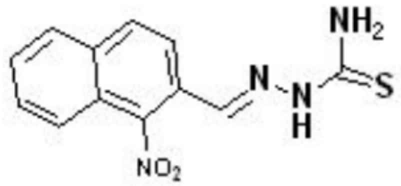

4p

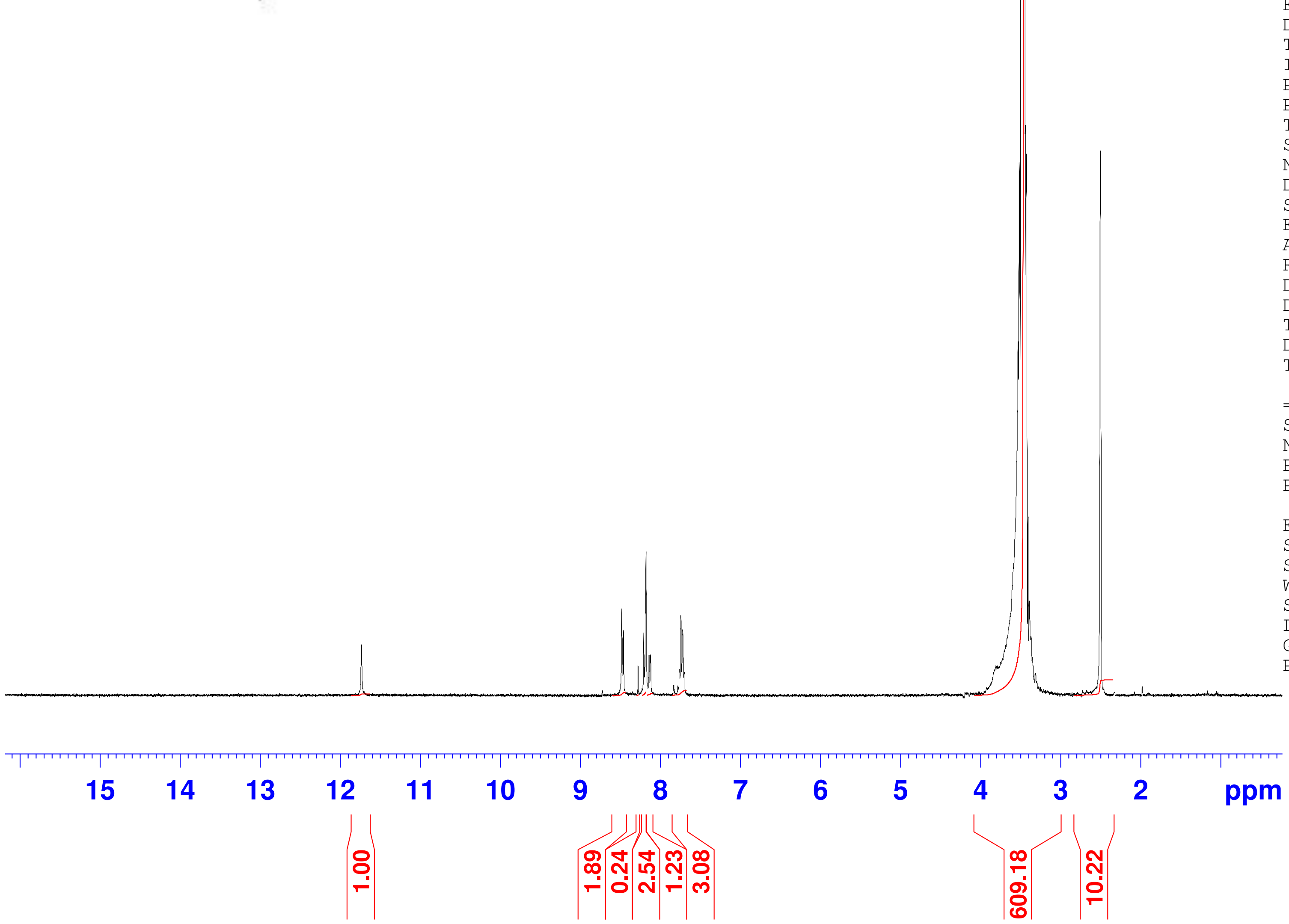

BRUKER

Current Data Parameters
NAME MG100
EXPNO 1
PROCNO 1

F2 - Acquisition Parameters
Date_ 20130329
Time 18.19
INSTRUM spect
PROBHD 5 mm PABBO BB-
PULPROG zg30
TD 65536
SOLVENT DMSO
NS 4
DS 2
SWH 8012.820 Hz
FIDRES 0.122266 Hz
AQ 4.0894465 sec
RG 64
DW 62.400 usec
DE 6.50 usec
TE 298.0 K
D1 1.00000000 sec
TD0 1

======== CHANNEL f1 ========
SFO1 400.1424710 MHz
NUC1 1H
P1 13.50 usec
PLW1 16.00000000 W

F2 - Processing parameters
SI 65536
SF 400.1400000 MHz
WDW EM
SSB 0
LB 0.30 Hz
GB 0
PC 1.00

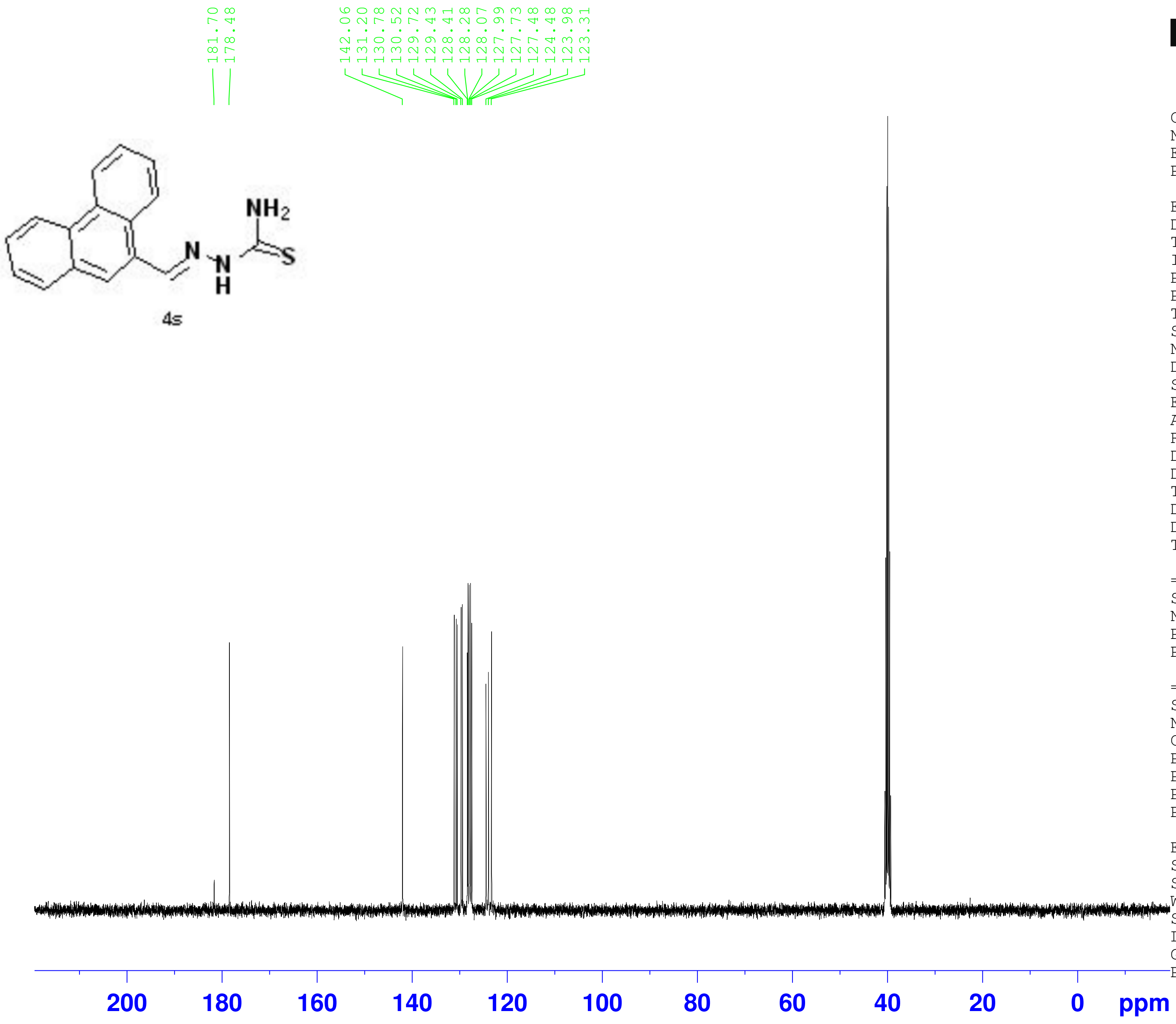

BRUKER

Current Data Parameters
NAME MG-I-146c
EXPNO 1
PROCNO 1

F2 - Acquisition Parameters
Date_ 20130524
Time 16.23
INSTRUM spect
PROBHD 5 mm PABBO BB-
PULPROG zgpg30
TD 65536
SOLVENT DMSO
NS 84
DS 4
SWH 24038.461 Hz
FIDRES 0.366798 Hz
AQ 1.3631488 sec
RG 161
DW 20.800 usec
DE 6.50 usec
TE 298.0 K
D1 2.00000000 sec
D11 0.03000000 sec
TD0 1

======== CHANNEL f1 ========
SFO1 100.6253441 MHz
NUC1 13C
P1 9.00 usec
PLW1 62.00000000 W

======== CHANNEL f2 ========
SFO2 400.1416006 MHz
NUC2 1H
CPDPRG[2 waltz16
PCPD2 90.00 usec
PLW2 16.00000000 W
PLW12 0.36000001 W
PLW13 0.29159999 W

F2 - Processing parameters
SI 32768
SF 100.6152830 MHz
WDW EM
SSB 0
LB 1.00 Hz
GB 0
PC 1.40

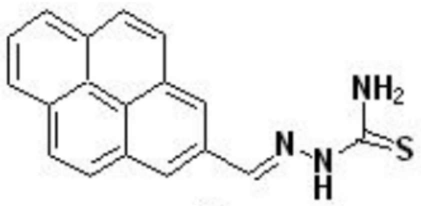

4t

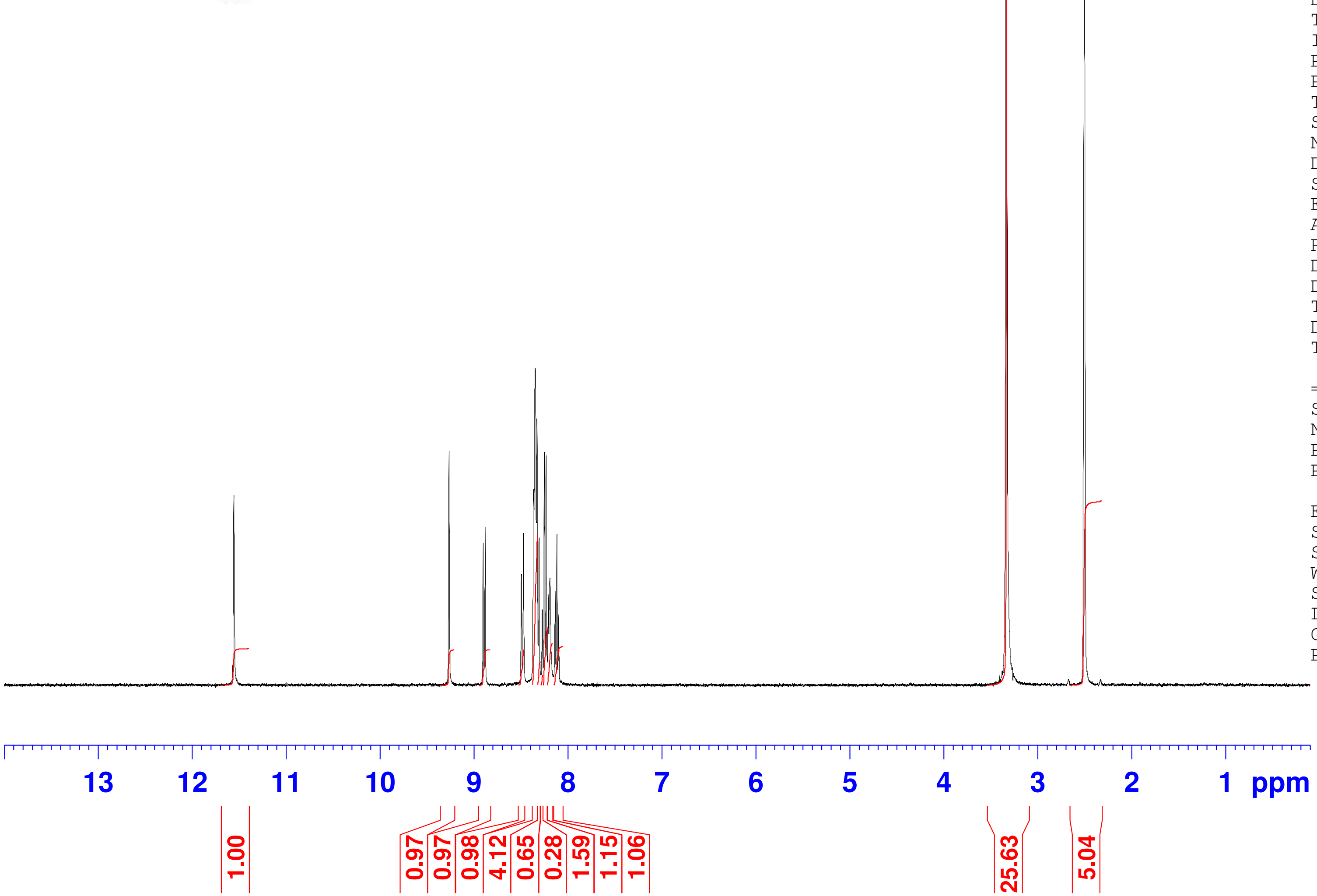


```
Current Data Parameters
NAME              MG-I-149
EXPNO                    1
PROCNO                   1

F2 - Acquisition Parameters
Date_             20130509
Time                 18.27
INSTRUM              spect
PROBHD    5 mm PABBO BB-
PULPROG               zg30
TD                   65536
SOLVENT               DMSO
NS                       6
DS                       2
SWH               8012.820 Hz
FIDRES            0.122266 Hz
AQ               4.0894465 sec
RG                     203
DW                  62.400 usec
DE                    6.50 usec
TE                   298.1 K
D1              1.00000000 sec
TD0                      1

======== CHANNEL f1 ========
SFO1           400.1424710 MHz
NUC1                    1H
P1                   13.50 usec
PLW1           16.00000000 W

F2 - Processing parameters
SI                   65536
SF             400.1400000 MHz
WDW                     EM
SSB       0
LB                    0.30 Hz
GB        0
PC                    1.00
```

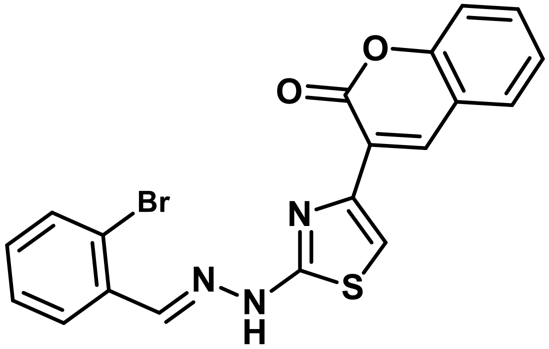

5a

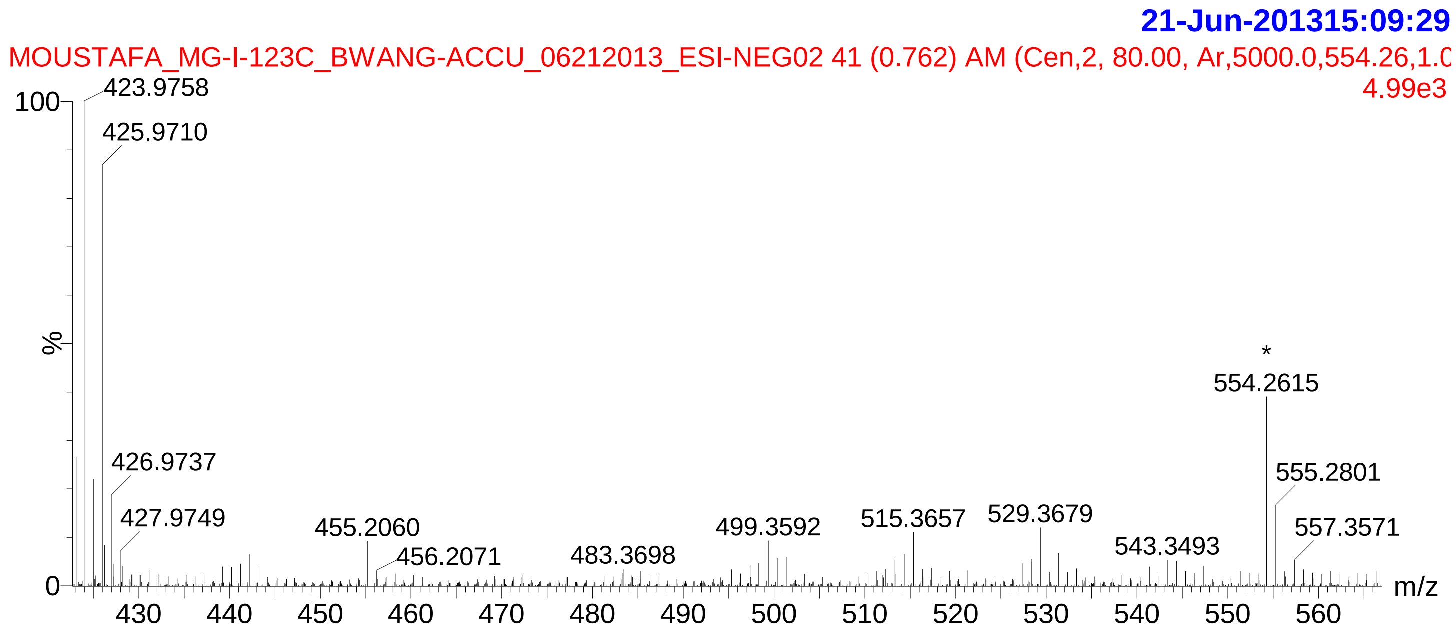

Elemental Composition Report

Single Mass Analysis
Tolerance = 5.0 PPM / DBE: min = -1.5, max = 100.0
Element prediction: Off
Number of isotope peaks used for i-FIT = 3

Monoisotopic Mass, Odd and Even Electron Ions
1136 formula(e) evaluated with 13 results within limits (all results (up to 1000) for each mass)
Elements Used:
C: 1-150 H: 1-150 N: 1-30 O: 1-60 S: 1-2 Br: 1-2

Minimum:				-1.5		
Maximum:		5.0	5.0	100.0		
Mass	Calc. Mass	mDa	PPM	DBE	i-FIT	Formula
423.9758	423.9749	0.9	2.1	6.5	121.5	C11 H15 N5 O4 S2 Br
	423.9762	-0.4	-0.9	6.0	106.9	C13 H17 N2 O5 S2 Br
	423.9776	-1.8	-4.2	11.0	79.7	C14 H13 N6 O S2 Br
	423.9742	1.6	3.8	16.0	43.3	C17 H9 N6 O S Br
	423.9755	0.3	0.7	15.5	42.1	C19 H11 N3 O2 S Br
	423.9747	1.1	2.6	9.0	222.5	C2 H5 N18 O2 S Br
	423.9747	1.1	2.6	3.5	260.5	C3 H11 N11 O7 S Br
	423.9760	-0.2	-0.5	8.5	192.6	C4 H7 N15 O3 S Br
	423.9760	-0.2	-0.5	3.0	227.9	C5 H13 N8 O8 S Br
	423.9752	0.6	1.4	1.0	959.5	C5 H18 N10 O S Br2
	423.9774	-1.6	-3.8	8.0	166.6	C6 H9 N12 O4 S Br
	423.9774	-1.6	-3.8	2.5	199.2	C7 H15 N5 O9 S Br
	423.9766	-0.8	-1.9	0.5	938.7	C7 H20 N7 O2 S Br2

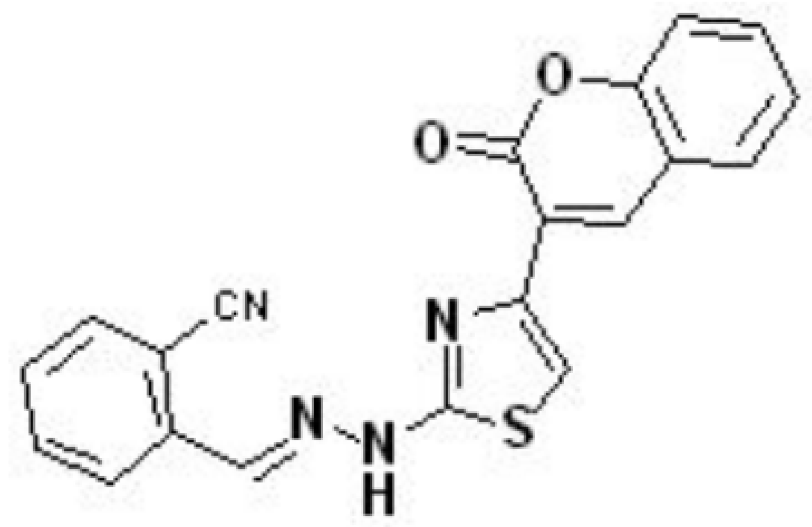

5b

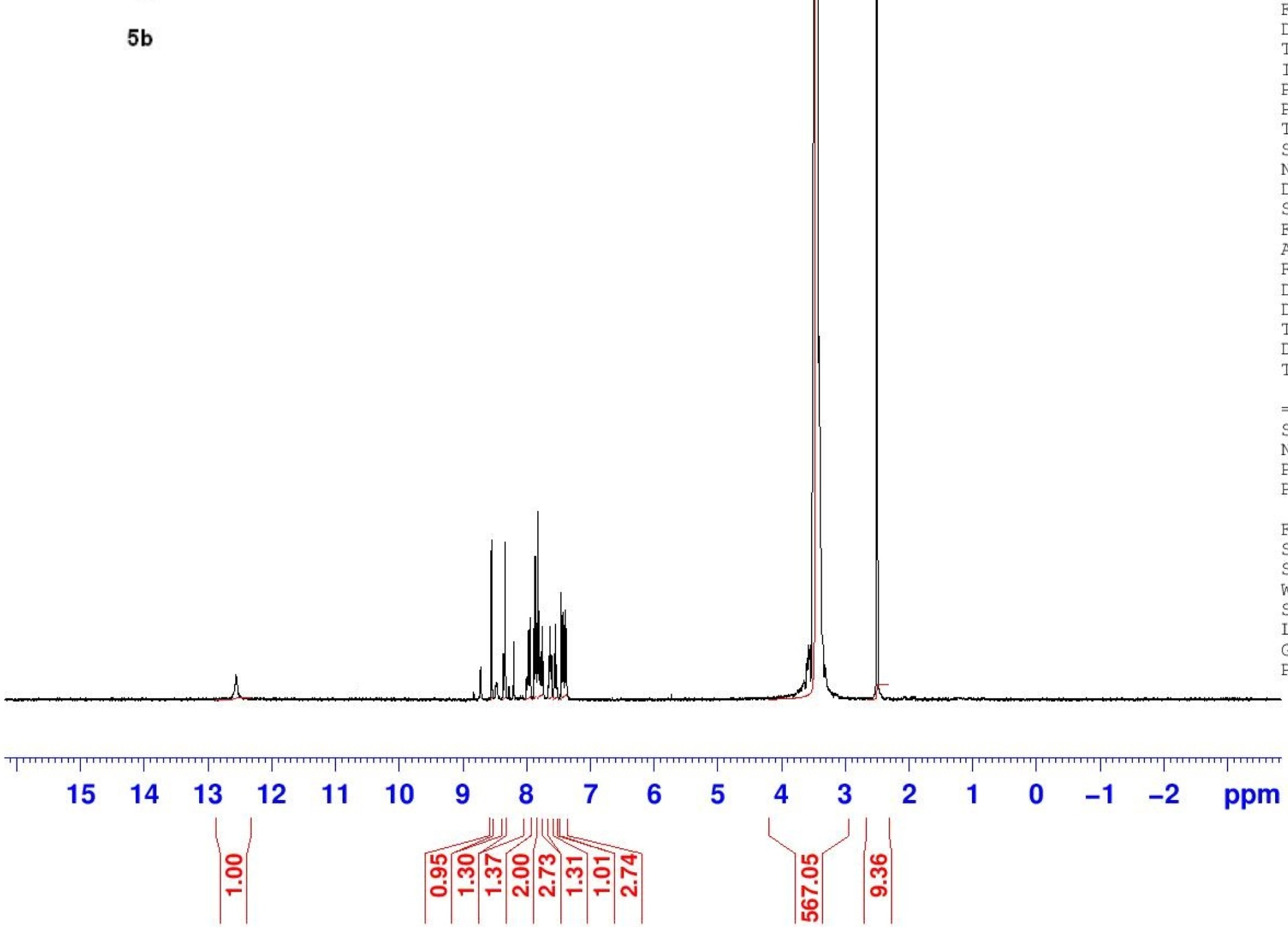


```
Current Data Parameters
NAME                MG150
EXPNO                   1
PROCNO                  1

F2 - Acquisition Parameters
Date_            20130418
Time                17.54
INSTRUM             spect
PROBHD   5 mm PABBO BB-
PULPROG              zg30
TD                  65536
SOLVENT              DMSO
NS                     16
DS                      2
SWH              8012.820 Hz
FIDRES           0.122266 Hz
AQ              4.0894465 sec
RG                     64
DW                 62.400 usec
DE                   6.50 usec
TE                  298.0 K
D1             1.00000000 sec
TD0                     1

======== CHANNEL f1 ========
SFO1          400.1424710 MHz
NUC1                   1H
P1                  13.50 usec
PLW1          16.00000000 W

F2 - Processing parameters
SI                  65536
SF            400.1400000 MHz
WDW                    EM
SSB      0
LB                   0.30 Hz
GB       0
PC                   1.40
```

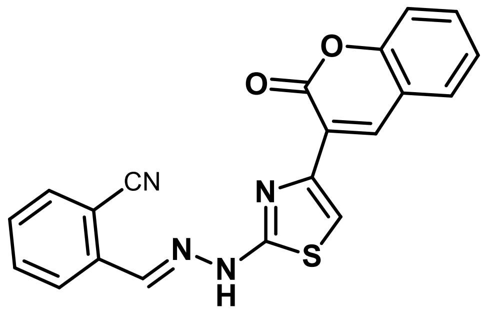

5b

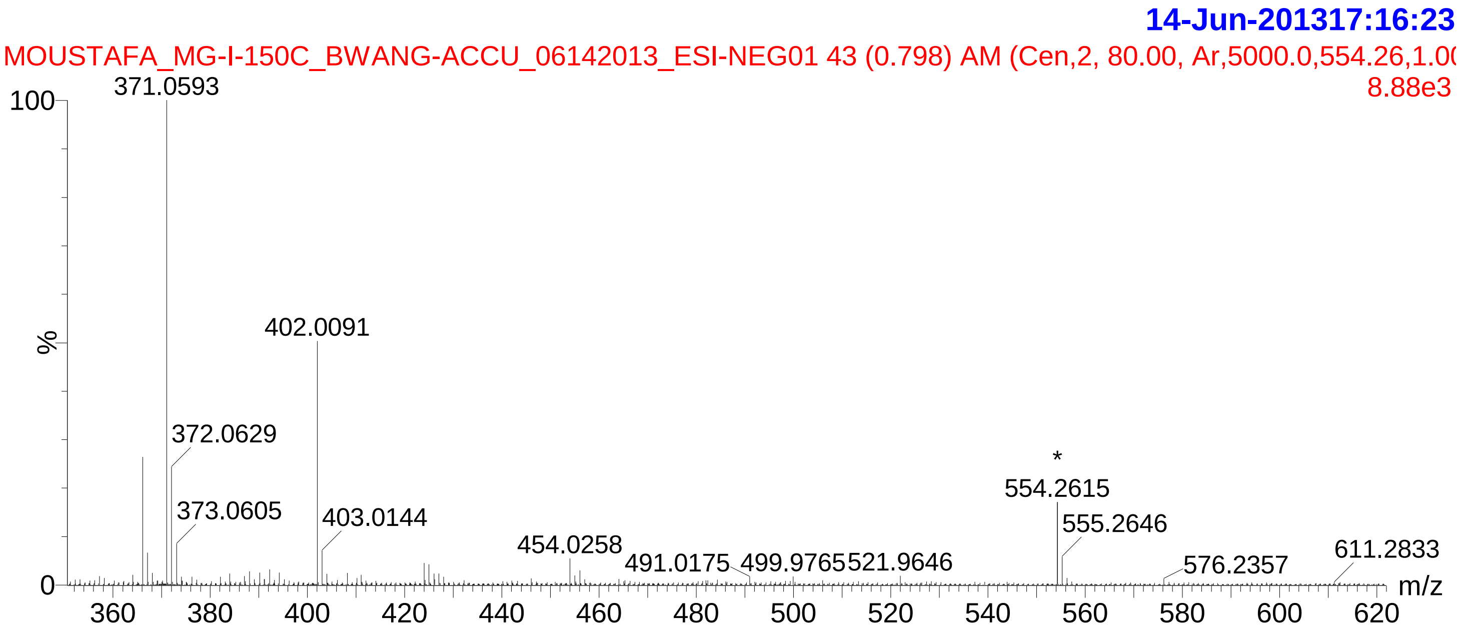

Elemental Composition Report

Single Mass Analysis
Tolerance = 5.0 PPM / DBE: min = -1.5, max = 100.0
Element prediction: Off
Number of isotope peaks used for i-FIT = 3

Monoisotopic Mass, Even Electron Ions
1064 formula(e) evaluated with 8 results within limits (all results (up to 1000) for each mass)
Elements Used:
C: 1-150 H: 1-150 N: 1-30 O: 1-60 S: 1-2

Minimum:				-1.5		
Maximum:		5.0	5.0	100.0		
Mass	Calc. Mass	mDa	PPM	DBE	i-FIT	Formula
371.0593	371.0581	1.2	3.2	11.5	406.0	C H3 N22 O S
	371.0583	1.0	2.7	3.5	203.4	C11 H19 N2 O8 S2
	371.0596	-0.3	-0.8	8.5	121.2	C12 H15 N6 O4 S2
	371.0603	-1.0	-2.7	17.5	3.0	C20 H11 N4 O2 S
	371.0581	1.2	3.2	0.5	499.5	C3 H15 N8 O11 S
	371.0594	-0.1	-0.3	5.5	371.7	C4 H11 N12 O7 S
	371.0608	-1.5	-4.0	10.5	270.4	C5 H7 N16 O3 S
	371.0608	-1.5	-4.0	-0.5	356.2	C7 H19 N2 O13 S

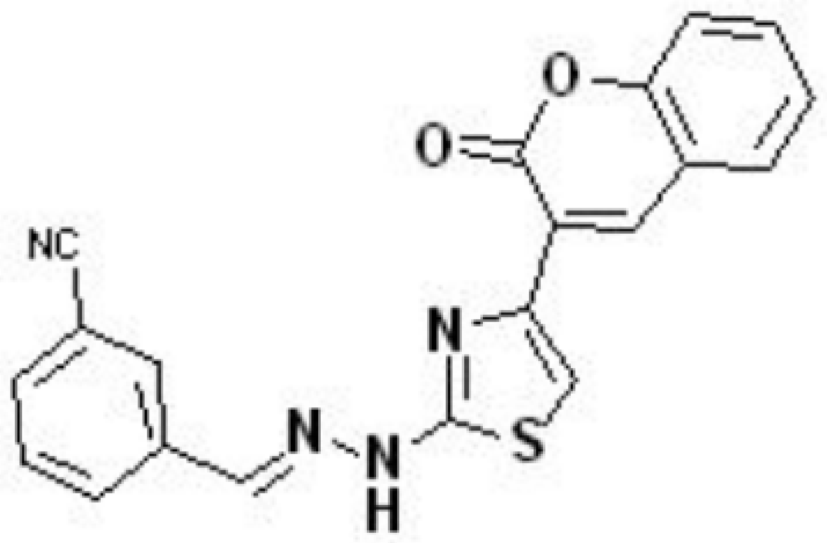

5c

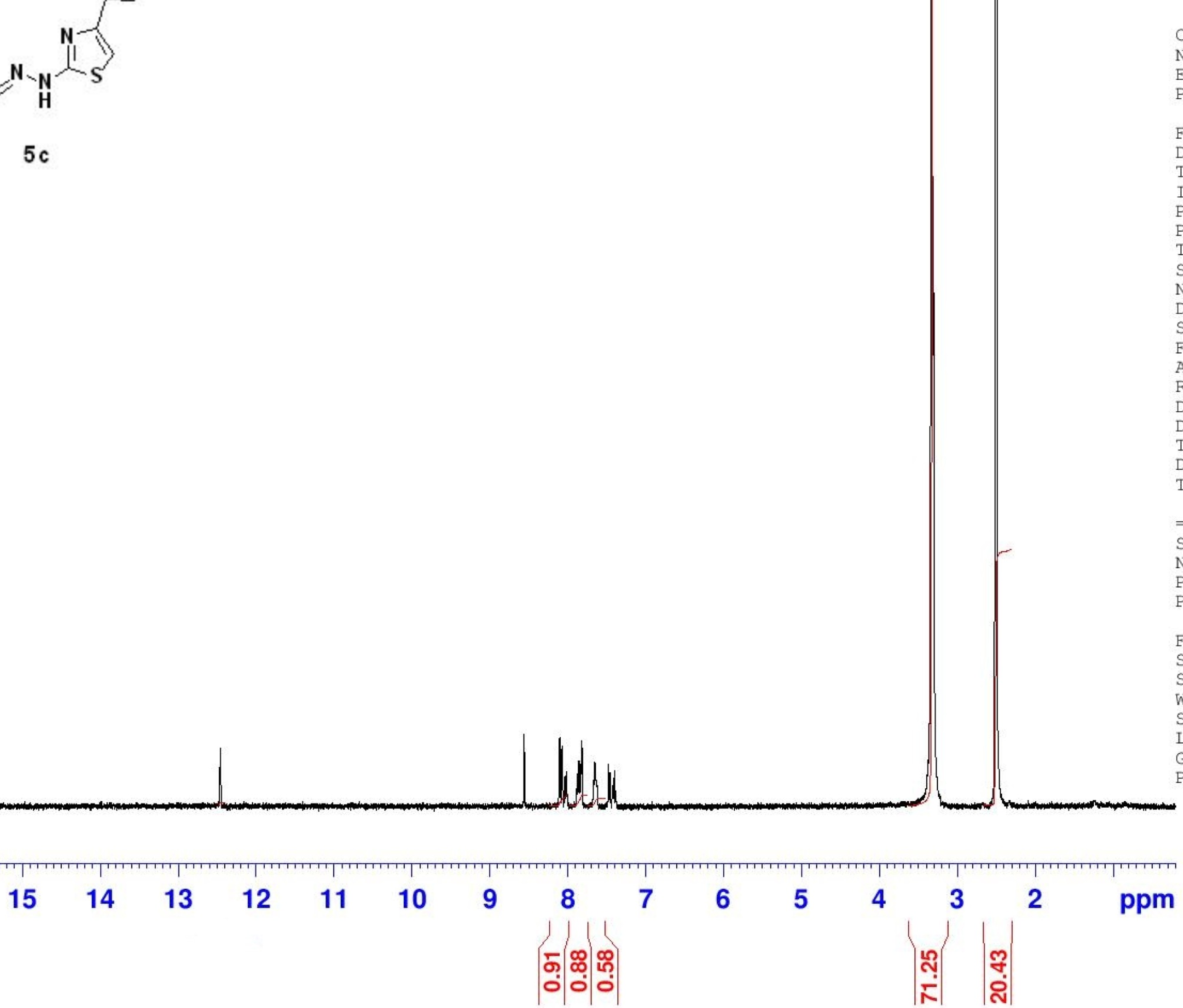


```
Current Data Parameters
NAME                 MG58
EXPNO                   1
PROCNO                  1

F2 - Acquisition Parameters
Date_            20130214
Time                16.08
INSTRUM             spect
PROBHD   5 mm PABBO BB-
PULPROG              zg30
TD                  65536
SOLVENT              DMSO
NS                     16
DS                      2
SWH              8012.820 Hz
FIDRES           0.122266 Hz
AQ              4.0894465 sec
RG                    203
DW                 62.400 usec
DE                   6.50 usec
TE                  298.0 K
D1             1.00000000 sec
TD0                     1

======== CHANNEL f1 ========
SFO1          400.1424710 MHz
NUC1                   1H
P1                  13.50 usec
PLW1          16.00000000 W

F2 - Processing parameters
SI                  65536
SF            400.1400000 MHz
WDW                    EM
SSB       0
LB                   0.30 Hz
GB        0
PC                   1.00
```

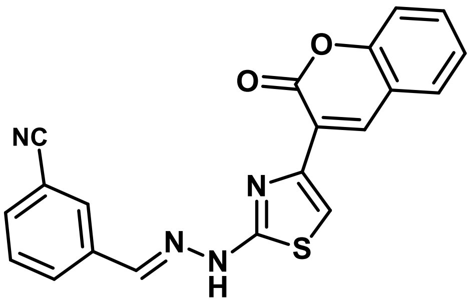

5c

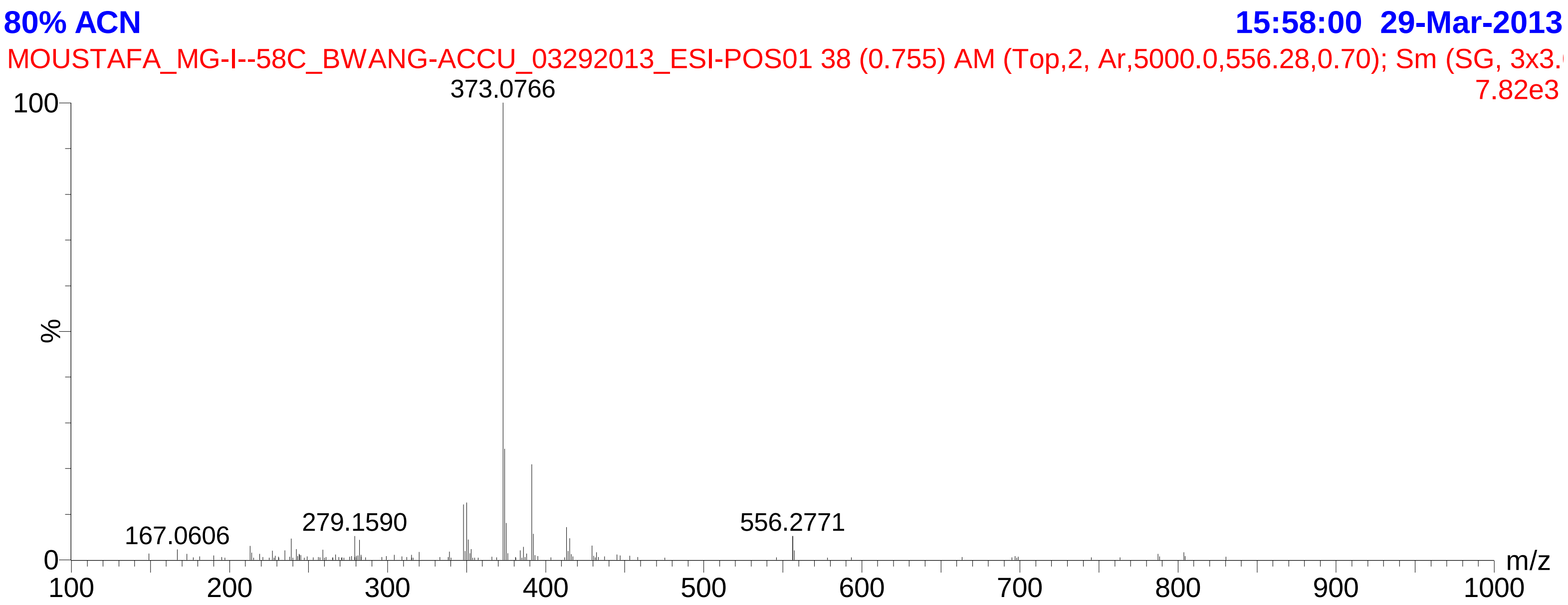

Elemental Composition Report

Single Mass Analysis

Tolerance = 5.0 PPM / DBE: min = -1.5, max = 50.0
Element prediction: Off
Number of isotope peaks used for i-FIT = 3

Monoisotopic Mass, Odd and Even Electron Ions
2006 formula(e) evaluated with 17 results within limits (all results (up to 1000) for each mass)
Elements Used:
C: 1-150 H: 1-150 N: 1-30 O: 1-60 S: 1-10

Minimum:				-1.5		
Maximum:		5.0	5.0	50.0		
Mass	Calc. Mass	mDa	PPM	DBE	i-FIT	Formula
373.0766	373.0785	-1.9	-5.1	-0.5	477.7	C H17 N12 O7 S2
	373.0753	1.3	3.5	7.5	120.1	C12 H17 N6 O4 S2
	373.0748	1.8	4.8	1.5	600.1	C12 H25 N2 O3 S4
	373.0766	0.0	0.0	7.0	110.0	C14 H19 N3 O5 S2
	373.0780	-1.4	-3.8	12.0	66.0	C15 H15 N7 O S2
	373.0759	0.7	1.9	16.5	2.6	C20 H13 N4 O2 S
	373.0773	-0.7	-1.9	16.0	6.5	C22 H15 N O3 S
	373.0751	1.5	4.0	10.0	284.2	C3 H7 N19 O2 S
	373.0751	1.5	4.0	4.5	320.4	C4 H13 N12 O7 S
	373.0760	0.6	1.6	3.5	407.8	C5 H17 N12 O2 S3
	373.0751	1.5	4.0	-1.0	370.0	C5 H19 N5 O12 S
	373.0764	0.2	0.5	9.5	229.8	C5 H9 N16 O3 S
	373.0764	0.2	0.5	4.0	264.5	C6 H15 N9 O8 S
	373.0778	-1.2	-3.2	9.0	182.6	C7 H11 N13 O4 S
	373.0773	-0.7	-1.9	3.0	385.2	C7 H19 N9 O3 S3
	373.0764	0.2	0.5	-1.5	312.2	C7 H21 N2 O13 S
	373.0778	-1.2	-3.2	3.5	215.7	C8 H17 N6 O9 S

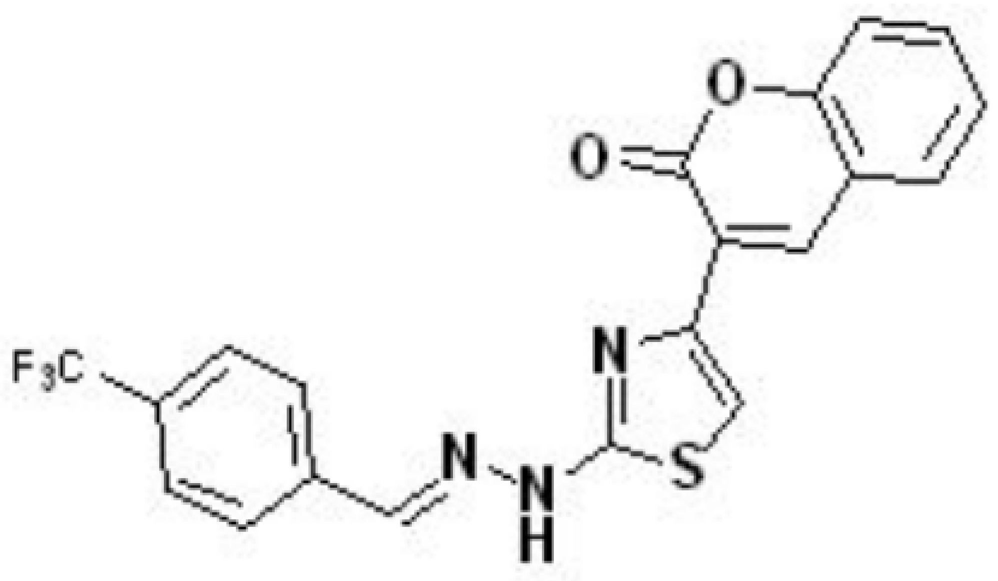

5d

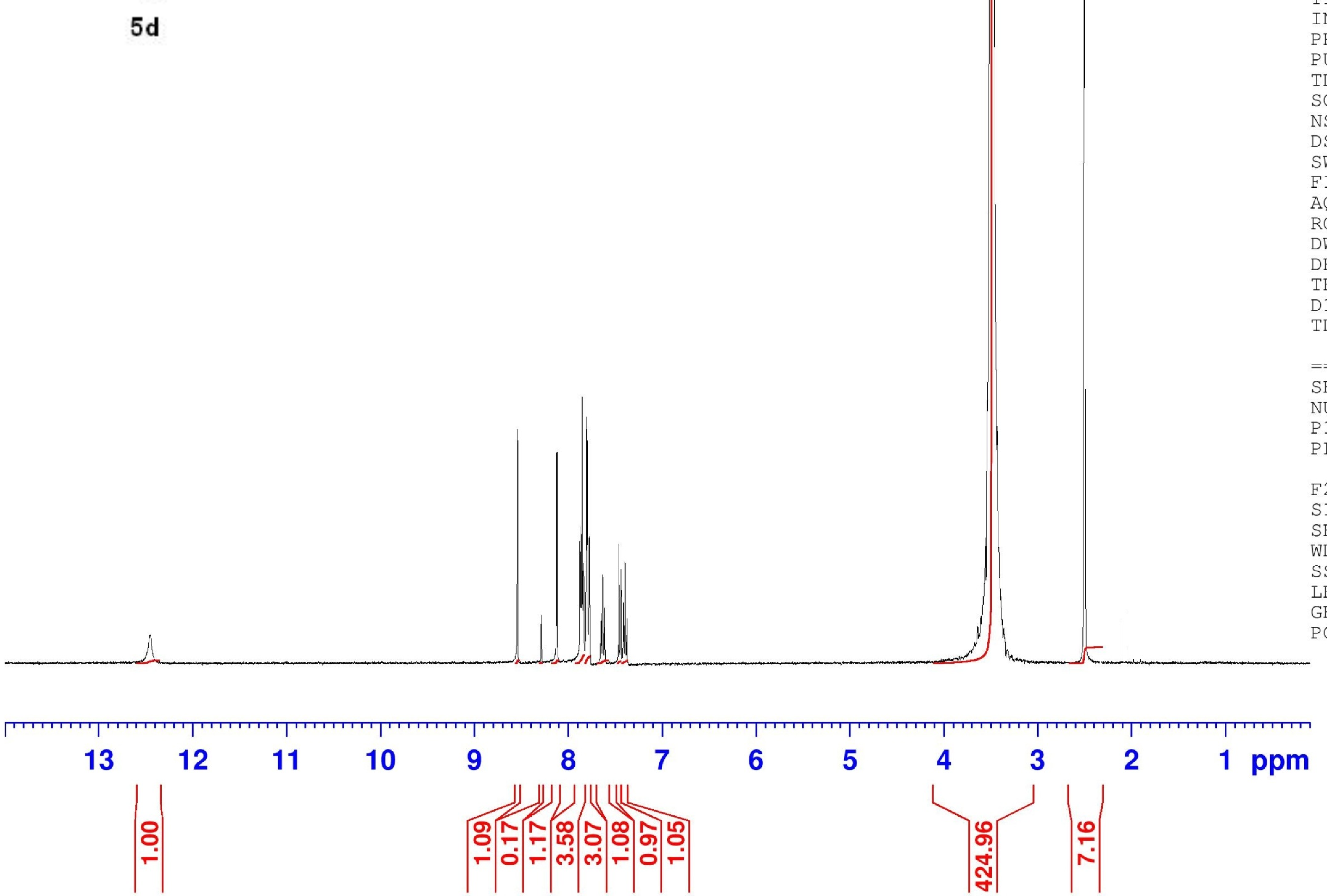

BRUKER

```
Current Data Parameters
NAME                 MG111
EXPNO                    1
PROCNO                   1

F2 - Acquisition Parameters
Date_             20130321
Time                 18.48
INSTRUM              spect
PROBHD   5 mm PABBO BB-
PULPROG               zg30
TD                   65536
SOLVENT               DMSO
NS                      16
DS                       2
SWH              8012.820 Hz
FIDRES           0.122266 Hz
AQ              4.0894465 sec
RG                      64
DW                  62.400 usec
DE                    6.50 usec
TE                   293.9 K
D1             1.00000000 sec
TD0                      1

======== CHANNEL f1 ========
SFO1          400.1424710 MHz
NUC1                    1H
P1                   13.50 usec
PLW1          16.00000000 W

F2 - Processing parameters
SI                   65536
SF            400.1400000 MHz
WDW                     EM
SSB      0
LB                    0.30 Hz
GB       0
PC                    1.40
```

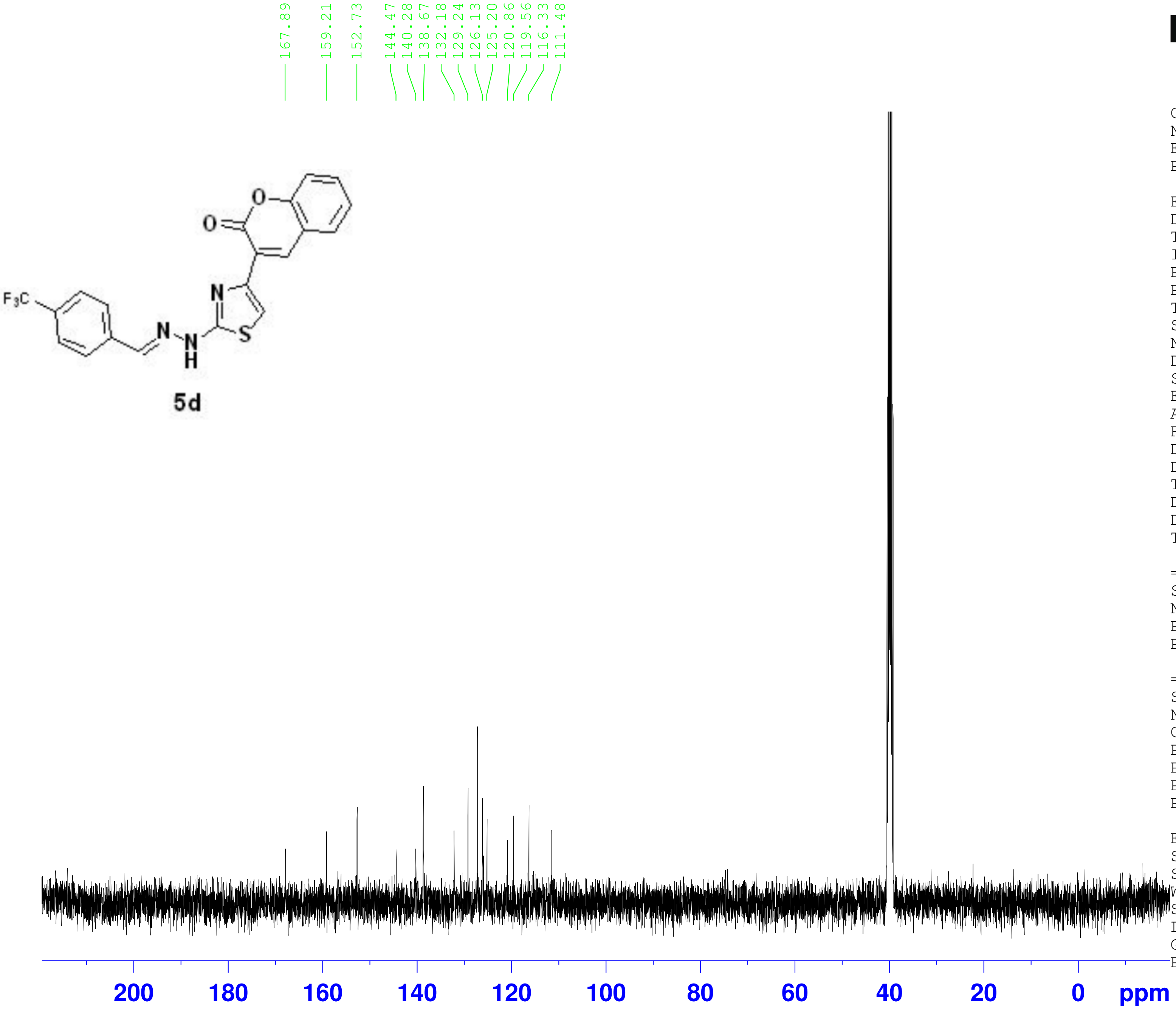

5d

Current Data Parameters
NAME MG111c
EXPNO 1
PROCNO 1

F2 - Acquisition Parameters
Date_ 20130325
Time 13.24
INSTRUM spect
PROBHD 5 mm PABBO BB-
PULPROG zgpg30
TD 65536
SOLVENT DMSO
NS 223
DS 4
SWH 24038.461 Hz
FIDRES 0.366798 Hz
AQ 1.3631488 sec
RG 203
DW 20.800 usec
DE 6.50 usec
TE 298.0 K
D1 2.00000000 sec
D11 0.03000000 sec
TD0 1

======== CHANNEL f1 ========
SFO1 100.6253441 MHz
NUC1 13C
P1 9.00 usec
PLW1 62.00000000 W

======== CHANNEL f2 ========
SFO2 400.1416006 MHz
NUC2 1H
CPDPRG[2 waltz16
PCPD2 90.00 usec
PLW2 16.00000000 W
PLW12 0.36000001 W
PLW13 0.29159999 W

F2 - Processing parameters
SI 32768
SF 100.6152830 MHz
WDW EM
SSB 0
LB 1.00 Hz
GB 0
PC 1.40

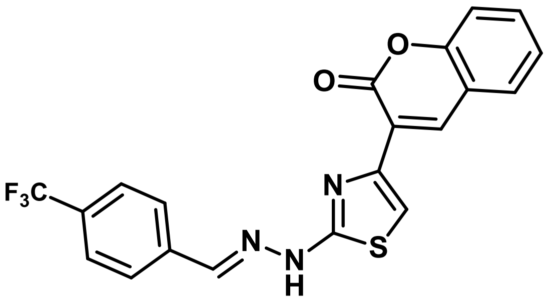

5d

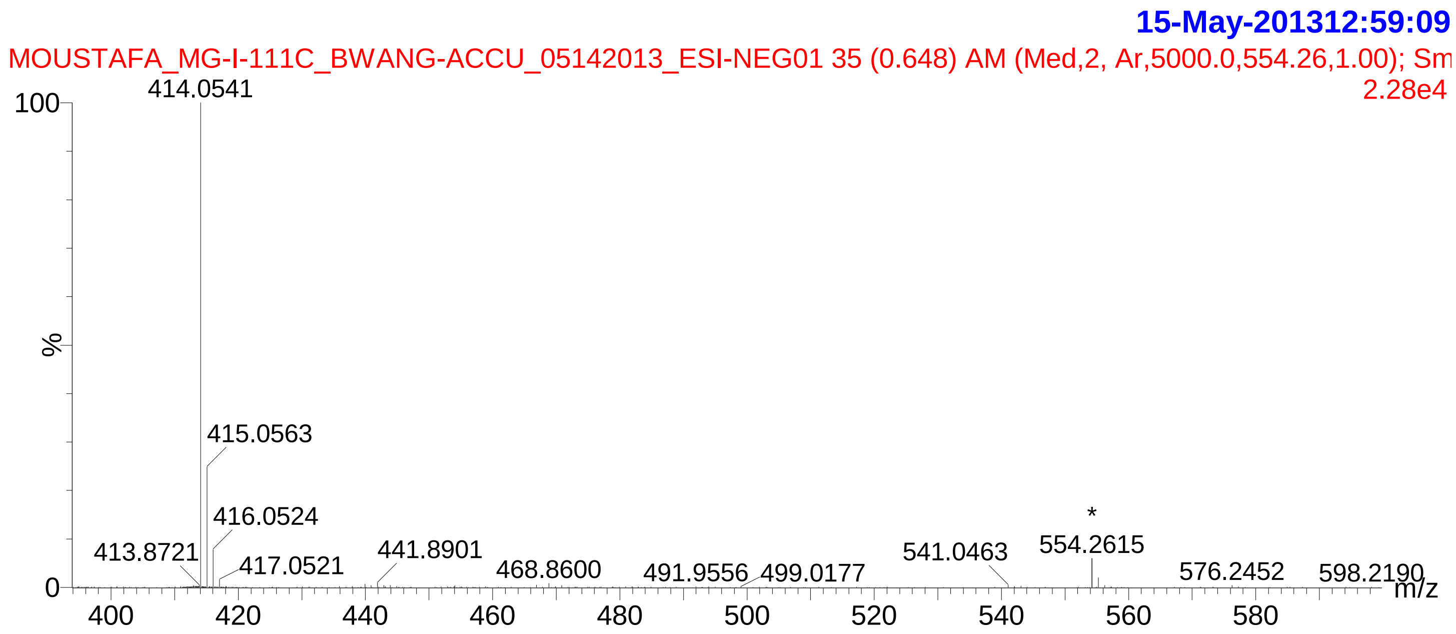

Elemental Composition Report

Single Mass Analysis

Tolerance = 5.0 PPM / DBE: min = -1.5, max = 50.0
Element prediction: Off
Number of isotope peaks used for i-FIT = 3

Monoisotopic Mass, Odd and Even Electron Ions
7294 formula(e) evaluated with 79 results within limits (all results (up to 1000) for each mass)
Elements Used:
C: 1-150 H: 1-150 N: 1-30 O: 1-60 F: 1-6 S: 1-4

Minimum:				-1.5		
Maximum:		5.0	5.0	50.0		
Mass	Calc. Mass	mDa	PPM	DBE	i-FIT	Formula
414.0541	414.0535	0.6	1.4	0.0	2094.2	C9 H23 N4 O5 F S4
	414.0551	-1.0	-2.4	1.5	1194.6	C9 H19 N5 O4 F3 S3
	414.0529	1.2	2.9	2.5	676.1	C9 H16 N5 O5 F4 S2
	414.0540	0.1	0.2	6.0	602.5	C9 H15 N8 O6 F S2
	414.0545	-0.4	-1.0	4.0	592.8	C9 H12 N6 O4 F6 S
	414.0556	-1.5	-3.6	7.5	471.4	C9 H11 N9 O5 F3 S
	414.0540	0.1	0.2	11.5	430.8	C8 H9 N15 O F S2
	414.0527	1.4	3.4	1.0	895.0	C8 H19 N4 O10 F S2
	414.0532	0.9	2.2	-1.0	851.2	C8 H16 N2 O8 F6 S
	414.0543	-0.2	-0.5	2.5	707.0	C8 H15 N5 O9 F3 S
	414.0543	-0.2	-0.5	8.0	599.7	C7 H9 N12 O4 F3 S
	414.0522	1.9	4.6	0.5	2134.6	C7 H21 N7 O4 F S4
	414.0560	-1.9	-4.6	1.0	2025.0	C7 H20 N8 O2 F2 S4
	414.0527	1.4	3.4	-1.5	1319.0	C7 H18 N5 O2 F6 S3
	414.0538	0.3	0.7	2.0	1253.3	C7 H17 N8 O3 F3 S3
	414.0554	-1.3	-3.1	3.5	711.5	C7 H13 N9 O2 F5 S2
	414.0527	1.4	3.4	6.5	682.5	C7 H13 N11 O5 F S2
	414.0532	0.9	2.2	4.5	741.6	C7 H10 N9 O3 F6 S
	414.0540	0.1	0.2	-1.5	1038.7	C6 H17 N5 O6 F5 S2
	414.0551	-1.0	-2.4	2.0	927.2	C6 H16 N8 O7 F2 S2

414.0529	1.2	2.9	3.0	860.2	C6 H13 N8 O8 F3 S
414.0529	1.2	2.9	8.5	748.8	C5 H7 N15 O3 F3 S
414.0547	-0.6	-1.4	1.5	2070.7	C5 H18 N11 O F2 S4
414.0554	-1.3	-3.1	-1.5	1148.6	C5 H16 N5 O10 F4 S
414.0524	1.7	4.1	2.5	1328.6	C5 H15 N11 O2 F3 S3
414.0540	0.1	0.2	4.0	823.3	C5 H11 N12 O F5 S2
414.0551	-1.0	-2.4	7.5	717.6	C5 H10 N15 O2 F2 S2
414.0560	-1.9	-4.6	1.5	1524.9	C4 H17 N11 O5 F S3
414.0527	1.4	3.4	-1.0	1177.5	C4 H15 N8 O5 F5 S2
414.0538	0.3	0.7	2.5	1048.0	C4 H14 N11 O6 F2 S2
414.0554	-1.3	-3.1	4.0	1007.3	C4 H10 N12 O5 F4 S
414.0538	0.3	0.7	8.0	831.9	C3 H8 N18 O F2 S2
414.0541	0.0	0.0	-1.0	1341.5	C3 H14 N8 O9 F4 S
414.0552	-1.1	-2.7	2.5	1158.2	C3 H13 N11 O10 F S
414.0546	-0.5	-1.2	14.5	229.9	C20 H14 N3 O F2 S2
414.0524	1.7	4.1	15.5	2.1	C20 H11 N3 O2 F3 S
414.0541	0.0	0.0	4.5	1195.9	C2 H8 N15 O4 F4 S
414.0552	-1.1	-2.7	8.0	1016.3	C2 H7 N18 O5 F S
414.0536	0.5	1.2	-1.5	1727.4	C2 H16 N11 O3 F4 S3
414.0547	-0.6	-1.4	2.0	1622.1	C2 H15 N14 O4 F S3
414.0525	1.6	3.9	3.0	1188.7	C2 H12 N14 O5 F2 S2
414.0552	-1.1	-2.7	0.0	1263.7	C2 H12 N12 O2 F6 S2
414.0560	-1.9	-4.6	14.5	11.5	C19 H13 N3 O5 F S
414.0542	-0.1	-0.2	9.0	896.8	C17 H19 N2 O3 F S3
414.0558	-1.7	-4.1	10.5	237.2	C17 H15 N3 O2 F3 S2
414.0536	0.5	1.2	11.5	57.7	C17 H12 N3 O3 F4 S
414.0547	-0.6	-1.4	15.0	26.6	C17 H11 N6 O4 F S
414.0533	0.8	1.9	10.0	100.2	C16 H15 N2 O8 F S
414.0533	0.8	1.9	15.5	59.7	C15 H9 N9 O3 F S
414.0528	1.3	3.1	9.5	867.8	C15 H17 N5 O2 F S3
414.0544	-0.3	-0.7	11.0	234.8	C15 H13 N6 O F3 S2

414.0522	1.9	4.6	12.0	108.6	C15 H10 N6 O2 F4 S
414.0553	-1.2	-2.9	5.0	976.8	C14 H20 N2 O4 F2 S3
414.0531	1.0	2.4	6.0	386.9	C14 H17 N2 O5 F3 S2
414.0547	-0.6	-1.4	7.5	209.8	C14 H13 N3 O4 F5 S
414.0558	-1.7	-4.1	11.0	143.2	C14 H12 N6 O5 F2 S
414.0545	-0.4	-1.0	6.0	283.0	C13 H16 N2 O9 F2 S
414.0551	-1.0	-2.4	1.0	1796.9	C12 H22 N2 O F4 S4
414.0529	1.2	2.9	2.0	996.4	C12 H19 N2 O2 F5 S3
414.0540	0.1	0.2	5.5	981.0	C12 H18 N5 O3 F2 S3
414.0556	-1.5	-3.6	7.0	372.8	C12 H14 N6 O2 F4 S2
414.0534	0.7	1.7	8.0	297.8	C12 H11 N6 O3 F5 S
414.0545	-0.4	-1.0	11.5	213.1	C12 H10 N9 O4 F2 S
414.0549	-0.8	-1.9	-0.5	2074.3	C11 H25 N O6 F S4
414.0526	1.5	3.6	0.5	1242.9	C11 H22 N O7 F2 S3
414.0542	-0.1	-0.2	2.0	597.4	C11 H18 N2 O6 F4 S2
414.0553	-1.2	-2.9	5.5	542.4	C11 H17 N5 O7 F S2
414.0531	1.0	2.4	6.5	375.7	C11 H14 N5 O8 F2 S
414.0558	-1.7	-4.1	3.5	465.8	C11 H14 N3 O5 F6 S
414.0531	1.0	2.4	12.0	303.2	C10 H8 N12 O3 F2 S
414.0540	0.1	0.2	0.5	808.0	C10 H21 N O11 F S2
414.0549	-0.8	-1.9	5.0	1799.4	C10 H19 N8 O F S4
414.0556	-1.5	-3.6	2.0	573.5	C10 H17 N2 O10 F3 S
414.0526	1.5	3.6	6.0	1000.3	C10 H16 N8 O2 F2 S3
414.0542	-0.1	-0.2	7.5	425.0	C10 H12 N9 O F4 S2
414.0553	-1.2	-2.9	11.0	376.2	C10 H11 N12 O2 F S2
414.0549	-0.8	-1.9	-1.5	1526.1	C H15 N11 O7 F3 S2
414.0527	1.4	3.4	-0.5	1554.2	C H12 N11 O8 F4 S
414.0538	0.3	0.7	3.0	1353.0	C H11 N14 O9 F S

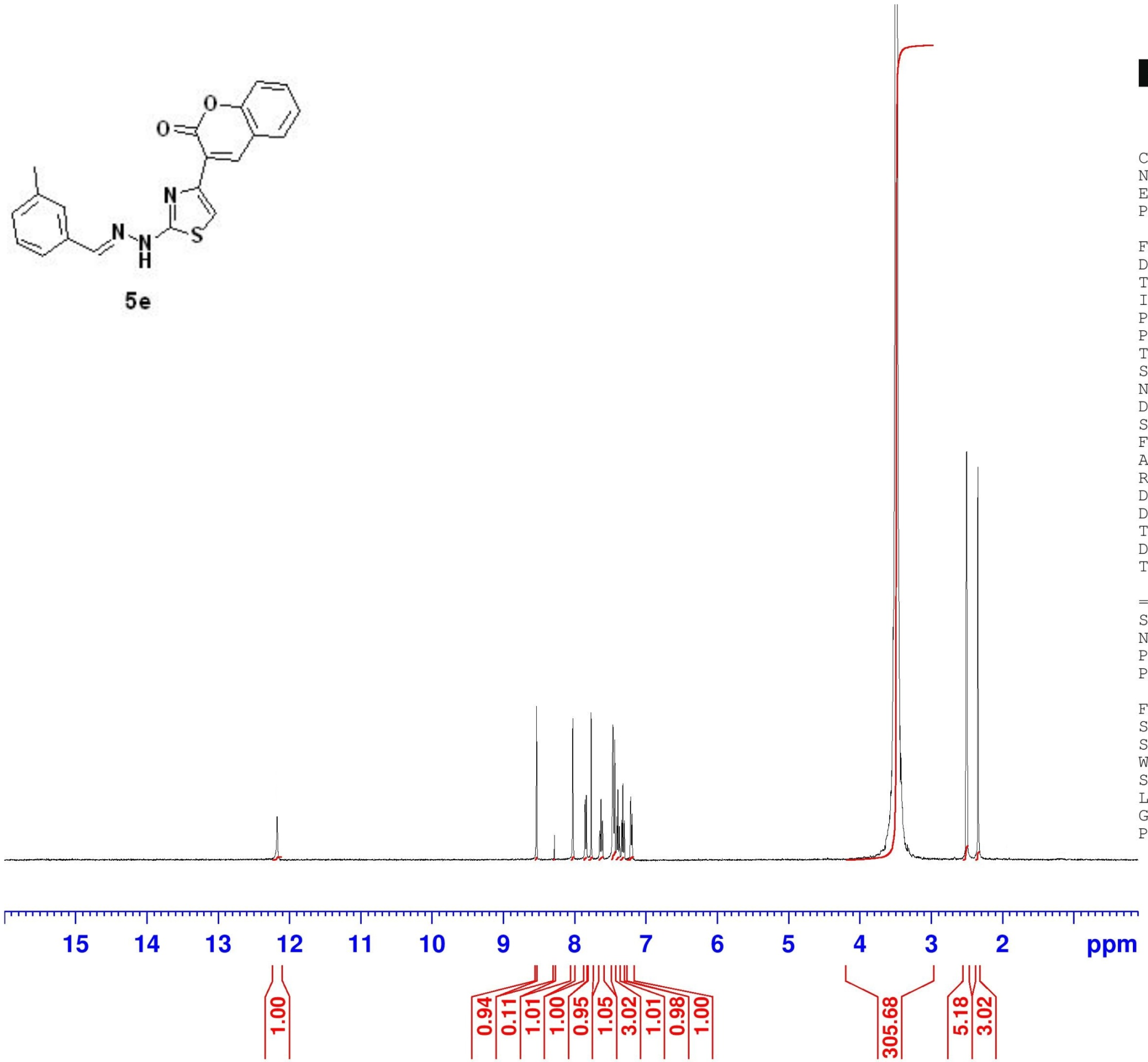

Current Data Parameters
NAME MG110p
EXPNO 1
PROCNO 1

F2 - Acquisition Parameters
Date_ 20130321
Time 18.35
INSTRUM spect
PROBHD 5 mm PABBO BB-
PULPROG zg30
TD 65536
SOLVENT DMSO
NS 16
DS 2
SWH 8012.820 Hz
FIDRES 0.122266 Hz
AQ 4.0894465 sec
RG 64
DW 62.400 usec
DE 6.50 usec
TE 293.9 K
D1 1.00000000 sec
TD0 1

======== CHANNEL f1 ========
SFO1 400.1424710 MHz
NUC1 1H
P1 13.50 usec
PLW1 16.00000000 W

F2 - Processing parameters
SI 65536
SF 400.1400000 MHz
WDW EM
SSB 0
LB 0.30 Hz
GB 0
PC 1.40

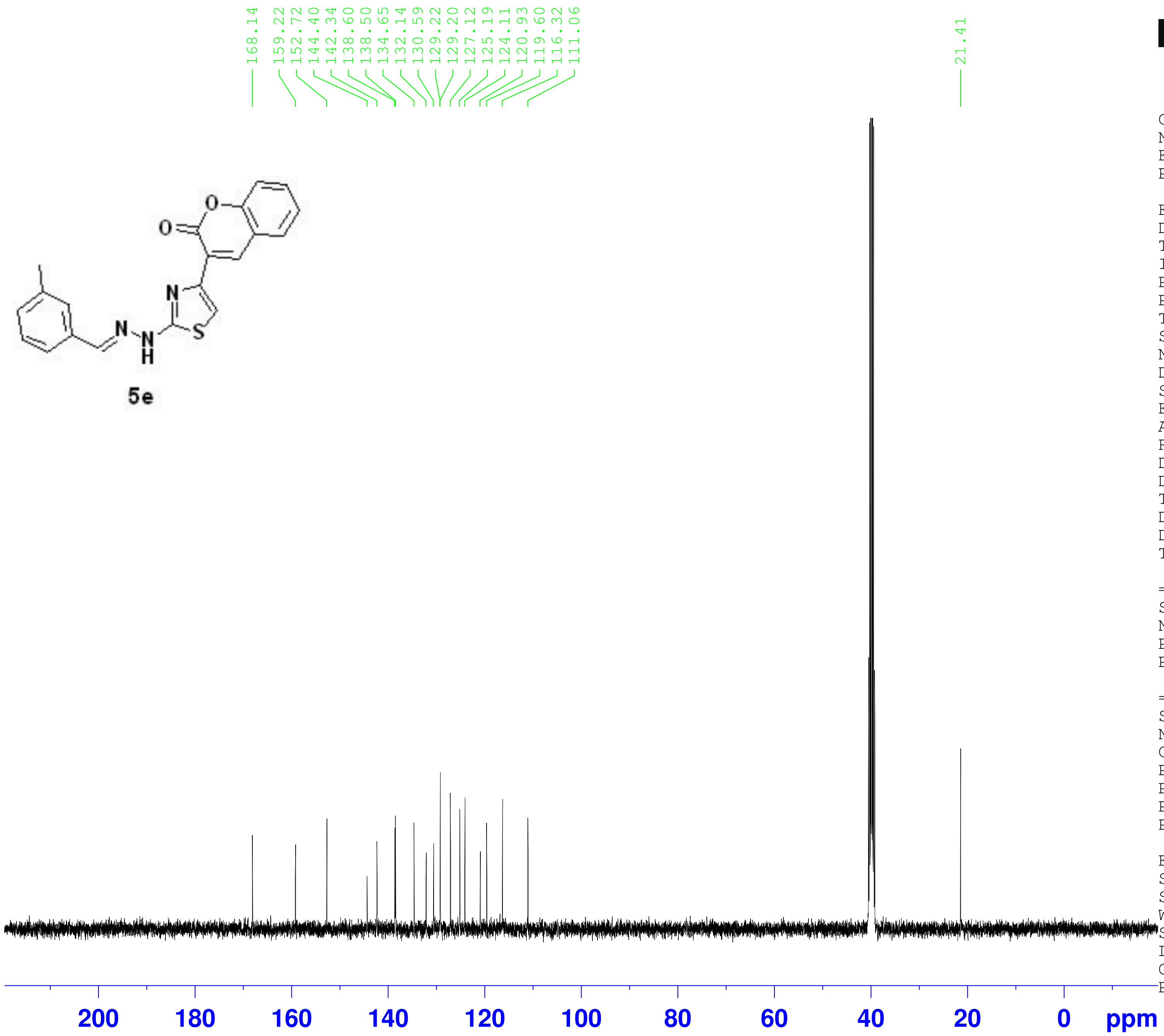

5e

```
Current Data Parameters
NAME                MG110c
EXPNO                    1
PROCNO                   1

F2 - Acquisition Parameters
Date_             20130325
Time                 13.09
INSTRUM              spect
PROBHD   5 mm PABBO BB-
PULPROG            zgpg30
TD                   65536
SOLVENT               DMSO
NS                     312
DS                       4
SWH              24038.461 Hz
FIDRES            0.366798 Hz
AQ               1.3631488 sec
RG                    90.5
DW                  20.800 usec
DE                    6.50 usec
TE                   298.0 K
D1              2.00000000 sec
D11             0.03000000 sec
TD0                      1

======== CHANNEL f1 ========
SFO1           100.6253441 MHz
NUC1                   13C
P1                    9.00 usec
PLW1           62.00000000 W

======== CHANNEL f2 ========
SFO2           400.1416006 MHz
NUC2                    1H
CPDPRG[2           waltz16
PCPD2                90.00 usec
PLW2           16.00000000 W
PLW12           0.36000001 W
PLW13           0.29159999 W

F2 - Processing parameters
SI                   32768
SF             100.6152830 MHz
WDW                     EM
SSB      0
LB                    1.00 Hz
GB       0
PC                    1.40
```

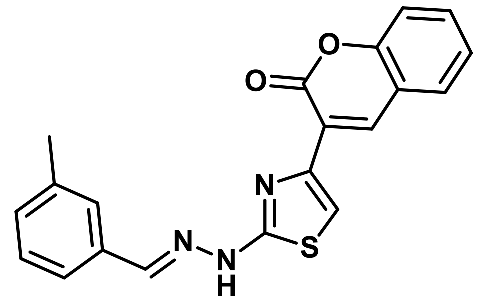

5e

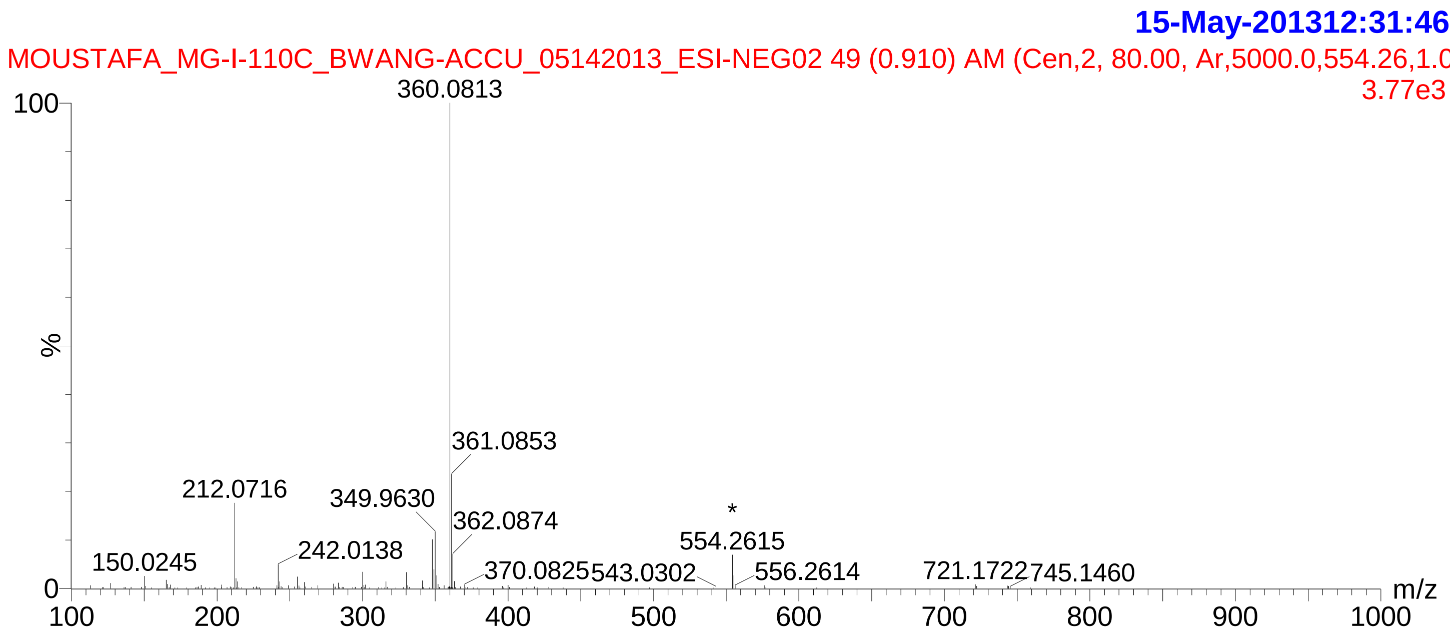

Elemental Composition Report

Single Mass Analysis

Tolerance = 5.0 PPM / DBE: min = -1.5, max = 50.0
Element prediction: Off
Number of isotope peaks used for i-FIT = 3

Monoisotopic Mass, Odd and Even Electron Ions
1424 formula(e) evaluated with 13 results within limits (all results (up to 1000) for each mass)
Elements Used:
C: 1-150 H: 1-150 N: 1-30 O: 1-60 S: 1-4

Minimum:				-1.5		
Maximum:		5.0	5.0	50.0		
Mass	Calc. Mass	mDa	PPM	DBE	i-FIT	Formula
360.0813	360.0800	1.3	3.6	6.5	74.3	C12 H18 N5 O4 S2
	360.0796	1.7	4.7	0.5	351.2	C12 H26 N O3 S4
	360.0814	-0.1	-0.3	6.0	70.9	C14 H20 N2 O5 S2
	360.0827	-1.4	-3.9	11.0	47.4	C15 H16 N6 O S2
	360.0807	0.6	1.7	15.5	1.6	C20 H14 N3 O2 S
	360.0798	1.5	4.2	9.0	127.4	C3 H8 N18 O2 S
	360.0798	1.5	4.2	3.5	147.6	C4 H14 N11 O7 S
	360.0812	0.1	0.3	8.5	101.5	C5 H10 N15 O3 S
	360.0807	0.6	1.7	2.5	232.5	C5 H18 N11 O2 S3
	360.0812	0.1	0.3	3.0	121.1	C6 H16 N8 O8 S
	360.0825	-1.2	-3.3	8.0	79.3	C7 H12 N12 O4 S
	360.0821	-0.8	-2.2	2.0	222.7	C7 H20 N8 O3 S3
	360.0825	-1.2	-3.3	2.5	98.1	C8 H18 N5 O9 S

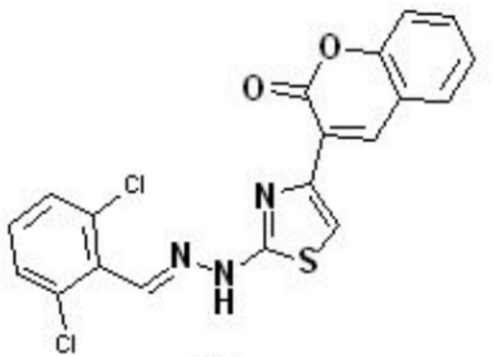

5f

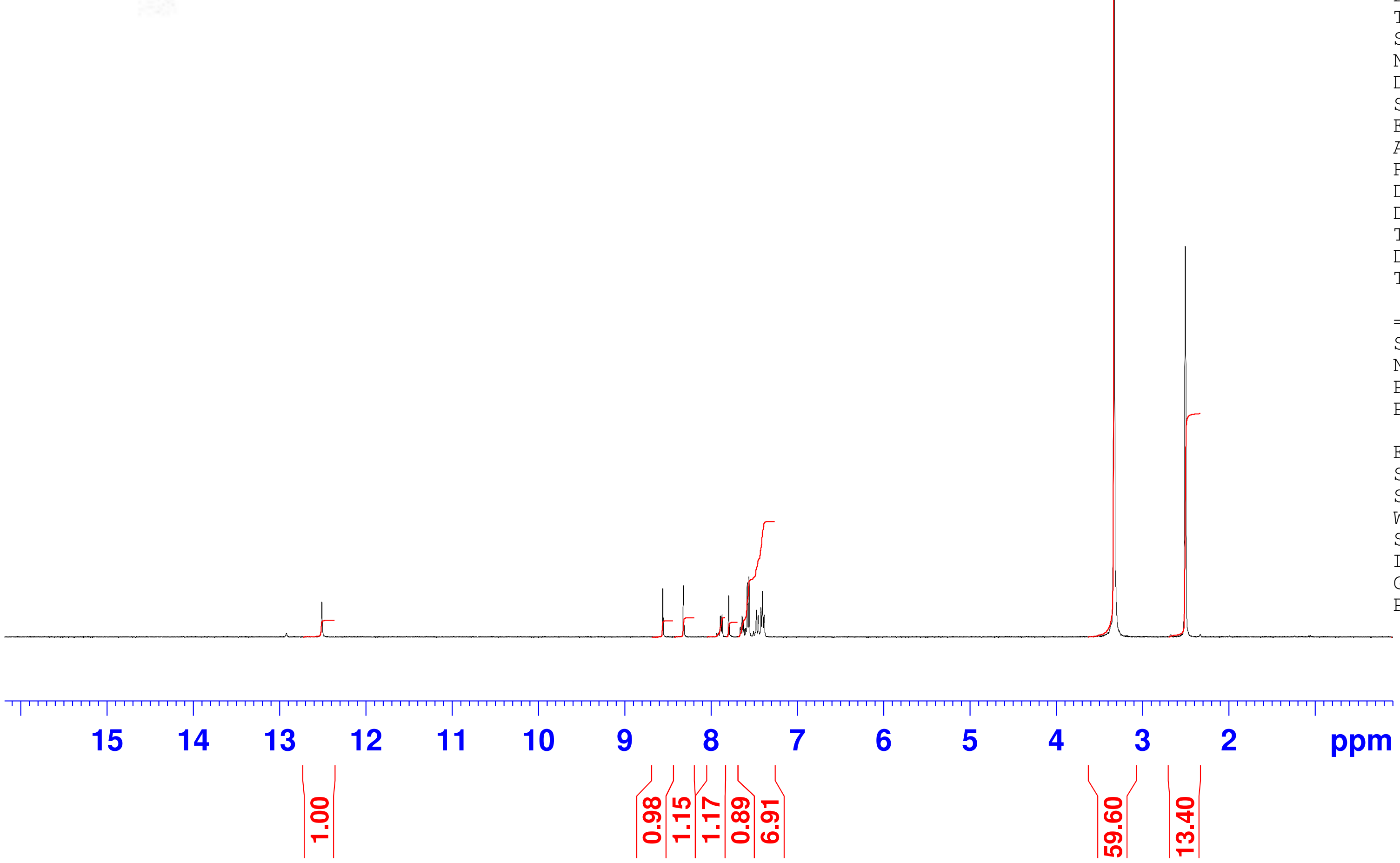

BRUKER

Current Data Parameters
NAME MG77
EXPNO 1
PROCNO 1

F2 - Acquisition Parameters
Date_ 20130227
Time 18.47
INSTRUM spect
PROBHD 5 mm PABBO BB-
PULPROG zg30
TD 65536
SOLVENT DMSO
NS 16
DS 2
SWH 8012.820 Hz
FIDRES 0.122266 Hz
AQ 4.0894465 sec
RG 203
DW 62.400 usec
DE 6.50 usec
TE 298.0 K
D1 1.00000000 sec
TD0 1

======== CHANNEL f1 ========
SFO1 400.1424710 MHz
NUC1 1H
P1 13.50 usec
PLW1 16.00000000 W

F2 - Processing parameters
SI 65536
SF 400.1400000 MHz
WDW EM
SSB 0
LB 0.30 Hz
GB 0
PC 1.40

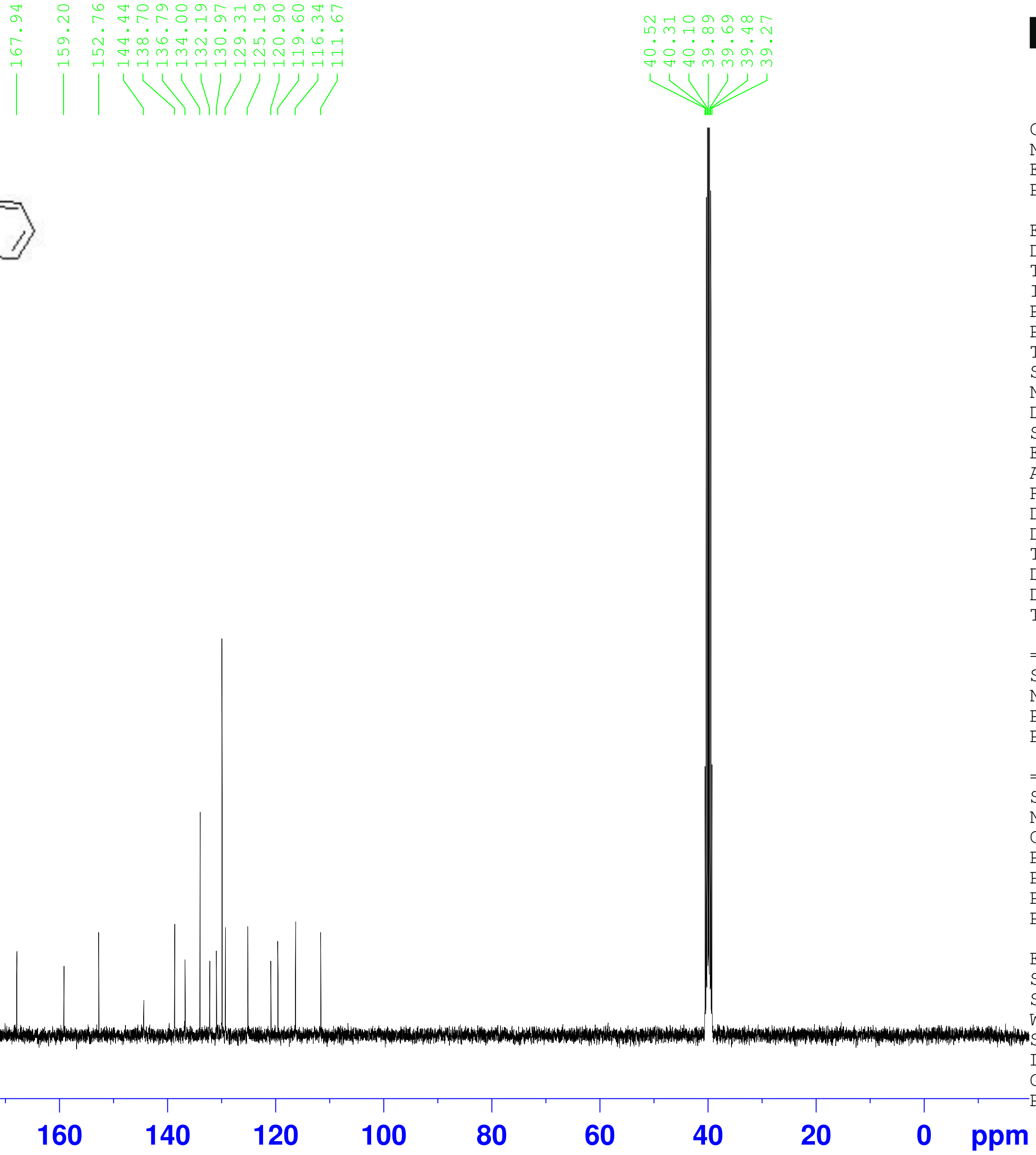

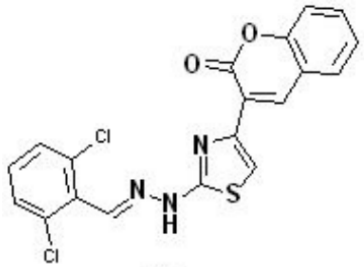

5f

BRUKER

Current Data Parameters
NAME MG94c
EXPNO 1
PROCNO 1

F2 - Acquisition Parameters
Date_ 20130314
Time 14.25
INSTRUM spect
PROBHD 5 mm PABBO BB-
PULPROG zgpg30
TD 65536
SOLVENT DMSO
NS 272
DS 4
SWH 24038.461 Hz
FIDRES 0.366798 Hz
AQ 1.3631488 sec
RG 161
DW 20.800 usec
DE 6.50 usec
TE 293.8 K
D1 2.00000000 sec
D11 0.03000000 sec
TD0 1

======== CHANNEL f1 ========
SFO1 100.6253441 MHz
NUC1 13C
P1 9.00 usec
PLW1 62.00000000 W

======== CHANNEL f2 ========
SFO2 400.1416006 MHz
NUC2 1H
CPDPRG[2 waltz16
PCPD2 90.00 usec
PLW2 16.00000000 W
PLW12 0.36000001 W
PLW13 0.29159999 W

F2 - Processing parameters
SI 32768
SF 100.6152830 MHz
WDW EM
SSB 0
LB 1.00 Hz
GB 0
PC 1.40

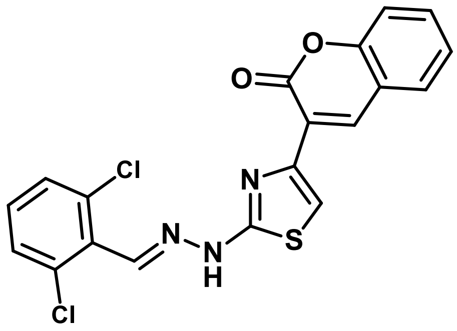
O
O
N
S
N
N
H
Cl
Cl

5f

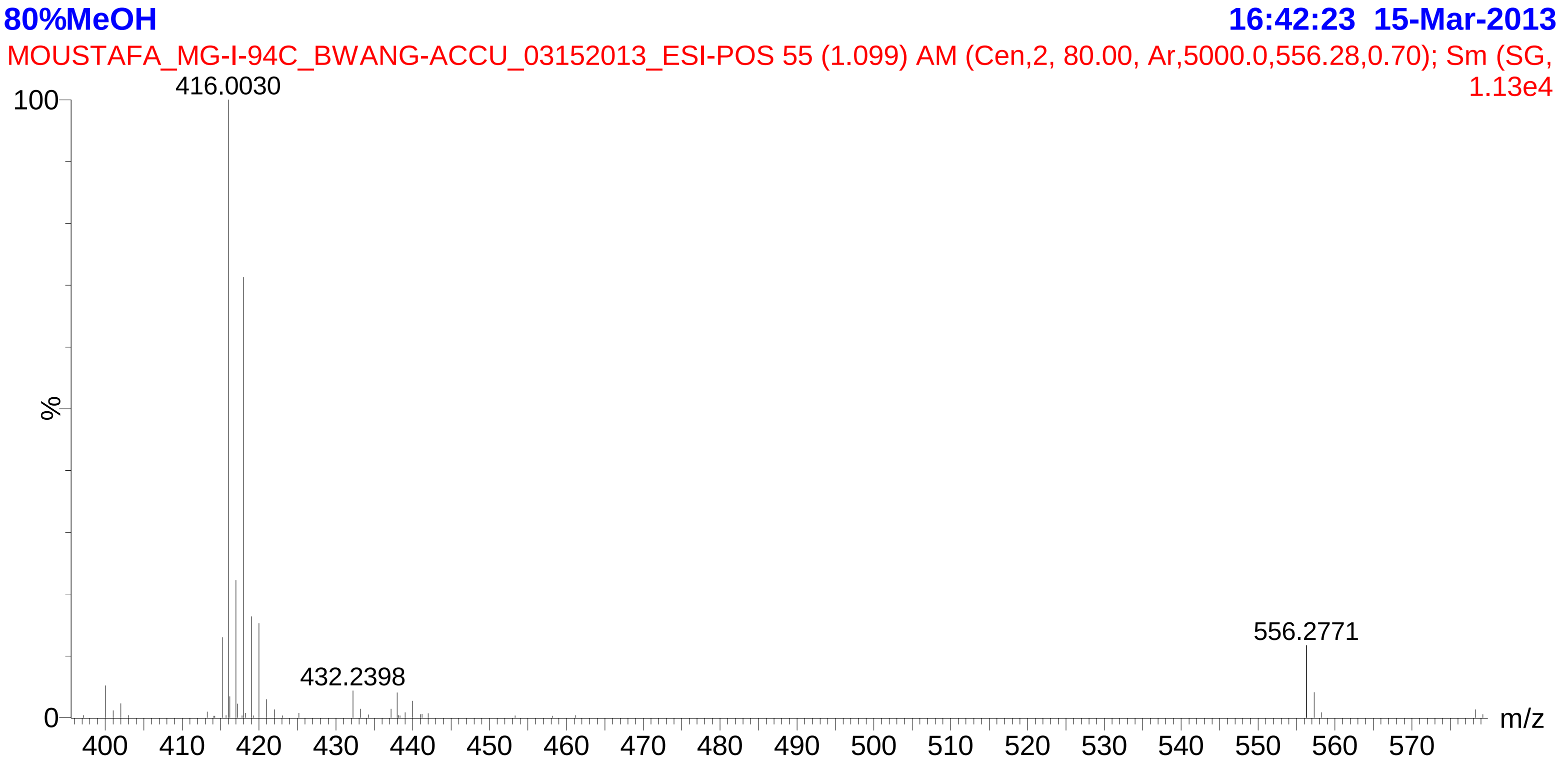
80%MeOH
16:42:23 15-Mar-2013
MOUSTAFA_MG-I-94C_BWANG-ACCU_03152013_ESI-POS 55 (1.099) AM (Cen,2, 80.00, Ar,5000.0,556.28,0.70); Sm (SG,
1.13e4
100
%
0
416.0030
432.2398
556.2771
400
410
420
430
440
450
460
470
480
490
500
510
520
530
540
550
560
570
m/z

Elemental Composition Report

Single Mass Analysis
Tolerance = 5.0 PPM / DBE: min = -1.5, max = 50.0
Element prediction: Off
Number of isotope peaks used for i-FIT = 3

Monoisotopic Mass, Odd and Even Electron Ions
3532 formula(e) evaluated with 40 results within limits (up to 100 closest results for each mass)
Elements Used:
C: 1-150 H: 1-150 N: 1-30 O: 1-60 S: 1-10 Cl: 1-2

Minimum:				-1.5			
Maximum:		5.0	5.0	50.0			
Mass	Calc. Mass		mDa	PPM	DBE	i-FIT	Formula
416.0030	416.0034	-0.4	-1.0	2.5	957.0	C H11 N13 O7 S2 Cl	
	416.0041	-1.1	-2.6	6.0	755.8	C10 H13 N4 O10 S Cl	
	416.0021	0.9	2.2	5.5	108.0	C11 H16 N5 O4 S2 Cl2	
	416.0050	-2.0	-4.8	5.0	370.8	C11 H17 N4 O5 S3 Cl	
	416.0016	1.4	3.4	-0.5	184.1	C11 H24 N O3 S4 Cl2	
	416.0034	-0.4	-1.0	5.0	76.3	C13 H18 N2 O5 S2 Cl2	
	416.0016	1.4	3.4	10.0	481.1	C14 H13 N4 O5 S2 Cl	
	416.0048	-1.8	-4.3	10.0	28.7	C14 H14 N6 O S2 Cl2	
	416.0029	0.1	0.2	15.0	501.2	C15 H9 N8 O S2 Cl	
	416.0029	0.1	0.2	9.5	463.6	C16 H15 N O6 S2 Cl	
	416.0014	1.6	3.8	15.0	1.1	C17 H10 N6 O S Cl2	
	416.0043	-1.3	-3.1	14.5	498.8	C17 H11 N5 O2 S2 Cl	
	416.0038	-0.8	-1.9	8.5	220.5	C17 H19 N O S4 Cl	
	416.0027	0.3	0.7	14.5	2.4	C19 H12 N3 O2 S Cl2	
	416.0014	1.6	3.8	2.0	417.1	C2 H14 N14 O S3 Cl2	
	416.0043	-1.3	-3.1	1.5	526.7	C2 H15 N13 O2 S4 Cl	
	416.0019	1.1	2.6	8.0	379.7	C2 H6 N18 O2 S Cl2	

416.0048	-1.8	-4.3	7.5	818.7	C2 H7 N17 O3 S2 Cl
416.0022	0.8	1.9	19.0	736.6	C22 H9 N2 O3 S Cl
416.0019	1.1	2.6	2.5	459.5	C3 H12 N11 O7 S Cl2
416.0048	-1.8	-4.3	2.0	859.2	C3 H13 N10 O8 S2 Cl
416.0028	0.2	0.5	1.5	348.1	C4 H16 N11 O2 S3 Cl2
416.0032	-0.2	-0.5	7.5	307.4	C4 H8 N15 O3 S Cl2
416.0009	2.1	5.0	6.5	517.5	C5 H11 N13 O2 S3 Cl
416.0032	-0.2	-0.5	2.0	380.2	C5 H14 N8 O8 S Cl2
416.0014	1.6	3.8	12.5	877.5	C5 H3 N17 O3 S Cl
416.0046	-1.6	-3.8	7.0	244.6	C6 H10 N12 O4 S Cl2
416.0009	2.1	5.0	1.0	544.9	C6 H17 N6 O7 S3 Cl
416.0041	-1.1	-2.6	1.0	288.2	C6 H18 N8 O3 S3 Cl2
416.0014	1.6	3.8	7.0	890.9	C6 H9 N10 O8 S Cl
416.0023	0.7	1.7	6.0	458.4	C7 H13 N10 O3 S3 Cl
416.0014	1.6	3.8	1.5	914.7	C7 H15 N3 O13 S Cl
416.0046	-1.6	-3.8	1.5	310.1	C7 H16 N5 O9 S Cl2
416.0018	1.2	2.9	0.0	244.7	C7 H21 N6 O2 S5 Cl
416.0027	0.3	0.7	12.0	812.7	C7 H5 N14 O4 S Cl
416.0027	0.3	0.7	6.5	817.8	C8 H11 N7 O9 S Cl
416.0023	0.7	1.7	0.5	477.5	C8 H19 N3 O8 S3 Cl
416.0036	-0.6	-1.4	5.5	409.6	C9 H15 N7 O4 S3 Cl
416.0032	-0.2	-0.5	-0.5	199.7	C9 H23 N3 O3 S5 Cl
416.0041	-1.1	-2.6	11.5	759.2	C9 H7 N11 O5 S Cl

test

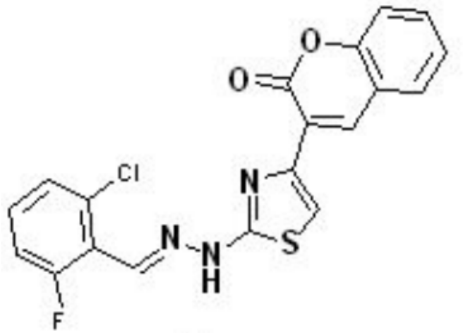

5g

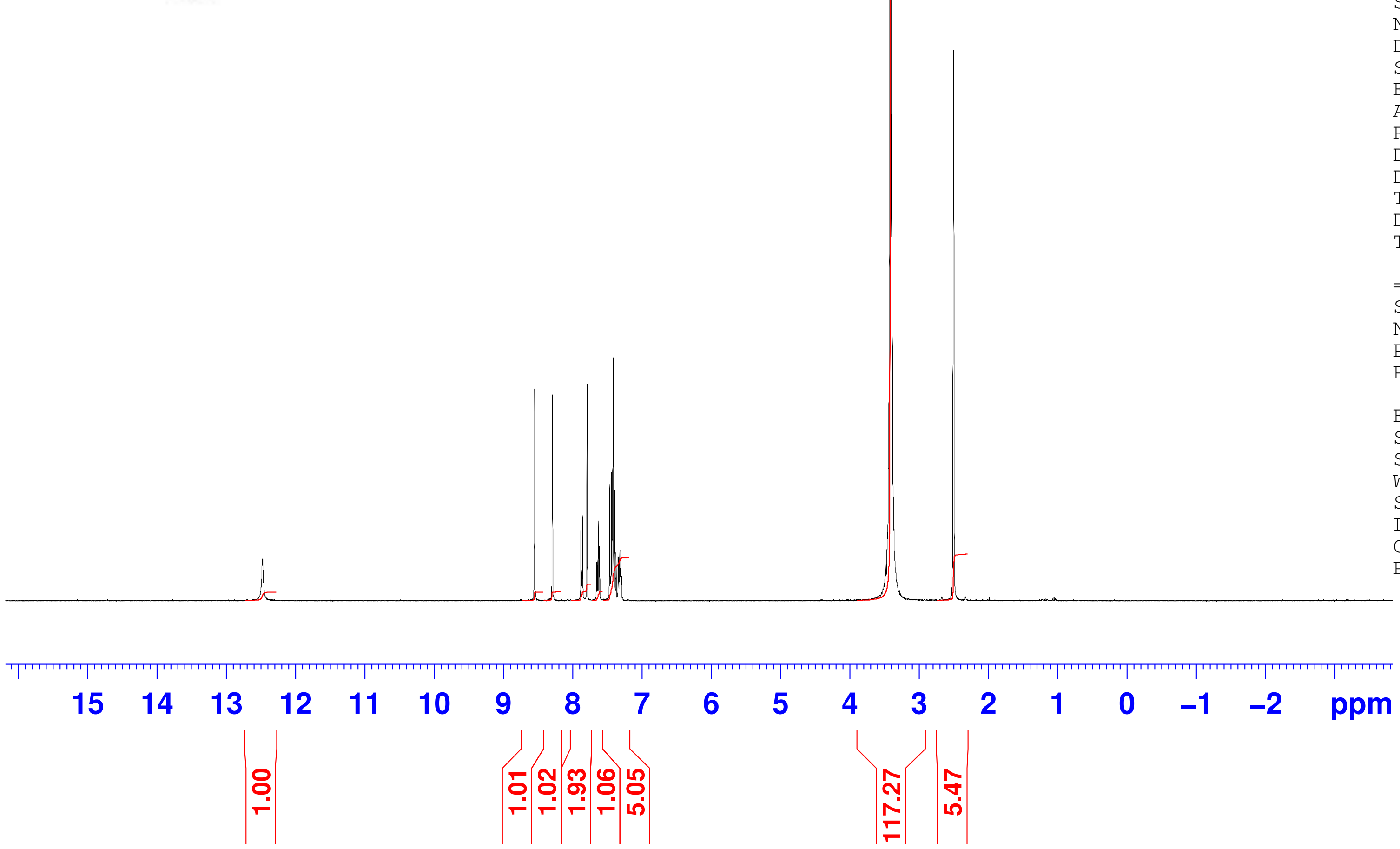

Current Data Parameters
NAME MG93
EXPNO 1
PROCNO 1

F2 - Acquisition Parameters
Date_ 20130313
Time 18.12
INSTRUM spect
PROBHD 5 mm PABBO BB-
PULPROG zg30
TD 65536
SOLVENT DMSO
NS 16
DS 2
SWH 8012.820 Hz
FIDRES 0.122266 Hz
AQ 4.0894465 sec
RG 101
DW 62.400 usec
DE 6.50 usec
TE 293.6 K
D1 1.00000000 sec
TD0 1

======== CHANNEL f1 ========
SFO1 400.1424710 MHz
NUC1 1H
P1 13.50 usec
PLW1 16.00000000 W

F2 - Processing parameters
SI 65536
SF 400.1400000 MHz
WDW EM
SSB 0
LB 0.30 Hz
GB 0
PC 1.00

test

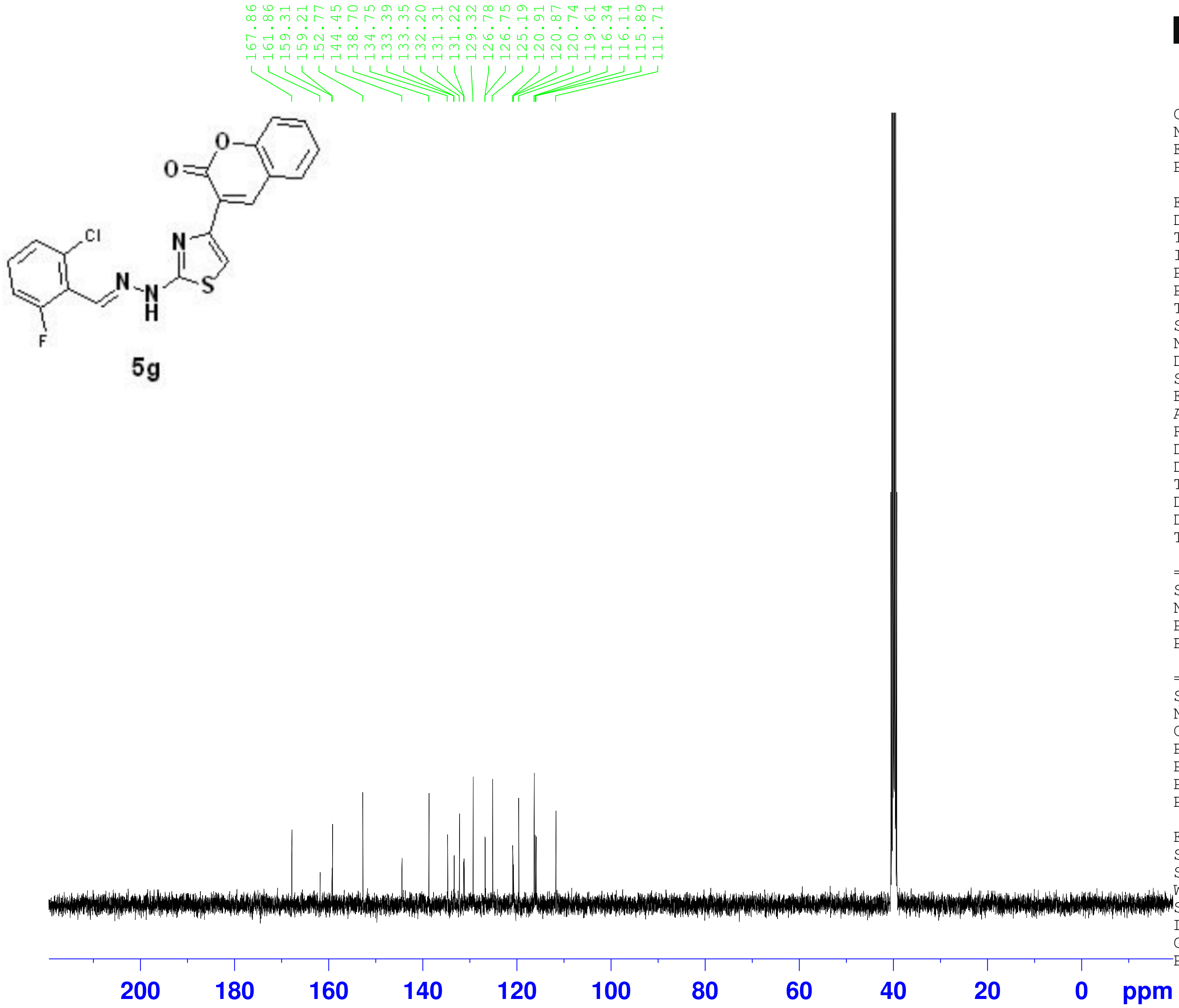

Current Data Parameters

NAME	MG93c
EXPNO	1
PROCNO	1

F2 - Acquisition Parameters

Date_	20130315
Time	12.55
INSTRUM	spect
PROBHD	5 mm PABBO BB-
PULPROG	zgpg30
TD	65536
SOLVENT	DMSO
NS	334
DS	4
SWH	24038.461 Hz
FIDRES	0.366798 Hz
AQ	1.3631488 sec
RG	181
DW	20.800 usec
DE	6.50 usec
TE	295.6 K
D1	2.00000000 sec
D11	0.03000000 sec
TD0	1

======== CHANNEL f1 ========

SFO1	100.6253441 MHz
NUC1	13C
P1	9.00 usec
PLW1	62.00000000 W

======== CHANNEL f2 ========

SFO2	400.1416006 MHz
NUC2	1H
CPDPRG[2	waltz16
PCPD2	90.00 usec
PLW2	16.00000000 W
PLW12	0.36000001 W
PLW13	0.29159999 W

F2 - Processing parameters

SI	32768
SF	100.6152830 MHz
WDW	EM
SSB	0
LB	1.00 Hz
GB	0
PC	1.40

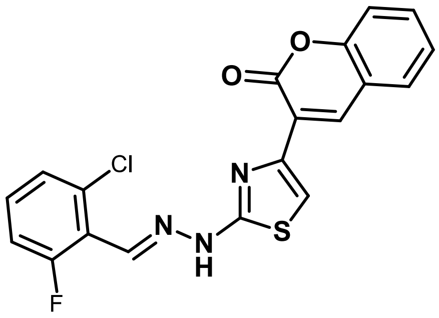

O
O
N
S
Cl
N
N
H
F

5g

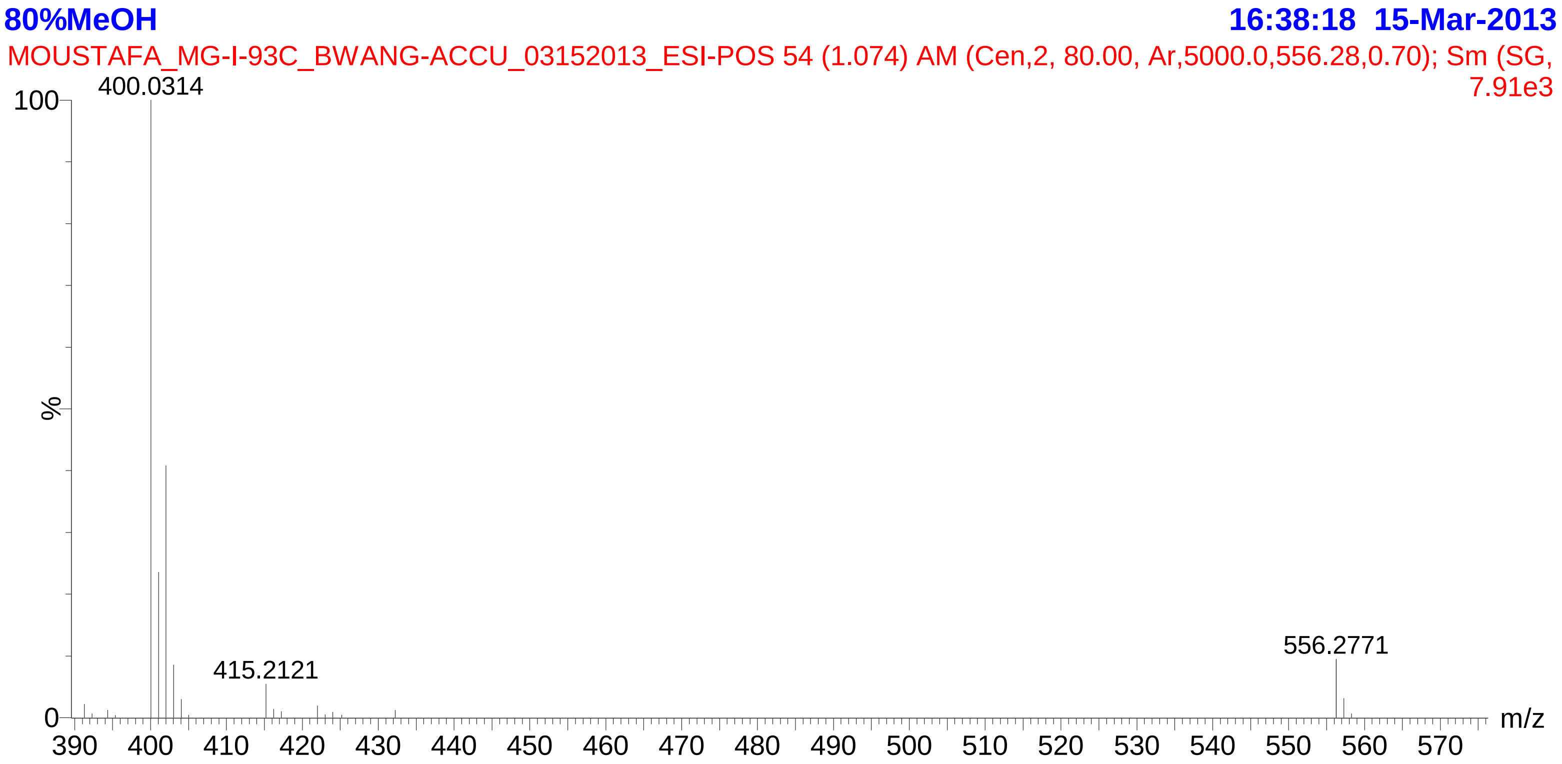

80%MeOH
16:38:18 15-Mar-2013
MOUSTAFA_MG-I-93C_BWANG-ACCU_03152013_ESI-POS 54 (1.074) AM (Cen,2, 80.00, Ar,5000.0,556.28,0.70); Sm (SG,
7.91e3
400.0314
100
%
0
415.2121
556.2771
m/z
390
400
410
420
430
440
450
460
470
480
490
500
510
520
530
540
550
560
570

Elemental Composition Report

Single Mass Analysis
Tolerance = 5.0 PPM / DBE: min = -1.5, max = 50.0
Element prediction: Off
Number of isotope peaks used for i-FIT = 3

Monoisotopic Mass, Odd and Even Electron Ions
8125 formula(e) evaluated with 61 results within limits (up to 100 closest results for each mass)
Elements Used:
C: 1-150 H: 1-150 N: 1-30 O: 1-60 F: 1-12 S: 1-10 Cl: 1-2

Minimum:				-1.5			
Maximum:		5.0	5.0	50.0			
Mass	Calc. Mass		mDa	PPM	DBE	i-FIT	Formula
400.0314	400.0328	-1.4	-3.5	0.0	476.4	C H10 N12 O3 F5 S Cl	
	400.0301	1.3	3.2	3.0	427.8	C H10 N14 O6 F S Cl	
	400.0333	-1.9	-4.7	3.0	852.5	C H11 N16 O2 F S Cl2	
	400.0322	-0.8	-2.0	-0.5	899.0	C H12 N13 O F4 S Cl2	
	400.0312	0.2	0.5	-1.5	450.8	C H14 N11 O4 F3 S2 Cl	
	400.0319	-0.5	-1.2	2.0	167.8	C10 H16 N2 O7 F3 S Cl	
	400.0312	0.2	0.5	1.5	659.3	C10 H18 N3 O5 F2 S Cl2	
	400.0303	1.1	2.7	0.5	166.7	C10 H20 N O8 F S2 Cl	
	400.0332	-1.8	-4.5	7.0	98.1	C11 H12 N6 O3 F3 S Cl	
	400.0321	-0.7	-1.7	3.5	129.1	C11 H13 N3 O2 F6 S Cl	
	400.0326	-1.2	-3.0	6.5	573.1	C11 H14 N7 O F2 S Cl2	
	400.0316	-0.2	-0.5	5.5	95.5	C11 H16 N5 O4 F S2 Cl	
	400.0305	0.9	2.2	2.0	123.7	C11 H17 N2 O3 F4 S2 Cl	
	400.0299	1.5	3.7	1.5	698.4	C11 H19 N3 O F3 S2 Cl2	
	400.0328	-1.4	-3.5	1.0	136.6	C11 H20 N2 O2 F3 S3 Cl	
	400.0312	0.2	0.5	-0.5	184.2	C11 H24 N O3 F S4 Cl	
	400.0307	0.7	1.7	11.5	53.0	C12 H9 N9 O F2 S Cl	

400.0307	0.7	1.7	6.0	75.5	C13 H15 N2 O6 F2 S Cl
400.0328	-1.4	-3.5	2.5	571.2	C13 H17 N O F5 S Cl2
400.0301	1.3	3.2	5.5	565.7	C13 H17 N3 O4 F S Cl2
400.0330	-1.6	-4.0	5.0	71.1	C13 H18 N2 O5 F S2 Cl
400.0321	-0.7	-1.7	11.0	33.2	C14 H11 N6 O2 F2 S Cl
400.0310	0.4	1.0	7.5	51.7	C14 H12 N3 O F5 S Cl
400.0316	-0.2	-0.5	5.0	70.2	C14 H19 N2 O F2 S3 Cl
400.0296	1.8	4.5	10.0	22.0	C16 H14 N2 O5 F S Cl
400.0328	-1.4	-3.5	10.0	483.4	C16 H15 N4 O F S Cl2
400.0309	0.5	1.2	15.0	4.6	C17 H10 N6 O F S Cl
400.0323	-0.9	-2.2	14.5	1.5	C19 H12 N3 O2 F S Cl
400.0310	0.4	1.0	2.0	333.9	C2 H14 N14 O F S3 Cl
400.0314	0.0	0.0	8.0	306.3	C2 H6 N18 O2 F S Cl
400.0303	1.1	2.7	4.5	362.9	C2 H7 N15 O F4 S Cl
400.0314	0.0	0.0	2.5	362.2	C3 H12 N11 O7 F S Cl
400.0303	1.1	2.7	-1.0	423.1	C3 H13 N8 O6 F4 S Cl
400.0297	1.7	4.2	-1.5	871.5	C3 H15 N9 O4 F3 S Cl2
400.0306	0.8	2.0	0.5	358.9	C4 H10 N9 O F7 S Cl
400.0301	1.3	3.2	2.5	286.6	C4 H13 N11 O3 F2 S2 Cl
400.0294	2.0	5.0	2.0	845.2	C4 H15 N12 O F S2 Cl2
400.0323	-0.9	-2.2	1.5	284.6	C4 H16 N11 O2 F S3 Cl
400.0328	-1.4	-3.5	7.5	252.1	C4 H8 N15 O3 F S Cl
400.0317	-0.3	-0.7	4.0	302.7	C4 H9 N12 O2 F4 S Cl
400.0328	-1.4	-3.5	2.0	303.5	C5 H14 N8 O8 F S Cl
400.0317	-0.3	-0.7	-1.5	358.5	C5 H15 N5 O7 F4 S Cl
400.0330	-1.6	-4.0	3.5	249.3	C6 H11 N9 O3 F4 S Cl
400.0319	-0.5	-1.2	0.0	299.4	C6 H12 N6 O2 F7 S Cl
400.0324	-1.0	-2.5	3.0	711.1	C6 H13 N10 O F3 S Cl2
400.0314	0.0	0.0	2.0	236.9	C6 H15 N8 O4 F2 S2 Cl
400.0303	1.1	2.7	-1.5	283.1	C6 H16 N5 O3 F5 S2 Cl
400.0308	0.6	1.5	1.5	806.1	C6 H17 N9 O2 F S2 Cl2

400.0305	0.9	2.2	8.0	170.5	C7 H8 N12 O F3 S Cl
400.0333	-1.9	-4.7	-0.5	246.7	C8 H14 N3 O3 F7 S Cl
400.0305	0.9	2.2	2.5	211.8	C8 H14 N5 O6 F3 S Cl
400.0294	2.0	5.0	-1.0	258.8	C8 H15 N2 O5 F6 S Cl
400.0326	-1.2	-3.0	-1.0	708.5	C8 H16 N4 O F6 S Cl2
400.0299	1.5	3.7	2.0	694.1	C8 H16 N6 O4 F2 S Cl2
400.0328	-1.4	-3.5	1.5	193.3	C8 H17 N5 O5 F2 S2 Cl
400.0321	-0.7	-1.7	1.0	770.9	C8 H19 N6 O3 F S2 Cl2
400.0319	-0.5	-1.2	7.5	131.1	C9 H10 N9 O2 F3 S Cl
400.0308	0.6	1.5	4.0	167.8	C9 H11 N6 O F6 S Cl
400.0303	1.1	2.7	6.0	125.8	C9 H14 N8 O3 F S2 Cl
400.0314	0.0	0.0	1.5	166.4	C9 H18 N5 O F3 S3 Cl
400.0298	1.6	4.0	0.0	212.4	C9 H22 N4 O2 F S4 Cl

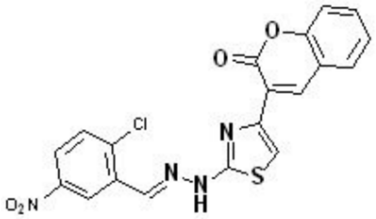

5h

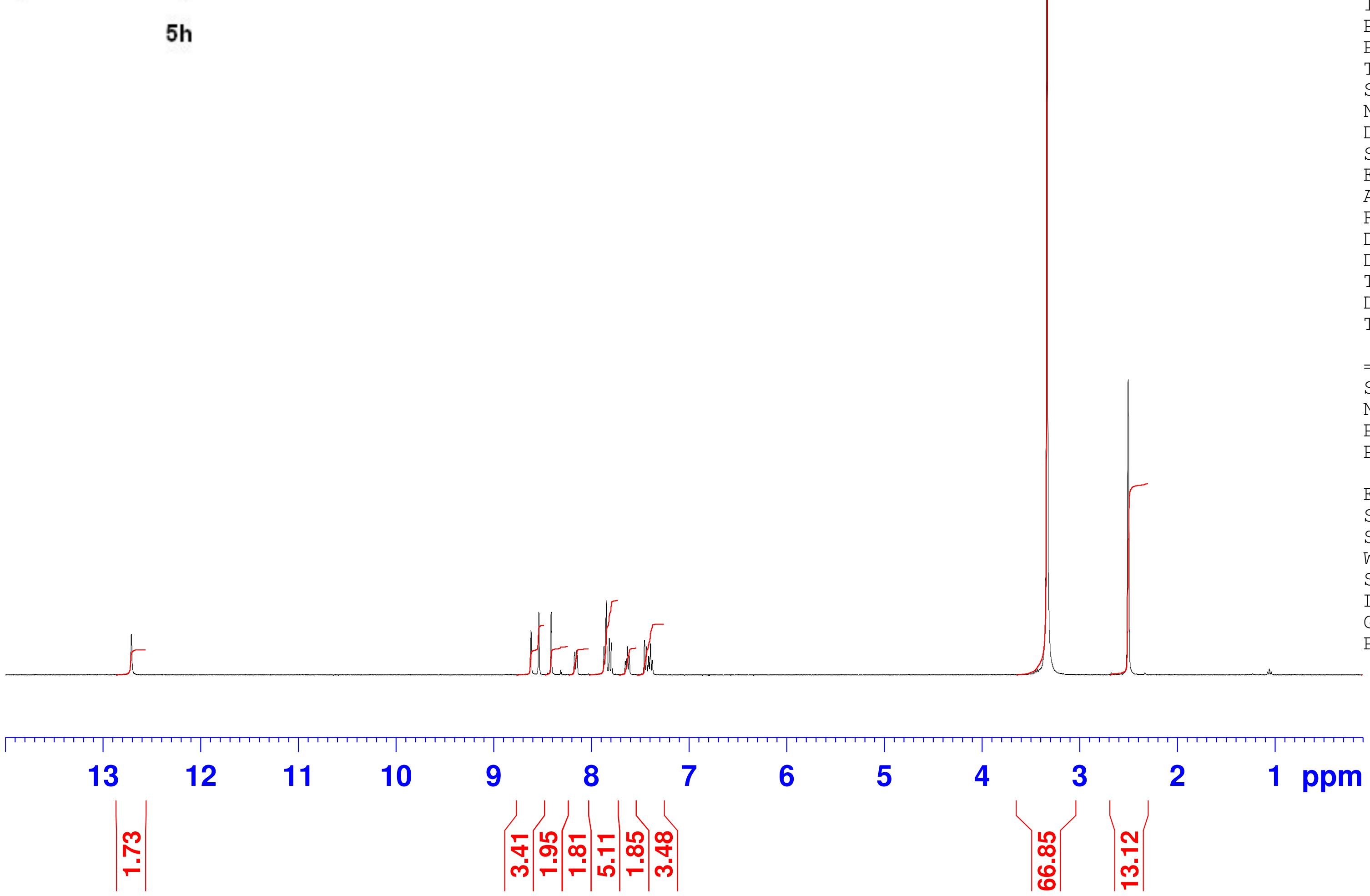

BRUKER

Current Data Parameters
NAME MG78
EXPNO 1
PROCNO 1

F2 - Acquisition Parameters
Date_ 20130227
Time 18.35
INSTRUM spect
PROBHD 5 mm PABBO BB-
PULPROG zg30
TD 65536
SOLVENT DMSO
NS 16
DS 2
SWH 8012.820 Hz
FIDRES 0.122266 Hz
AQ 4.0894465 sec
RG 203
DW 62.400 usec
DE 6.50 usec
TE 298.1 K
D1 1.00000000 sec
TD0 1

======== CHANNEL f1 ========
SFO1 400.1424710 MHz
NUC1 1H
P1 13.50 usec
PLW1 16.00000000 W

F2 - Processing parameters
SI 65536
SF 400.1400000 MHz
WDW EM
SSB 0
LB 0.30 Hz
GB 0
PC 1.40

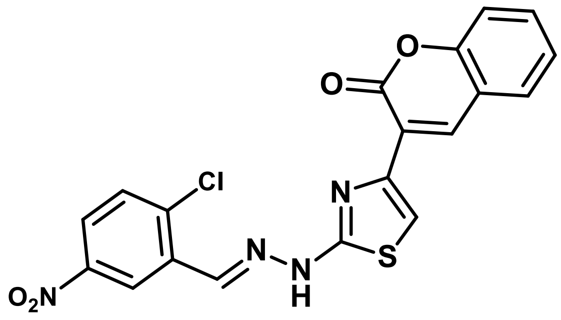

5h

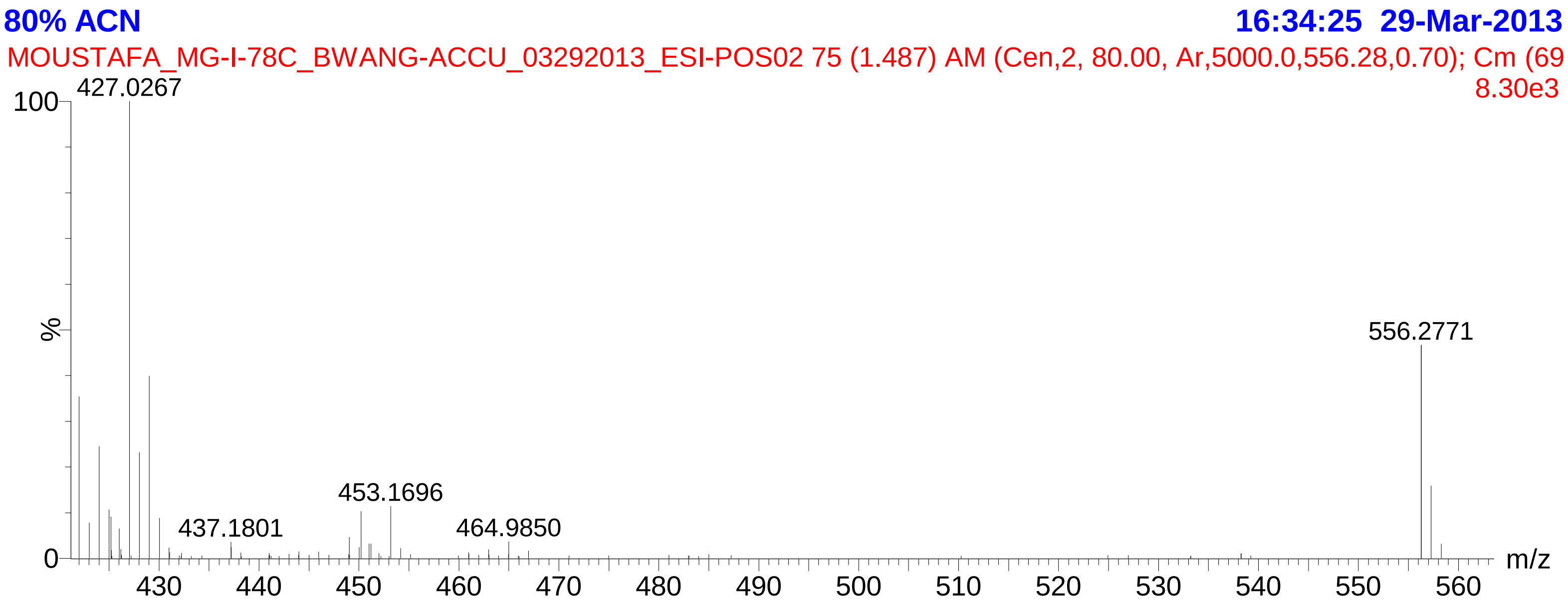

Elemental Composition Report

Single Mass Analysis

Tolerance = 5.0 PPM / DBE: min = -1.5, max = 50.0
Element prediction: Off
Number of isotope peaks used for i-FIT = 3

Monoisotopic Mass, Odd and Even Electron Ions
3911 formula(e) evaluated with 41 results within limits (all results (up to 1000) for each mass)
Elements Used:
C: 1-150 H: 1-150 N: 1-30 O: 1-60 S: 1-10 Cl: 1-2

Minimum:				-1.5		
Maximum:		5.0	5.0	50.0		
Mass	Calc. Mass	mDa	PPM	DBE	i-FIT	Formula
427.0267	427.0246	2.1	4.9	4.0	411.9	C H10 N15 O8 S Cl
	427.0278	-1.1	-2.6	4.0	906.8	C H11 N17 O4 S Cl2
	427.0261	0.6	1.4	12.0	61.8	C10 H10 N13 O S2 Cl
	427.0248	1.9	4.4	1.5	163.8	C10 H20 N2 O10 S2 Cl
	427.0280	-1.3	-3.0	1.5	803.5	C10 H21 N4 O6 S2 Cl2
	427.0261	0.6	1.4	6.5	92.9	C11 H16 N6 O6 S2 Cl
	427.0257	1.0	2.3	0.5	201.0	C11 H24 N2 O5 S4 Cl
	427.0288	-2.1	-4.9	0.5	947.8	C11 H25 N4 O S4 Cl2
	427.0246	2.1	4.9	12.0	572.0	C12 H11 N11 O S Cl2
	427.0275	-0.8	-1.9	11.5	43.3	C12 H12 N10 O2 S2 Cl
	427.0270	-0.3	-0.7	5.5	143.0	C12 H20 N6 O S4 Cl
	427.0246	2.1	4.9	6.5	622.8	C13 H17 N4 O6 S Cl2
	427.0275	-0.8	-1.9	6.0	69.8	C13 H18 N3 O7 S2 Cl
	427.0259	0.8	1.9	11.5	553.6	C14 H13 N8 O2 S Cl2
	427.0288	-2.1	-4.9	11.0	30.6	C14 H14 N7 O3 S2 Cl
	427.0255	1.2	2.8	5.5	778.9	C14 H21 N4 O S3 Cl2
	427.0283	-1.6	-3.7	5.0	130.8	C14 H22 N3 O2 S4 Cl
	427.0259	0.8	1.9	6.0	601.2	C15 H19 N O7 S Cl2
	427.0273	-0.6	-1.4	11.0	540.0	C16 H15 N5 O3 S Cl2
	427.0268	-0.1	-0.2	5.0	762.9	C16 H23 N O2 S3 Cl2

427.0254	1.3	3.0	16.0	1.3	C17 H10 N7 O3 S Cl
427.0250	1.7	4.0	10.0	51.5	C17 H18 N3 O2 S3 Cl
427.0286	-1.9	-4.4	10.5	530.0	C18 H17 N2 O4 S Cl2
427.0268	-0.1	-0.2	15.5	0.5	C19 H12 N4 O4 S Cl
427.0255	1.2	2.8	3.0	336.3	C2 H14 N15 O3 S3 Cl
427.0246	2.1	4.9	-1.5	481.1	C2 H16 N8 O13 S Cl
427.0278	-1.1	-2.6	-1.5	986.0	C2 H17 N10 O9 S Cl2
427.0259	0.8	1.9	9.0	288.5	C2 H6 N19 O4 S Cl
427.0281	-1.4	-3.3	15.0	4.5	C21 H14 N O5 S Cl
427.0259	0.8	1.9	3.5	346.4	C3 H12 N12 O9 S Cl
427.0268	-0.1	-0.2	2.5	287.4	C4 H16 N12 O4 S3 Cl
427.0273	-0.6	-1.4	8.5	234.8	C4 H8 N16 O5 S Cl
427.0273	-0.6	-1.4	3.0	287.8	C5 H14 N9 O10 S Cl
427.0286	-1.9	-4.4	13.5	146.6	C5 H4 N20 O S Cl
427.0286	-1.9	-4.4	8.0	187.8	C6 H10 N13 O6 S Cl
427.0253	1.4	3.3	2.5	869.4	C6 H17 N10 O4 S2 Cl2
427.0282	-1.5	-3.5	2.0	244.6	C6 H18 N9 O5 S3 Cl
427.0286	-1.9	-4.4	2.5	236.0	C7 H16 N6 O11 S Cl
427.0266	0.1	0.2	2.0	834.4	C8 H19 N7 O5 S2 Cl2
427.0248	1.9	4.4	7.0	121.7	C9 H14 N9 O5 S2 Cl
427.0280	-1.3	-3.0	7.0	744.5	C9 H15 N11 O S2 Cl2

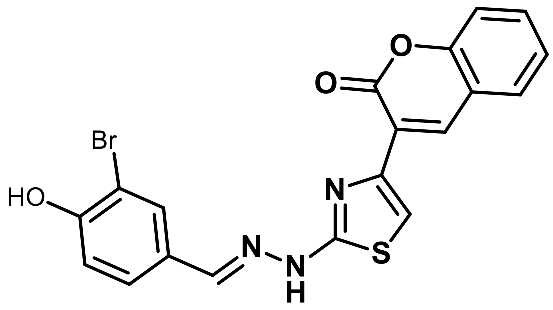

5i

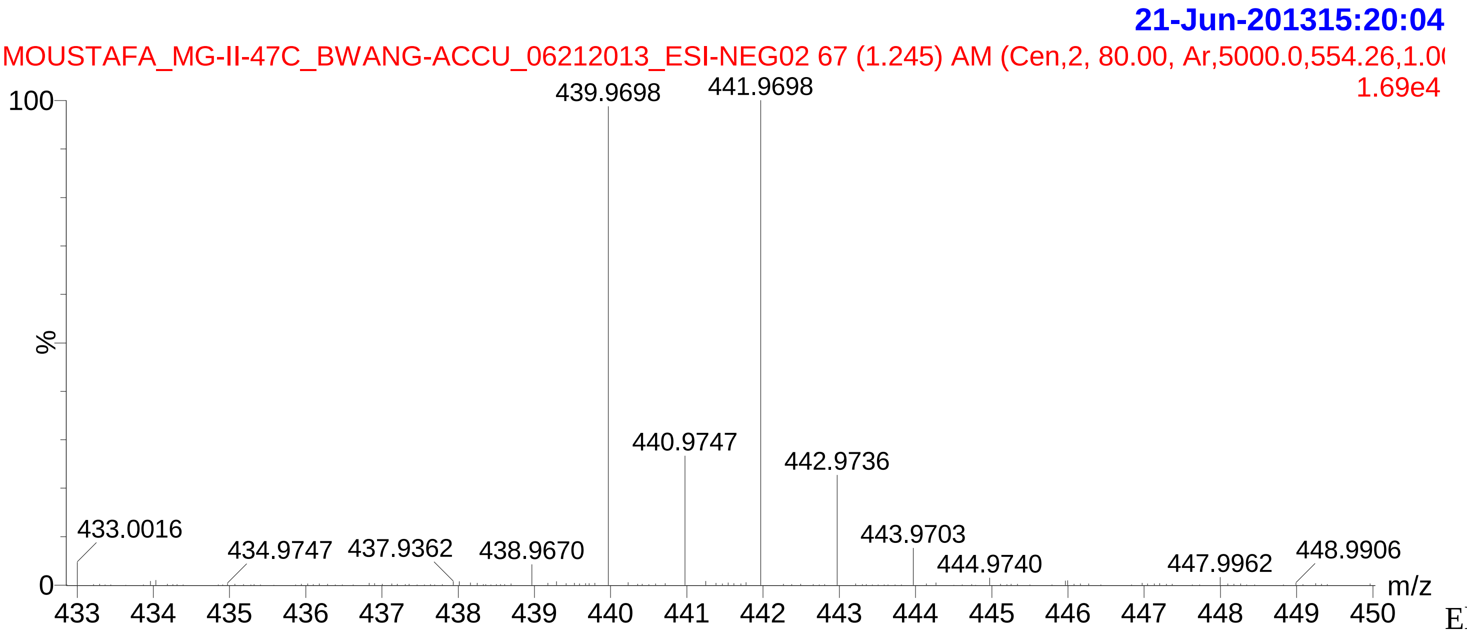

Elemental Composition Report

Single Mass Analysis

Tolerance = 5.0 PPM / DBE: min = -1.5, max = 100.0
Element prediction: Off
Number of isotope peaks used for i-FIT = 3

Monoisotopic Mass, Even Electron Ions
1335 formula(e) evaluated with 11 results within limits (all results (up to 1000) for each mass)
Elements Used:
C: 1-150 H: 1-150 N: 1-30 O: 1-60 S: 1-2 Br: 1-2

Minimum:				-1.5		
Maximum:		5.0	5.0	100.0		
Mass	Calc. Mass	mDa	PPM	DBE	i-FIT	Formula
439.9698	439.9685	1.3	3.0	1.5	598.0	C10 H19 N O9 S2 Br
	439.9677	2.1	4.8	-0.5	2775.7	C10 H24 N3 O2 S2 Br2
	439.9698	0.0	0.0	6.5	412.5	C11 H15 N5 O5 S2 Br
	439.9711	-1.3	-3.0	11.5	263.7	C12 H11 N9 O S2 Br
	439.9678	2.0	4.5	16.5	125.2	C15 H7 N9 O S Br
	439.9704	-0.6	-1.4	15.5	53.6	C19 H11 N3 O3 S Br
	439.9683	1.5	3.4	-1.5	1343.1	C2 H15 N7 O12 S Br
	439.9696	0.2	0.5	3.5	1046.8	C3 H11 N11 O8 S Br
	439.9688	1.0	2.3	1.5	3033.5	C3 H16 N13 O S Br2
	439.9710	-1.2	-2.7	8.5	787.3	C4 H7 N15 O4 S Br
	439.9715	-1.7	-3.9	0.5	2855.8	C7 H20 N7 O3 S Br2

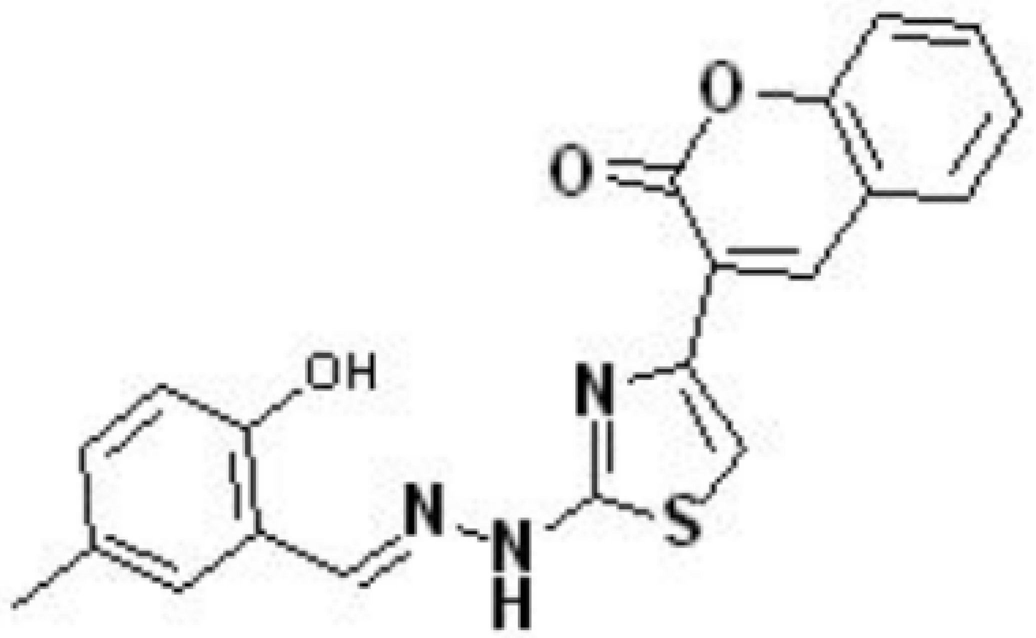

5j

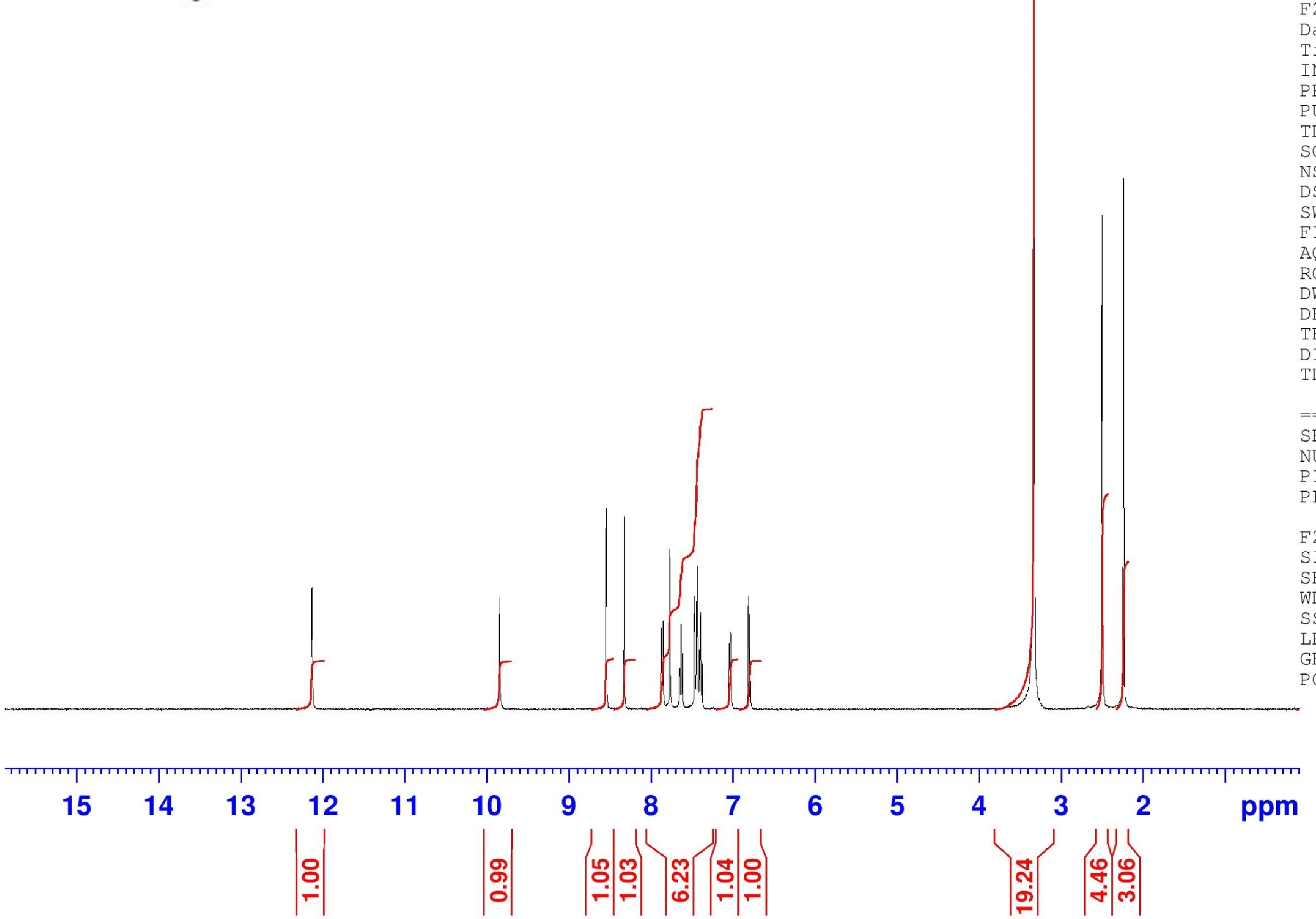

BRUKER

```
Current Data Parameters
NAME                 MG121
EXPNO                    1
PROCNO                   1

F2 - Acquisition Parameters
Date_             20130327
Time                 12.08
INSTRUM              spect
PROBHD   5 mm PABBO BB-
PULPROG               zg30
TD                   65536
SOLVENT               DMSO
NS                      16
DS                       2
SWH             8012.820 Hz
FIDRES          0.122266 Hz
AQ             4.0894465 sec
RG                     181
DW                62.400 usec
DE                  6.50 usec
TE                 298.0 K
D1            1.00000000 sec
TD0                      1

======== CHANNEL f1 ========
SFO1         400.1424710 MHz
NUC1                    1H
P1                 13.50 usec
PLW1         16.00000000 W

F2 - Processing parameters
SI                   65536
SF           400.1400000 MHz
WDW                     EM
SSB      0
LB                  0.30 Hz
GB       0
PC                    1.00
```

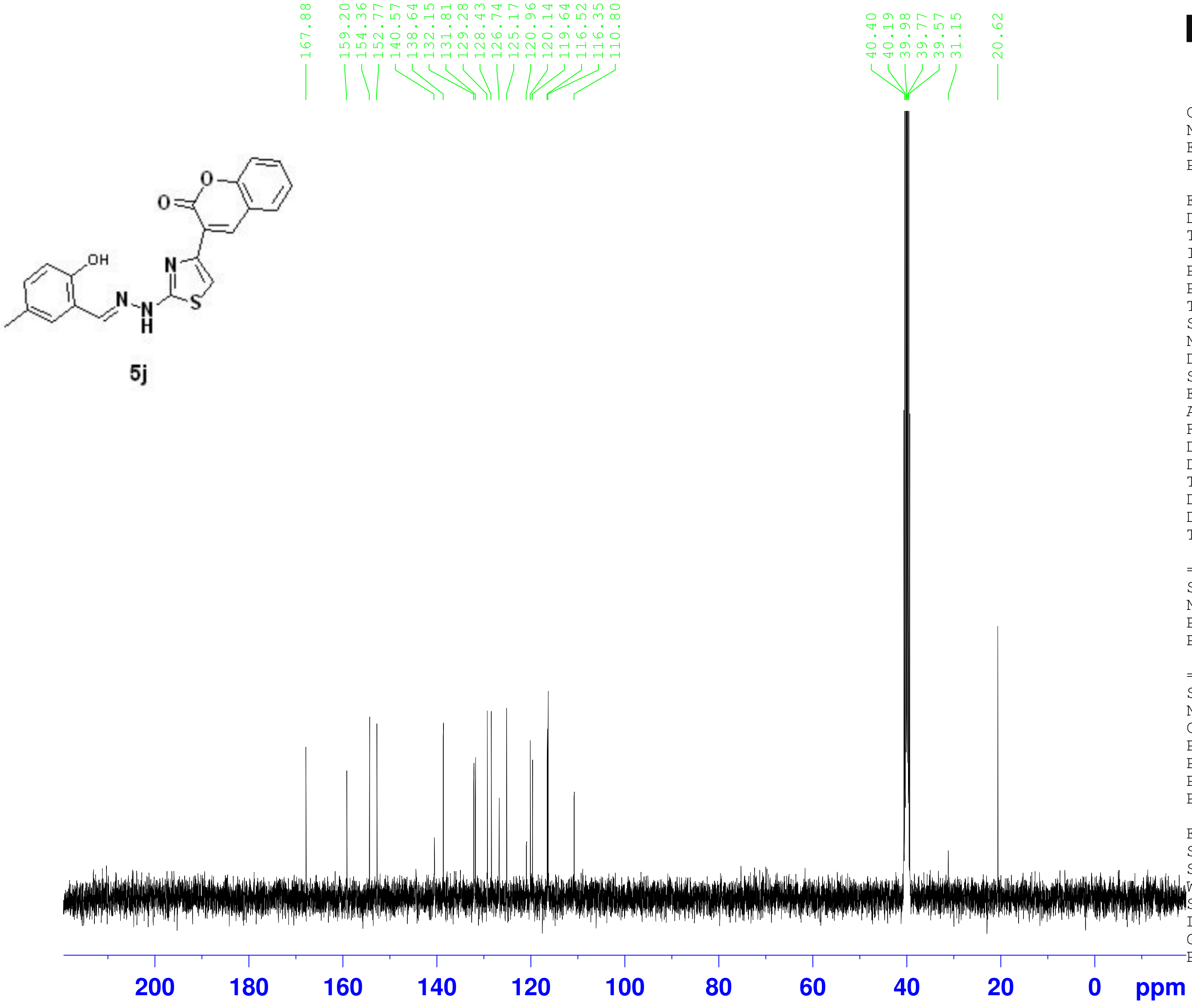

BRUKER

Current Data Parameters
NAME MG121c
EXPNO 1
PROCNO 1

F2 - Acquisition Parameters
Date_ 20130401
Time 13.23
INSTRUM spect
PROBHD 5 mm PABBO BB-
PULPROG zgpg30
TD 65536
SOLVENT DMSO
NS 187
DS 4
SWH 24038.461 Hz
FIDRES 0.366798 Hz
AQ 1.3631488 sec
RG 161
DW 20.800 usec
DE 6.50 usec
TE 298.0 K
D1 2.00000000 sec
D11 0.03000000 sec
TD0 1

======== CHANNEL f1 ========
SFO1 100.6253441 MHz
NUC1 13C
P1 9.00 usec
PLW1 62.00000000 W

======== CHANNEL f2 ========
SFO2 400.1416006 MHz
NUC2 1H
CPDPRG[2 waltz16
PCPD2 90.00 usec
PLW2 16.00000000 W
PLW12 0.36000001 W
PLW13 0.29159999 W

F2 - Processing parameters
SI 32768
SF 100.6152830 MHz
WDW EM
SSB 0
LB 1.00 Hz
GB 0
PC 1.40

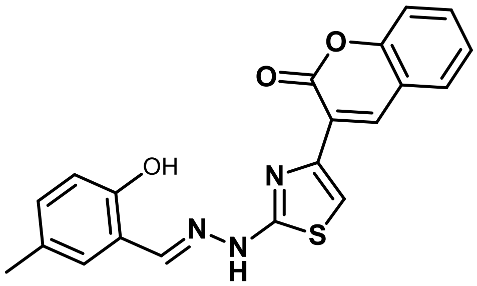

5j

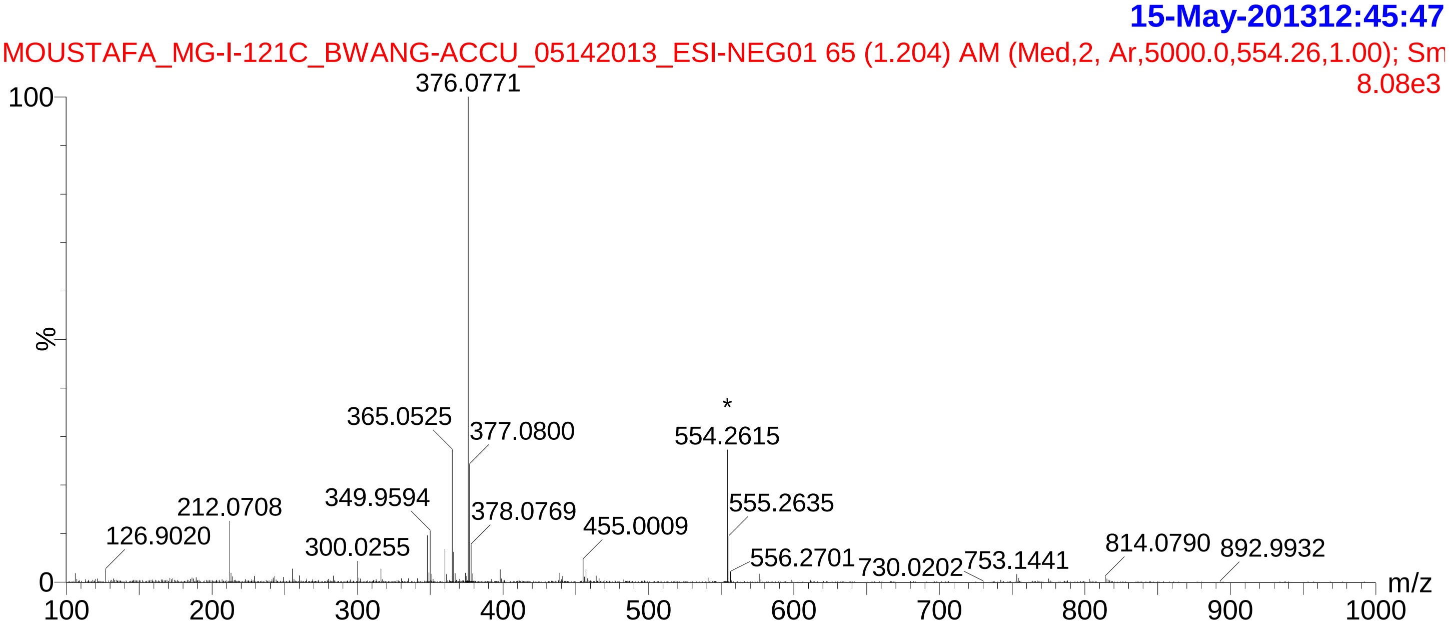

Elemental Composition Report

Single Mass Analysis

Tolerance = 5.0 PPM / DBE: min = -1.5, max = 50.0

Element prediction: Off
Number of isotope peaks used for i-FIT = 3

Monoisotopic Mass, Even Electron Ions
1659 formula(e) evaluated with 9 results within limits (all results (up to 1000) for each mass)
Elements Used:
C: 1-150 H: 1-150 N: 1-30 O: 1-60 S: 1-4

Minimum:				-1.5		
Maximum:		5.0	5.0	50.0		
Mass	Calc. Mass	mDa	PPM	DBE	i-FIT	Formula
376.0771	376.0781	-1.0	-2.7	-1.5	525.5	C H18 N11 O8 S2
	376.0763	0.8	2.1	11.5	88.8	C13 H14 N9 O S2
	376.0790	-1.9	-5.1	10.5	89.9	C17 H18 N3 O3 S2
	376.0756	1.5	4.0	15.5	0.6	C20 H14 N3 O3 S
	376.0761	1.0	2.7	8.5	240.8	C5 H10 N15 O4 S
	376.0756	1.5	4.0	2.5	470.8	C5 H18 N11 O3 S3
	376.0774	-0.3	-0.8	2.5	229.5	C8 H18 N5 O10 S
	376.0788	-1.7	-4.5	7.5	147.6	C9 H14 N9 O6 S
	376.0783	-1.2	-3.2	1.5	427.7	C9 H22 N5 O5 S3

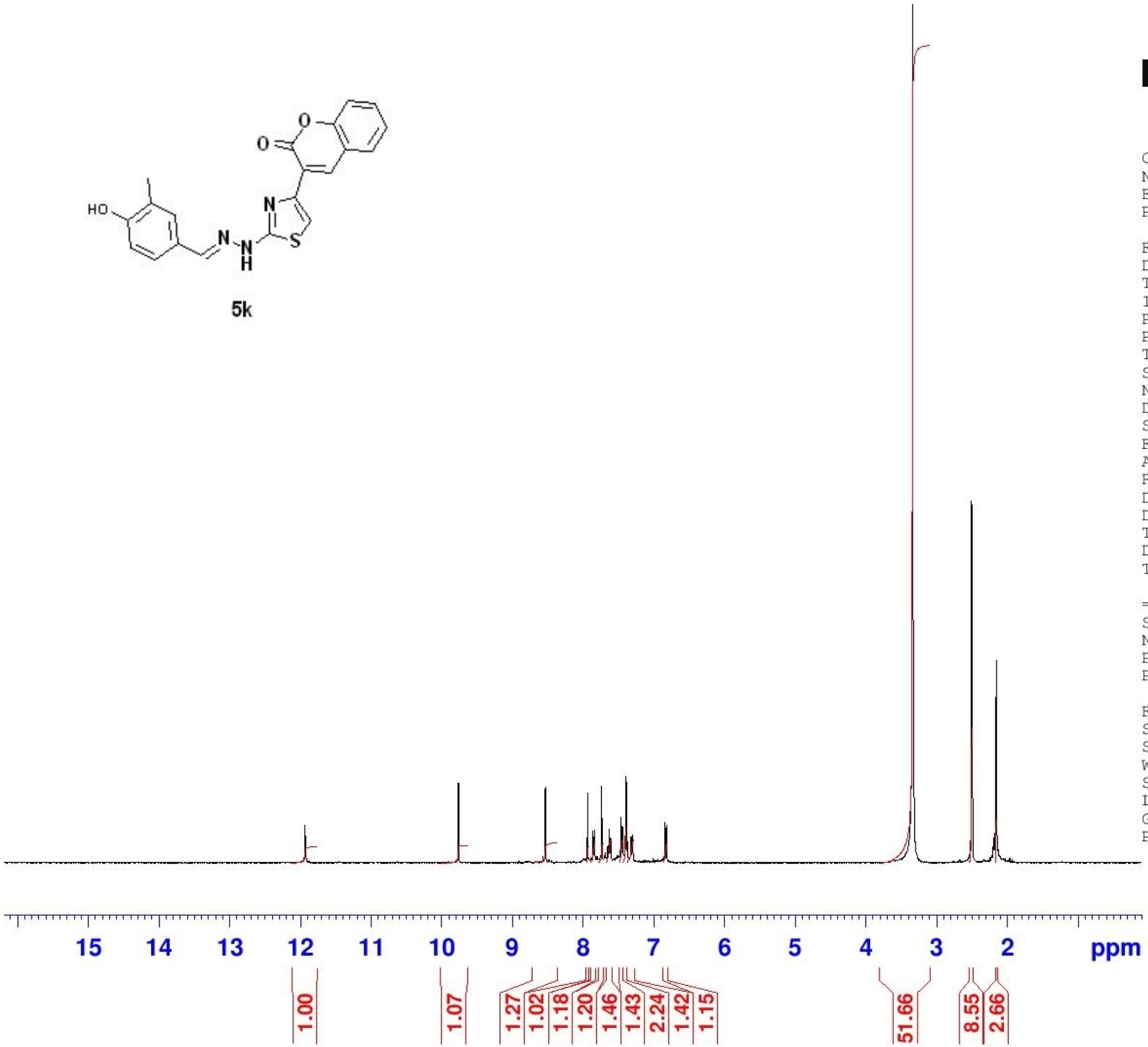

5k

Current Data Parameters
NAME MG95pp
EXPNO 1
PROCNO 1

F2 - Acquisition Parameters
Date_ 20130328
Time 18.27
INSTRUM spect
PROBHD 5 mm PABBO BB-
PULPROG zg30
TD 65536
SOLVENT DMSO
NS 16
DS 2
SWH 8012.820 Hz
FIDRES 0.122266 Hz
AQ 4.0894465 sec
RG 181
DW 62.400 usec
DE 6.50 usec
TE 298.0 K
D1 1.00000000 sec
TD0 1

======== CHANNEL f1 ========
SFO1 400.1424710 MHz
NUC1 1H
P1 13.50 usec
PLW1 16.00000000 W

F2 - Processing parameters
SI 65536
SF 400.1400000 MHz
WDW EM
SSB 0
LB 0.30 Hz
GB 0
PC 1.40

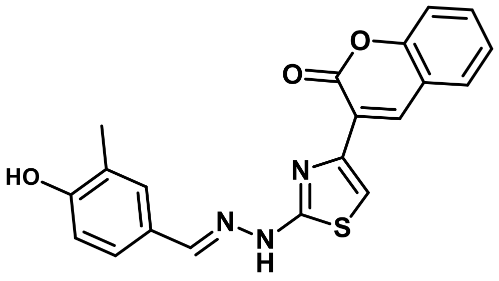

5k

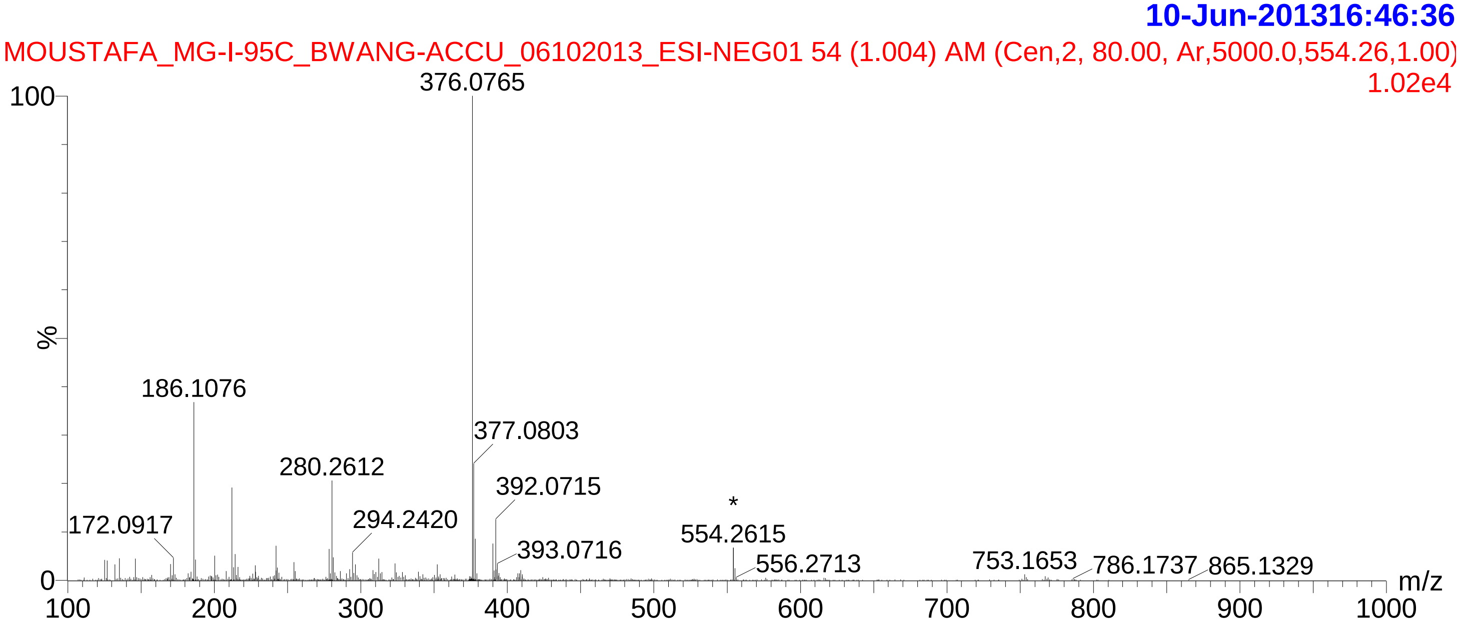

Elemental Composition Report

Single Mass Analysis
Tolerance = 5.0 PPM / DBE: min = -1.5, max = 100.0

Element prediction: Off
Number of isotope peaks used for i-FIT = 3

Monoisotopic Mass, Odd and Even Electron Ions
1078 formula(e) evaluated with 12 results within limits (all results (up to 1000) for each mass)
Elements Used:
C: 1-150 H: 1-150 N: 1-30 O: 1-60 S: 1-2

Minimum:				-1.5			
Maximum:		5.0	5.0	100.0			
Mass	Calc. Mass		mDa	PPM	DBE	i-FIT	Formula
376.0765	376.0781		-1.6	-4.3	-1.5	620.9	C H18 N11 O8 S2
	376.0749		1.6	4.3	6.5	144.3	C12 H18 N5 O5 S2
	376.0763		0.2	0.5	11.5	76.0	C13 H14 N9 O S2
	376.0763		0.2	0.5	6.0	127.1	C14 H20 N2 O6 S2
	376.0776		-1.1	-2.9	11.0	69.7	C15 H16 N6 O2 S2
	376.0756		0.9	2.4	15.5	2.9	C20 H14 N3 O3 S
	376.0747		1.8	4.8	9.0	386.1	C3 H8 N18 O3 S
	376.0748		1.7	4.5	3.5	431.3	C4 H14 N11 O8 S
	376.0761		0.4	1.1	8.5	311.2	C5 H10 N15 O4 S
	376.0761		0.4	1.1	3.0	354.3	C6 H16 N8 O9 S
	376.0774		-0.9	-2.4	8.0	246.4	C7 H12 N12 O5 S
	376.0774		-0.9	-2.4	2.5	287.0	C8 H18 N5 O10 S

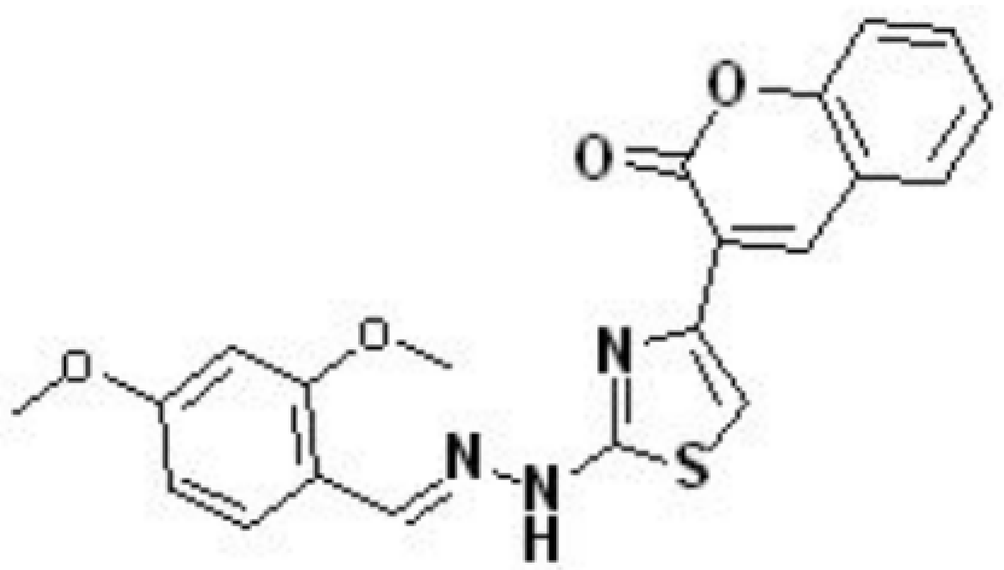

5l

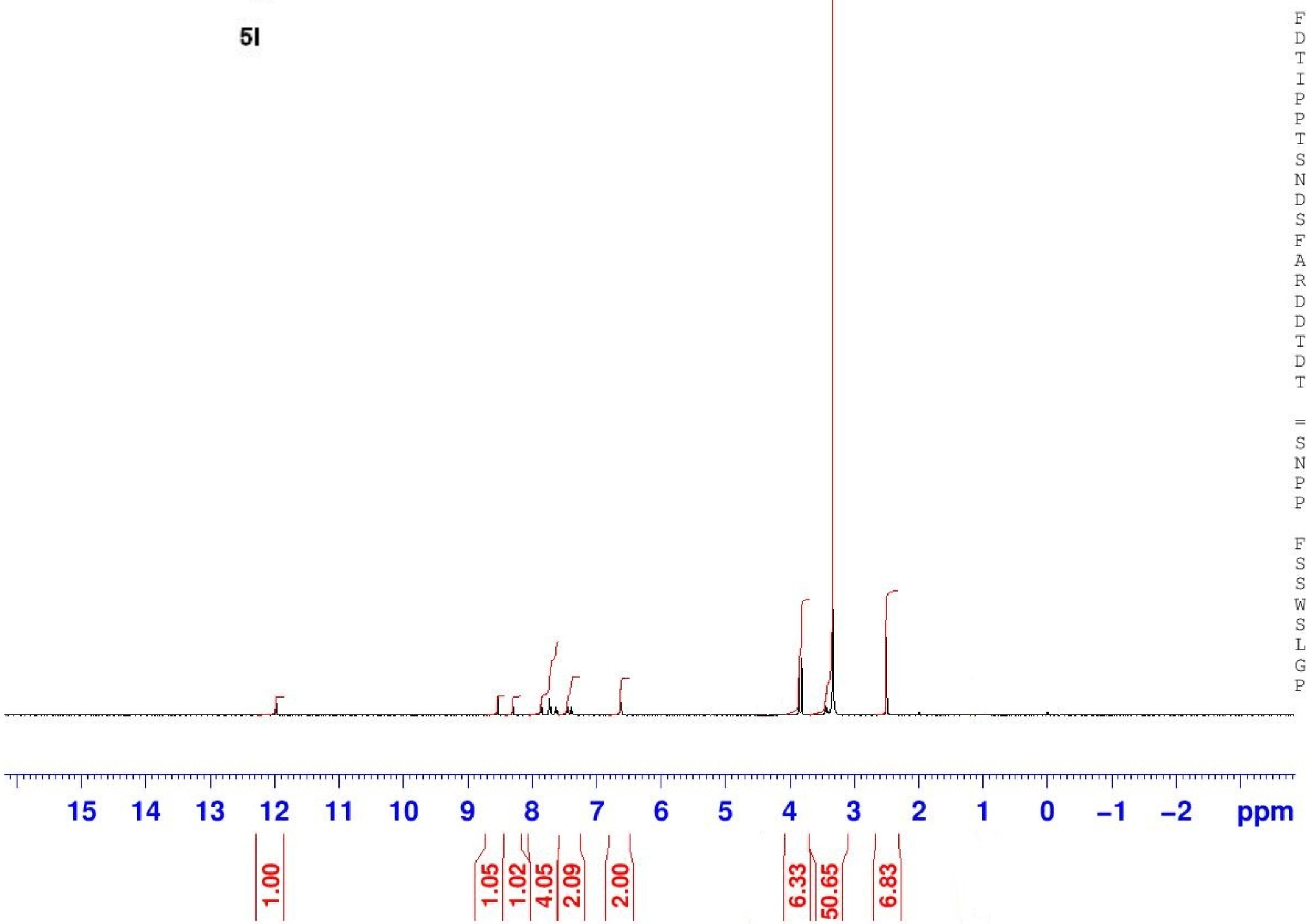

BRUKER

Current Data Parameters
NAME MG75
EXPNO 1
PROCNO 1

F2 - Acquisition Parameters
Date_ 20130227
Time 18.41
INSTRUM spect
PROBHD 5 mm PABBO BB-
PULPROG zg30
TD 65536
SOLVENT DMSO
NS 16
DS 2
SWH 8012.820 Hz
FIDRES 0.122266 Hz
AQ 4.0894465 sec
RG 181
DW 62.400 usec
DE 6.50 usec
TE 298.0 K
D1 1.00000000 sec
TD0 1

======== CHANNEL f1 ========
SFO1 400.1424710 MHz
NUC1 1H
P1 13.50 usec
PLW1 16.00000000 W

F2 - Processing parameters
SI 65536
SF 400.1400000 MHz
WDW EM
SSB 0
LB 0.30 Hz
GB 0
PC 1.40

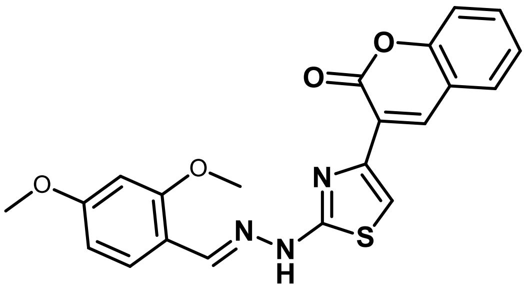

5l

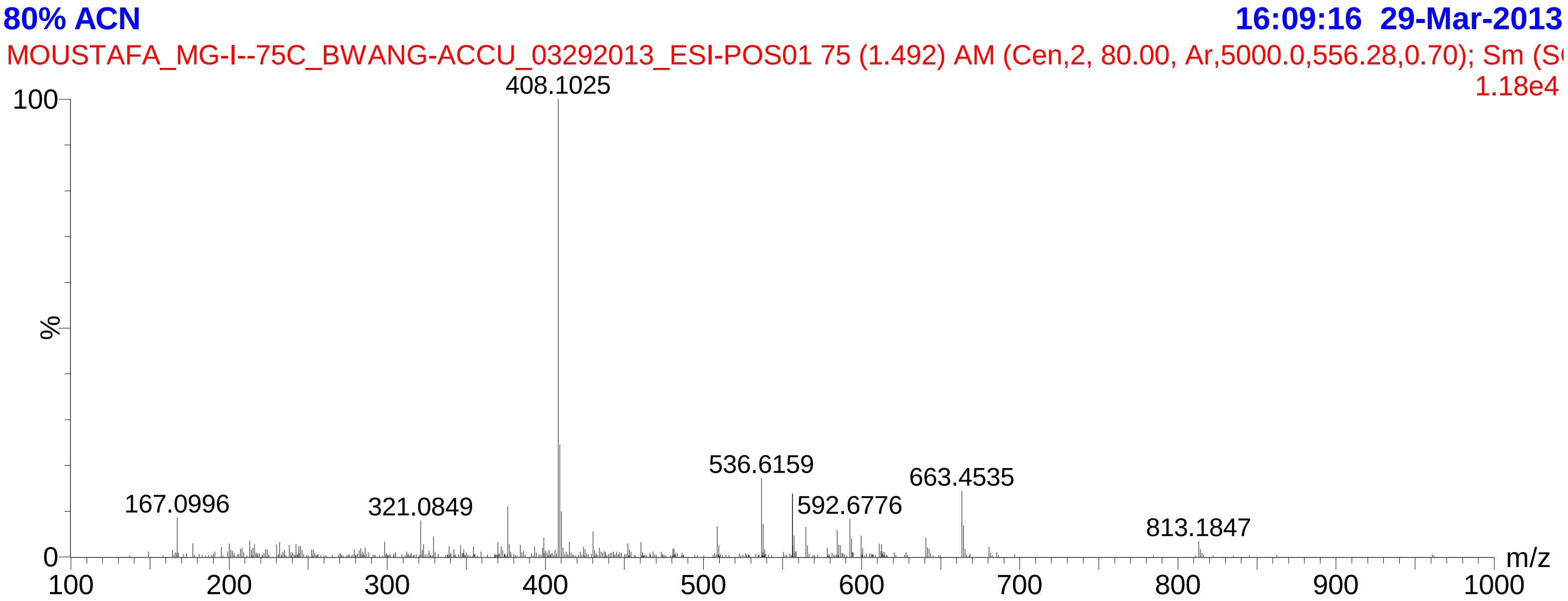

Elemental Composition Report

Single Mass Analysis

Tolerance = 5.0 PPM / DBE: min = -1.5, max = 50.0

Element prediction: Off
Number of isotope peaks used for i-FIT = 3

Monoisotopic Mass, Odd and Even Electron Ions
2791 formula(e) evaluated with 24 results within limits (all results (up to 1000) for each mass)
Elements Used:
C: 1-150   H: 1-150   N: 1-30   O: 1-60   S: 1-10

Minimum:				-1.5		
Maximum:		5.0	5.0	50.0		
Mass	Calc. Mass	mDa	PPM	DBE	i-FIT	Formula
408.1025	408.1037	-1.2	-2.9	1.5	303.6	C9 H22 N5 O11 S
	408.1045	-2.0	-4.9	6.0	279.4	C9 H20 N12 O S3
	408.1032	-0.7	-1.7	1.0	403.6	C8 H24 N8 O5 S3
	408.1036	-1.1	-2.7	7.0	270.6	C8 H16 N12 O6 S
	408.1023	0.2	0.5	2.0	379.2	C7 H20 N8 O10 S
	408.1036	-1.1	-2.7	12.5	254.1	C7 H10 N19 O S
	408.1018	0.7	1.7	1.5	443.0	C6 H22 N11 O4 S3
	408.1023	0.2	0.5	7.5	343.0	C6 H14 N15 O5 S
	408.1010	1.5	3.7	2.5	464.9	C5 H18 N11 O9 S
	408.1005	2.0	4.9	2.0	491.8	C4 H20 N14 O3 S3
	408.1010	1.5	3.7	8.0	425.6	C4 H12 N18 O4 S
	408.1045	-2.0	-4.9	19.0	52.7	C24 H16 N4 O S
	408.1018	0.7	1.7	14.5	16.6	C21 H18 N3 O4 S
	408.1005	2.0	4.9	15.0	23.7	C19 H16 N6 O3 S
	408.1034	-0.9	-2.2	4.0	538.8	C16 H28 N2 O2 S4
	408.1038	-1.3	-3.2	10.0	37.2	C16 H20 N6 O3 S2
	408.1025	0.0	0.0	5.0	86.7	C15 H24 N2 O7 S2
	408.1020	0.5	1.2	4.5	528.1	C14 H26 N5 O S4
	408.1025	0.0	0.0	10.5	45.3	C14 H18 N9 O2 S2
	408.1007	1.8	4.4	-0.5	628.3	C13 H30 N O5 S4
	408.1012	1.3	3.2	5.5	106.9	C13 H22 N5 O6 S2

408.1011	1.4	3.4	11.0	62.2	C12 H16 N12 O S2
408.1045	-2.0	-4.9	0.5	373.7	C10 H26 N5 O6 S3
408.1043	-1.8	-4.4	3.0	516.6	C H16 N18 O4 S2

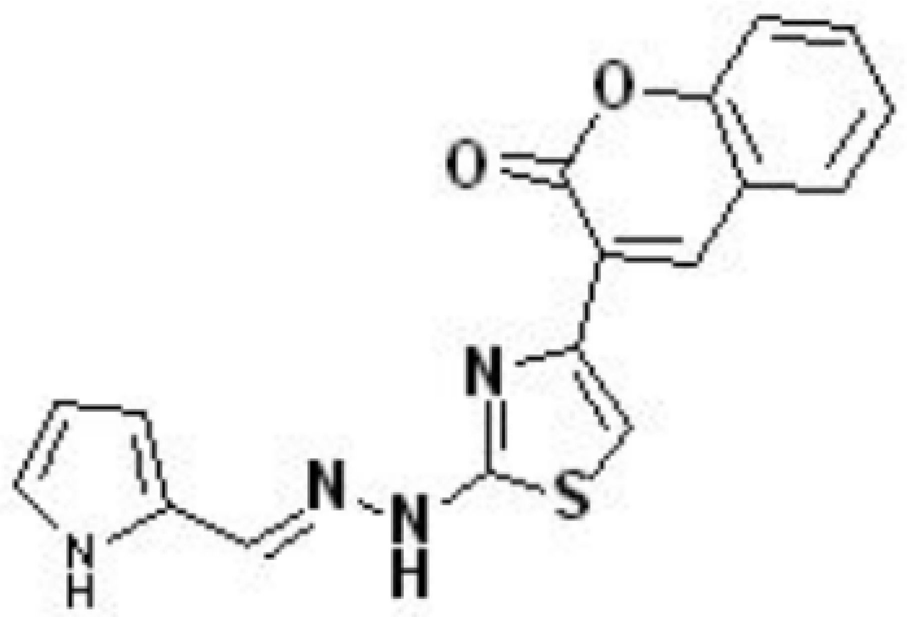

5m

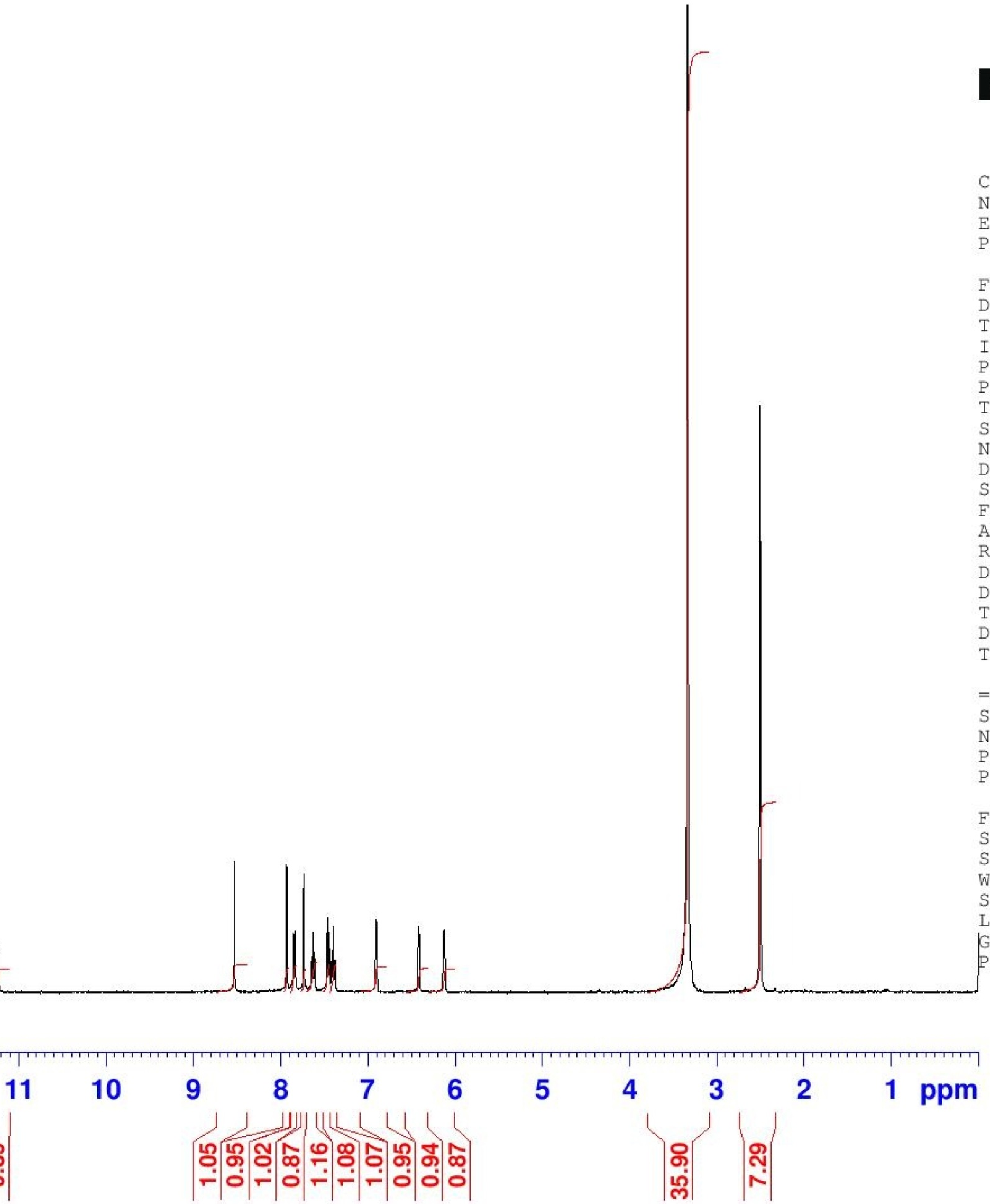


```
Current Data Parameters
NAME                 MG124
EXPNO                    1
PROCNO                   1

F2 - Acquisition Parameters
Date_             20130327
Time                 12.03
INSTRUM              spect
PROBHD   5 mm PABBO BB-
PULPROG               zg30
TD                   65536
SOLVENT               DMSO
NS                      16
DS                       2
SWH               8012.820 Hz
FIDRES            0.122266 Hz
AQ               4.0894465 sec
RG                     181
DW                  62.400 usec
DE                    6.50 usec
TE                   297.9 K
D1              1.00000000 sec
TD0                      1

======== CHANNEL f1 ========
SFO1           400.1424710 MHz
NUC1                    1H
P1                   13.50 usec
PLW1           16.00000000 W

F2 - Processing parameters
SI                   65536
SF             400.1400000 MHz
WDW                     EM
SSB      0
LB                    0.30 Hz
GB       0
PC                    1.00
```

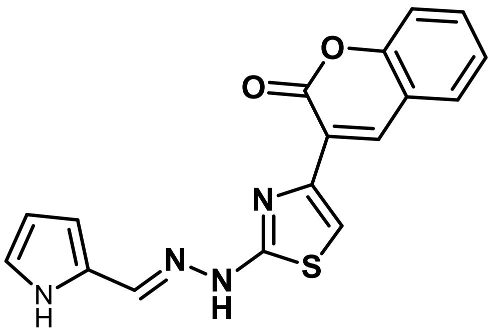

5m

17-May-201315:20:56

MOUSTAFA_MG-I-124C_BWANG-ACCU_05172013_ESI-NEG01 75 (1.397) AM (Cen,2, 80.00, Ar,5000.0,554.26,1.0

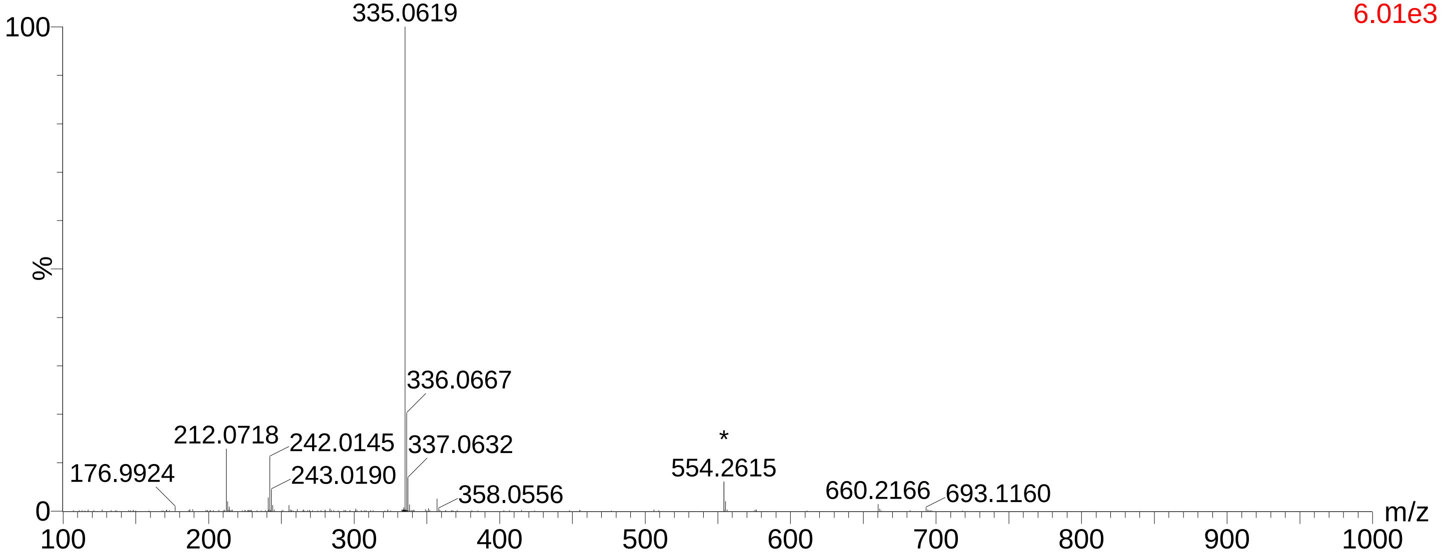

Elemental Composition Report

Single Mass Analysis
Tolerance = 5.0 PPM / DBE: min = -1.5, max = 50.0
Element prediction: Off
Number of isotope peaks used for i-FIT = 3

Monoisotopic Mass, Odd and Even Electron Ions
1139 formula(e) evaluated with 13 results within limits (all results (up to 1000) for each mass)
Elements Used:
C: 1-150 H: 1-150 N: 1-30 O: 1-60 S: 1-4

Minimum:				-1.5		
Maximum:		5.0	5.0	50.0		
Mass	Calc. Mass	mDa	PPM	DBE	i-FIT	Formula
335.0619	335.0610	0.9	2.7	5.0	106.0	C11 H17 N3 O5 S2
	335.0623	-0.4	-1.2	10.0	65.0	C12 H13 N7 O S2
	335.0603	1.6	4.8	14.5	2.6	C17 H11 N4 O2 S
	335.0616	0.3	0.9	14.0	7.6	C19 H13 N O3 S
	335.0603	1.6	4.8	1.5	383.9	C2 H15 N12 O2 S3
	335.0608	1.1	3.3	7.5	184.1	C2 H7 N16 O3 S
	335.0608	1.1	3.3	2.0	219.0	C3 H13 N9 O8 S
	335.0617	0.2	0.6	1.0	362.0	C4 H17 N9 O3 S3
	335.0621	-0.2	-0.6	7.0	142.8	C4 H9 N13 O4 S
	335.0621	-0.2	-0.6	1.5	175.9	C5 H15 N6 O9 S
	335.0635	-1.6	-4.8	6.5	107.9	C6 H11 N10 O5 S
	335.0630	-1.1	-3.3	0.5	345.4	C6 H19 N6 O4 S3
	335.0635	-1.6	-4.8	1.0	139.2	C7 H17 N3 O10 S

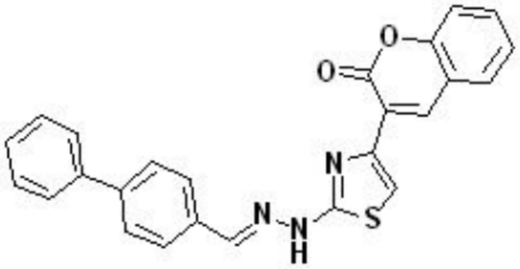

5n

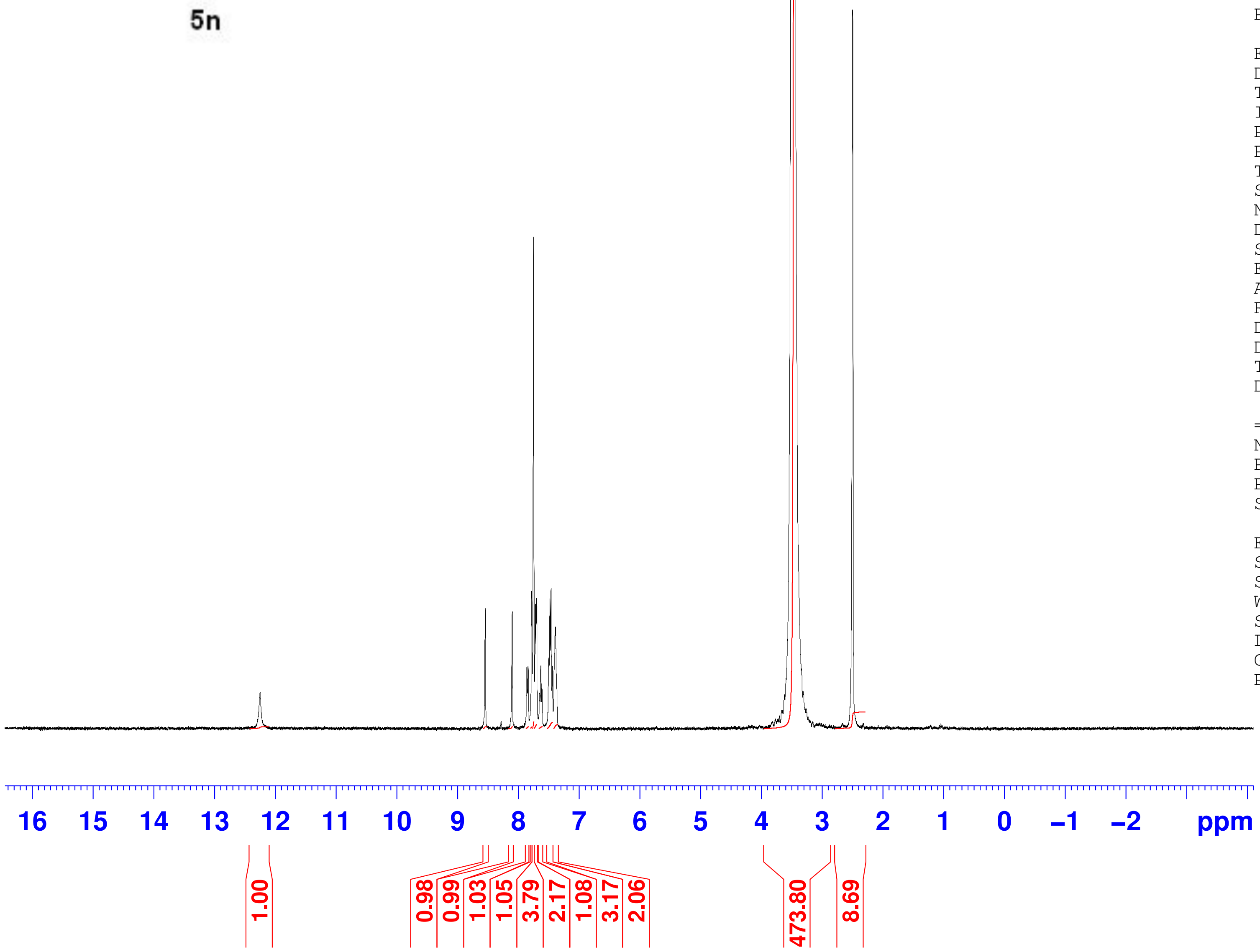

BRUKER

Current Data Parameters
NAME MG152
EXPNO 1
PROCNO 1

F2 - Acquisition Parameters
Date_ 20130417
Time 18.27
INSTRUM spect
PROBHD 5 mm PABBO BB-
PULPROG zg30
TD 65536
SOLVENT DMSO
NS 16
DS 2
SWH 8223.685 Hz
FIDRES 0.125483 Hz
AQ 3.9846387 sec
RG 64
DW 60.800 usec
DE 6.50 usec
TE 294.4 K
D1 1.00000000 sec

======== CHANNEL f1 ========
NUC1 1H
P1 13.50 usec
PLW1 16.00000000 W
SFO1 400.1424710 MHz

F2 - Processing parameters
SI 65536
SF 400.1400000 MHz
WDW EM
SSB 0
LB 0.30 Hz
GB 0
PC 1.00

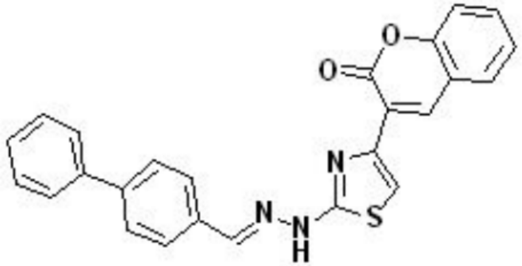

5n

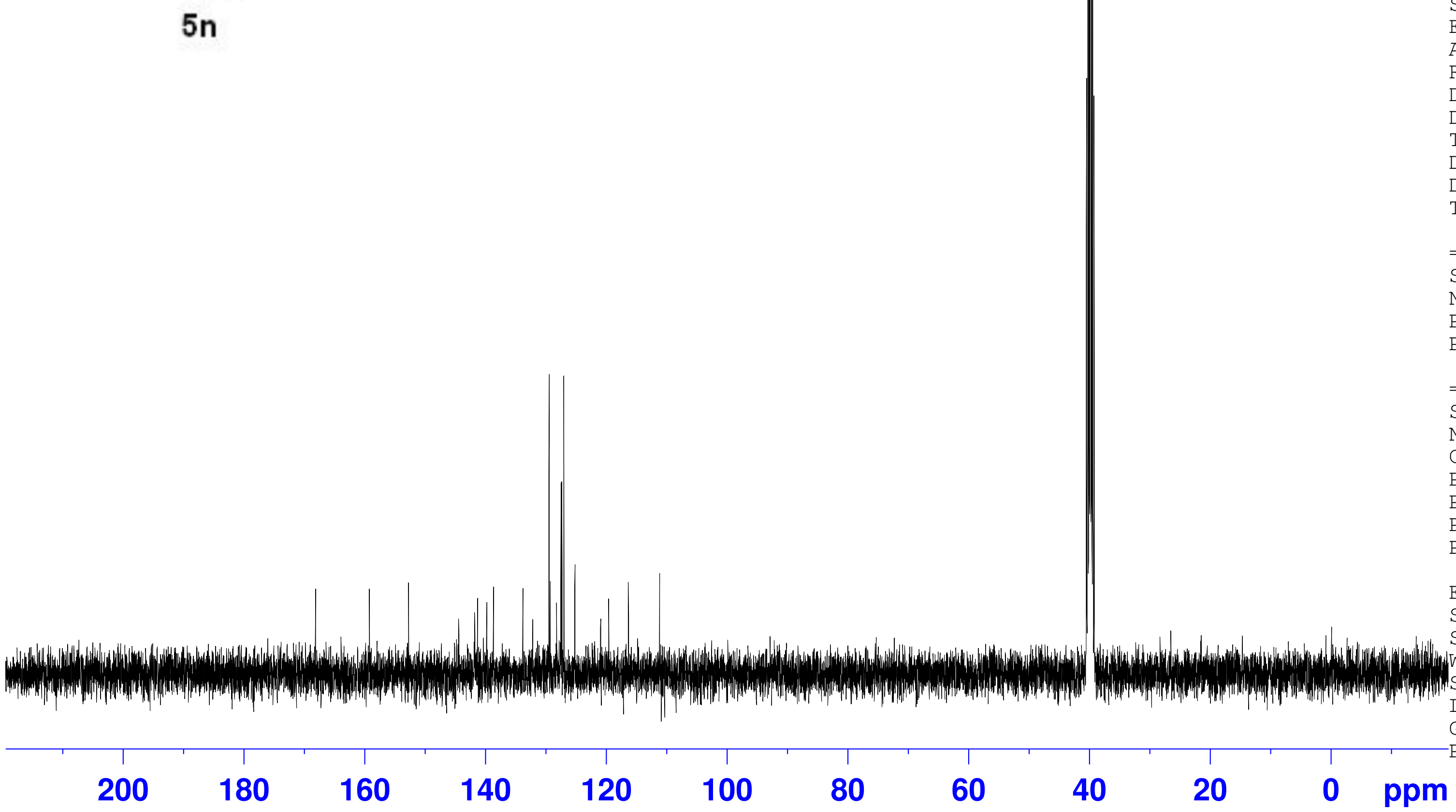

BRUKER

Current Data Parameters
NAME MG152c
EXPNO 1
PROCNO 1

F2 - Acquisition Parameters
Date_ 20130418
Time 17.25
INSTRUM spect
PROBHD 5 mm PABBO BB-
PULPROG zgpg30
TD 65536
SOLVENT DMSO
NS 157
DS 4
SWH 24038.461 Hz
FIDRES 0.366798 Hz
AQ 1.3631488 sec
RG 181
DW 20.800 usec
DE 6.50 usec
TE 298.0 K
D1 2.00000000 sec
D11 0.03000000 sec
TD0 1

======== CHANNEL f1 ========
SFO1 100.6253441 MHz
NUC1 13C
P1 9.00 usec
PLW1 62.00000000 W

======== CHANNEL f2 ========
SFO2 400.1416006 MHz
NUC2 1H
CPDPRG[2 waltz16
PCPD2 90.00 usec
PLW2 16.00000000 W
PLW12 0.36000001 W
PLW13 0.29159999 W

F2 - Processing parameters
SI 32768
SF 100.6152830 MHz
WDW EM
SSB 0
LB 1.00 Hz
GB 0
PC 1.40

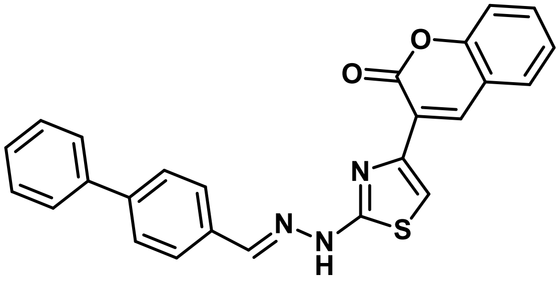

5n

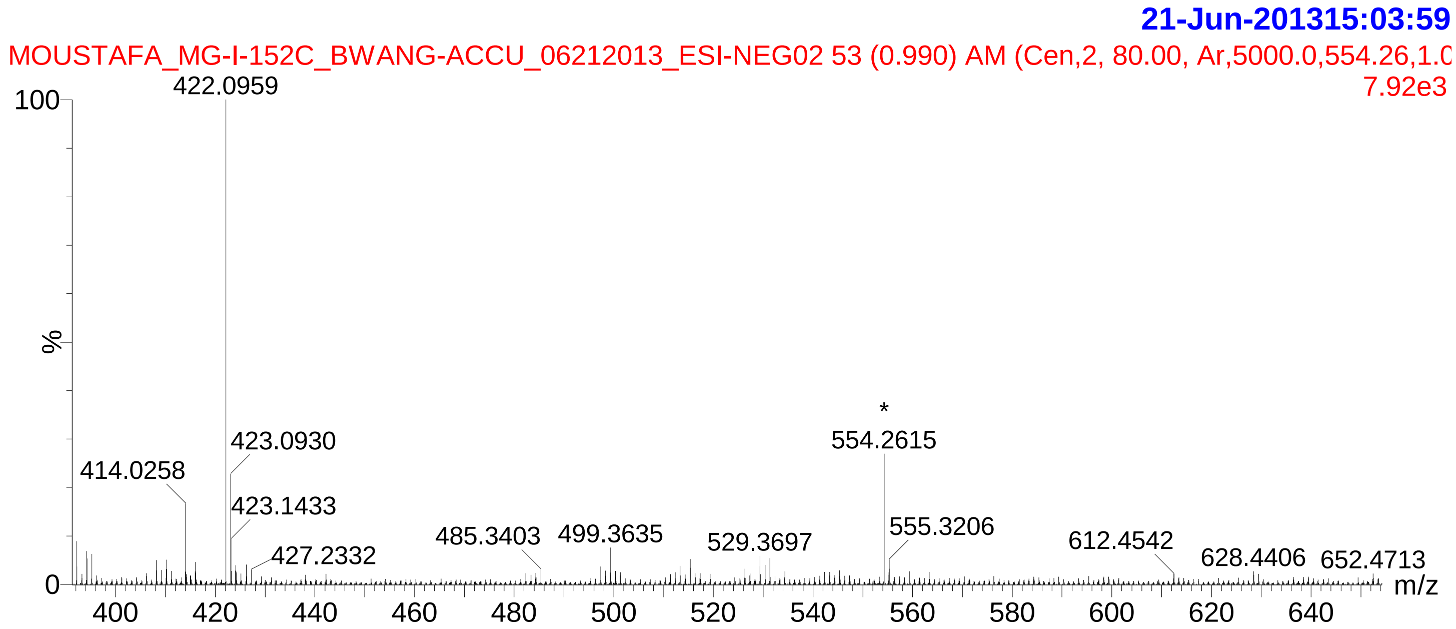

Elemental Composition Report

Single Mass Analysis

Tolerance = 5.0 PPM / DBE: min = -1.5, max = 100.0

Element prediction: Off
Number of isotope peaks used for i-FIT = 3

Monoisotopic Mass, Odd and Even Electron Ions
1556 formula(e) evaluated with 19 results within limits (all results (up to 1000) for each mass)
Elements Used:
C: 1-150 H: 1-150 N: 1-30 O: 1-60 S: 1-2

Minimum:				-1.5		
Maximum:		5.0	5.0	100.0		
Mass	Calc. Mass	mDa	PPM	DBE	i-FIT	Formula
422.0959	422.0955	0.4	0.9	7.5	187.9	C9 H16 N11 O7 S
	422.0942	1.7	4.0	2.5	290.0	C8 H20 N7 O11 S
	422.0955	0.4	0.9	13.0	116.6	C8 H10 N18 O2 S
	422.0941	1.8	4.3	8.0	200.0	C7 H14 N14 O6 S
	422.0941	1.8	4.3	13.5	128.1	C6 H8 N21 O S
	422.0975	-1.6	-3.8	3.0	630.3	C4 H18 N14 O6 S2
	422.0975	-1.6	-3.8	8.5	499.4	C3 H12 N21 O S2
	422.0963	-0.4	-0.9	19.5	253.7	C25 H16 N3 O2 S
	422.0950	0.9	2.1	20.0	205.7	C23 H14 N6 O S
	422.0962	-0.3	-0.7	3.5	643.9	C2 H16 N17 O5 S2
	422.0970	-1.1	-2.6	10.0	561.7	C19 H22 N2 O5 S2
	422.0957	0.2	0.5	10.5	522.1	C17 H20 N5 O4 S2
	422.0943	1.6	3.8	5.5	586.3	C16 H24 N O8 S2
	422.0943	1.6	3.8	11.0	487.3	C15 H18 N8 O3 S2
	422.0968	-0.9	-2.1	1.5	271.4	C12 H24 N O13 S
	422.0968	-0.9	-2.1	7.0	183.9	C11 H18 N8 O8 S
	422.0955	0.4	0.9	2.0	276.8	C10 H22 N4 O12 S
	422.0968	-0.9	-2.1	12.5	113.1	C10 H12 N15 O3 S
	422.0948	1.1	2.6	-1.5	812.3	C H20 N13 O9 S2

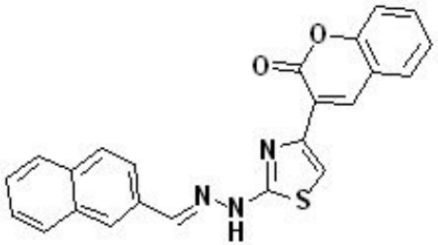

5o

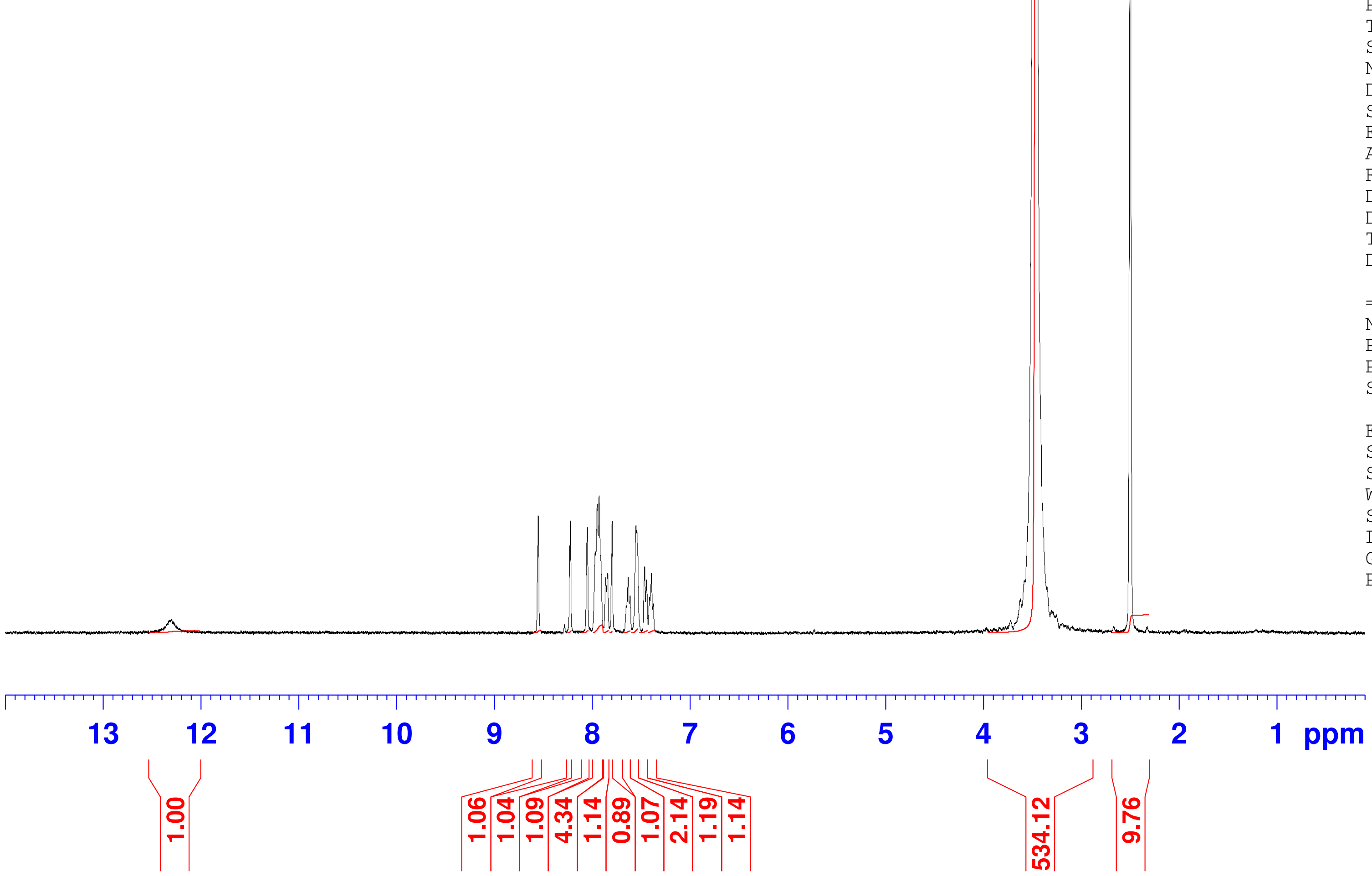

BRUKER

Current Data Parameters
NAME MG151
EXPNO 1
PROCNO 1

F2 - Acquisition Parameters
Date_ 20130417
Time 18.22
INSTRUM spect
PROBHD 5 mm PABBO BB-
PULPROG zg30
TD 65536
SOLVENT DMSO
NS 16
DS 2
SWH 8223.685 Hz
FIDRES 0.125483 Hz
AQ 3.9846387 sec
RG 64
DW 60.800 usec
DE 6.50 usec
TE 294.4 K
D1 1.00000000 sec

======== CHANNEL f1 ========
NUC1 1H
P1 13.50 usec
PLW1 16.00000000 W
SFO1 400.1424710 MHz

F2 - Processing parameters
SI 65536
SF 400.1400000 MHz
WDW EM
SSB 0
LB 0.30 Hz
GB 0
PC 1.00

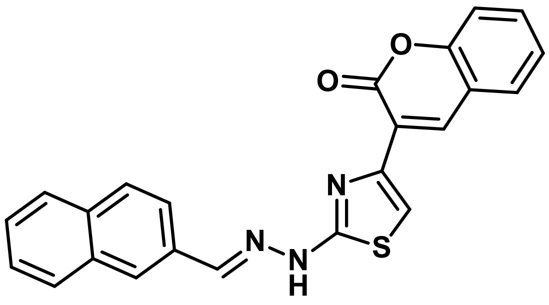

5o

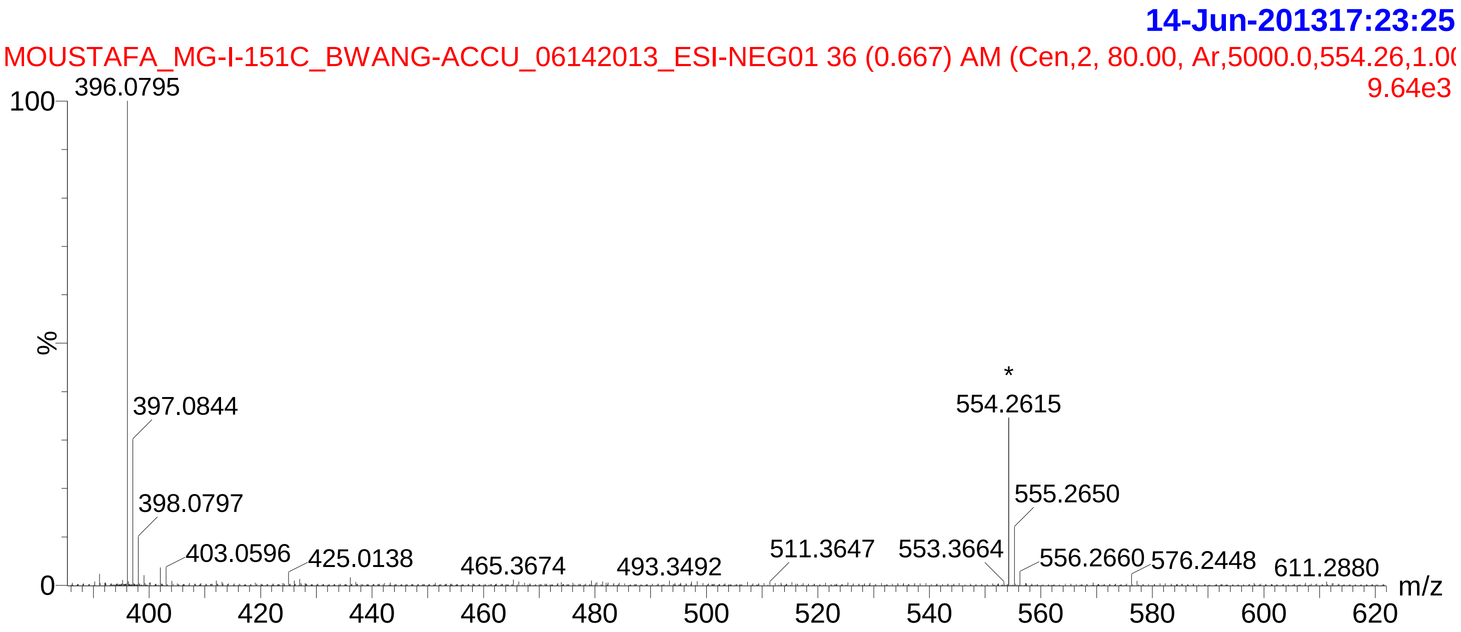

Elemental Composition Report

Single Mass Analysis

Tolerance = 5.0 PPM / DBE: min = -1.5, max = 100.0
Element prediction: Off
Number of isotope peaks used for i-FIT = 3

Monoisotopic Mass, Even Electron Ions
1275 formula(e) evaluated with 8 results within limits (all results (up to 1000) for each mass)
Elements Used:
C: 1-150 H: 1-150 N: 1-30 O: 1-60 S: 1-2

Minimum:				-1.5		
Maximum:		5.0	5.0	100.0		
Mass	Calc. Mass	mDa	PPM	DBE	i-FIT	Formula
396.0795	396.0812	-1.7	-4.3	0.5	452.2	C10 H22 N O13 S
	396.0787	0.8	2.0	4.5	239.5	C14 H22 N O8 S2
	396.0800	-0.5	-1.3	9.5	152.3	C15 H18 N5 O4 S2
	396.0807	-1.2	-3.0	18.5	16.0	C23 H14 N3 O2 S
	396.0785	1.0	2.5	12.5	526.4	C4 H6 N21 O S
	396.0785	1.0	2.5	1.5	608.3	C6 H18 N7 O11 S
	396.0798	-0.3	-0.8	6.5	480.4	C7 H14 N11 O7 S
	396.0812	-1.7	-4.3	11.5	375.1	C8 H10 N15 O3 S

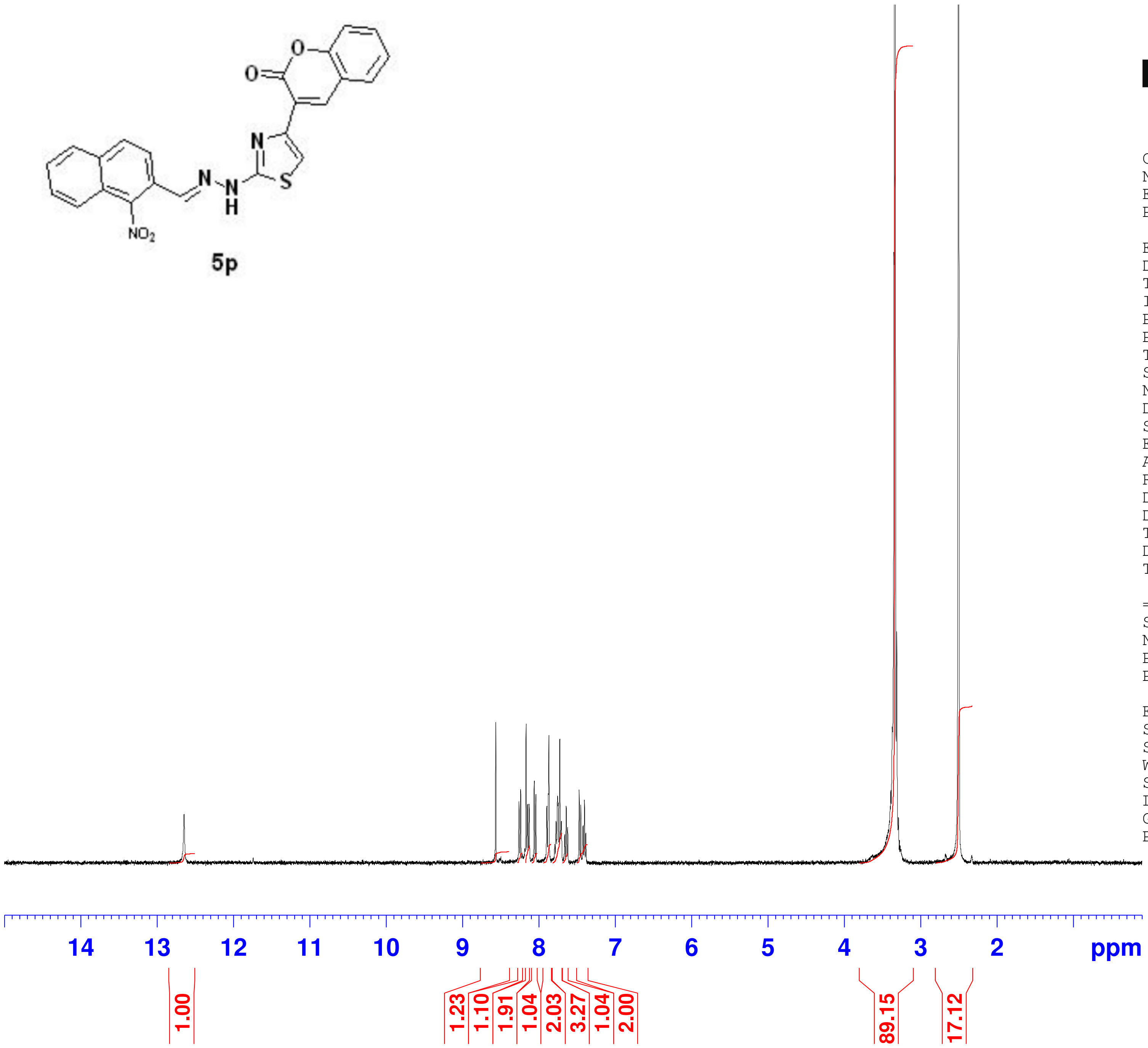


```
Current Data Parameters
NAME                  MG122p
EXPNO                      1
PROCNO                     1

F2 - Acquisition Parameters
Date_               20130328
Time                   18.22
INSTRUM                spect
PROBHD    5 mm PABBO BB-
PULPROG                 zg30
TD                     65536
SOLVENT                 DMSO
NS                         4
DS                         2
SWH                 8012.820 Hz
FIDRES              0.122266 Hz
AQ                 4.0894465 sec
RG                       203
DW                    62.400 usec
DE                      6.50 usec
TE                     298.0 K
D1                1.00000000 sec
TD0                        1

======== CHANNEL f1 ========
SFO1             400.1424710 MHz
NUC1                      1H
P1                     13.50 usec
PLW1             16.00000000 W

F2 - Processing parameters
SI                     65536
SF               400.1400000 MHz
WDW                       EM
SSB      0
LB                      0.30 Hz
GB       0
PC                      1.40
```

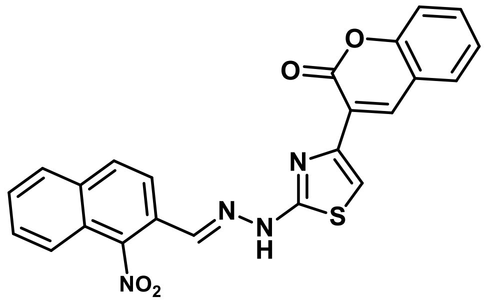

5p

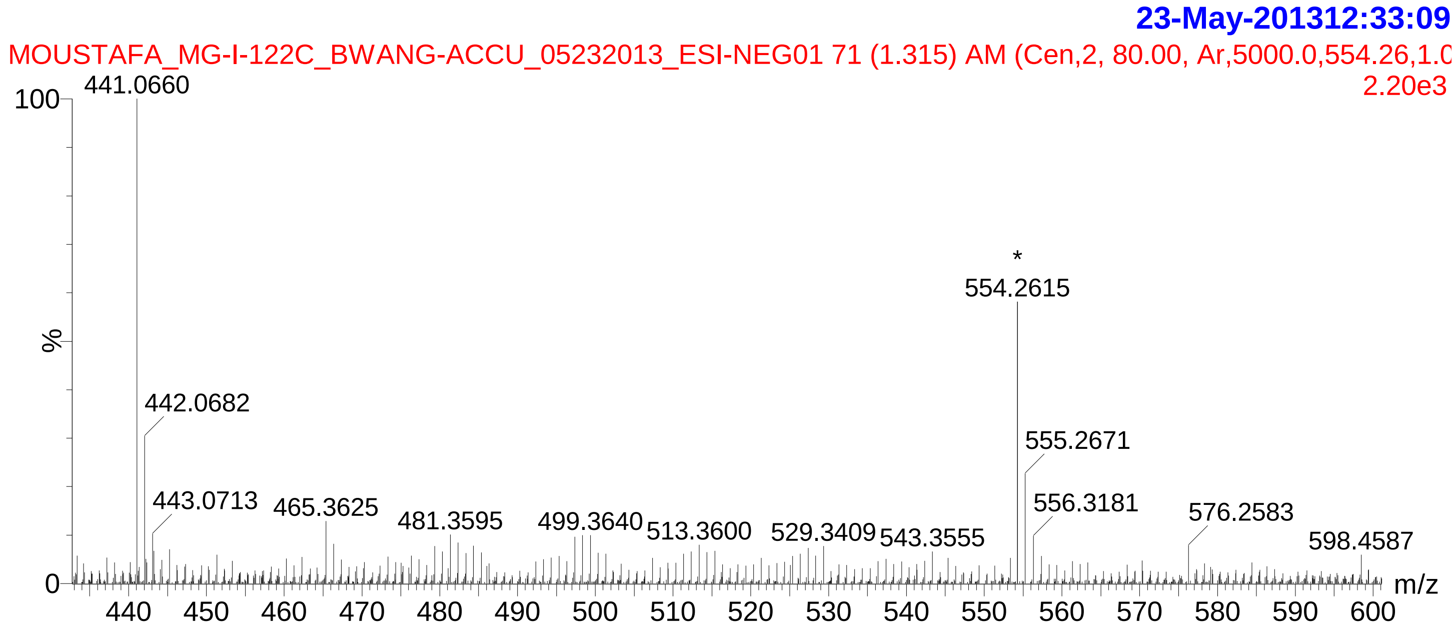

Elemental Composition Report

Single Mass Analysis
Tolerance = 5.0 PPM / DBE: min = -1.5, max = 100.0

Element prediction: Off

Monoisotopic Mass, Even Electron Ions
1035 formula(e) evaluated with 6 results within limits (all results (up to 1000) for each mass)
Elements Used:
C: 1-150   H: 1-150   N: 1-30   O: 1-60   S: 1-1

Minimum:				-1.5	
Maximum:		5.0	5.0	100.0	
Mass	Calc. Mass	mDa	PPM	DBE	Formula
441.0660	441.0663	-0.3	-0.7	1.5	C10 H21 N2 O15 S
	441.0676	-1.6	-3.6	6.5	C11 H17 N6 O11 S
	441.0658	0.2	0.5	19.5	C23 H13 N4 O4 S
	441.0649	1.1	2.5	7.5	C7 H13 N12 O9 S
	441.0663	-0.3	-0.7	12.5	C8 H9 N16 O5 S
	441.0676	-1.6	-3.6	17.5	C9 H5 N20 O S

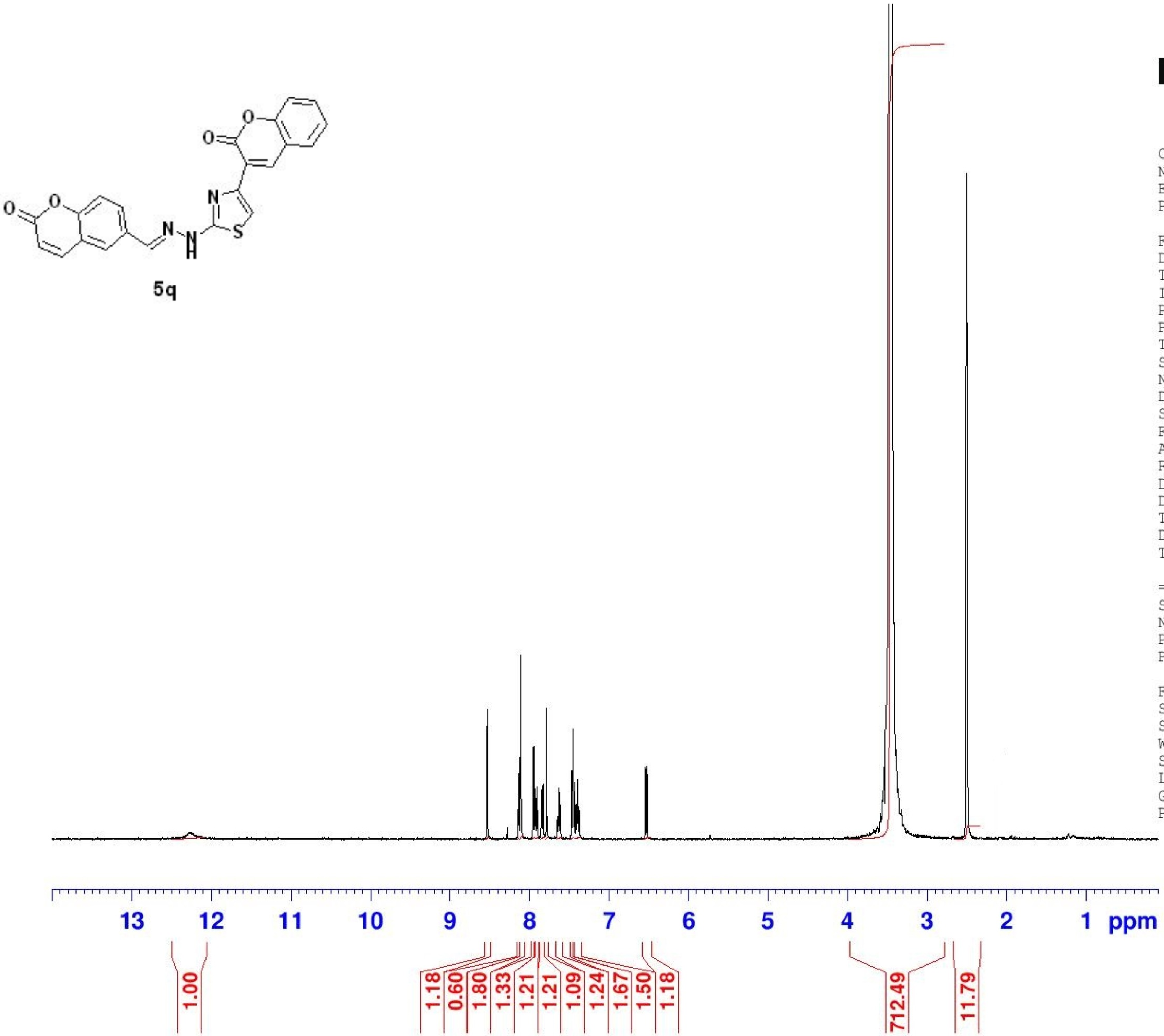

5q

BRUKER

```
Current Data Parameters
NAME                    MG153
EXPNO                       1
PROCNO                      1

F2 - Acquisition Parameters
Date_                20130418
Time                    18.09
INSTRUM                 spect
PROBHD    5 mm PABBO BB-
PULPROG                  zg30
TD                      65536
SOLVENT                  DMSO
NS                         16
DS                          2
SWH                  8012.820 Hz
FIDRES               0.122266 Hz
AQ                  4.0894465 sec
RG                         64
DW                     62.400 usec
DE                       6.50 usec
TE                      298.1 K
D1                 1.00000000 sec
TD0                         1

======== CHANNEL f1 ========
SFO1              400.1424710 MHz
NUC1                       1H
P1                      13.50 usec
PLW1              16.00000000 W

F2 - Processing parameters
SI                      65536
SF                400.1400000 MHz
WDW                        EM
SSB       0
LB                       0.30 Hz
GB        0
PC                       1.40
```

test

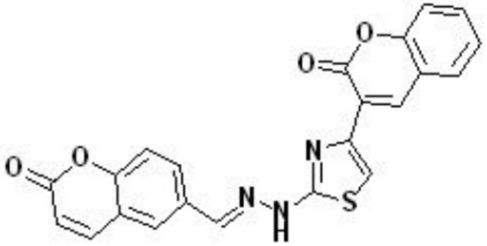

5q

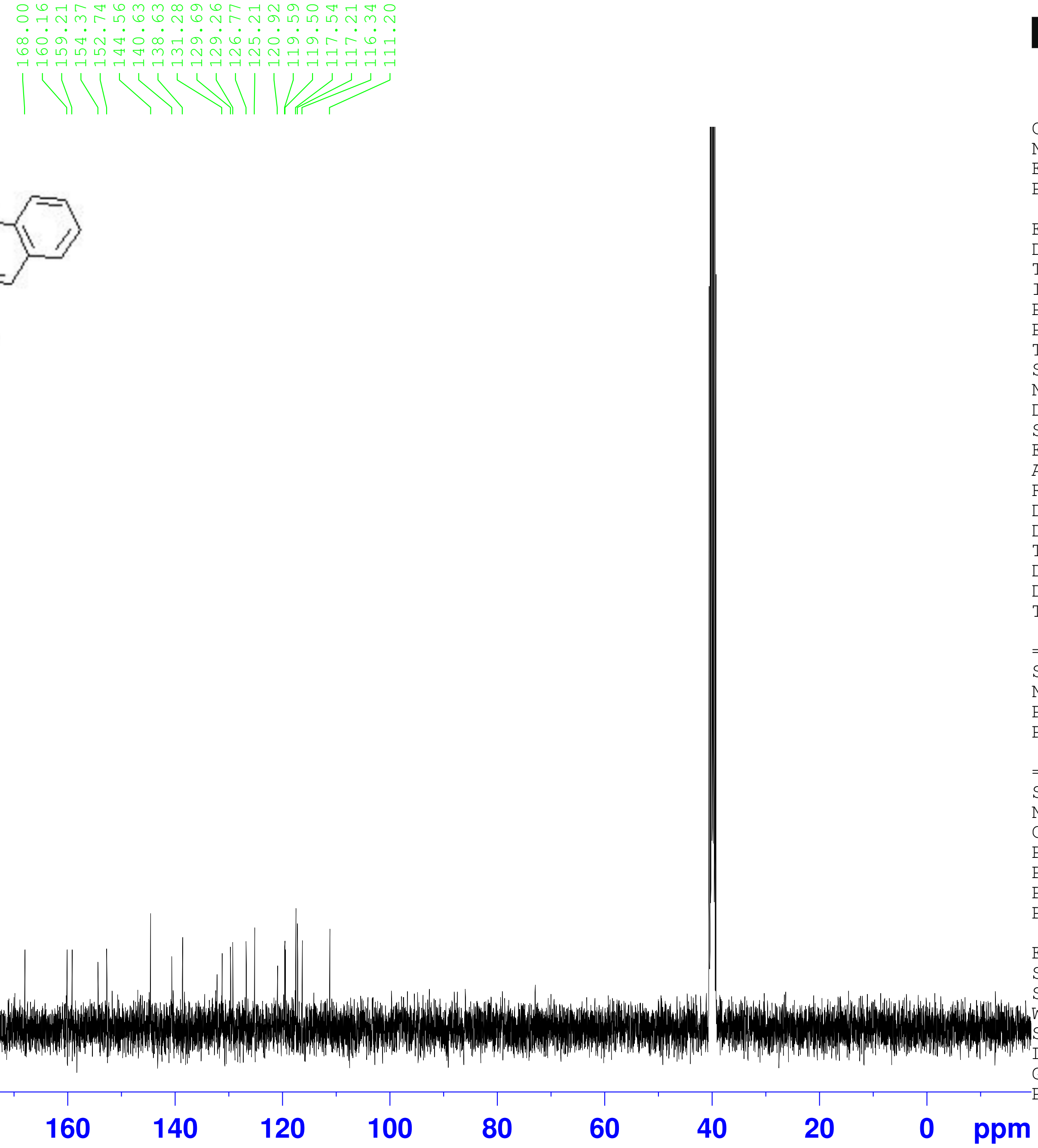

BRUKER

Current Data Parameters
NAME MG153cc
EXPNO 1
PROCNO 1

F2 - Acquisition Parameters
Date_ 20130429
Time 12.17
INSTRUM spect
PROBHD 5 mm PABBO BB-
PULPROG zgpg30
TD 65536
SOLVENT DMSO
NS 215
DS 4
SWH 24038.461 Hz
FIDRES 0.366798 Hz
AQ 1.3631488 sec
RG 114
DW 20.800 usec
DE 6.50 usec
TE 298.3 K
D1 2.00000000 sec
D11 0.03000000 sec
TD0 1

======== CHANNEL f1 ========
SFO1 100.6253441 MHz
NUC1 13C
P1 9.00 usec
PLW1 62.00000000 W

======== CHANNEL f2 ========
SFO2 400.1416006 MHz
NUC2 1H
CPDPRG[2 waltz16
PCPD2 90.00 usec
PLW2 16.00000000 W
PLW12 0.36000001 W
PLW13 0.29159999 W

F2 - Processing parameters
SI 32768
SF 100.6152830 MHz
WDW EM
SSB 0
LB 1.00 Hz
GB 0
PC 1.40

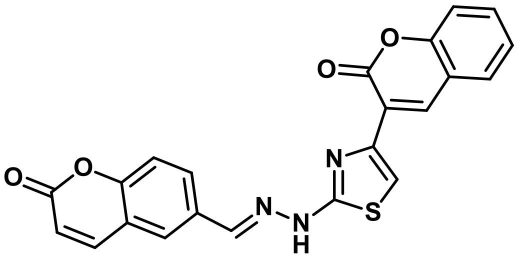

5q

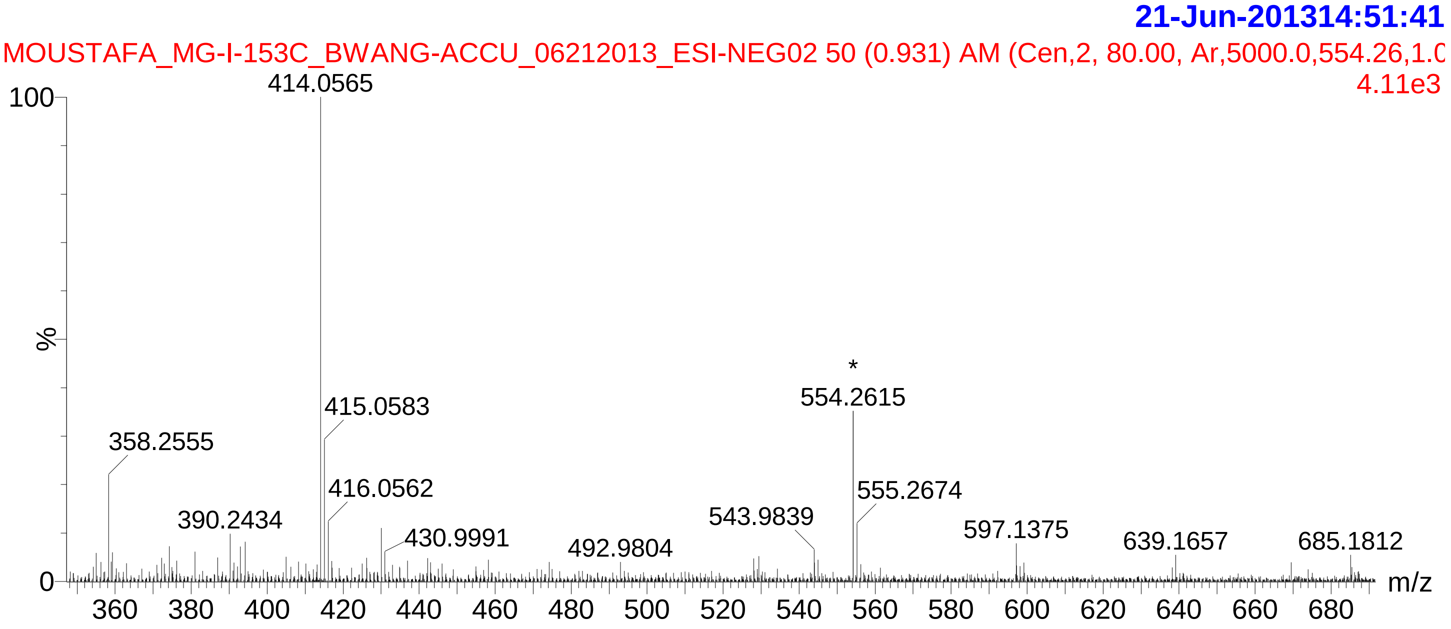

Elemental Composition Report

Single Mass Analysis
Tolerance = 5.0 PPM / DBE: min = -1.5, max = 100.0
Element prediction: Off

Number of isotope peaks used for i-FIT = 3

Monoisotopic Mass, Odd and Even Electron Ions
1476 formula(e) evaluated with 18 results within limits (all results (up to 1000) for each mass)
Elements Used:
C: 1-150 H: 1-150 N: 1-30 O: 1-60 S: 1-2

Minimum:				-1.5		
Maximum:		5.0	5.0	100.0		
Mass	Calc. Mass	mDa	PPM	DBE	i-FIT	Formula
414.0565	414.0560	0.5	1.2	2.0	301.6	C H14 N14 O8 S2
	414.0567	-0.2	-0.5	5.5	173.7	C10 H16 N5 O11 S
	414.0580	-1.5	-3.6	16.0	132.0	C10 H6 N16 O2 S
	414.0580	-1.5	-3.6	10.5	136.1	C11 H12 N9 O7 S
	414.0580	-1.5	-3.6	5.0	145.1	C12 H18 N2 O12 S
	414.0555	1.0	2.4	14.5	33.8	C15 H12 N9 O2 S2
	414.0555	1.0	2.4	9.0	46.0	C16 H18 N2 O7 S2
	414.0569	-0.4	-1.0	14.0	23.3	C17 H14 N6 O3 S2
	414.0582	-1.7	-4.1	13.5	15.7	C19 H16 N3 O4 S2
	414.0574	-0.9	-2.2	7.0	236.0	C2 H10 N18 O4 S2
	414.0549	1.6	3.9	18.5	24.1	C22 H12 N3 O4 S
	414.0575	-1.0	-2.4	23.0	19.1	C25 H10 N4 O S
	414.0574	-0.9	-2.2	1.5	265.5	C3 H16 N11 O9 S2
	414.0554	1.1	2.7	11.5	194.3	C7 H8 N15 O5 S
	414.0554	1.1	2.7	6.0	205.0	C8 H14 N8 O10 S
	414.0567	-0.2	-0.5	16.5	158.8	C8 H4 N19 O S
	414.0567	-0.2	-0.5	11.0	163.7	C9 H10 N12 O6 S
	414.0554	1.1	2.7	0.5	220.5	C9 H20 N O15 S

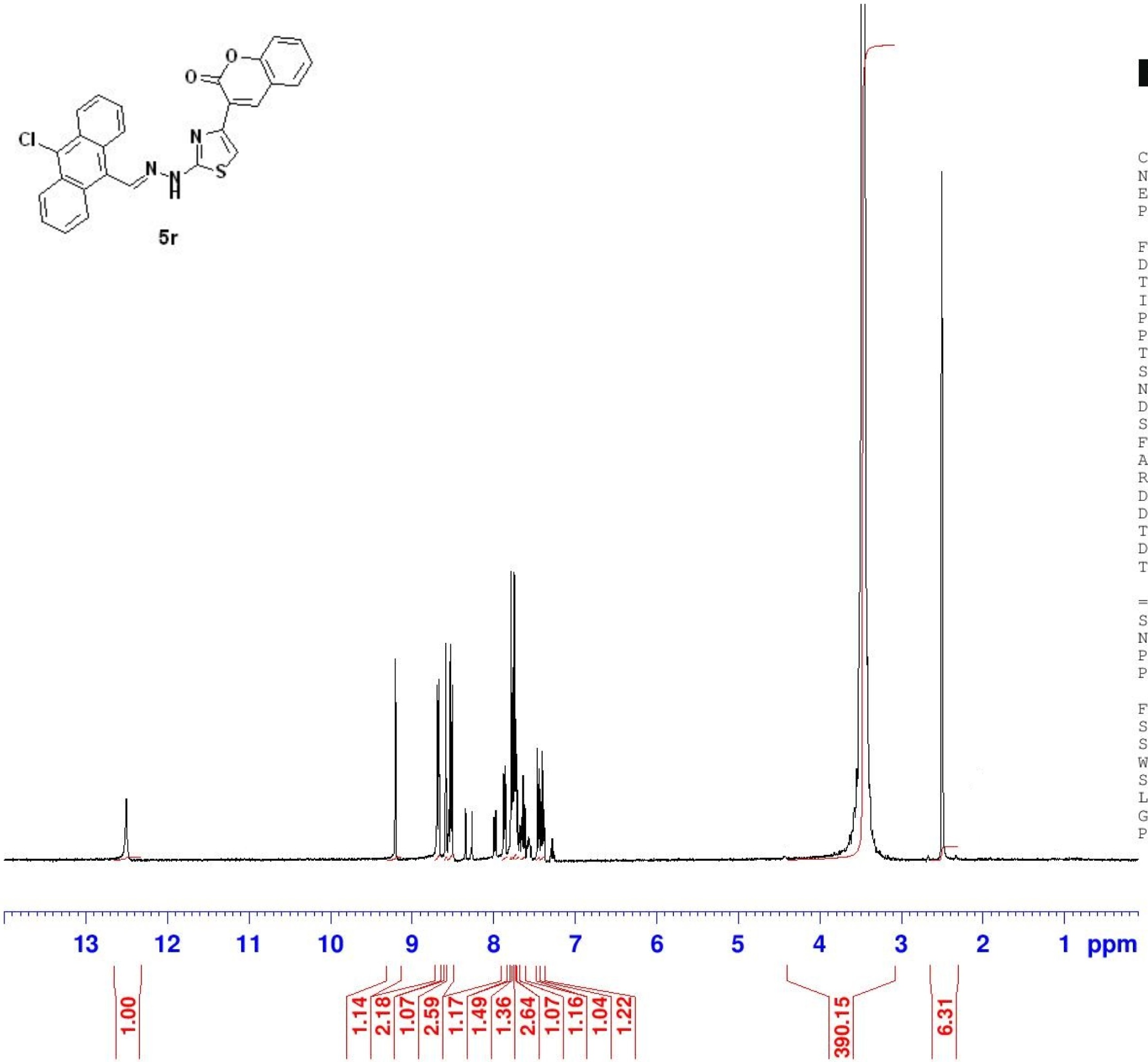

5r


```
Current Data Parameters
NAME              MG155
EXPNO                 1
PROCNO                1

F2 - Acquisition Parameters
Date_          20130418
Time              18.14
INSTRUM           spect
PROBHD   5 mm PABBO BB-
PULPROG            zg30
TD                65536
SOLVENT            DMSO
NS                   16
DS                    2
SWH            8012.820 Hz
FIDRES         0.122266 Hz
AQ            4.0894465 sec
RG                   64
DW               62.400 usec
DE                 6.50 usec
TE                298.1 K
D1           1.00000000 sec
TD0                   1

======== CHANNEL f1 ========
SFO1        400.1424710 MHz
NUC1                 1H
P1                13.50 usec
PLW1        16.00000000 W

F2 - Processing parameters
SI                65536
SF          400.1400000 MHz
WDW                  EM
SSB      0
LB                 0.30 Hz
GB       0
PC                 1.40
```

test

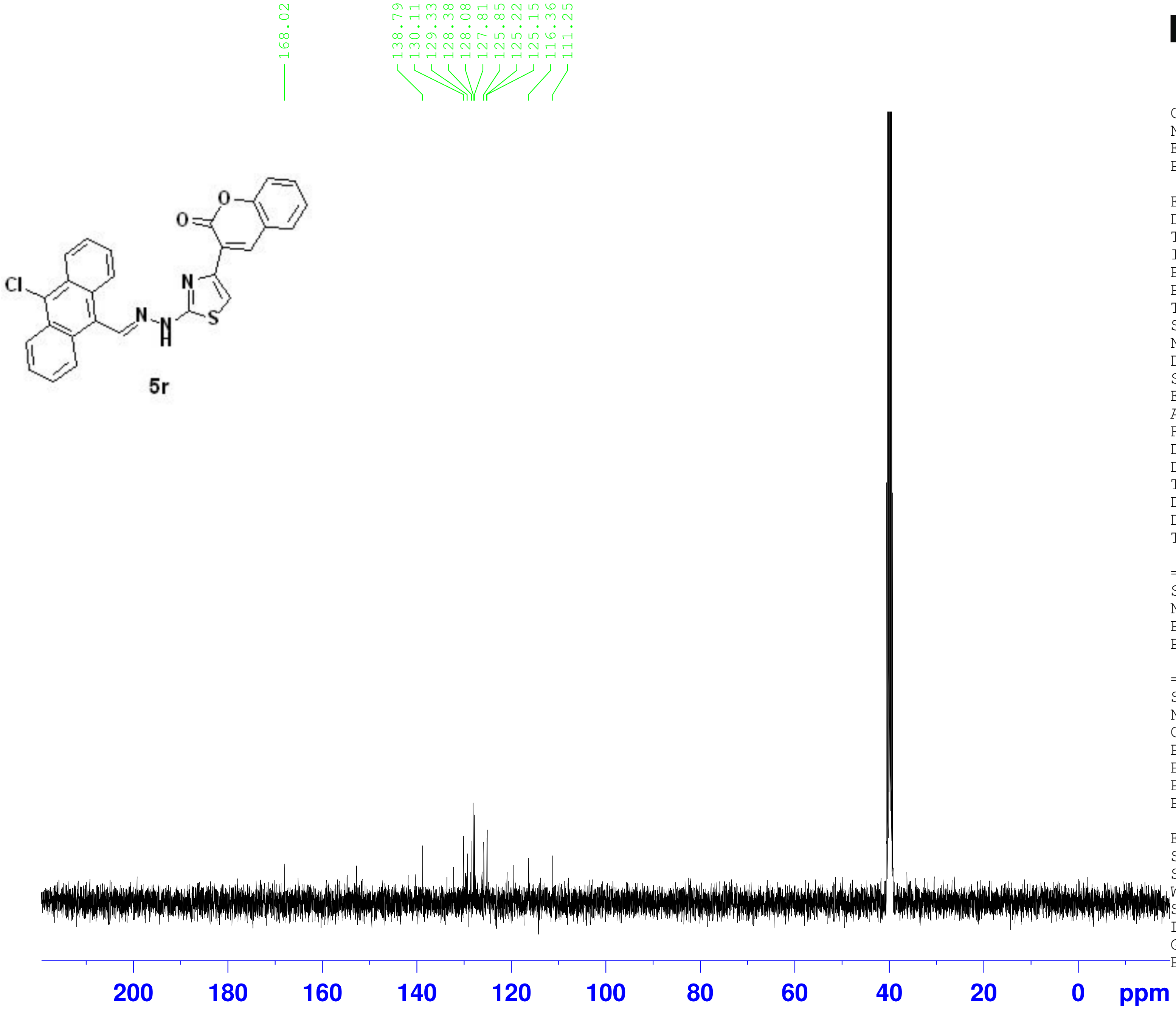

Current Data Parameters

NAME	MG155c
EXPNO	1
PROCNO	1

F2 - Acquisition Parameters

Date_	20130422
Time	17.55
INSTRUM	spect
PROBHD	5 mm PABBO BB-
PULPROG	zgpg30
TD	65536
SOLVENT	DMSO
NS	164
DS	4
SWH	24038.461 Hz
FIDRES	0.366798 Hz
AQ	1.3631488 sec
RG	181
DW	20.800 usec
DE	6.50 usec
TE	297.9 K
D1	2.00000000 sec
D11	0.03000000 sec
TD0	1

======== CHANNEL f1 ========

SFO1	100.6253441 MHz
NUC1	13C
P1	9.00 usec
PLW1	62.00000000 W

======== CHANNEL f2 ========

SFO2	400.1416006 MHz
NUC2	1H
CPDPRG[2	waltz16
PCPD2	90.00 usec
PLW2	16.00000000 W
PLW12	0.36000001 W
PLW13	0.29159999 W

F2 - Processing parameters

SI	32768
SF	100.6152830 MHz
WDW	EM
SSB	0
LB	1.00 Hz
GB	0
PC	1.40

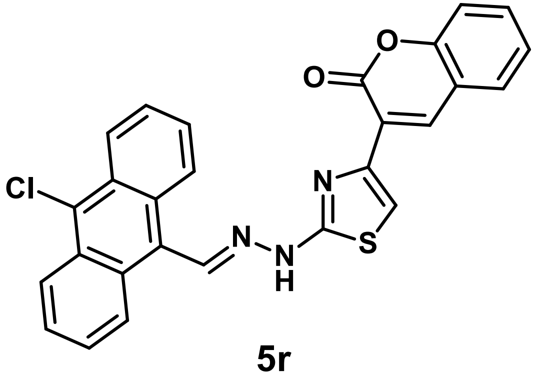

5r

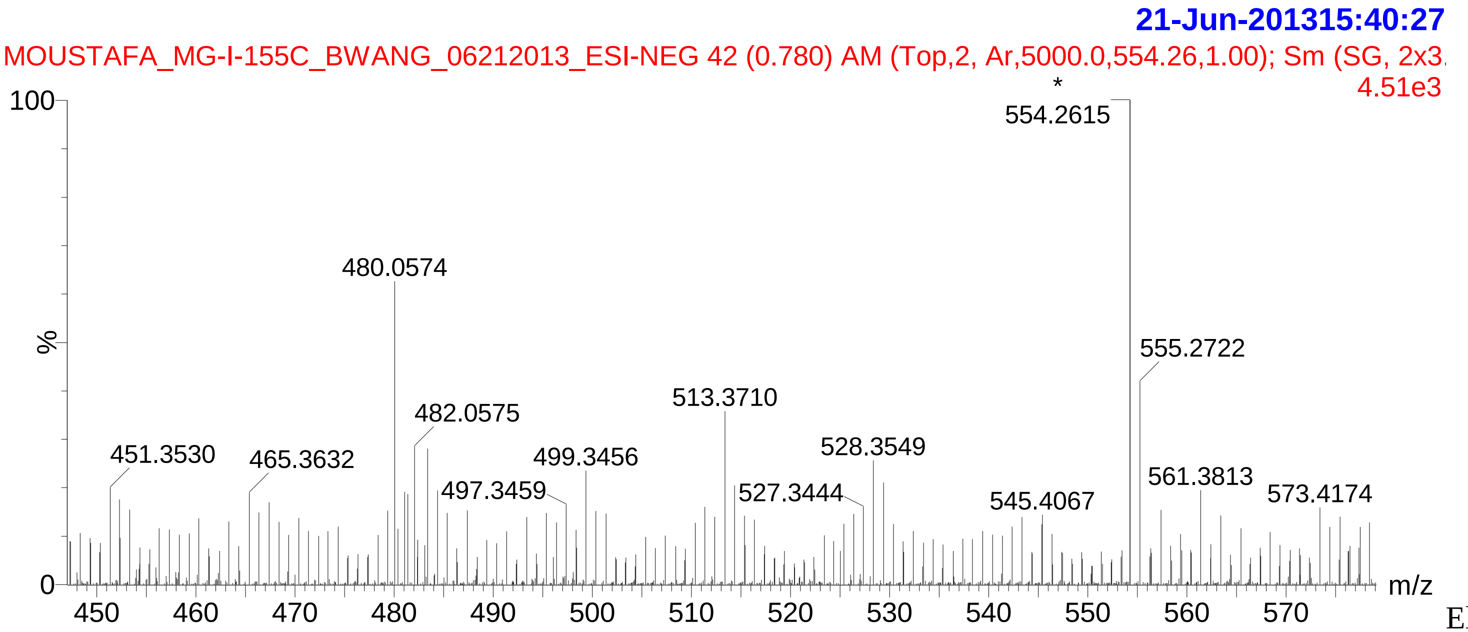

Elemental Composition Report

Single Mass Analysis

Tolerance = 5.0 PPM / DBE: min = -1.5, max = 100.0
Element prediction: Off
Number of isotope peaks used for i-FIT = 3

Monoisotopic Mass, Odd and Even Electron Ions
3218 formula(e) evaluated with 44 results within limits (all results (up to 1000) for each mass)
Elements Used:
C: 1-150 H: 1-150 N: 1-30 O: 1-60 S: 1-2 Cl: 1-2

Minimum:				-1.5		
Maximum:		5.0	5.0	100.0		
Mass	Calc. Mass	mDa	PPM	DBE	i-FIT	Formula
480.0574	480.0574	0.0	0.0	21.5	6.3	C27 H15 N3 O2 S Cl
	480.0572	0.2	0.4	5.5	141.0	C4 H15 N17 O5 S2 Cl
	480.0572	0.2	0.4	0.0	161.3	C5 H21 N10 O10 S2 Cl
	480.0572	0.2	0.4	8.0	202.9	C16 H22 N6 O3 S2 Cl2
	480.0577	-0.3	-0.6	1.0	353.2	C H18 N18 O4 S2 Cl2
	480.0570	0.4	0.8	10.5	226.4	C7 H12 N19 O S Cl2
	480.0570	0.4	0.8	-0.5	272.6	C9 H24 N5 O11 S Cl2
	480.0570	0.4	0.8	5.0	248.8	C8 H18 N12 O6 S Cl2
	480.0578	-0.4	-0.8	17.0	126.9	C24 H18 N4 O S Cl2
	480.0579	-0.5	-1.0	14.5	51.1	C12 H11 N15 O3 S Cl
	480.0579	-0.5	-1.0	3.5	72.2	C14 H23 N O13 S Cl
	480.0579	-0.5	-1.0	9.0	60.7	C13 H17 N8 O8 S Cl
	480.0580	-0.6	-1.2	12.0	7.9	C21 H21 N2 O5 S2 Cl
	480.0567	0.7	1.5	12.5	12.9	C19 H19 N5 O4 S2 Cl
	480.0565	0.9	1.9	9.5	74.8	C11 H15 N11 O7 S Cl
	480.0583	-0.9	-1.9	4.5	234.9	C10 H20 N9 O7 S Cl2
	480.0565	0.9	1.9	4.0	87.6	C12 H21 N4 O12 S Cl
	480.0583	-0.9	-1.9	10.0	213.3	C9 H14 N16 O2 S Cl2
	480.0565	0.9	1.9	15.0	63.9	C10 H9 N18 O2 S Cl
	480.0584	-1.0	-2.1	-1.0	257.5	C11 H26 N2 O12 S Cl2

480.0585	-1.1	-2.3	7.5	195.5	C18 H24 N3 O4 S2 Cl2
480.0585	-1.1	-2.3	10.5	103.9	C5 H11 N21 O S2 Cl
480.0585	-1.1	-2.3	5.0	121.3	C6 H17 N14 O6 S2 Cl
480.0586	-1.2	-2.5	-0.5	140.4	C7 H23 N7 O11 S2 Cl
480.0560	1.4	2.9	22.0	6.2	C25 H13 N6 O S Cl
480.0559	1.5	3.1	3.0	230.2	C15 H26 N2 O7 S2 Cl2
480.0559	1.5	3.1	0.5	184.2	C3 H19 N13 O9 S2 Cl
480.0559	1.5	3.1	6.0	162.6	C2 H13 N20 O4 S2 Cl
480.0590	-1.6	-3.3	0.5	335.3	C3 H20 N15 O5 S2 Cl2
480.0558	1.6	3.3	8.5	211.4	C14 H20 N9 O2 S2 Cl2
480.0557	1.7	3.5	0.0	288.8	C7 H22 N8 O10 S Cl2
480.0557	1.7	3.5	5.5	264.3	C6 H16 N15 O5 S Cl2
480.0592	-1.8	-3.7	8.5	48.4	C15 H19 N5 O9 S Cl
480.0592	-1.8	-3.7	14.0	40.1	C14 H13 N12 O4 S Cl
480.0592	-1.8	-3.7	16.5	125.6	C26 H20 N O2 S Cl2
480.0554	2.0	4.2	7.5	26.7	C18 H23 N O8 S2 Cl
480.0594	-2.0	-4.2	17.0	2.0	C22 H17 N6 O S2 Cl
480.0554	2.0	4.2	13.0	19.4	C17 H17 N8 O3 S2 Cl
480.0552	2.2	4.6	4.5	104.8	C10 H19 N7 O11 S Cl
480.0552	2.2	4.6	12.5	146.0	C21 H20 N3 O4 S Cl2
480.0552	2.2	4.6	15.5	78.6	C8 H7 N21 O S Cl
480.0552	2.2	4.6	10.0	90.8	C9 H13 N14 O6 S Cl
480.0597	-2.3	-4.8	4.0	222.3	C12 H22 N6 O8 S Cl2
480.0597	-2.3	-4.8	9.5	201.6	C11 H16 N13 O3 S Cl2

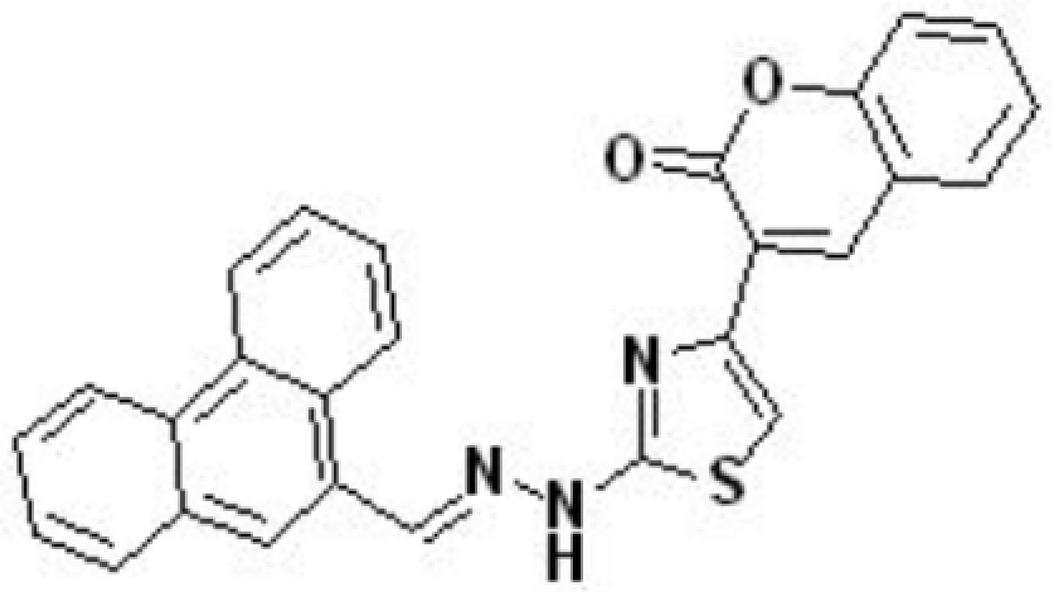

5s

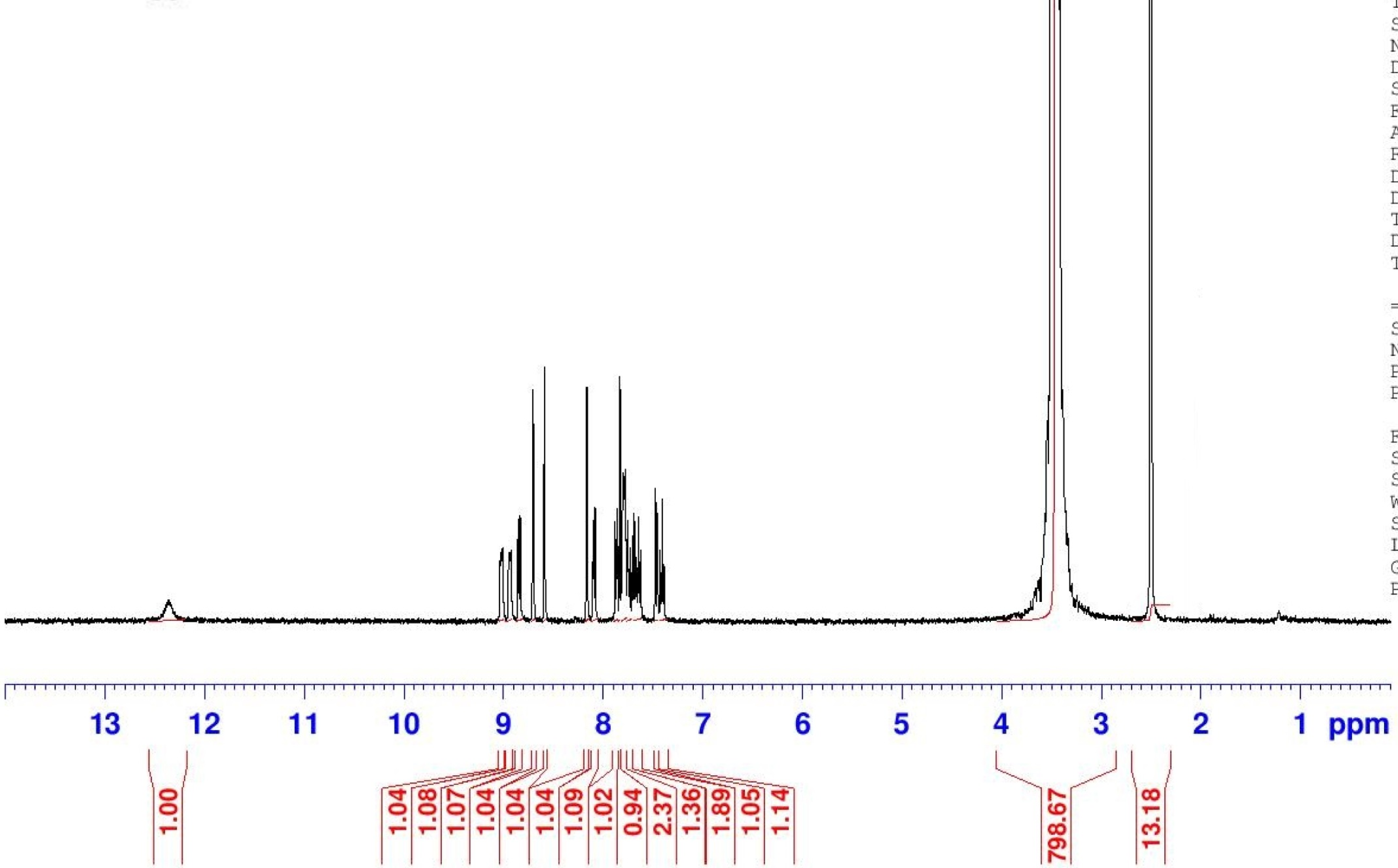

Current Data Parameters
NAME MG154
EXPNO 1
PROCNO 1

F2 - Acquisition Parameters
Date_ 20130418
Time 18.04
INSTRUM spect
PROBHD 5 mm PABBO BB-
PULPROG zg30
TD 65536
SOLVENT DMSO
NS 16
DS 2
SWH 8012.820 Hz
FIDRES 0.122266 Hz
AQ 4.0894465 sec
RG 64
DW 62.400 usec
DE 6.50 usec
TE 298.1 K
D1 1.00000000 sec
TD0 1

======== CHANNEL f1 ========
SFO1 400.1424710 MHz
NUC1 1H
P1 13.50 usec
PLW1 16.00000000 W

F2 - Processing parameters
SI 65536
SF 400.1400000 MHz
WDW EM
SSB 0
LB 0.30 Hz
GB 0
PC 1.40

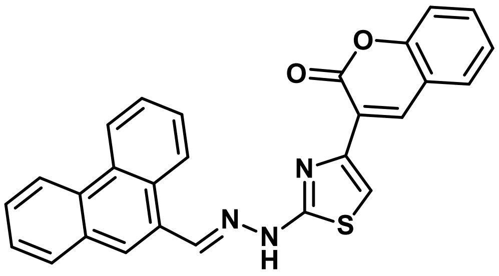

5s

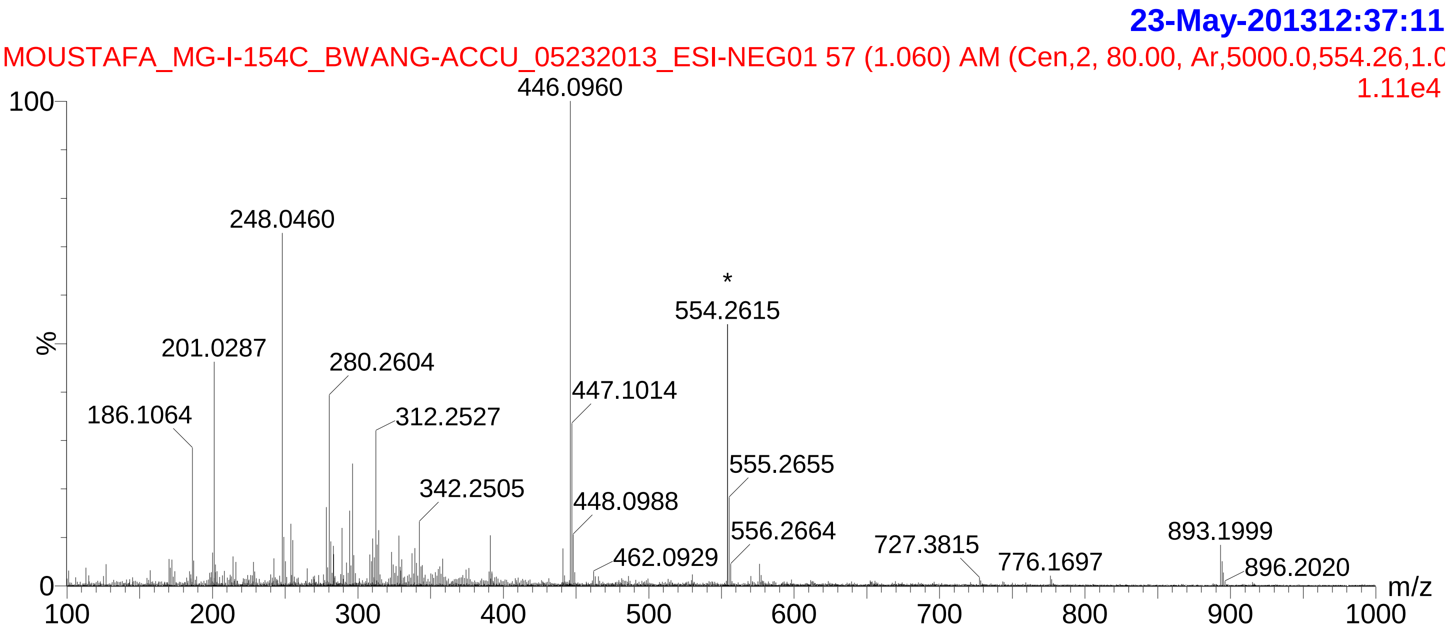

Elemental Composition Report

Single Mass Analysis
Tolerance = 5.0 PPM / DBE: min = -1.5, max = 100.0

Element prediction: Off
Number of isotope peaks used for i-FIT = 3

Monoisotopic Mass, Even Electron Ions
1042 formula(e) evaluated with 7 results within limits (all results (up to 1000) for each mass)
Elements Used:
C: 1-150   H: 1-150   N: 1-30   O: 1-60   S: 1-1

Minimum:				-1.5		
Maximum:		5.0	5.0	100.0		
Mass	Calc. Mass	mDa	PPM	DBE	i-FIT	Formula
446.0960	446.0941	1.9	4.3	15.5	470.5	C8 H8 N21 O S
	446.0963	-0.3	-0.7	21.5	4.4	C27 H16 N3 O2 S
	446.0982	-2.2	-4.9	8.5	289.7	C15 H20 N5 O9 S
	446.0968	-0.8	-1.8	3.5	394.5	C14 H24 N O13 S
	446.0968	-0.8	-1.8	14.5	323.1	C12 H12 N15 O3 S
	446.0955	0.5	1.1	9.5	422.9	C11 H16 N11 O7 S
	446.0942	1.8	4.0	4.5	546.0	C10 H20 N7 O11 S

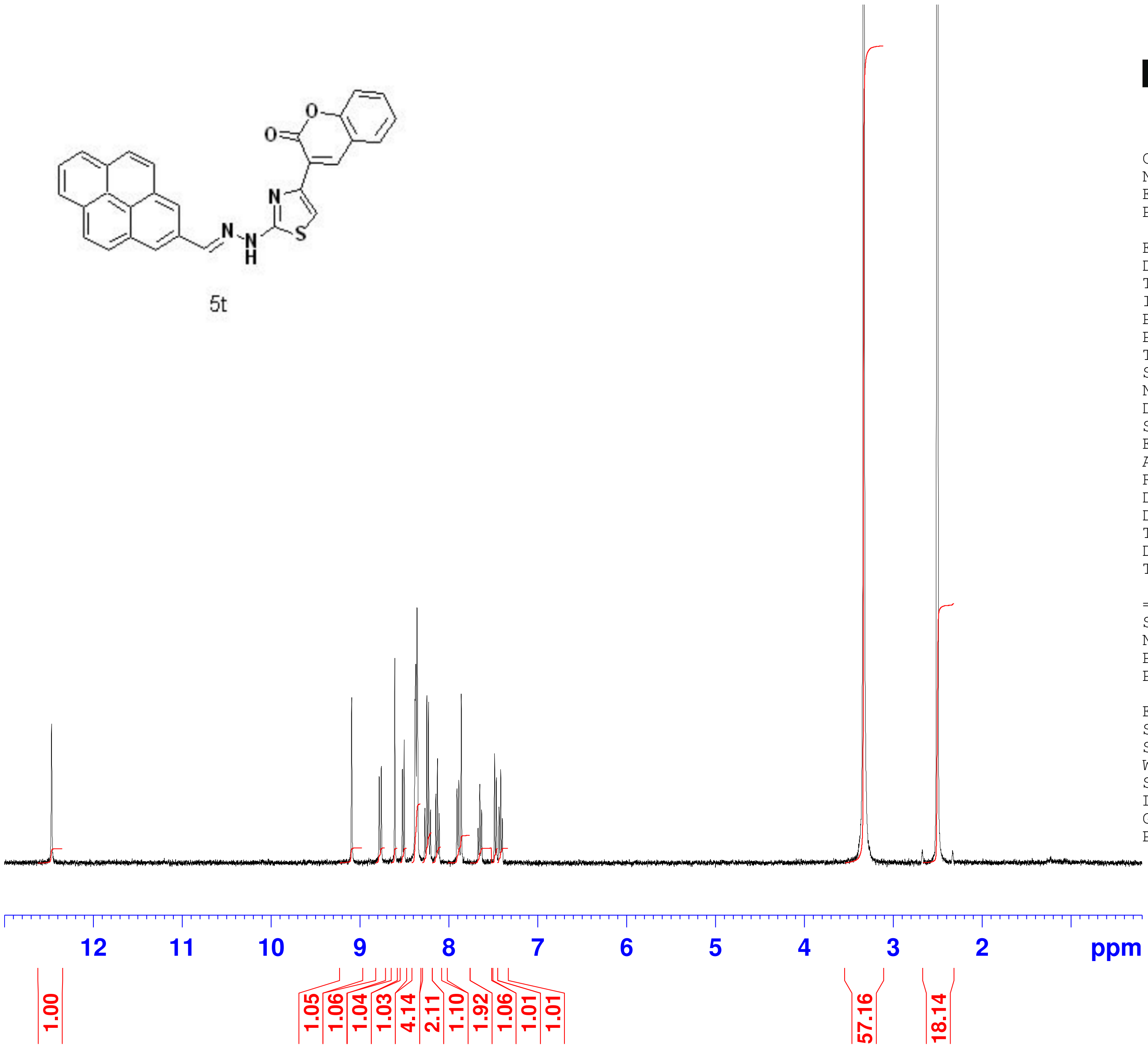

5t

Current Data Parameters

NAME	MG156
EXPNO	1
PROCNO	1

F2 - Acquisition Parameters

Date_	20130419
Time	18.11
INSTRUM	spect
PROBHD	5 mm PABBO BB-
PULPROG	zg30
TD	65536
SOLVENT	DMSO
NS	5
DS	2
SWH	8012.820 Hz
FIDRES	0.122266 Hz
AQ	4.0894465 sec
RG	203
DW	62.400 usec
DE	6.50 usec
TE	297.9 K
D1	1.00000000 sec
TD0	1

======== CHANNEL f1 ========

SFO1	400.1424710 MHz
NUC1	1H
P1	13.50 usec
PLW1	16.00000000 W

F2 - Processing parameters

SI	65536
SF	400.1400000 MHz
WDW	EM
SSB	0
LB	0.30 Hz
GB	0
PC	1.40

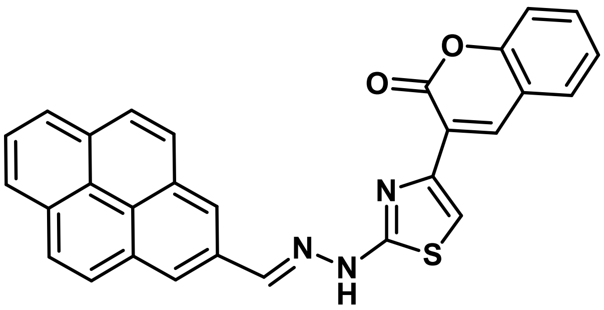

5t

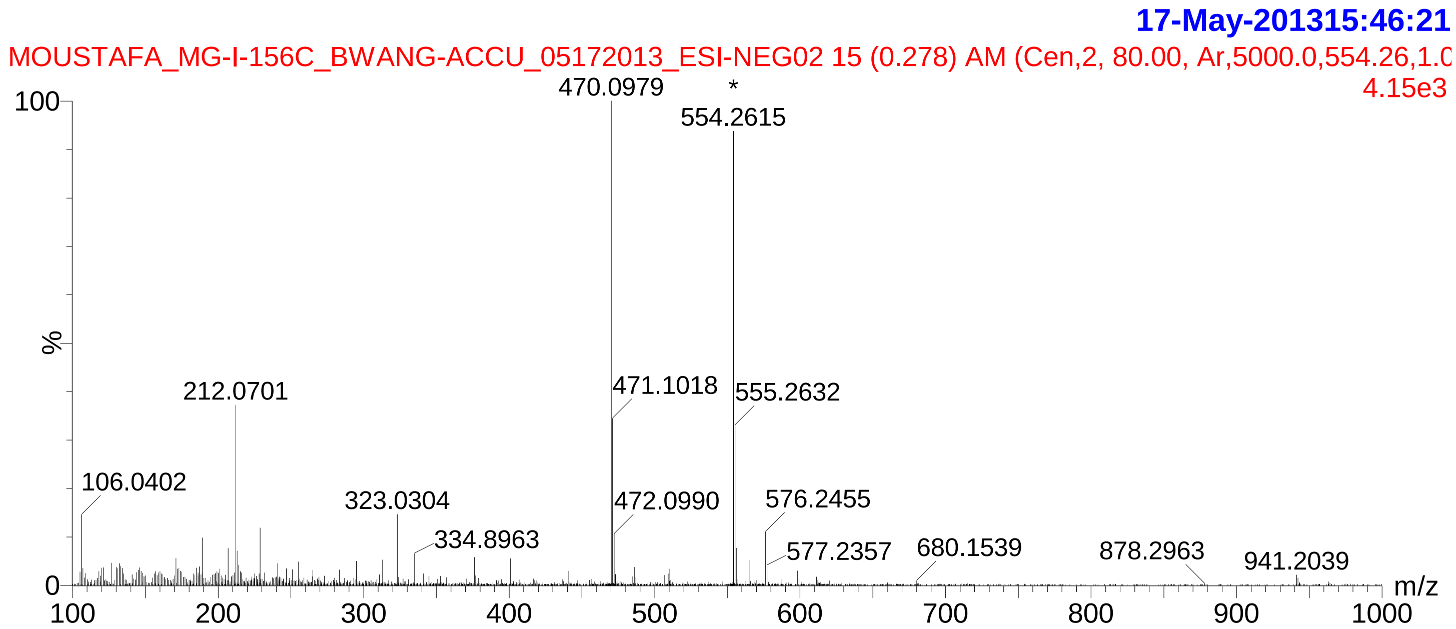

Elemental Composition Report

Single Mass Analysis

Tolerance = 5.0 PPM / DBE: min = -1.5, max = 50.0
Element prediction: Off
Number of isotope peaks used for i-FIT = 3

Monoisotopic Mass, Odd and Even Electron Ions
3573 formula(e) evaluated with 42 results within limits (all results (up to 1000) for each mass)
Elements Used:
C: 1-150 H: 1-150 N: 1-30 O: 1-60 S: 1-4

Minimum:				-1.5		
Maximum:		5.0	5.0	50.0		
Mass	Calc. Mass	mDa	PPM	DBE	i-FIT	Formula
470.0979	470.0975	0.4	0.9	1.5	225.8	C9 H24 N7 O11 S2
	470.0984	-0.5	-1.1	6.0	299.3	C9 H22 N14 O S4
	470.0989	-1.0	-2.1	12.0	141.8	C9 H14 N18 O2 S2
	470.0971	0.8	1.7	1.0	359.2	C8 H26 N10 O5 S4
	470.0975	0.4	0.9	7.0	192.6	C8 H18 N14 O6 S2
	470.0962	1.7	3.6	2.0	252.4	C7 H22 N10 O10 S2
	470.0975	0.4	0.9	12.5	164.0	C7 H12 N21 O S2
	470.0957	2.2	4.7	1.5	375.6	C6 H24 N13 O4 S4
	470.0962	1.7	3.6	7.5	218.6	C6 H16 N17 O5 S2
	470.1000	-2.1	-4.5	3.0	332.8	C4 H18 N14 O11 S
	470.0995	-1.6	-3.4	2.5	329.9	C3 H20 N17 O5 S3
	470.1000	-2.1	-4.5	8.5	310.9	C3 H12 N21 O6 S
	470.0963	1.6	3.4	23.5	0.2	C29 H16 N3 O2 S
	470.0997	-1.8	-3.8	18.5	28.5	C26 H20 N3 O2 S2
	470.0984	-0.5	-1.1	19.0	26.3	C24 H18 N6 O S2
	470.0970	0.9	1.9	14.0	41.4	C23 H22 N2 O5 S2
	470.0957	2.2	4.7	14.5	44.7	C21 H20 N5 O4 S2
	470.1000	-2.1	-4.5	14.0	294.2	C2 H6 N28 O S
	470.0987	-0.8	-1.7	3.5	372.7	C2 H16 N17 O10 S
	470.0995	-1.6	-3.4	10.0	65.3	C19 H22 N2 O10 S

470.0990	-1.1	-2.3	9.5	136.3	C18 H24 N5 O4 S3
470.0995	-1.6	-3.4	15.5	55.9	C18 H16 N9 O5 S
470.0977	0.2	0.4	4.5	174.1	C17 H28 N O8 S3
470.0982	-0.3	-0.6	10.5	82.8	C17 H20 N5 O9 S
470.0968	1.1	2.3	5.5	117.5	C16 H24 N O13 S
470.0977	0.2	0.4	10.0	140.7	C16 H22 N8 O3 S3
470.0982	-0.3	-0.6	16.0	73.0	C16 H14 N12 O4 S
470.0964	1.5	3.2	5.0	182.1	C15 H26 N4 O7 S3
470.0968	1.1	2.3	11.0	103.1	C15 H18 N8 O8 S
470.0964	1.5	3.2	10.5	147.6	C14 H20 N11 O2 S3
470.0968	1.1	2.3	16.5	93.2	C14 H12 N15 O3 S
470.1002	-2.3	-4.9	0.5	181.2	C13 H28 N O13 S2
470.0997	-1.8	-3.8	0.0	334.9	C12 H30 N4 O7 S4
470.1002	-2.3	-4.9	6.0	149.7	C12 H22 N8 O8 S2
470.0989	-1.0	-2.1	1.0	202.1	C11 H26 N4 O12 S2
470.0997	-1.8	-3.8	5.5	289.7	C11 H24 N11 O2 S4
470.1002	-2.3	-4.9	11.5	122.5	C11 H16 N15 O3 S2
470.0984	-0.5	-1.1	0.5	345.9	C10 H28 N7 O6 S4
470.0989	-1.0	-2.1	6.5	169.6	C10 H20 N11 O7 S2
470.0973	0.6	1.3	-1.5	443.1	C H20 N13 O14 S
470.0982	-0.3	-0.6	3.0	356.8	C H18 N20 O4 S3
470.0987	-0.8	-1.7	9.0	350.6	C H10 N24 O5 S
